# Mobile health interventions for African adults with hypertension: Evidence from a systematic review of implementation research outcomes

**DOI:** 10.1371/journal.pdig.0001612

**Published:** 2026-08-14

**Authors:** Priyanka Lanka, Ayomide Enitan, Angela Makgabo, Sai Sravanthi Rompicharla, Afnan Alsufyani, John Patena, Dorice Vieira, Joyce Gyamfi, Emmanuel Peprah

**Affiliations:** 1 Department of Global and Environmental Health, School of Global Public Health, New York University, New York, New York, United States of America; 2 ISEE (Implementing Sustainable Evidence-based interventions through Engagement) Lab, School of Global Public Health, New York University, New York, New York, United States of America; Iran University of Medical Sciences, IRAN, ISLAMIC REPUBLIC OF

## Abstract

Africa faces a disproportionate burden of hypertension, with a prevalence of 27% compared to 18% in the Americas. Despite cost‐effective lifestyle and medical interventions, the region suffers from poor detection, treatment, and control rates. Mobile health (mHealth) interventions show promise for hypertension management, however, implementation outcomes in African countries remain underexplored. To fill this gap, this systematic review assessed the acceptability, feasibility, and adoption rates of mHealth interventions among adults diagnosed with hypertension in Africa and identified various facilitators and barriers to its implementation. The review was conducted across nine databases, adhering to the World Bank’s classification of low and middle-income countries (LMICs) and the Preferred Reporting Items for Systematic Reviews and Meta-Analyses (PRISMA) checklist. The search was conducted in October 2023 and updated in January 2024 with no date restrictions. Studies evaluating mHealth interventions for hypertension management among African adults, using experimental, observational, or qualitative designs, and reporting Proctor’s implementation outcomes were included. Studies involving individuals without diagnosed hypertension and pregnant women with preeclampsia, and African population living outside Africa were excluded. Risk of bias was evaluated using the ASSESS tool (A comprehenSive tool to Support rEporting and critical appraiSal of qualitative, quantitative, and mixed methods implementation reSearch Outcomes). Data was extracted independently in Covidence and conflicts were resolved by consensus. In the context of contemporary research practices, it is important to note that no large language models or other AI systems were utilized in the development of this paper. We found eighteen articles focusing on mHealth interventions for hypertension in Africa, exploring acceptability (n = 10), feasibility (n = 11), adoption (n = 4), and appropriateness (n = 2). Service outcomes including effectiveness (n = 3), efficacy (n = 1), patient-centeredness (n = 1), and scalability (n = 2) were also examined. Acceptability and effectiveness were the most frequently reported outcomes. However, feasibility challenges, mainly financial constraints, and cellular network issues, were prominent. Most studies were urban-centric, indicating a gap in understanding rural challenges. Risk of bias was low across all the included studies. Application-based mHealth interventions appears to have gained significant utilization in Africa, particularly in countries like Nigeria, Ghana, and South Africa, while SMS-based interventions show promise in reaching non-smartphone users. Despite successes, feasibility challenges persist, necessitating targeted interventions for financial constraints and network infrastructure. The limitations of this systematic review include exclusion of hypertension with comorbidities beyond diabetes and stroke. Moreover, most studies were confined to urban areas potentially limiting the scalability of these interventions in rural and non-urban regions in resource-constrained areas. Future research should aim to bridge the urban-rural gap and explore innovative solutions to enhance the feasibility, adoption, scalability, and sustainability of mHealth interventions for hypertension management across diverse African settings.

## 1. Background/Introduction

Hypertension, defined as an average of repeatedly measured systolic blood pressure ≥ 130 mmHg and or diastolic blood pressure ≥80 mm Hg [1], affects over 1.28 billion adults aged 30–79 years globally, with at least two-thirds (857 million) accounting for people living in low- and middle-income countries (LMICs) [2]. Africa is disproportionately burdened with hypertension compared to other LMICs, with the World Health Organization estimating the highest prevalence of hypertension (27%) in the region compared to prevalence rates of 18% in the Americas [2]. Despite cost‐effective lifestyle and medical interventions for hypertension control, with potential to avoid unnecessary death and disability, the African region still bears a very high disease prevalence, with poor rates of detection, treatment, and control.

Hypertension is the leading preventable risk factor for cardiovascular diseases (CVD) and disability globally [3]. The largest number of deaths attributed to elevated systolic BP are linked to ischemic heart disease (4.9 million), ischemic stroke (1.5 million), and hemorrhagic stroke (2 million) [4]. Notably, there is a significant rise in stroke prevalence in sub-Saharan Africa, with hypertension serving as a highly modifiable risk factor [5]. Many studies on interventions for hypertension management include patients with stroke due to the direct impact of elevated BP on stroke incidence [5]. Additionally, diabetes is frequently considered in these studies because it shares common risk factors with hypertension, such as obesity, physical inactivity, and unhealthy dietary habits [6]. Our primary outcome for this systematic review was assessing hypertension management. However, we did not exclude studies that reported on patients with hypertension and other comorbid conditions (e.g., stroke and diabetes), as hypertension is an underlying risk factor for several diseases. We did, however, omit inclusion of preeclampsia. The incidence of preeclampsia, which is exacerbated by hypertension in pregnant women, is significantly higher in Africa (“10% of all pregnant women in Africa suffer from pregnancy induced hypertension compared to the global average of 2 percent”) [7]. Preeclampsia, has a complex disease pathology [7], and addressing preeclampsia as a significant research gap which is beyond the scope of this systematic review.

Evidence-based interventions (EBI) exists for hypertension management including educational initiatives to improve patients hypertension knowledge, medication adherence strategies, medical team-based approaches to increase access to preventative care, and community-based screening and self-monitoring [8]. The use of community- and peer-based interventions seems especially promising for reaching underserved populations [9]. Furthermore, mobile health (mHealth) is one such intervention that offers a potential approach to managing chronic conditions in LMICs. mHealth is a subset of digital health and encompasses a wide range of mobile and wireless technologies to help improve health care and overcome barriers such as limited access to healthcare facilities and resources [10]. It has demonstrated efficacy in enhancing patient–provider communication and supporting disease management through various channels. This includes the dissemination of healthy lifestyle information via Short-Message-Service (SMS), utilizing mHealth applications (mHealth Apps) for remote monitoring of blood pressure readings to monitor patients’ progress, sending reminders for medication schedules to enhance treatment plan adherence, and providing tele/remote consultations for patients who cannot physically visit a healthcare facility for in-person consultations with a healthcare provider [10]. The adoption and application of information and communications technology (ICT) tools are increasingly viewed as a promising opportunity to enhance health systems in various countries. Notably, the prevalence and penetration of mobile technologies, especially mobile phones in LMICs, have proven to be a major ingredient needed to improve healthcare service delivery [11], although its sustainability is challenging due to internet connectivity challenges in resource-constrained settings.

In 2021, the number of mobile phone users worldwide stood at 7.1 billion, with forecasts suggesting this is likely to rise to 7.49 billion by 2025 [12]. Within continents, there is country-to-country variability, but penetration in some LMICs exceeds 100% [13]. With the basic connectivity infrastructure in place, the integration of mobile technology into the healthcare system may be a feasible way to complement and improve current chronic disease management strategies in LMICs. According to Gupta et al, 2014, the widespread adoption of mobile technology is positively impacting the African region, as it is being utilized to address health problems [14].

To date, relatively few studies address usability and feasibility issues from an African context [15]. With Africa facing a disproportionately high burden of hypertension, contributing significantly to the region’s overall disease profile, understanding the acceptability, feasibility, and adoption of mHealth interventions is vital for addressing this prevalent health issue. Many regions in Africa also experience challenges related to limited access to healthcare services due to compromised health care systems with scarce human and material resources [16]. mHealth interventions have the potential to bridge gaps in accessibility, thus offering a feasible solution for reaching a broader population and providing timely hypertension management. Assessing the acceptability of mHealth interventions ensures that healthcare approaches align with patients’ preferences and needs. Tailoring hypertension management to be patient-centric enhances engagement, self-efficacy, and adherence, thus contributing to improved health outcomes. Understanding the feasibility and adoption rates of mHealth interventions in Africa will contribute to health system strengthening. Identifying potential barriers and facilitators to implementation will allow for strategic planning and resource allocation, optimizing the integration of mHealth into existing healthcare structures.

We hypothesize that widespread implementation and adoption of EBI including mHealth technologies can facilitate management of hypertension in African patients improving health outcomes which could potentially address the disease burden. This systematic review addresses this knowledge gap of the utilization of mHealth for hypertension management by examining the acceptability, adoption, and feasibility of evidence-based mHealth interventions within the African context. This reviews aims to characterize the factors affecting the successful implementation of mHealth interventions using Proctor’s framework on implementation research outcomes to provide insights, contributing factors, facilitators and barriers, as well as limitations that may exist in the implementation and scale-up of mHealth solutions for hypertension management amongst adults [17]. According to Proctor et al., [17] acceptability is characterized by the perception among implementation stakeholders (including beneficiaries and implementers) that the innovation is agreeable, palatable, or satisfactory. Adoption is defined as the intention, initial decision, or action to try or employ the innovation (i.e., uptake). Lastly, feasibility is defined as the extent to which the innovation can be successfully utilized or carried out within a given agency or setting [17]. In the context of Africa, acceptability, feasibility, and adoption are crucial for the long-term sustainability and scalability of evidence-based interventions, particularly for hypertension, which has a significant impact across the continent.

## 2. Methods

### Search strategy

This systematic review was registered with OSF (https://doi.org/10.17605/OSF.IO/F7DHE) on January 19, 2024. We developed an extensive search strategy to locate published studies across nine databases employing the Preferred Reporting Items for Systematic Reviews and Meta-Analysis (PRISMA) [18] and the World Bank [19] criteria to delineate LMICs (S1 and S2 Files). Our search strategy did not impose any date restrictions. The following databases were searched: OVID Medline, PubMed, Embase, Engineering Village, Cochrane Library, Global Health, Web of Science, PAIS (Public Affairs Information Service), and APA PsycInfo. We developed a set of comprehensive search syntax specific for each database including key terms and their Medical Subject Heading (MeSH) for ‘hypertension’, ‘high blood pressure,’ ‘mobile health,’ and ‘implementation outcomes’ according to Proctor et al [17] and ‘Africa’ as defined by the World Bank. The search was conducted in October 2023 and updated in January 2024. A sample of the search strategy is provided in supporting information (S3 File).

### Inclusion and exclusion criteria

The articles were included if they met the following criteria: 1) studies addressing hypertension management (either on treatment, screening, referral to care, treatment adherence) using experimental, observational or qualitative study designs including case studies and preprints, 2) study population diagnosed with hypertension according to AHA guidelines which defines Hypertension as an average of repeatedly measured systolic blood pressure ≥ 130 mmHg and or diastolic blood pressure ≥80 mm Hg with or without comorbidities (e.g., stoke or diabetes) [1] 3) studies that investigate the use of mHealth intervention in the management of hypertension, 4) all studies had to be conducted in Africa and 5) utilized Proctor’s taxonomy for implementation outcomes (Tables 1 and 2).

**Table 1:** Implementation outcomes and their definitions according to Proctor et al. [17].

Outcome	Definition
Acceptability	The perception among implementation stakeholders (beneficiaries and implementers) that the innovation is agreeable, palatable or satisfactory.
Adoption	The intention, initial decision, or action to try or employ the innovation (i.e., uptake).
Appropriateness	The perceived fit, relevance or compatibility of the innovation for a given practice setting, provider, or beneficiary; and/or perceived fit of the innovation to address a particular issue or problem (therapeutic use of hydroxyurea for sickle cell disease).
Cost	(Incremental or implementation cost) is defined as the cost impact of an implementation effort.
Feasibility	The extent to which the innovation can be successfully used or carried out within a given agency or setting.
Fidelity	Degree to which the innovation can be implemented as it was prescribed in the original protocol or as it was intended by the programme developer.
Penetration	The integration of a practice within a service setting and its subsystems.
Sustainability	The extent to which a newly implemented innovation is maintained or institutionalized within a service setting’s ongoing, stable operations.

**Table 2:** Service outcomes and their definitions according to Proctor et al. [17].

Outcome	Definition
Efficiency	The avoidance of waste, including waste of equipment, supplies, ideas and energy.
Safety	The avoidance of injuries to patients from the care that is intended to help them.
Effectiveness	Provision of services based on scientific knowledge to all who could benefit and refraining from providing services to those not likely to benefit (avoiding underuse and overuse, respectively).
Equity	Provision of care that does not vary in quality because of personal characteristics such as gender, ethnicity, geographical location and socioeconomic status.
Patient-centeredness	Provision of care that is respectful of and responsive to individual patient preferences, needs, and values and ensuring that patient values guide all clinical decisions.
Timeliness	Reduction of waits and sometimes harmful delays for both those who receive and those who give care.

Studies were excluded if they met the following criteria: 1) studies that included African adults not diagnosed with hypertension 2) studies related to Preeclampsia amongst pregnant women. 3) Studies conducted amongst African populations living abroad, 4) studies not addressing hypertension management amongst adults, and 5) systematic reviews, abstracts, protocols, and commentaries (S4–S6 Files).

Resulting articles were imported into a citation management tool, Endnote, followed by a screening and data extraction tool for conducting systematic reviews, Covidence, where duplicates were removed. Title and abstracts for all articles were independently screened by a minimum of two reviewers (PL, AE, AM, SR, AA) and conflicts were resolved by a third reviewer manually assigned within Covidence. Full text reviews were then conducted on the screened articles and relevant information was extracted by 4 reviewers (PL, AE, AM, SR). Each article underwent extraction based on the following variables: study design, study location, implementation of mhealth (SMS or Application based), implementation outcomes outlined by Proctor et al., [17] encompassing Adoption, Appropriateness, Acceptability, Cost, Feasibility, Fidelity, Penetration, and Sustainability and finally facilitators and barriers for the implementation of the evidence-based mHealth intervention. Service outcomes outlined by Proctor et al., [17] Efficiency, Safety, Effectiveness, Equity, Patient centeredness and Timeliness were also assessed. For this review, acceptability, adoption, and feasibility of mHealth interventions within the African context were the main focus (S7 File).

### Inter-rater agreement

Inter-rater reliability during the title and abstract screening stages was assessed using Cohen’s kappa coefficient, calculated for reviewer pairs to quantify agreement beyond chance. Kappa values were interpreted using established benchmarks (e.g., slight, fair, moderate, substantial agreement), and proportionate (raw) agreement was also examined to aid interpretation, particularly in the presence of highly imbalanced inclusion decisions (S2 Table). Discrepancies were resolved through consensus discussion, with adjudication by a third reviewer when necessary. Although kappa values were lower during the title and abstract screening stage, this likely reflects the broad eligibility criteria and low prevalence of studies meeting inclusion criteria, both of which can influence kappa estimates despite high raw agreement. Reviewer agreement improved during full-text screening, indicating greater consistency in the application of eligibility criteria at later stages of study selection.

### Quality assessment

We employed the ASSESS tool (A comprehenSive tool to Support rEporting and critical appraiSal of qualitative, quantitative, and mixed methods implementation reSearch Outcomes)[20] to evaluate the risk of bias in the study. ASSESS is a 24 item tool that standardizes the synthesis and reporting of implementation efforts and describes studies evaluating implementation research outcomes. Two reviewers (PL and AM) individually assessed the risk of bias of each study that met the inclusion criteria. Conflicts were resolved via discussion and overseen by the medical librarian (DV). Each criterion was used to assess the study, and an aggregate score was calculated to categorize studies into high or low bias. A score of 1–2 indicate high risk of bias and 3–5 indicate low risk of bias, or unclear (unable to be assessed) (S3 Table and Fig 2).

**Fig 1:**
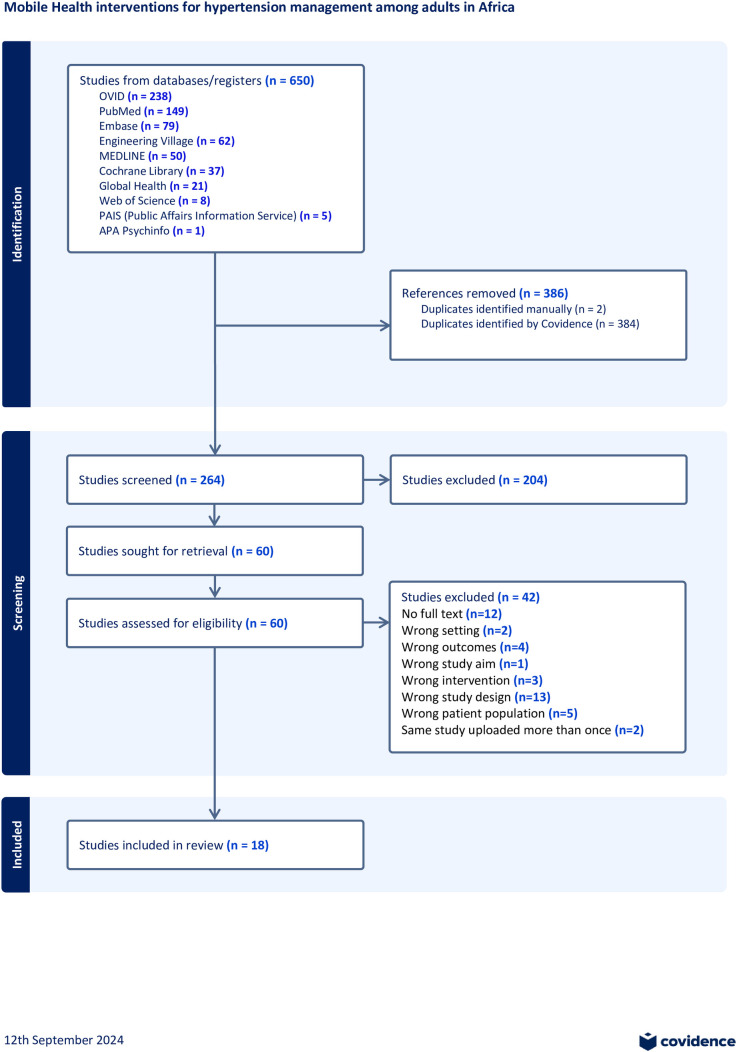
PRISMA flow chart illustrating the study selection process. PRISMA, Preferred Reporting Items for Systematic Reviews and Meta-Analyses.

**Fig 2:**
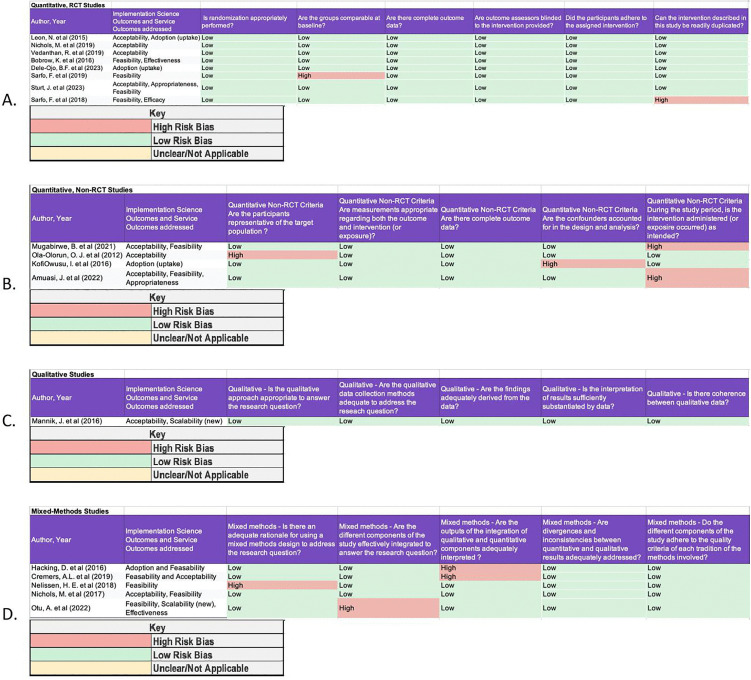
Risk of Bias Assessment of the included studies using the ASSESS tool.

## 3. Results

### Literature search

The initial database search produced 650 articles, resulting in 265 remaining after the elimination of 385 duplicates. Upon conducting a more detailed review of the abstract and title screening process, we identified 204 articles as irrelevant. These exclusions were based on criteria aligned with our research parameters, including studies conducted on African populations outside of Africa, studies lacking mHealth as part of their intervention, study designs such as study protocols, and conference abstracts. Sixty articles were further assessed under full text review out of which ultimately 18 articles were found to be relevant to the review topic. All articles included in the final review and data extraction were recently published (ranging from 2012 to 2023). Our review comprised randomized control trials (n = 8) [21–28], qualitative assessments (n = 1) [29], quantitative assessments (n = 4) [30–33], and mixed methods studies (n = 5) [5,34–37]. We visually outlined the data extraction process through a PRISMA flowchart, developed using the Covidence search and data extraction tool (Fig 1). Inter-rater reliability at the title and abstract screening stage was generally low and variable (Cohen’s κ = –0.13 to 0.37), with high raw agreement in some pairs but negative or near-zero Kappa values reflecting skewed exclusion decisions and highlighting greater subjectivity compared to the full-text review stage (S2 Table).

### Characteristics of identified studies

#### I. Demographic and study duration characteristics.

Characteristics of the studies reviewed are summarized in Table 3. All 18 included studies targeted adult populations, encompassing both men and women with varying age criteria. The mean duration of the studies was approximately eleven months, ranging from a minimum of one month to a maximum of thirty-two months.

**Table 3:** Characteristics of the studies.

Author (year)	Design	Study duration	Country	Sample(N)	Intervention Type	Setting	Key Implementation Outcomes	Other outcomes (e.g., client, service and clinical outcomes)
Amuasi et al., (2022) [30]	Cross sectional- Quantitative study	9 months	Ghana	4000	**mHealth App**	Urban	Feasibility, Acceptability	Appropriateness
Bobrow, K., (2016) [21]	RCT	5 months	South Africa	1372	**SMS**	Urban	Feasibility	Effectiveness
Cremers, A.L. et al.,(2019) [34]	Mixed Methods study	13 months	Nigeria	30 + 328	**mHealth App**	Urban	Feasibility and Acceptability	NR
Dele-Ojo et al., (2023) [22]	RCT	6 months	Ghana and Nigeria	225	**mHealth App**	Urban	Adoption	NR
Hacking,D.,et al (2016) [35]	Mixed Methods study	17 weeks	South Africa	223	**SMS**	Urban	Adoption and Feasibility	Effectiveness
KofiOwusu (2016) [31]	Cohort - Quantitative study	6 months	Ghana	150	**mHealth App** The model consisted of a mobile tablet, an automated blood pressure machine, and a novel software application for longitudinal patient management.	Urban	Adoption	NR
Leon, N. et al., (2015) [23]	RCT	12 months	South Africa	22	**SMS**	Urban	Acceptability and Adoption	NR
Mannik, J. et al., (2016) [29]	Community screening (qualitative study)	22 months	Rural Kenya	2865	**SMS**	Rural	Acceptability	Scalability
Mugabirwe, B. et al., (2021) [32]	Cohort - Quantitative study	30 days	Uganda	37	**mHealth App**	Rural	Acceptability Feasibility	NR
Nelissen, H. E.et al., (2018) [36]	Mixed Methods study	6-8 months	Nigeria	336	**mHealth App**	Urban	Feasibility	NR
Nichols et al., (2017) [5]	Mixed Method study	Not mentioned	Ghana	200	**mHealth App** Bluetooth enabled blood pressure monitor, smartphone app, and SMS	Urban	Acceptability, Feasibility	NR
Nichols, M. et al., (2019) [24]	RCT	3 months	Ghana	16	**mHealth App** Phone-based Intervention under Nurse Guidance (PINGS)	Urban	Acceptability	NR
Ola-Olorun, O. J. et al., (2012) [33]	Cross sectional – Quantitaive study	8 months	Nigeria	117	**SMS**	Urban	Acceptability	Patient-centeredness
Otu, A et al., (2022) [37]	Mixed Methods	12 months	Nigeria	24	**mHealth App** mHealth training module (video & text based) delivered via android tablets and group support via Whatsapp	Peri-Urban	Feasibility	Scalability and Effectiveness
Sarfo et al., (2018) [25]	RCT	3 months	Ghana	60	**mHealth App-** Bluetooth enabled smartphone	Urban	Feasibility	Efficacy
Sarfo et al., (2019) [26]	RCT	9 months	Ghana	60	**mHealth App** - Bluetooth link to blood pressure apparatus	Urban	Feasibility	NR
Sturt et al., (2023) [27]	RCT	12 months	Nigeria and Tanzania	20 clusters (100/ cluster)	**mHealth App**	Urban	Acceptability and Feasibility	Appropriateness
Vedanthan et al., (2019) [28]	RCT	32 months	Western Kenya	1460	**SMS**	Rural	Acceptability	NR

#### II. Geographical distribution and setting.

Fourteen studies predominantly collected data in urban settings [5,21–27,30,31,33–36], three in rural areas [28,29,32], and one in a peri-urban [37] location. Notably, 39% of the 18 studies were conducted in Ghana [5,22,24–26,30,31], 33% in Nigeria [22,27,33,34,36,37], and 17% in South Africa [21,23,35], with the remaining studies in Kenya [28,29], Uganda [32] and Tanzania [27].

#### III. Target population and health focus.

All the experimental research concentrated on subjects with elevated blood pressure and confirmed hypertension. The studies aimed to enhance hypertension control, treatment adherence, health knowledge, or assess the effectiveness of mHealth tools in hypertension management. Comorbidity considerations were present in five studies involving patients with stroke and hypertension [5,24–26,30], one study involving diabetes mellitus [37] and one involving patients with HIV [32].

#### IV. Technological interventions.

Mobile phone technology, including SMS and mobile Apps, was uniformly utilized across all studies. Our findings revealed successful strategies employing SMS to improve adherence behaviors and a high receptivity towards mHealth apps for blood pressure monitoring. However, some articles identified concerns related to patients’ ability to use technology, comprehend relayed information, and inconsistencies in network connectivity [22,24,26,35].

#### V. Study design and technological approaches.

Study designs comprised randomized controlled trials (RCTs) 8 out of 18 studies (44%) [21–28], 5 (28%) mixed methods [5,34–37], 4 (22%) quantitative [30–33], and 1 (6%) qualitative studies [29]. App-based technology was predominant in over 12 out of 18 (67%) of the studies [5,22,24–27,30–32,34,36,37], while 6 studies (33%) utilized SMS-based interventions [21,23,28,29,33,35].

### Implementation outcomes

Acceptability and feasibility was the most common reported implementation outcome throughout all the studies. Approximately 61% of the 18 studies focused on feasibility [5,21,25–27,30,32,34–37], 56% on acceptability [5,23,24,27,29,30,32–34], 22% on adoption [22,23,31,35], 17% effectiveness [21,35,37], and 11% on appropriateness [27,30]. Furthermore, approximately 11% of the studies reported on scalability [29,37]. The latter is defined and reported according to the WHO ExpandNet definition, as ‘deliberate efforts to increase the impact of successfully tested health innovations, to benefit more people and to foster policy and program development on a lasting basis’ [38]. None of the studies discussed fidelity, penetration, and sustainability. In the collective analysis of the nine articles that evaluated acceptability, both participants and healthcare providers expressed noteworthy satisfaction with the mHealth intervention.

Most of the participants demonstrated a high level of comfort with the SMS reminders and mobile Apps utilized for blood pressure monitoring. Particularly, stroke patients and their caregivers exhibited an overwhelming positive response, citing the intervention’s role in enhancing medication adherence, heightening awareness of blood pressure control, fostering improved dietary practices, and encouraging stress reduction [26]. The overall sentiment suggests a robust acceptance and appreciation for the mHealth strategies employed, affirming their positive impact on both patients and their caregivers. The eleven articles that evaluated feasibility revealed that the accessibility of mobile phones played a crucial role in the feasibility of adopting mHealth interventions. Additionally, aligning the intervention with accessible pharmacies or involving nurses in the triaging process emerged as factors that could further enhance feasibility. The four studies assessing the adoption rates advocated for addition of mHealth to the usual care among patients with hypertension which would lead to an increase in the proportion of those with good blood pressure control [22,23,31,35]. Effectiveness, as evaluated by three studies, revealed that participants perceived the SMS messages positively. Rather than being seen as merely conveying new information, the SMS messages were regarded as motivational reminders for promoting behavioral change. Participants described the SMS messages as “trustworthy” and “caring,” creating a sense of being looked after, which contributed to their overall positive reception and effectiveness in encouraging behavioral adjustments [21,35,37]. A study measuring acceptability and patient centeredness revealed that their intervention garnered positive perceptions from the involved patients, and the assessment of patient-centeredness highlighted a strong inclination towards the chosen means of communication [33].

Participants demonstrated a strong perception of the intervention’s usefulness and showed a positive inclination towards its adoption within the realm of mHealth. Notably, a distinctive exploration of its scalability by Mannik, J. et al (2016) [29] stands out in this context. This study delved into the scalability of a novel mHealth tool named AFYACHAT, derived from ‘afya,’ meaning health in Kiswahili (Kenya’s national language). The research concluded that the innovative tool holds the potential to empower Community Health Workers (CHWs) in efficiently screening large numbers of patients in rural African communities for CVD risk. The AFYACHAT system gained widespread acceptance among the target population and underscores the scalability of mHealth intervention in conjunction with the extensive reach of CHWs across rural Africa, suggesting its capacity for significant and far-reaching public health impact.

Studies assessing the feasibility, acceptability, and appropriateness of a mHealth intervention from the perspective of health workers, PINGS (Phone-based Intervention under Nurse Guidance after Stroke) was also found to be feasible, acceptable, and suitable for blood pressure control among stroke survivors in Ghana [24,26]. One study also demonstrated the feasibility of implementing mHealth interventions for post-stroke care in resource-limited settings [30]. Finally, another study emphasized the feasibility of implementing of task-shifting strategies and culturally tailored mHealth interventions in enhancing post-stroke care in sub-Saharan Africa [25].

Of the 18 included studies, a subset reported clinical outcomes alongside implementation outcomes. Dele-Ojo et al., (2023) found that mHealth integration with usual care produced a significant increase in blood pressure control, directly supporting the adoption findings. Sarfo et al. (2018, 2019) reported improvements in blood pressure levels among stroke survivors in their PINGS RCTs, lending clinical weight to the feasibility and acceptability data documented in those studies. Bobrow et al., (2016) demonstrated that SMS-based interventions reduced systolic blood pressure compared to control, underlying the feasibility findings from that trial. Kofi Owusu et al., (2016) reported a significant decrease in systolic blood pressure and an 82% improvement in health awareness, reinforcing the adoption evidence. In contrast, studies that reported implementation challenges—particularly around network connectivity and technology literacy (Sarfo et al., 2019; Cremers et al., 2019)—did not consistently demonstrate clinical improvements, suggesting that feasibility barriers may have limited clinical impact. Studies that reported only qualitative or descriptive implementation data without clinical endpoints (e.g., Nichols et al., 2017; Mannik et al., 2016) limited authors ability to conclude whether high acceptability translated to clinical benefit

### Facilitators and barriers to implementing mHealth technology in hypertension management in Africa

Tables 4 and 5 presents the barriers and facilitators influencing the implementation of mHealth for hypertension management in the African context reported in the cited publications. Facilitators included Africa’s rapidly growing cell phone market enabling broad SMS reach, localization and personalization of messages to enhance adoption, behavioral theory based mobile phone adherence interventions, increased awareness of hypertensions chronic nature, high quality pharmacist training supporting task shifting, and culturally relevant, cost effective interventions leveraging existing infrastructure for sustainability (Table 4). Barriers identified were widespread misconceptions about hypertension symptoms leading to emotional distress and disengagement, rejection or high cost of antihypertensive drugs, technology related challenges such as poor internet connectivity and digital insecurity among older adults, socioeconomic and financial constraints limiting access to care and mobile devices, proximity barriers reducing use of remote consultations, and overburdened healthcare systems with high patient to physician ratios necessitating task-shifting strategies (Table 5).

**Table 4:** Facilitators to implement mHealth technology in hypertension management in Africa.

Facilitators	Description
**Growing Cell Phone Market in Africa**	Africa’s rapidly expanding cell phone market, with over 75% ownership among the low-income South African population, facilitates the reach of SMS interventions [12].
**Localization of SMS Messages**	SMS messages translated into local provincial languages potentially contributed to increased adoption. Personalization and tailoring of SMS messages to individual recipients show a marked improvement in outcomes [26].
**Mobile Phone-Based Adherence Interventions**	Implementation of mobile phone-based adherence interventions grounded in established behavior change theories can be more effective in promoting long-term adherence to treatment. [23]
**Awareness of Chronic Nature of Hypertension**	Recognition of hypertension’s chronic nature and the necessity of continuous drug intake, as well as maintaining a healthy lifestyle (healthy nutrition and physical activity) [24].
**Quality Training of Pharmacists**	Five-year training of pharmacists is of high quality, facilitating task shifting. Frequent booster training also helps with Knowledge retention [33].
**Culturally Relevant and Cost-Effective Interventions**	Culturally tailored and relevant, cost-effective interventions built upon existing infrastructure for expediency, and sustainability [26,39].

**Table 5:** Barriers to mHealth technology implementation for hypertension management in Africa.

Barriers	
**General Belief in Hypertension Symptoms**	Emotional distress along with fear and frustration from seeing consistently high blood pressure readings posed as a barrier and reduced engagement with the intervention [32].
**Rejection of Cheaper Local Hypertensive Drugs**	Rejection of cheaper local hypertensive drugs by some individuals [21,32].
**High Cost of Anti-hypertensive Drugs**	High cost of anti-hypertensive drugs acts as a barrier [32].
**Technology-Related Concerns**	Some patients, especially older ones, may experience increased insecurity due to technology. - Unstable internet connection as a technological barrier [24,26,35].
**Socioeconomic Disparities**	Differences in monthly income and education level contribute to disparities [22].
**Proximity to Care Issues**	Proximity to care prevents patients from resorting to remote consultations [27].
**Financial Limitations**	Financial constraints may hinder owning a cellphone or affording hypertensive medications [22,23].
**High Patient to Physician Ratios**	High patient to physician ratios can be addressed by substituting physicians with Community Health Workers (CHWs) and Pharmacists or Nurses for remote monitoring and intervention design. - CHWs and healthcare professionals play a key role in promoting medication adherence and behavior change through remote interventions. Task-shifting strategies can be employed [25].

### Quality assessment findings

The ASSESS tool [20] was instrumental for evaluating the risk of bias within the included studies. It provided a standardized and systematic approach for identifying potential biases, ensuring consistency across the assessments. The risk of bias (RoB) was evaluated for the four study designs represented in this review: quantitative randomized controlled trials (RCTs; *n* = 8), quantitative non-RCTs (*n* = 4), qualitative studies (*n* = 1), and mixed-methods studies (*n* = 5). Among the eight quantitative RCTs, six studies demonstrated low RoB across all six domains assessed, while two studies exhibited high RoB in one domain each. For the four quantitative non-RCTs, each study showed high RoB in one of the five domains, with overall low RoB ratings across the remaining domains. The single qualitative study had low RoB across all five domains assessed. Among the five mixed-methods studies, four had high RoB in one domain while maintaining low RoB in the remaining domains; one study demonstrated low RoB across all domains. (Tables 6 and S3 and Fig 2 and S4 File).

**Table 6:** Risk of Bias Assessment (summary) [20].

Article	Score	Degree of Bias
Amuasi et al. (2022) [30]	4	Low bias
Bobrow, K. et al. (2016) [21]	5	Low bias
Cremers, A.L. et al (2019) [34]	4	Low bias
Dele-Ojo et al. (2023) [22]	5	Low bias
Hacking, D. et al (2016) [35]	4	Low bias
KofiOwusu et al. (2016) [31]	4	Low bias
Leon, N. (2015) [23]	5	Low bias
Mannik, J. et al (2016) [29]	5	Low bias
Mugabirwe, B. et al (2021) [32]	4	Low bias
Nelissen, H. E. et al (2018) [36]	4	Low bias
Nichols et al. (2017) [5]	5	Low bias
Nichols, M. et al (2019) [24]	5	Low bias
Ola-Olorun, O. J. et al (2012) [33]	4	Low bias
Otu, A. et al. (2022) [37]	4	Low Bias
Sarfo et al. (2018) [25]	4	Low Bias
Sarfo et al. (2019) [26]	4	Low Bias
Sturt et al. (2023) [27]	5	Low Bias
Vedanthan et al (2019) [28]	5	Low bias

## 4. Discussion

Our analysis of a diverse range of studies on mHealth interventions for hypertension in African countries reveals significant trends and considerations. These trends have been grouped into facilitators and barriers that can support or impede the successful implementation of evidence-based mHealth interventions in Africa (Table 3).

A primary driver of mHealth adoption is the increasing availability and usage of mobile phones in Africa. This high mobile penetration rate enhances the feasibility of mhealth interventions across the continent [12]. For acceptability, much of the research focused on patient’s attitudes towards receiving health information via SMS or calls that can be supported on currently available devices in Africa. Studies showed that mHealth interventions are acceptable to patients, particularly when delivered in local languages [23]. This highlights the importance of cultural sensitivity and localization in maximizing the acceptability of mHealth interventions.

Adoption, defined as the intention, initial decision, or action to try or employ an innovation or EBI or practice [17] showed promising results. Elevated adoption rates signify the successful integration of technology, enhancing its potential for widespread impact and opportunity for scale-up. Several studies have demonstrated the positive impact of mHealth interventions on hypertension management. Study conducted by Dele-Ojo et al., [22] found that integrating mHealth with usual care among patients with hypertension led to significant increase in blood pressure control in the target population. Similarly, Kofi Owusu et al., [31] demonstrated that the adoption of the Akoma Pa program resulted in substantial blood pressure reduction across the entire participant cohort, with an impressive 82% improvement in overall health awareness and a 95% participant retention rate. While this study lacked a control group, the significant decrease in systolic blood pressure, particularly among those with initially uncontrolled blood pressure, is noteworthy. Moreover, Mugabirwe et al., [32] demonstrated an increase in medication adherence among adult participants receiving clinical care for hypertension in both peri-urban and rural settings, suggesting the generalizability of these findings to other low-income countries with comparable mobile phone usage.

Overall, where clinical indicators were reported alongside positive implementation outcomes, the evidence was broadly supportive; however, many studies lacked clinical outcome data, limiting the extent to which implementation success can be interpreted as clinically meaningful.

Despite promise of mHealth, several challenges hinder the widespread adoption and effectiveness. Technological barriers remain significant. Unreliable network services, data privacy concerns, and low digital literacy can hinder seamless implementation as well as reduce the acceptability and adoption of mHealth technologies due to fear and mistrust. For example, some studies noted that older population’s unfamiliarity with technology posed a barrier [34]. Sarfo et al., [26] highlighted patients’ inability to use technology and comprehend information conveyed as a significant challenge. According to the Global System for Mobile Communications report, smartphones are becoming more common, and are predicted to reach 61% by 2025 [40]. However, the problem exists that nearly 40% of people in Sub-Saharan Africa use basic cell phones or have no mobile phone access. This digital divide necessitates careful consideration when designing and implementing mHealth interventions.

Beyond technology, patient related barriers also exist. A significant barrier to adoption of mHealth interventions is the misconceptions that hypertension is only problematic when symptomatic, undermining proactive mHealth engagement. Addressing these beliefs through targeted educational components is crucial, as it directly impacts the feasibility and effectiveness of implementing mHealth solutions to address this condition. Furthermore, while our study revealed positive behavioral changes and improvement in hypertension management, most studies lacked comprehensive impact measurement via adherence tracking and long-term clinical outcomes (see S1 Table). For instance, Dele Ojo et al., [22] used Hill-Bone Adherence scale to assess adherence to scheduled clinic appointments during the study period. However, none of the studies suggested the use of such measures beyond the study period. This gap shows the need for more robust evaluation methods to assess the sustained impact of mHealth interventions.

Finally external factors can pose a significant challenge and could greatly influence feasibility and adoption of evidence-based mHealth interventions. Hacking et al., [35] showed that over 29% of SMS deliveries failed due to service provider issues while Sarfo et al., [26] noted inconsistent network connectivity. Mannik, J. et al., (2016) [29] identified issues of power interruptions at clinics housing the central database, as a major obstacle, leading to intermittent system availability. Furthermore, Community Health Workers (CHWs) faced significant challenges sending SMS messages particularly during instances when a sizable group of patients were awaiting screenings. To overcome these limitations, CHWs employed innovative solutions such as recording the results and sending SMS messages at later times. These strategies could be integrated into the initial training of healthcare providers or incorporated directly into the design of the mHealth interventions. No other study reported effective alternatives to prevent healthcare service delivery failures.

This systematic review highlights the potential of mHealth interventions to positively impact both patients and providers in the context of hypertension management in Africa. However, it has also revealed a critical gap in the existing literature. While evidence suggests that mHealth can be effective, there is dearth of research specifically examining the implementation outcomes of EBIs delivered via mHealth for hypertension management in the diverse contexts of Africa. This review, to our knowledge, represents the first attempt, to systematically assess these implementation outcomes. This highlights a crucial area for future research, as a deeper understanding of how effectively implement and scale-up mHealth interventions is essential to harness their full potential in addressing the burden of hypertension across the continent.

### Strengths and limitations

Our systematic review has several important strengths and limitations that should be considered when interpreting the findings. A key strength of this review is that it provides a structured synthesis of implementation outcomes for mHealth interventions targeting adults with hypertension in Africa, offering a clear picture of the current evidence base. The predominance of urban-based studies, while a limitation in terms of generalizability, also reflects where implementation research has been most actively conducted. This concentration of evidence in urban settings allows for more consistent characterization of implementation outcomes, such as acceptability, feasibility, and adoption, within contexts that may have relatively stronger digital infrastructure and health system capacity. As such, the review accurately captures the settings in which mHealth strategies are currently being tested and scaled.

However, several limitations warrant consideration. First, although we did not explicitly focus on hypertension in the presence of comorbid conditions, relevant findings were included when identified. A broader search strategy incorporating common comorbidities (e.g., diabetes or cardiovascular disease) might have provided a more comprehensive perspective. Nevertheless, our primary aim was to characterize the state of mHealth interventions specifically for adults diagnosed with hypertension in Africa. Second, most included studies were conducted in urban settings, with limited representation from rural areas. This imbalance restricts the generalizability of findings to rural and resource-constrained contexts, where digital access, connectivity, device ownership, and digital literacy may differ substantially. The limited representation of rural settings underscores a critical evidence gap rather than a methodological oversight. Third, follow-up durations were generally short to medium term, limiting insight into long-term sustainability and durability of implementation outcomes. Finally, few studies addressed technological disruptions (e.g., SMS failures or app interruptions), highlighting the need for future research to more intentionally examine digital equity, contextual determinants, and system-level resilience to support sustainable scale-up of mHealth interventions.

### Conclusion

Our systematic review is the first to comprehensively characterize the landscape of evidence-based mHealth interventions for managing hypertension in African adults, focusing on implementation outcomes. We assessed these outcomes according to Proctor et al.‘s framework, [17] encompassing adoption, appropriateness, acceptability, cost, feasibility, fidelity, penetration, and sustainability as well as facilitators and barriers to implementation. Our findings indicate that approximately 61% of the 18 studies examined focused on feasibility, 56% on acceptability, 22% on adoption, 17% on effectiveness, and 11% on appropriateness. Notably, none of the studies addressed implementation outcomes such as cost, fidelity, penetration, or sustainability within the African context.

The absence of the above the outcomes can be due to several factors. Resource limitations and a focus on proving feasibility acceptability have taken priority in mHealth research in Africa, particularly as these outcomes are of more immediate concerns in resource-constrained settings. Additionally long-term studies that assess sustainability and fidelity are rare, and challenges such as healthcare disparities, economic constraints, and the digital divide make it difficult to generalize findings across diverse populations. Given Africa’s rapidly growing cell phone market, where over 75% of the low-income population owns or has access to a cell phone, especially in countries like Nigeria, Ghana, and South Africa, mHealth technologies are increasingly viable and future research should address this gap. Furthermore, only 11 percent of studies considered the scalability of these mHealth interventions, which should be an area of focus for future studies to ensure optimal reach in resource-constrained settings.

Several notable facilitators and barriers can influence the scaling up of evidence-based mHealth interventions for reducing hypertension in Africa. Facilitators such as the expanding cell phone market, growing awareness of hypertension, and culturally relevant interventions can promote greater adoption among the population. However, barriers like high medication costs and a lack of urgency in addressing asymptomatic hypertension pose significant challenges.

We found that high acceptability of mHealth interventions is closely linked to their cultural relevance and personalization, while challenges such as mistrust and misconceptions about symptoms persist. Moreover, feasibility of implementing mHealth interventions is bolstered by the expanding cell phone market and cost-effective solutions, yet technical and financial barriers remain. Adoption of the hypertension management programs using mHealth is supported by effective training and proven efficacy, although socio-economic disparities and strains on healthcare systems can limit widespread use. Furthermore, emerging best practices, such as the Patient-Centered Care Model involving patients, pharmacists, and cardiologists, offer promising avenues for improving health outcomes for adults with hypertension. Understanding these factors is crucial for the successful implementation and effectiveness of interventions.

As anticipated, mHealth interventions have proven effective in managing hypertension in European countries and hold promise for improving health outcomes. However, to optimize these interventions for African populations, further targeted research is needed. Future studies should investigate combining ongoing patient education with economic initiatives to enhance access to mobile technologies, including phones and SMS services, across various educational backgrounds. Additionally, increasing patient awareness of hypertension as a serious but often asymptomatic condition is essential. Integrating these elements can enhance the effectiveness of mHealth strategies, ensuring broader adoption and sustained impact, particularly in resource constrained settings.

## Supporting information

S1 Table Clinical assessment of blood pressure outcomes reported across included studies. (XLSX)

S2 Table Covidence inter-rater reliability scores. (XLSX)

S3 Table Risk of Bias Assessment. (XLSX)

S1 File PRISMA 2020 checklist. Reproduced from PageMJ, McKenzie JE, Bossuyt PM, et al. *The PRISMA 2020 statement: an updated guideline for reporting systematic reviews.* Licensed under the Creative Commons Attribution 4.0 International (CC BY 4.0) License.
(DOCX)

S2 File PRISMA 2020 abstract checklist. (DOCX)

S3 File Search strategy used for identifying eligible studies. (DOCX)

S4 File List of excluded and irrelevant articles used during screening. (XLSX)

S5 File Complete list of articles retrieved during database searching. (PDF)

S6 File Included article dataset. (XLSX)

S7 File Final data extraction table. (XLSX)
