# Supplementary material for: Mobile health interventions for African adults with hypertension: Evidence from a systematic review of implementation research outcomes
Source: PLOS Digit Health. 2026 Aug 14;5(8):e0001612. doi: 10.1371/journal.pdig.0001612 (PMC13475905; doi:10.1371/journal.pdig.0001612)
Supplement: Supplementary file 1 [file pdig.0001612.s008.pdf]

1. Turcotte-Tremblay A-M, Leerapan B, Akweongo P, Amponsah F, Aryal A, Asai D, et al. Tracking health system performance in times of crisis using routine health data: lessons learned from a multicountry consortium. Health Research Policy and Systems. 2023;21:1-8.
2. Anderson AC, O'Rourke E, Chin MH, Ponce NA, Bernheim SM, Burstin H. Promoting Health Equity And Eliminating Disparities Through Performance Measurement And Payment. Health Affairs. 2018;37(3):371-7,7A-7F.
3. Coleman R, Gill G, Wilkinson D. Noncommunicable disease management in resource-poor settings: A primary care model from rural South Africa. World Health Organization Bulletin of the World Health Organization. 1998;76(6):633-40.
4. Zullig LL, McCant F, Silberberg M, Johnson F, Granger BB, Bosworth HB. Changing CHANGE: adaptations of an evidence-based telehealth cardiovascular disease risk reduction intervention. Transl Behav Med. 2018;8(2):225-32.
5. Ziegler A, Öner A, Quadflieg G, Betschart RO, Thiéry A, Babel H, et al. Cost-effectiveness of a telemonitoring programme in patients with cardiovascular diseases compared with standard of care. Heart. 2023;109(21):1617-23.
6. Ziegler A, Mann M, Brandewiede B, Dittrich H, Hintz S, Krockenberger K, et al. Statistical analysis plan for the randomized controlled trial CardioCare MV investigating a novel integrated care concept (NICC) for patients suffering from chronic cardiovascular disease. Trials. 2020;21(1):131.
7. Zha P, Qureshi R, Porter S, Chao YY, Pacquiao D, Chase S, et al. Utilizing a Mobile Health Intervention to Manage Hypertension in an Underserved Community. West J Nurs Res. 2020;42(3):201-9.
8. Zelelew AN, Workie DL. Joint Modeling of Blood Pressure Measurements and Survival Time to Cardiovascular Disease Complication among Hypertension Patients Follow-up at DebreTabor Hospital, Ethiopia. Vasc Health Risk Manag. 2023;19:621-35.
9. Zelelew AN, Workie DL. Joint Modeling of Blood Pressure Measurements and Survival Time to Cardiovascular Disease Complication among Hypertension Patients Follow-up at DebreTabor Hospital, Ethiopia. Vascular health and risk management. 2023;19:621-35.
10. Zelelew AN, Workie DL. Joint Modeling of Blood Pressure Measurements and Survival Time to Cardiovascular Disease Complication among Hypertension Patients Followup at DebreTabor Hospital, Ethiopia. Vascular Health and Risk Management. 2023;19:621-35.
11. Zelelew AN, Workie DL. Joint Modeling of Blood Pressure Measurements and Survival Time to Cardiovascular Disease Complication among Hypertension Patients Follow-up at DebreTabor Hospital, Ethiopia. Vascular health and risk management. 2023;19:621-35.
12. Zelelew AN, Workie DL. Joint Modeling of Blood Pressure Measurements and Survival Time to Cardiovascular Disease Complication among Hypertension Patients Followup at DebreTabor Hospital, Ethiopia. Vascular Health and Risk Management. 2023;19:621-35.
13. Zakane SA. The Use of Computerised Clinical Decision Support System in Maternal and Neonatal Care in Rural Africa : Enthusiasm and

Concerns: ProQuest LLC; 2018.
14. Yeo EJ, Kralles H, Sternberg D, McCullough D, Nadanasabesan A, Mayo R, et al. Implementing a low-threshold audio-only telehealth model for medication-assisted treatment of opioid use disorder at a community-based non-profit organization in Washington, D.C. Harm Reduct J. 2021;18(1):127.
15. Yazdanshenas H, Bazargan M, Jones L, Vawer M, Seto TB, Farooq S, et al. Engaging Gatekeeper-Stakeholders in Development of a Mobile Health Intervention to Improve Medication Adherence Among African American and Pacific Islander Elderly Patients With Hypertension. JMIR MHEALTH AND UHEALTH. 2016;4(4).
16. Yazdanshenas H, Bazargan M, Jones L, Vawer M, Seto TB, Farooq S, et al. Engaging Gatekeeper-Stakeholders in Development of a Mobile Health Intervention to Improve Medication Adherence Among African American and Pacific Islander Elderly Patients With Hypertension. JMIR Mhealth Uhealth. 2016;4(4):e116.
17. Yao Y, Su Y, editors. Health outcomes and cost benefit of home e-health in helping to manage heart failure. 15th IFIP WG 81 International Conference on Informatics and Semiotics in Organisations, ICISO 2014, May 23, 2014 - May 24, 2014; 2014; Shanghai, China: Springer Science and Business Media, LLC.
18. Wimble M. Understanding health and health-related behavior of users of internet health information. Telemedicine and e-Health. 2016;22(10):809-15.
19. Welch G, Balder A, Zagarins S. Telehealth program for type 2 diabetes: Usability, satisfaction, and clinical usefulness in an urban community health center. Telemedicine and e-Health. 2015;21(5):395-403.
20. Watkins JA, Goudge J, Gomez-Olive FX, Huxley C, Dodd K, Griffiths F. mHealth text and voice communication for monitoring people with chronic diseases in low-resource settings: a realist review. BMJ Global Health. 2018;3(2):e000543.
21. Watkins JA, Goudge J, Gomez-Olive FX, Huxley C, Dodd K, Griffiths F. mHealth text and voice communication for monitoring people with chronic diseases in low-resource settings: a realist review. BMJ Global Health. 2018;3(2):e000543.
22. Waris A, Vekaria-Hirani V, Saulo M. Outcomes of home based oxygen therapy in children discharged from Kenyatta National Hospital, a retrospective cohort study. African Journal of Respiratory Medicine. 2020;15(1).
23. Waris A, Vekaria-Hirani V, Saulo M. Outcomes of home based oxygen therapy in children discharged from Kenyatta National Hospital, a retrospective cohort study. African Journal of Respiratory Medicine. 2020;15(1).
24. Walcott-Bryant A, Ogallo W, Remy SL, Tryon K, Shena W, Bosker-Kibacha M. Addressing Care Continuity and Quality Challenges in the Management of Hypertension: Case Study of the Private Health Care Sector in Kenya. J Med Internet Res. 2021;23(2):e18899.
25. Walcott-Bryant A, Ogallo W, Remy SL, Tryon K, Shena W, Bosker-Kibacha M. Addressing care continuity and quality challenges in the

management of hypertension: Case study of the private health care sector in Kenya. Journal of Medical Internet Research. 2021;23(2):e18899.
26. Walcott-Bryant A, Ogallo W, Remy SL, Tryon K, Shena W, Bosker-Kibacha M. Addressing care continuity and quality challenges in the management of hypertension: Case study of the private health care sector in Kenya. Journal of Medical Internet Research. 2021;23(2):e18899.
27. Wabnitz AM, Chandler J, Treiber F, Sen S, Jenkins C, Newman JC, et al. Program to Avoid Cerebrovascular Events through Systematic Electronic Tracking and Tailoring of an Eminent Risk factor: Protocol of a RCT. J Stroke Cerebrovasc Dis. 2021;30(8):105815.
28. von Dadelszen P, Bhutta ZA, Sharma S, Bone J, Singer J, Wong H, et al. The Community-Level Interventions for Pre-eclampsia (CLIP) cluster randomised trials in Mozambique, Pakistan, and India: an individual participant-level meta-analysis. The Lancet. 2020;396(10250):553-63.
29. von Dadelszen P, Bhutta ZA, Sharma S, Bone J, Singer J, Wong H, et al. The Community-Level Interventions for Pre-eclampsia (CLIP) cluster randomised trials in Mozambique, Pakistan, and India: an individual participant-level meta-analysis. The Lancet. 2020;396(10250):553-63.
30. von Dadelszen P, Bhutta ZA, Sharma S, Bone J, Singer J, Wong H, et al. The Community-Level Interventions for Pre-eclampsia (CLIP) cluster randomised trials in Mozambique, Pakistan, and India: an individual participant-level meta-analysis. Lancet (London, England). 2020;396(10250):553-63.
31. von Dadelszen P, Bhutta ZA, Sharma S, Bone J, Singer J, Wong H, et al. The Community-Level Interventions for Pre-eclampsia (CLIP) cluster randomised trials in Mozambique, Pakistan, and India: an individual participant-level meta-analysis. Lancet (london, england). 2020;396(10250):553.
32. von Dadelszen P, Bhutta ZA, Sharma S, Bone J, Singer J, Wong H, et al. The Community-Level Interventions for Pre-eclampsia (CLIP) cluster randomised trials in Mozambique, Pakistan, and India: an individual participant-level meta-analysis. Lancet (london, england). 2020;396(10250):553.
33. von Dadelszen P, Bhutta ZA, Sharma S, Bone J, Singer J, Wong H, et al. The Community-Level Interventions for Pre-eclampsia (CLIP) cluster randomised trials in Mozambique, Pakistan, and India: an individual participant-level meta-analysis. Lancet. 2020;396(10250):553-63.
34. von Dadelszen P, Bhutta ZA, Sharma S, Bone J, Singer J, Wong H, et al. The Community-Level Interventions for Pre-eclampsia (CLIP) cluster randomised trials in Mozambique, Pakistan, and India: an individual participant-level meta-analysis. Lancet (London, England). 2020;396(10250):553-63.
35. von Dadelszen P, Bhutta ZA, Sharma S, Bone J, Singer J, Wong H, et al. The Community-Level Interventions for Pre-eclampsia (CLIP) cluster randomised trials in Mozambique, Pakistan, and India: an

individual participant-level meta-analysis. Lancet (London, England). 2020;396(10250):553-63.
36. Vizeshfar F, Zare M, Keshtkaran Z. Role-play versus lecture methods in community health volunteers. Nurse education today. 2019;79:175.
37. Vizeshfar F, Zare M, Keshtkaran Z. Role-play versus lecture methods in community health volunteers. Nurse education today. 2019;79:175.
38. Vilme H, Duke NN, Muiruri C, Wordlaw L, Skinner AC. Using Telehealth to Disseminate Primary, Secondary, and Tertiary CVD Interventions to Rural Populations. Curr Hypertens Rep. 2019;21(12):92.
39. Viljoen A, Klinker K, Wiesche M, Uebernickel F, Krcmar H. Design Principles for mHealth Application Development in Rural Parts of Developing Countries: The Case of Noncommunicable Diseases in Kenya. IEEE Transactions on Engineering Management. 2023;70(3):894-911.
40. Viljoen A, Klinker K, Wiesche M, Uebernickel F, Krcmar H. Design Principles for mHealth Application Development in Rural Parts of Developing Countries: The Case of Noncommunicable Diseases in Kenya. IEEE Transactions on Engineering Management. 2023:894-911.
41. Vedanthan R, Kamano JH, DeLong AK, Naanyu V, Binanay CA, Bloomfield GS, et al. Community Health Workers Improve Linkage to Hypertension Care in Western Kenya. J Am Coll Cardiol. 2019;74(15):1897-906.
42. Vedanthan R, Kamano JH, DeLong AK, Naanyu V, Binanay CA, Bloomfield GS, et al. Community Health Workers Improve Linkage to Hypertension Care in Western Kenya. Journal of the American College of Cardiology. 2019;74(15):1897-906.
43. Vedanthan R, Kamano JH, DeLong AK, Naanyu V, Binanay CA, Bloomfield GS, et al. Community Health Workers Improve Linkage to Hypertension Care in Western Kenya. Journal of the American College of Cardiology. 2019;74(15):1897-906.
44. Vedanthan R, Kamano JH, DeLong AK, Naanyu V, Binanay CA, Bloomfield GS, et al. Community Health Workers Improve Linkage to Hypertension Care in Western Kenya. Journal of the American College of Cardiology. 2019;74(15):1897.
45. Vedanthan R, Kamano JH, DeLong AK, Naanyu V, Binanay CA, Bloomfield GS, et al. Community Health Workers Improve Linkage to Hypertension Care in Western Kenya. Journal of the American College of Cardiology. 2019;74(15):1897-906.
46. Vedanthan R, Kamano JH, DeLong AK, Naanyu V, Binanay CA, Bloomfield GS, et al. Community Health Workers Improve Linkage to Hypertension Care in Western Kenya. Journal of the American College of Cardiology. 2019;74(15):1897.
47. Vedanthan R, Kamano JH, Bloomfield GS, Manji I, Pastakia S, Kimaiyo SN. Engaging the Entire Care Cascade in Western Kenya: A Model to Achieve the Cardiovascular Disease Secondary Prevention Roadmap Goals. Glob Heart. 2015;10(4):313-7.
48. Vedanthan R, Kamano JH, Bloomfield GS, Manji I, Pastakia S,

Kimaiyo SN. Engaging the Entire Care Cascade in Western Kenya: A Model to Achieve the Cardiovascular Disease Secondary Prevention Roadmap Goals. Global heart. 2015;10(4):313-7.
49. Vedanthan R, Kamano JH, Bloomfield GS, Manji I, Pastakia S, Kimaiyo SN. Engaging the Entire Care Cascade in Western Kenya: A Model to Achieve the Cardiovascular Disease Secondary Prevention Roadmap Goals. Global Heart. 2015;10(4):313-7.
50. Vedanthan R, Kamano JH, Bloomfield GS, Manji I, Pastakia S, Kimaiyo SN. Engaging the Entire Care Cascade in Western Kenya: A Model to Achieve the Cardiovascular Disease Secondary Prevention Roadmap Goals. Global heart. 2015;10(4):313-7.
51. Vedanthan R, Kamano JH, Bloomfield GS, Manji I, Pastakia S, Kimaiyo SN. Engaging the Entire Care Cascade in Western Kenya: A Model to Achieve the Cardiovascular Disease Secondary Prevention Roadmap Goals. Global heart. 2015;10(4):313-7.
52. Vedanthan R, Kamano JH, Bloomfield GS, Manji I, Pastakia S, Kimaiyo SN. Engaging the Entire Care Cascade in Western Kenya: A Model to Achieve the Cardiovascular Disease Secondary Prevention Roadmap Goals. Global Heart. 2015;10(4):313-7.
53. Vedanthan R, Blank E, Tuikong N, Kamano J, Misoi L, Tulienge D, et al. Usability and feasibility of a tablet-based Decision-Support and Integrated Record-keeping (DESIRE) tool in the nurse management of hypertension in rural western Kenya. Int J Med Inform. 2015;84(3):207-19.
54. Vedanthan R, Blank E, Tuikong N, Kamano J, Misoi L, Tulienge D, et al. Usability and feasibility of a tablet-based Decision-Support and Integrated Record-keeping (DESIRE) tool in the nurse management of hypertension in rural western Kenya. International journal of medical informatics. 2015;84(3):207-19.
55. Vedanthan R, Blank E, Tuikong N, Kamano J, Misoi L, Tulienge D, et al. Usability and feasibility of a tablet-based Decision-Support and Integrated Record-keeping (DESIRE) tool in the nurse management of hypertension in rural western Kenya. International Journal of Medical Informatics. 2015;84(3):207-19.
56. Vedanthan R, Blank E, Tuikong N, Kamano J, Misoi L, Tulienge D, et al. Usability and feasibility of a tablet-based decision-support and integrated Record-keeping (DESIRE) tool in the nurse management of hypertension in rural western Kenya. International Journal of Medical Informatics. 2015;84(3):207-19.
57. Vedanthan R, Blank E, Tuikong N, Kamano J, Misoi L, Tulienge D, et al. Usability and feasibility of a tablet-based Decision-Support and Integrated Record-keeping (DESIRE) tool in the nurse management of hypertension in rural western Kenya. International journal of medical informatics. 2015;84(3):207-19.
58. Vedanthan R, Blank E, Tuikong N, Kamano J, Misoi L, Tulienge D, et al. Usability and feasibility of a tablet-based Decision-Support and Integrated Record-keeping (DESIRE) tool in the nurse management of hypertension in rural western Kenya. International journal of medical informatics. 2015;84(3):207-19.
59. Vedanthan R, Blank E, Tuikong N, Kamano J, Misoi L, Tulienge

D, et al. Usability and feasibility of a tablet-based Decision-Support and Integrated Record-keeping (DESIRE) tool in the nurse management of hypertension in rural western Kenya. International Journal of Medical Informatics. 2015;84(3):207-19.
60. Vedanthan R, Blank E, Tuikong N, Kamano J, Misoi L, Tulienge D, et al. Usability and feasibility of a tablet-based decision-support and integrated Record-keeping (DESIRE) tool in the nurse management of hypertension in rural western Kenya. International Journal of Medical Informatics. 2015;84(3):207-19.
61. Vedanthan R, Blank E, Tuikong N, Kamano J, Misoi L, Tulienge D, et al. Usability and feasibility of a tablet-based Decision-Support and Integrated Record-keeping (DESIRE) tool in the nurse management of hypertension in rural western Kenya. International Journal of Medical Informatics. 2015;84(3):207-19.
62. Vedanthan R, Blank E, Tuikong N, Kamano J, Misoi L, Tulienge D, et al. Usability and feasibility of a tablet-based decision-support and integrated Record-keeping (DESIRE) tool in the nurse management of hypertension in rural western Kenya. International Journal of Medical Informatics. 2015;84(3):207-19.
63. Tweheyo R, Selig H, Gibson DG, Pariyo GW, Rutebemberwa E. User Perceptions and Experiences of an Interactive Voice Response Mobile Phone Survey Pilot in Uganda: Qualitative Study. JMIR Form Res. 2020;4(12):e21671.
64. Tweheyo R, Selig H, Gibson DG, Pariyo GW, Rutebemberwa E. User Perceptions and Experiences of an Interactive Voice Response Mobile Phone Survey Pilot in Uganda: Qualitative Study. JMIR formative research. 2020;4(12):e21671.
65. Tweheyo R, Selig H, Gibson DG, Pariyo GW, Rutebemberwa E. User Perceptions and Experiences of an Interactive Voice Response Mobile Phone Survey Pilot in Uganda: Qualitative Study. JMIR formative research. 2020;4(12):e21671.
66. Tomás CC, Oliveira E, Sousa D, Uba-Chupel M, Furtado G, Rocha C, et al. Proceedings of the 3rd IPLeiria's International Health Congress : Leiria, Portugal. 6-7 May 2016. BMC Health Serv Res. 2016;16 Suppl 3(Suppl 3):200.
67. Thiga M, Kimeto P, Mgala M, Kweyu E, Wanyee S, Mwirigi T, editors. A Remote Blood Pressure Data Collection and Monitoring System for Expectant Mothers. 2022 IST-Africa Conference, IST-Africa 2022, May 16, 2022 - May 20, 2022; 2022; Virtual, Online, Ireland: Institute of Electrical and Electronics Engineers Inc.
68. Thiga M, Kimeto P, Mgala M, Kweyu E, Wanyee S, Mwirigi T, editors. A Remote Blood Pressure Data Collection and Monitoring System for Expectant Mothers. 2022 IST-Africa Conference (IST-Africa), 16-20 May 2022; 2022; Piscataway, NJ, USA: IEEE.
69. Takpor TO, Atayero AA, editors. Integrating internet of things and EHealth solutions for students' healthcare. 2015 World Congress on Engineering, WCE 2015, July 1, 2015 - July 3, 2015; 2015; London, United kingdom: Newswood Limited.
70. Tabi AE. Improving Antihypertensive Medication Adherence among African American Adult Patients Using the Medisafe Mobile App:

ProQuest LLC; 2021.
71. Sturt J, Rogers R, Griffiths F, Ajisola M, Akinyemi JO, Chipwaza B, et al. Safety and upscaling of remote consulting for long-term conditions in primary health care in Nigeria and Tanzania (REaCH trials): stepped-wedge trials of training, mobile data allowance, and implementation. Lancet Global Health. 2023;11(11):e1753-e64.
72. Sturt J, Rogers R, Griffiths F, Ajisola M, Akinyemi JO, Chipwaza B, et al. Safety and upscaling of remote consulting for long-term conditions in primary health care in Nigeria and Tanzania (REaCH trials): stepped-wedge trials of training, mobile data allowance, and implementation. Lancet Global Health. 2023;11(11):e1753-e64.
73. Sturt J, Griffiths F, Ajisola M, Akinyemi JO, Chipwaza B, Fayehun O, et al. Safety and upscaling of remote consulting for long-term conditions in primary health care in Nigeria and Tanzania (REaCH trials): stepped-wedge trials of training, mobile data allowance, and implementation. Lancet Glob Health. 2023;11(11):e1753-e64.
74. Sturt J, Griffiths F, Ajisola M, Akinyemi JO, Chipwaza B, Fayehun O, et al. Safety and upscaling of remote consulting for long-term conditions in primary health care in Nigeria and Tanzania (REaCH trials): stepped-wedge trials of training, mobile data allowance, and implementation. The Lancet Global health. 2023;11(11):e1753-e64.
75. Sturt J, Griffiths F, Ajisola M, Akinyemi JO, Chipwaza B, Fayehun O, et al. Safety and upscaling of remote consulting for long-term conditions in primary health care in Nigeria and Tanzania (REaCH trials): stepped-wedge trials of training, mobile data allowance, and implementation. The Lancet Global health. 2023;11(11):e1753-e64.
76. Sturt J, Griffiths F, Ajisola M, Akinyemi JO, Chipwaza B, Fayehun O, et al. Safety and upscaling of remote consulting for long-term conditions in primary health care in Nigeria and Tanzania (REaCH trials): stepped-wedge trials of training, mobile data allowance, and implementation. The Lancet Global health. 2023;11(11):e1753-e64.
77. Sturt J, Griffiths F, Ajisola M, Akinyemi JO, Chipwaza B, Fayehun O, et al. Safety and upscaling of remote consulting for long-term conditions in primary health care in Nigeria and Tanzania (REaCH trials): stepped-wedge trials of training, mobile data allowance, and implementation. The Lancet Global health. 2023;11(11):e1753-e64.
78. Sturt J, Griffiths F, Ajisola M, Akinyemi J, Chipwaza B, Rogers R, et al. IDF2022-0837 Are remote consultations in low-middle income countries feasible, safe and trustworthy? A stepped wedge RCT of REaCH. Diabetes Research and Clinical Practice. 2023;197(Supplement 1):110484.
79. Sturt J, Griffiths F, Ajisola M, Akinyemi J, Chipwaza B, Rogers R, et al. IDF2022-0837 Are remote consultations in low-middle income countries feasible, safe and trustworthy? A stepped wedge RCT of REaCH. Diabetes Research and Clinical Practice. 2023;197(Supplement 1):110484.
80. Stokes K, Oronti B, Cappuccio FP, Pecchia L. Use of technology to prevent, detect, manage and control hypertension in sub-Saharan Africa: a systematic review. BMJ Open. 2022;12(4):e058840.
81. Still CH, Jones LM, Moss KO, Variath M, Wright KD. African

American Older Adults' Perceived Use of Technology for Hypertension Self-Management. Res Gerontol Nurs. 2018;11(5):249-56.
82. Stephen BU-A, Uzoewulu BC, Asuquo PM, Ozuomba S. Diabetes and hypertension MobileHealth systems: a review of general challenges and advancements. Journal of Engineering and Applied Science. 2023;70(1).
83. Starr N, Gebeyehu N, Tesfaye A, Forrester JA, Bekele A, Bitew S, et al. Value and Feasibility of Telephone Follow-Up in Ethiopian Surgical Patients. Surgical Infections. 2020;21(6):533-9.
84. Starr N, Gebeyehu N, Tesfaye A, Forrester JA, Bekele A, Bitew S, et al. Value and Feasibility of Telephone Follow-Up in Ethiopian Surgical Patients. Surgical Infections. 2020;21(6):533-9.
85. Stanifer JW, Turner EL, Egger JR, Thielman N, Karia F, Maro V, et al. Knowledge, Attitudes, and Practices Associated with Chronic Kidney Disease in Northern Tanzania: A Community-Based Study. PLoS One. 2016;11(6):e0156336.
86. Stanifer JW, Turner EL, Egger JR, Thielman N, Karia F, Maro V, et al. Knowledge, Attitudes, and Practices Associated with Chronic Kidney Disease in Northern Tanzania: A Community-Based Study. PloS one. 2016;11(6):e0156336.
87. Stanifer JW, Turner EL, Egger JR, Thielman N, Karia F, Maro V, et al. Knowledge, attitudes, and practices associated with chronic kidney disease in northern Tanzania: A community-based study. PLoS ONE. 2016;11(6):e0156336.
88. Stanifer JW, Turner EL, Egger JR, Thielman N, Karia F, Maro V, et al. Knowledge, Attitudes, and Practices Associated with Chronic Kidney Disease in Northern Tanzania: A Community-Based Study. PloS one. 2016;11(6):e0156336.
89. Stanifer JW, Turner EL, Egger JR, Thielman N, Karia F, Maro V, et al. Knowledge, Attitudes, and Practices Associated with Chronic Kidney Disease in Northern Tanzania: A Community-Based Study. PloS one. 2016;11(6):e0156336.
90. Stanifer JW, Turner EL, Egger JR, Thielman N, Karia F, Maro V, et al. Knowledge, attitudes, and practices associated with chronic kidney disease in northern Tanzania: A community-based study. PLoS ONE. 2016;11(6):e0156336.
91. Staffileno BA, Tangney CC, Fogg L, Darmoc R. Making Behavior Change Interventions Available to Young African American Women: Development and Feasibility of an eHealth Lifestyle Program. J Cardiovasc Nurs. 2015;30(6):497-505.
92. Staffileno BA, Tangney CC, Fogg L. Favorable Outcomes Using an eHealth Approach to Promote Physical Activity and Nutrition Among Young African American Women. J Cardiovasc Nurs. 2018;33(1):62-71.
93. Spruill TM, Williams O, Teresi JA, Lehrer S, Pezzin L, Waddy SP, et al. Comparative effectiveness of home blood pressure telemonitoring (HBPTM) plus nurse case management versus HBPTM alone among Black and Hispanic stroke survivors: study protocol for a randomized controlled trial. Trials. 2015;16:97.
94. Springer DB, Bobrow KL, Levitt N, Farmer A, Tarassenko L, editors. The SMS-text Adherence support (StAR) study - Hardware and software infrastructure. 6th International Conference on Information

and Communications Technologies and Development, ICTD 2013, December 7, 2013 - December 10, 2013; 2013; Cape Town, South africa: Association for Computing Machinery.
95. Song Y, Li Y, Guo C, Wang Y, Huang L, Tan M, et al. Cross-sectional comparisons of sodium content in processed meat and fish products among five countries: potential for feasible targets and reformulation. BMJ Open. 2021;11(10):e046412.
96. Song Y, Li Y, Guo C, Wang Y, Huang L, Tan M, et al. Cross-sectional comparisons of sodium content in processed meat and fish products among five countries: potential for feasible targets and reformulation. BMJ open. 2021;11(10):e046412.
97. Song Y, Li Y, Guo C, Wang Y, Huang L, Tan M, et al. Cross-sectional comparisons of sodium content in processed meat and fish products among five countries: potential for feasible targets and reformulation. BMJ Open. 2021;11(10).
98. Song Y, Li Y, Guo C, Wang Y, Huang L, Tan M, et al. Cross-sectional comparisons of sodium content in processed meat and fish products among five countries: Potential for feasible targets and reformulation. BMJ Open. 2021;11(10):e046412.
99. Song Y, Li Y, Guo C, Wang Y, Huang L, Tan M, et al. Cross-sectional comparisons of sodium content in processed meat and fish products among five countries: potential for feasible targets and reformulation. BMJ open. 2021;11(10):e046412.
100. Song Y, Li Y, Guo C, Wang Y, Huang L, Tan M, et al. Cross-sectional comparisons of sodium content in processed meat and fish products among five countries: potential for feasible targets and reformulation. BMJ Open. 2021;11(10).
101. Song Y, Li Y, Guo C, Wang Y, Huang L, Tan M, et al. Cross-sectional comparisons of sodium content in processed meat and fish products among five countries: Potential for feasible targets and reformulation. BMJ Open. 2021;11(10):e046412.
102. Soltanimehr E, Bahrampour E, Yousefvand Z. Efficacy of diode and CO2 lasers along with calcium and fluoride-containing compounds for the remineralization of primary teeth. BMC oral health. 2019;19(1):N.
103. Soltanimehr E, Bahrampour E, Yousefvand Z. Efficacy of diode and CO2 lasers along with calcium and fluoride-containing compounds for the remineralization of primary teeth. BMC oral health. 2019;19(1):N.
104. Skolarus LE, Dinh M, Kidwell KM, Lin CC, Buis LR, Brown DL, et al. Reach Out Emergency Department: A Randomized Factorial Trial to Determine the Optimal Mobile Health Components to Reduce Blood Pressure. Circ Cardiovasc Qual Outcomes. 2023;16(5):e009606.
105. Skolarus LE, Cowdery J, Dome M, Bailey S, Baek J, Byrd JB, et al. Reach Out Churches: A Community-Based Participatory Research Pilot Trial to Assess the Feasibility of a Mobile Health Technology Intervention to Reduce Blood Pressure Among African Americans. Health Promot Pract. 2018;19(4):495-505.
106. Skau JK, Nordin AB, Cheah JC, Ali R, Zainal R, Aris T, et al. A complex behavioural change intervention to reduce the risk of

diabetes and prediabetes in the pre-conception period in Malaysia: study protocol for a randomised controlled trial. Trials. 2016;17(1):215.
107. Sithole N, Castle A, Nxumalo S, Mazibuko L, Manyaapelo T, Abrahams-Gessel S, et al. Protocol: Implementation evaluation of a combination intervention for sustainable blood pressure control in rural KwaZulu-Natal, South Africa (IMPACT BP): A three-arm, unblinded, parallel group individually randomized clinical trial. Contemp Clin Trials. 2023;131:107258.
108. Sithole N, Castle A, Nxumalo S, Mazibuko L, Manyaapelo T, Abrahams-Gessel S, et al. Protocol: Implementation evaluation of a combination intervention for sustainable blood pressure control in rural KwaZulu-Natal, South Africa (IMPACT BP): A three-arm, unblinded, parallel group individually randomized clinical trial. Contemporary clinical trials. 2023;131:107258.
109. Sithole N, Castle A, Nxumalo S, Mazibuko L, Manyaapelo T, Abrahams-Gessel S, et al. Protocol: Implementation evaluation of a combination intervention for sustainable blood pressure control in rural KwaZulu-Natal, South Africa (IMPACT BP): A three-arm, unblinded, parallel group individually randomized clinical trial. Contemporary Clinical Trials. 2023;131:107258.
110. Sithole N, Castle A, Nxumalo S, Mazibuko L, Manyaapelo T, Abrahams-Gessel S, et al. Protocol: Implementation evaluation of a combination intervention for sustainable blood pressure control in rural KwaZulu-Natal, South Africa (IMPACT BP): A three-arm, unblinded, parallel group individually randomized clinical trial. Contemporary clinical trials. 2023;131:107258.
111. Sithole N, Castle A, Nxumalo S, Mazibuko L, Manyaapelo T, Abrahams-Gessel S, et al. Protocol: implementation evaluation of a combination intervention for sustainable blood pressure control in rural KwaZulu-Natal, South Africa (IMPACT BP): a three-arm, unblinded, parallel group individually randomized clinical trial. Contemporary clinical trials. 2023:107258.
112. Sithole N, Castle A, Nxumalo S, Mazibuko L, Manyaapelo T, Abrahams-Gessel S, et al. Protocol: Implementation evaluation of a combination intervention for sustainable blood pressure control in rural KwaZulu-Natal, South Africa (IMPACT BP): A three-arm, unblinded, parallel group individually randomized clinical trial. Contemporary clinical trials. 2023;131:107258.
113. Sithole N, Castle A, Nxumalo S, Mazibuko L, Manyaapelo T, Abrahams-Gessel S, et al. Protocol: Implementation evaluation of a combination intervention for sustainable blood pressure control in rural KwaZulu-Natal, South Africa (IMPACT BP): A three-arm, unblinded, parallel group individually randomized clinical trial. Contemporary Clinical Trials. 2023;131:107258.
114. Sithole N, Castle A, Nxumalo S, Mazibuko L, Manyaapelo T, Abrahams-Gessel S, et al. Protocol: implementation evaluation of a combination intervention for sustainable blood pressure control in rural KwaZulu-Natal, South Africa (IMPACT BP): a three-arm, unblinded, parallel group individually randomized clinical trial. Contemporary

clinical trials. 2023:107258.
115. Sieverdes JC, Adams ZW, Nemeth L, Brunner-Jackson B, Mueller M, Anderson A, et al. Formative evaluation on cultural tailoring breathing awareness meditation smartphone apps to reduce stress and blood pressure. Mhealth. 2017;3:44.
116. Shahid K, Kolomeyer AM, Nayak NV, Salameh N, Pelaez G, Khouri AS, et al. Ocular telehealth screenings in an urban community. Telemed J E Health. 2012;18(2):95-100.
117. Shahid K, Kolomeyer AM, Nayak NV, Salameh N, Pelaez G, Khouri AS, et al. Ocular Telehealth Screenings in an Urban Community. Telemedicine Journal and e-Health. 2012;18(2):95-100.
118. Shahid K, Kolomeyer AM, Nayak NV, Salameh N, Pelaez G, Khouri AS, et al. Ocular telehealth screenings in an urban community. Telemedicine and e-Health. 2012;18(2):95-100.
119. Sevene E, Sharma S, Munguambe K, Sacoor C, Vala A, Macuacua S, et al. Community-level interventions for pre-eclampsia (CLIP) in Mozambique: A cluster randomised controlled trial. Pregnancy Hypertens. 2020;21:96-105.
120. Sevene E, Sharma S, Munguambe K, Sacoor C, Vala A, Macuacua S, et al. Community-level interventions for pre-eclampsia (CLIP) in Mozambique: A cluster randomised controlled trial. Pregnancy hypertension. 2020;21:96-105.
121. Sevene E, Sharma S, Munguambe K, Sacoor C, Vala A, Macuacua S, et al. Community-level interventions for pre-eclampsia (CLIP) in Mozambique: A cluster randomised controlled trial. Pregnancy Hypertension. 2020;21:96-105.
122. Sevene E, Sharma S, Munguambe K, Sacoor C, Vala A, Macuacua S, et al. Community-level interventions for pre-eclampsia (CLIP) in Mozambique: A cluster randomised controlled trial. Pregnancy hypertension. 2020;21:96-105.
123. Sevene E, Sharma S, Munguambe K, Sacoor C, Vala A, Macuacua S, et al. Community-level interventions for pre-eclampsia (CLIP) in Mozambique: a cluster randomised controlled trial. Pregnancy hypertension. 2020;21:96.
124. Sevene E, Sharma S, Munguambe K, Sacoor C, Vala A, Macuacua S, et al. Community-level interventions for pre-eclampsia (CLIP) in Mozambique: A cluster randomised controlled trial. Pregnancy hypertension. 2020;21:96-105.
125. Sevene E, Sharma S, Munguambe K, Sacoor C, Vala A, Macuacua S, et al. Community-level interventions for pre-eclampsia (CLIP) in Mozambique: A cluster randomised controlled trial. Pregnancy Hypertension. 2020;21:96-105.
126. Sevene E, Sharma S, Munguambe K, Sacoor C, Vala A, Macuacua S, et al. Community-level interventions for pre-eclampsia (CLIP) in Mozambique: a cluster randomised controlled trial. Pregnancy hypertension. 2020;21:96.
127. Schutte AE, Srinivasapura Venkateshmurthy N, Mohan S, Prabhakaran D. Hypertension in Low- and Middle-Income Countries. Circ Res. 2021;128(7):808-26.
128. Schoenthaler A, Leon M, Butler M, Steinhaeuser K, Wardzinski

W. Development and Evaluation of a Tailored Mobile Health Intervention to Improve Medication Adherence in Black Patients With Uncontrolled Hypertension and Type 2 Diabetes: Pilot Randomized Feasibility Trial. JMIR Mhealth Uhealth. 2020;8(9):e17135.
129. Schmidt C, Öner A, Mann M, Krockenberger K, Abbondanzieri M, Brandewiede B, et al. A novel integrated care concept (NICC) versus standard care in the treatment of chronic cardiovascular diseases: protocol for the randomized controlled trial CardioCare MV. Trials. 2018;19(1):120.
130. Sarma P, Cassidy R, Corlett S, Katusiime B. Ageing with HIV: Medicine Optimisation Challenges and Support Needs for Older People Living with HIV: A Systematic Review. Drugs and Aging. 2023;40(3):179-240.
131. Sarma P, Cassidy R, Corlett S, Katusiime B. Ageing with HIV: Medicine Optimisation Challenges and Support Needs for Older People Living with HIV: A Systematic Review. Drugs and Aging. 2023;40(3):179-240.
132. Sarfo FS, Treiber F, Jenkins C, Patel S, Gebregziabher M, Singh A, et al. Phone-based Intervention under Nurse Guidance after Stroke (PINGS): study protocol for a randomized controlled trial. Trials. 2016;17(1):436.
133. Sarfo FS, Treiber F, Jenkins C, Patel S, Gebregziabher M, Singh A, et al. Phone-based Intervention under Nurse Guidance after Stroke (PINGS): study protocol for a randomized controlled trial. Trials. 2016;17(1):436.
134. Sarfo FS, Treiber F, Jenkins C, Patel S, Gebregziabher M, Singh A, et al. Phone-based Intervention under Nurse Guidance after Stroke (PINGS): study protocol for a randomized controlled trial. Trials. 2016;17(1):436.
135. Sarfo FS, Treiber F, Jenkins C, Patel S, Gebregziabher M, Singh A, et al. Phone-based Intervention under Nurse Guidance after Stroke (PINGS): study protocol for a randomized controlled trial. Trials. 2016;17(1):436.
136. Sarfo FS, Treiber F, Jenkins C, Patel S, Gebregziabher M, Singh A, et al. Phone-based Intervention under Nurse Guidance after Stroke (PINGS): study protocol for a randomized controlled trial. Trials. 2016;17(1):436.
137. Sarfo FS, Treiber F, Jenkins C, Patel S, Gebregziabher M, Singh A, et al. Phone-based Intervention under Nurse Guidance after Stroke (PINGS): study protocol for a randomized controlled trial. Trials. 2016;17(1):436.
138. Sarfo FS, Treiber F, Gebregziabher M, Adamu S, Nichols M, Singh A, et al. Phone-based intervention for blood pressure control among Ghanaian stroke survivors: A pilot randomized controlled trial. International journal of stroke : official journal of the International Stroke Society. 2019;14(6):630-8.
139. Sarfo FS, Treiber F, Gebregziabher M, Adamu S, Nichols M, Singh A, et al. Phone-based intervention for blood pressure control among Ghanaian stroke survivors: a pilot randomized controlled trial. International journal of stroke. 2019;14(6):630.

140. Sarfo FS, Treiber F, Gebregziabher M, Adamu S, Nichols M, Singh A, et al. Phone-based intervention for blood pressure control among Ghanaian stroke survivors: a pilot randomized controlled trial. International journal of stroke. 2019;14(6):630.
141. Sarfo FS, Treiber F, Gebregziabher M, Adamu S, Nichols M, Singh A, et al. Phone-based intervention for blood pressure control among Ghanaian stroke survivors: A pilot randomized controlled trial. Int J Stroke. 2019;14(6):630-8.
142. Sarfo FS, Treiber F, Gebregziabher M, Adamu S, Nichols M, Singh A, et al. Phone-based intervention for blood pressure control among Ghanaian stroke survivors: A pilot randomized controlled trial. International journal of stroke : official journal of the International Stroke Society. 2019;14(6):630-8.
143. Sarfo FS, Treiber F, Gebregziabher M, Adamu S, Nichols M, Singh A, et al. Phone-based intervention for blood pressure control among Ghanaian stroke survivors: A pilot randomized controlled trial. International Journal of Stroke. 2019;14(6):630-8.
144. Sarfo FS, Treiber F, Gebregziabher M, Adamu S, Nichols M, Singh A, et al. Phone-based intervention for blood pressure control among Ghanaian stroke survivors: a pilot randomized controlled trial. International journal of stroke. 2019;14(6):630.
145. Sarfo FS, Treiber F, Gebregziabher M, Adamu S, Nichols M, Singh A, et al. Phone-based intervention for blood pressure control among Ghanaian stroke survivors: A pilot randomized controlled trial. International journal of stroke : official journal of the International Stroke Society. 2019;14(6):630-8.
146. Sarfo FS, Treiber F, Gebregziabher M, Adamu S, Nichols M, Singh A, et al. Phone-based intervention for blood pressure control among Ghanaian stroke survivors: A pilot randomized controlled trial. International Journal of Stroke. 2019;14(6):630-8.
147. Sarfo FS, Treiber F, Gebregziabher M, Adamu S, Nichols M, Singh A, et al. Phone-based intervention for blood pressure control among Ghanaian stroke survivors: a pilot randomized controlled trial. International journal of stroke. 2019;14(6):630.
148. Sarfo FS, Ovbiagele B. Key determinants of long-term post-stroke mortality in Ghana. J Neurol Sci. 2022;434:120123.
149. Sarfo FS, Akpalu A, Bockarie A, Appiah L, Nguah SB, Ayisi-Boateng NK, et al. Phone-Based Intervention under Nurse Guidance after Stroke (PINGS II) Study: Protocol for a Phase III Randomized Clinical Trial. J Stroke Cerebrovasc Dis. 2021;30(8):105888.
150. Sarfo FS, Akpalu A, Bockarie A, Appiah L, Nguah SB, Ayisi-Boateng NK, et al. Phone-Based Intervention under Nurse Guidance after Stroke (PINGS II) Study: Protocol for a Phase III Randomized Clinical Trial. Journal of stroke and cerebrovascular diseases : the official journal of National Stroke Association. 2021;30(8):105888.
151. Sarfo FS, Akpalu A, Bockarie A, Appiah L, Nguah SB, Ayisi-Boateng NK, et al. Phone-Based Intervention under Nurse Guidance after Stroke (PINGS II) Study: Protocol for a Phase III Randomized Clinical Trial. Journal of Stroke and Cerebrovascular Diseases. 2021;30(8):105888.

152. Sarfo FS, Akpalu A, Bockarie A, Appiah L, Nguah SB, Ayisi-Boateng NK, et al. Phone-Based Intervention under Nurse Guidance after Stroke (PINGS II) Study: Protocol for a Phase III Randomized Clinical Trial. Journal of stroke and cerebrovascular diseases : the official journal of National Stroke Association. 2021;30(8):105888.
153. Sarfo FS, Akpalu A, Bockarie A, Appiah L, Nguah SB, Ayisi-Boateng NK, et al. Phone-Based Intervention under Nurse Guidance after Stroke (PINGS II) Study: Protocol for a Phase III Randomized Clinical Trial. Journal of stroke and cerebrovascular diseases : the official journal of National Stroke Association. 2021;30(8):105888.
154. Sarfo FS, Akpalu A, Bockarie A, Appiah L, Nguah SB, Ayisi-Boateng NK, et al. Phone-Based Intervention under Nurse Guidance after Stroke (PINGS II) Study: protocol for a Phase III Randomized Clinical Trial. Journal of stroke and cerebrovascular diseases. 2021;30(8):105888.
155. Sarfo FS, Akpalu A, Bockarie A, Appiah L, Nguah SB, Ayisi-Boateng NK, et al. Phone-Based Intervention under Nurse Guidance after Stroke (PINGS II) Study: Protocol for a Phase III Randomized Clinical Trial. Journal of stroke and cerebrovascular diseases : the official journal of National Stroke Association. 2021;30(8):105888.
156. Sarfo FS, Akpalu A, Bockarie A, Appiah L, Nguah SB, Ayisi-Boateng NK, et al. Phone-Based Intervention under Nurse Guidance after Stroke (PINGS II) Study: Protocol for a Phase III Randomized Clinical Trial. Journal of Stroke and Cerebrovascular Diseases. 2021;30(8):105888.
157. Sarfo FS, Akpalu A, Bockarie A, Appiah L, Nguah SB, Ayisi-Boateng NK, et al. Phone-Based Intervention under Nurse Guidance after Stroke (PINGS II) Study: protocol for a Phase III Randomized Clinical Trial. Journal of stroke and cerebrovascular diseases. 2021;30(8):105888.
158. Sarfo F, Treiber F, Gebregziabher M, Adamu S, Patel S, Nichols M, et al. PINGS (Phone-Based Intervention Under Nurse Guidance After Stroke): Interim Results of a Pilot Randomized Controlled Trial. Stroke. 2018;49(1):236-9.
159. Santamore WP, Homko CJ, Kashem A, McConnell TR, Menapace FJ, Bove AA. Accuracy of blood pressure measurements transmitted through a telemedicine system in underserved populations. Telemed J E Health. 2008;14(4):333-8.
160. Santamore WP, Homko CJ, Kashem A, McConnell TR, Menapace FJ, Bove AA. Accuracy of blood pressure measurements transmitted through a telemedicine system in underserved populations. Telemedicine and e-Health. 2008;14(4):333-8.
161. Sahu M, Grover A, Joshi A. Role of mobile phone technology in health education in Asian and African countries: a systematic review. International Journal of Electronic Healthcare. 2014;7(4):269-86.
162. Ryu D, Dong Hyun K, Price JT, Jong Yoon L, Ha Uk C, Allen E, et al. Comprehensive pregnancy monitoring with a network of wireless, soft, and flexible sensors in high- and low-resource health settings. Proceedings of the National Academy of Sciences of the United States of America. 2021;118(20):1-11.

163. Rui W, Fangzhou X, editors. Low-cost Portable Ventilator Design for Underdeveloped Regions. 2021 IEEE Integrated STEM Education Conference (ISEC), 13 March 2021; 2021; Piscataway, NJ, USA: IEEE.
164. Rubin R. Taking Medicine to the Streets to Care for Those Who Live There. JAMA - Journal of the American Medical Association. 2020;323(8):695-8.
165. Rubin R. Taking Medicine to the Streets to Care for Those Who Live There. JAMA - Journal of the American Medical Association. 2020;323(8):695-8.
166. Rogers MA, Small D, Buchan DA, Butch CA, Stewart CM, Krenzer BE, et al. Home monitoring service improves mean arterial pressure in patients with essential hypertension. A randomized, controlled trial. Ann Intern Med. 2001;134(11):1024-32.
167. Robles MC, Newman MW, Doshi A, Bailey S, Huang L, Choi SJ, et al. A Physical Activity Just-in-time Adaptive Intervention Designed in Partnership With a Predominantly Black Community: Virtual, Community-Based Participatory Design Approach. JMIR Form Res. 2022;6(3):e33087.
168. Rivera RL, Adams M, Dawkins E, Carter A, Zhang X, Tu W, et al. Delivering Food Resources and Kitchen Skills (FoRKS) to Adults with Food Insecurity and Hypertension: A Pilot Study. Nutrients. 2023;15(6).
169. Rehman H, Kamal AK, Morris PB, Sayani S, Merchant AT, Virani SS. Mobile Health (mHealth) Technology for the Management of Hypertension and Hyperlipidemia: Slow Start but Loads of Potential. Curr Atheroscler Rep. 2017;19(3):12.
170. Rahmah ASN, Tamtomo D, Murti B. Meta analysis the effect of electronic health-based self management in controlling blood pressure in hipertensive patients. Journal of Health Policy and Management. 2023;8(2):105-18.
171. Rahmah ASN, Tamtomo D, Murti B. Meta analysis the effect of electronic health-based self management in controlling blood pressure in hipertensive patients. Journal of Health Policy and Management. 2023;8(2):105-18.
172. Pozo-Martin F, Akazili J, Der R, Laar A, Adler AJ, Lamptey P, et al. Cost-effectiveness of a Community-based Hypertension Improvement Project (ComHIP) in Ghana: results from a modelling study. BMJ Open. 2021;11(9):e039594.
173. Pozo-Martin F, Akazili J, Der R, Laar A, Adler AJ, Lamptey P, et al. Cost-effectiveness of a Community-based Hypertension Improvement Project (ComHIP) in Ghana: results from a modelling study. BMJ open. 2021;11(9):e039594.
174. Pozo-Martin F, Akazili J, Der R, Laar A, Adler AJ, Lamptey P, et al. Cost-effectiveness of a community-based hypertension improvement project (ComHIP) in Ghana: results from a modelling study. BMJ Open. 2021;11(9).
175. Pozo-Martin F, Akazili J, Der R, Laar A, Adler AJ, Lamptey P, et al. Cost-effectiveness of a Community-based Hypertension Improvement Project (ComHIP) in Ghana: Results from a modelling study. BMJ Open. 2021;11(9):e039594.
176. Pozo-Martin F, Akazili J, Der R, Laar A, Adler AJ, Lamptey P,

et al. Cost-effectiveness of a Community-based Hypertension Improvement Project (ComHIP) in Ghana: results from a modelling study. BMJ open. 2021;11(9):e039594.
177. Pozo-Martin F, Akazili J, Der R, Laar A, Adler AJ, Lamptey P, et al. Cost-effectiveness of a Community-based Hypertension Improvement Project (ComHIP) in Ghana: results from a modelling study. BMJ open. 2021;11(9):e039594.
178. Pozo-Martin F, Akazili J, Der R, Laar A, Adler AJ, Lamptey P, et al. Cost-effectiveness of a Community-based Hypertension Improvement Project (ComHIP) in Ghana: results from a modelling study. BMJ open. 2021;11(9):e039594.
179. Pozo-Martin F, Akazili J, Der R, Laar A, Adler AJ, Lamptey P, et al. Cost-effectiveness of a Community-based Hypertension Improvement Project (ComHIP) in Ghana: results from a modelling study. BMJ open. 2021;11(9):e039594.
180. Pozo-Martin F, Akazili J, Der R, Laar A, Adler AJ, Lamptey P, et al. Cost-effectiveness of a community-based hypertension improvement project (ComHIP) in Ghana: results from a modelling study. BMJ Open. 2021;11(9).
181. Pozo-Martin F, Akazili J, Der R, Laar A, Adler AJ, Lamptey P, et al. Cost-effectiveness of a Community-based Hypertension Improvement Project (ComHIP) in Ghana: Results from a modelling study. BMJ Open. 2021;11(9):e039594.
182. Pozo-Martin F, Akazili J, Der R, Laar A, Adler AJ, Lamptey P, et al. Cost-effectiveness of a Community-based Hypertension Improvement Project (ComHIP) in Ghana: results from a modelling study. BMJ open. 2021;11(9):e039594.
183. Postigo M, Beltran-Agullo L, Pujol O, Bila R, Garcia J, Varo R, et al. Validation of a low-cost mobile retinal scanner prototype as a screening tool for retinal diseases in Mozambique. American Journal of Tropical Medicine and Hygiene. 2020;103(5 SUPPL):242.
184. Postigo M, Beltran-Agullo L, Pujol O, Bila R, Garcia J, Varo R, et al. Validation of a low-cost mobile retinal scanner prototype as a screening tool for retinal diseases in Mozambique. American Journal of Tropical Medicine and Hygiene. 2020;103(5 SUPPL):242.
185. Pokuaa S. Will the Use of a Mobile Adherence Application Improve Systolic Blood Pressure by 5 mmHg Among Adult African Americans in Two Months? : ProQuest LLC; 2022.
186. Plessis ED, Swart F, Maree D, Van Heerden J, Esterhuizen TM, Irusen EM, et al. The utility of hand-held mobile spirometer technology in a resource-constrained setting. South African Medical Journal. 2019;109(4):219-22.
187. Plessis ED, Swart F, Maree D, Van Heerden J, Esterhuizen TM, Irusen EM, et al. The utility of hand-held mobile spirometer technology in a resource-constrained setting. South African Medical Journal. 2019;109(4):219-22.
188. Piot P, Aerts A, Wood DA, Lamptey P, Oti S, Connell K, et al. Innovating healthcare delivery to address noncommunicable diseases in low-income settings: the example of hypertension. Future Cardiol. 2016;12(4):401-3.

189. Pedrosa VM, Abrantes AV. The role of mHealth in chronic disease management in Sub-Saharan Africa. European Journal of Public Health, suppl 5. 2020;30.
190. Patel S, Jacobus-Kantor L, Marshall L, Ritchie C, Kaplinski M, Khurana PS, et al. Mobilizing your medications: an automated medication reminder application for mobile phones and hypertension medication adherence in a high-risk urban population. J Diabetes Sci Technol. 2013;7(3):630-9.
191. Palmer MJ, Barnard S, Perel P, Free C. Mobile phone-based interventions for improving adherence to medication prescribed for the primary prevention of cardiovascular disease in adults. Cochrane Database of Systematic Reviews. 2018;2018(6):CD012675.
192. Palmer MJ, Barnard S, Perel P, Free C. Mobile phone-based interventions for improving adherence to medication prescribed for the primary prevention of cardiovascular disease in adults. Cochrane Database of Systematic Reviews. 2018;2018(6):CD012675.
193. Padwal RS. Designing interventions for blood pressure control in challenging settings: Active not passive intervention is needed. J Clin Hypertens (Greenwich). 2019;21(1):37-8.
194. Oyedele GT, Adedara IA, Ikeji CN, Afolabi BA, Rocha JBT, Farombi EO. Metoprolol elicits neurobehavioral insufficiency and oxidative damage in nontarget Nauphoeta cinerea nymphs. 2023.
195. Owusu I, Adomako-Boateng F, Kueffer F, Guy M, Langat C, Grossman D, et al. Improved blood pressure control and high adherence to a novel management model of hypertension care in a west african population. Hypertension. 2017;70(Supplement 1).
196. Owusu I, Adomako-Boateng F, Kueffer F, Guy M, Langat C, Grossman D, et al. Improved blood pressure control and high adherence to a novel management model of hypertension care in a west african population. Hypertension. 2017;70(Supplement 1).
197. Owusu I, Adomako-Boateng F, Kueffer F, Guy M, Lang'At C, Whitman T, et al. Increased clinic efficiency and decreased blood pressure with a novel hypertension management model of care in a west Africa. Journal of Hypertension. 2018;36(Supplement 1):e259.
198. Owusu I, Adomako-Boateng F, Kueffer F, Guy M, Lang'at C, Whitman T, et al. INCREASED CLINIC EFFICIENCY AND DECREASED BLOOD PRESSURE WITH A NOVEL HYPERTENSION MANAGEMENT MODEL OF CARE IN A WEST AFRICA. J Hypertens. 2018;36:e259.
199. Owusu I, Adomako-Boateng F, Kueffer F, Guy M, Lang'at C, Whitman T, et al. INCREASED CLINIC EFFICIENCY AND DECREASED BLOOD PRESSURE WITH A NOVEL HYPERTENSION MANAGEMENT MODEL OF CARE IN A WEST AFRICA. J Hypertens. 2018;36:e259.
200. Owusu I, Adomako-Boateng F, Kueffer F, Guy M, Lang'At C, Whitman T, et al. Increased clinic efficiency and decreased blood pressure with a novel hypertension management model of care in a west Africa. Journal of Hypertension. 2018;36(Supplement 1):e259.
201. Owusu I, Adomako-Boateng F, Kueffer F, Guy M, Lang'at C, Grossman D, et al. Abstract 091: Improved Blood Pressure Control and High Adherence to a Novel Management Model of Hypertension Care in a West African Population. Hypertension. 2017;70(Suppl_1):A091.

202. Owusu I, Adomako-Boateng F, Kueffer F, Guy M, Lang'at C, Grossman D, et al. Abstract 091: Improved Blood Pressure Control and High Adherence to a Novel Management Model of Hypertension Care in a West African Population. Hypertension. 2017;70(Suppl_1):A091.
203. Owusu I, Adomako-Boateng F, Grossman D, Kueffer F, Guy M, Lang'At C, et al. [PP.25.42] NOVEL HYPERTENSION MANAGEMENT MODEL OF CARE DECREASES BLOOD PRESSURE IN WEST AFRICAN POPULATION. J Hypertens. 2017;35:e305.
204. Owusu I, Adomako-Boateng F, Grossman D, Kueffer F, Guy M, Lang'At C, et al. [PP.25.42] NOVEL HYPERTENSION MANAGEMENT MODEL OF CARE DECREASES BLOOD PRESSURE IN WEST AFRICAN POPULATION. J Hypertens. 2017;35:e305.
205. Owsley C, Rhodes LA, McGwin G, Jr., Mennemeyer ST, Bregantini M, Patel N, et al. Eye Care Quality and Accessibility Improvement in the Community (EQUALITY) for adults at risk for glaucoma: study rationale and design. Int J Equity Health. 2015;14:135.
206. Ovbiagele B. Phone-based intervention under nurse guidance after stroke: concept for lowering blood pressure after stroke in Sub-Saharan Africa. J Stroke Cerebrovasc Dis. 2015;24(1):1-9.
207. Otu AA, Effa EE, Onwusaka O, Omoyele C, Arakelyan S, Okuzu O, et al. mHealth guideline training for non-communicable diseases in primary care facilities in Nigeria: a mixed methods pilot study. BMJ Open. 2022;12(8):e060304.
208. Otu AA, Effa EE, Onwusaka O, Omoyele C, Arakelyan S, Okuzu O, et al. mHealth guideline training for non-communicable diseases in primary care facilities in Nigeria: a mixed methods pilot study. BMJ open. 2022;12(8):e060304.
209. Otu AA, Effa EE, Onwusaka O, Omoyele C, Arakelyan S, Okuzu O, et al. MHealth guideline training for non-communicable diseases in primary care facilities in Nigeria: a mixed methods pilot study. BMJ Open. 2022;12(8):060304.
210. Otu AA, Effa EE, Onwusaka O, Omoyele C, Arakelyan S, Okuzu O, et al. mHealth guideline training for non-communicable diseases in primary care facilities in Nigeria: a mixed methods pilot study. BMJ open. 2022;12(8):e060304.
211. Otu AA, Effa EE, Onwusaka O, Omoyele C, Arakelyan S, Okuzu O, et al. mHealth guideline training for non-communicable diseases in primary care facilities in Nigeria: a mixed methods pilot study. BMJ open. 2022;12(8):e060304.
212. Otu AA, Effa EE, Onwusaka O, Omoyele C, Arakelyan S, Okuzu O, et al. MHealth guideline training for non-communicable diseases in primary care facilities in Nigeria: a mixed methods pilot study. BMJ Open. 2022;12(8):060304.
213. Otieno P, van Andel J, Agyemang C, van der Kloet B, Wilunda C, Teerling J, et al. Effect of Patient Support Groups for Hypertension on Blood Pressure among Patients with and Without Multimorbidity: Findings from a Cohort Study of Patients on a Home-Based Self-Management Program in Kenya. Global Heart. 2023;18(1):28.
214. Otieno P, van Andel J, Agyemang C, van der Kloet B, Wilunda C, Teerling J, et al. Effect of Patient Support Groups for Hypertension

on Blood Pressure among Patients with and Without Multimorbidity: Findings from a Cohort Study of Patients on a Home-Based Self-Management Program in Kenya. Global Heart. 2023;18(1):28.
215. Oronti IB, Iadanza E, Pecchia L. Hypertension Diagnosis and Management in Africa Using Mobile Phones: A Scoping Review. IEEE Rev Biomed Eng. 2022;Pp.
216. Oronti IB, Iadanza E, Pecchia L. Hypertension Diagnosis and Management in Africa Using Mobile Phones: A Scoping Review. 2022:1-17.
217. Olajide GZ. Improving Medication Adherence Among African American Adults (Ages 18-65) Diagnosed with Hypertension with the Aid of the Care Clinic Mobile Reminder App2023 2023.
218. Olajide G. Improving Medication Adherence Among African American Adults (Ages 18-65) Diagnosed with Hypertension with the Aid of the Care Clinic Mobile Reminder App: ProQuest LLC; 2023.
219. Oladapo H, Chakraborty J, editors. Blood Pressure Concerns: Findings from a Usability Study of Culturally Infused mHealth Design. 16th International Conference on Universal Access in Human-Computer Interaction, UAHCI 2022 Held as Part of the 24th HCI International Conference, HCII 2022, June 26, 2022 - July 1, 2022; 2022; Virtual, Online: Springer Science and Business Media Deutschland GmbH.
220. Oladapo H, Chakraborty J, editors. Blood Pressure Concerns: Findings from a Usability Study of Culturally Infused mHealth Design. International Conference on Human-Computer Interaction, 26 June-1 July 2022; 2022; Berlin, Germany: Springer.
221. Ola-Olorun OJ, Afolabi MO, Oyebisi TO, Ogunsina AO, Akintomide AO, Adebayo RA. Exploring medicine information needs of hypertensive patients using short message service. British Journal of Medicine and Medical Research. 2014;4(34):5368-80.
222. Ola-Olorun OJ, Afolabi MO, Oyebisi TO, Ogunsina AO, Akintomide AO, Adebayo RA. Exploring medicine information needs of hypertensive patients using short message service. British Journal of Medicine and Medical Research. 2014;4(34):5368-80.
223. Ola-Olorun OJ, Afolabi MO, Ogunsina AO, Oyebisi TO, Akinyemi OA, Akintomide AO, et al. Exploring medicine information needs of hypertensive patients using short message service (SMS) of mobilie phone. Pharmacoepidemiology and Drug Safety. 2012;21(1):116.
224. Ola-Olorun OJ, Afolabi MO, Ogunsina AO, Oyebisi TO, Akinyemi OA, Akintomide AO, et al. Exploring medicine information needs of hypertensive patients using short message service (SMS) of mobilie phone. Pharmacoepidemiology and drug safety. 2012;21(1):116.
225. Ola-Olorun OJ, Afolabi MO, Ogunsina AO, Oyebisi TO, Akinyemi OA, Akintomide AO, et al. Exploring medicine information needs of hypertensive patients using short message service (SMS) of mobilie phone. Pharmacoepidemiology and Drug Safety. 2012;21(1):116.
226. Ola-Olorun OJ, Afolabi MO, Ogunsina AO, Oyebisi TO, Akinyemi OA, Akintomide AO, et al. Exploring medicine information needs of hypertensive patients using short message service (SMS) of mobilie phone. Pharmacoepidemiology and drug safety. 2012;21(1):116.
227. Oduro-Mensah E, Agyepong IA, Frimpong E, Zweekhorst M, Vanotoo LA. Implementation of a referral and expert advice call center for

maternal and newborn care in the resource constrained health system context of the Greater Accra Region of Ghana. BMC Pregnancy and Childbirth. 2021;21(56).
228. Oduro-Mensah E, Agyepong IA, Frimpong E, Zweekhorst M, Vanotoo LA. Implementation of a referral and expert advice call center for maternal and newborn care in the resource constrained health system context of the Greater Accra Region of Ghana. BMC Pregnancy and Childbirth. 2021;21(56).
229. Oduor E, Pang C, Wachira C, Bellamy RKE, Nyota T, Remy SL, et al., editors. Exploring rural community practices in HIV management for the design of technology for hypertensive patients living with HIV. 2019 ACM Conference on Designing Interactive Systems, DIS 2019, June 23, 2019 - June 28, 2019; 2019; San Diego, CA, United states: Association for Computing Machinery.
230. Oduor E, Nyota T, Wachira C, Osebe S, Remy SL, Walcott A, editors. Medication Management Companion (MMC) for a rural kenyan community. 21st ACM Conference on Computer-Supported Cooperative Work and Social Computing, CSCW 2018, November 3, 2018 - November 7, 2018; 2018; Jersey City, NJ, United states: Association for Computing Machinery.
231. Ochieng BO, Aw J, Mannik J, Chesire J, Hawkes M. Community-based screening for cardiovascular risk using a novel mobile health (mhealth) technology in rural Kenya. American Journal of Tropical Medicine and Hygiene. 2016;95(5 Supplement 1):63-4.
232. Ochieng BO, Aw J, Mannik J, Chesire J, Hawkes M. Community-based screening for cardiovascular risk using a novel mobile health (mhealth) technology in rural Kenya. American Journal of Tropical Medicine and Hygiene. 2016;95(5 Supplement 1):63-4.
233. Nyongo DO, Wambua J. Enabling community-led integrated women health care models for women cancers screening and early detection through EMPOWER (Enabling and Motivating Partnerships Owned by Women who Engage and Reclaim their lives) project. Journal of Clinical Oncology. 2022;40(16 Supplement 1).
234. Nyongo DO, Wambua J. Enabling community-led integrated women health care models for women cancers screening and early detection through EMPOWER (Enabling and Motivating Partnerships Owned by Women who Engage and Reclaim their lives) project. Journal of Clinical Oncology. 2022;40(16 Supplement 1).
235. Nyongo D, Wambua J. Enabling community-led integrated women health care models for women cancers screening and early detection through EMPOWER (Enabling and Motivating Partnerships Owned by Women who Engage and Reclaim their lives) project. J Clin Oncol. 2022;40(16_suppl):1541.
236. Nyongo D, Wambua J. Enabling community-led integrated women health care models for women cancers screening and early detection through EMPOWER (Enabling and Motivating Partnerships Owned by Women who Engage and Reclaim their lives) project. J Clin Oncol. 2022;40(16_suppl):1541.
237. Nwokochah GO. Using Education and Mobile Medication Adherence Application to Decrease Blood Pressure among African American Adults:

ProQuest LLC; 2021.
238. Nichols M, Singh A, Sarfo FS, Treiber F, Tagge R, Jenkins C, et al. Post-intervention qualitative assessment of mobile health technology to manage hypertension among Ghanaian stroke survivors. J Neurol Sci. 2019;406:116462.
239. Nichols M, Singh A, Sarfo FS, Treiber F, Tagge R, Jenkins C, et al. Post-intervention qualitative assessment of mobile health technology to manage hypertension among Ghanaian stroke survivors. Journal of the neurological sciences. 2019;406:116462.
240. Nichols M, Singh A, Sarfo FS, Treiber F, Tagge R, Jenkins C, et al. Post-intervention qualitative assessment of mobile health technology to manage hypertension among Ghanaian stroke survivors. Journal of the Neurological Sciences. 2019;406:116462.
241. Nichols M, Singh A, Sarfo FS, Treiber F, Tagge R, Jenkins C, et al. Post-intervention qualitative assessment of mobile health technology to manage hypertension among Ghanaian stroke survivors. Journal of the neurological sciences. 2019;406:116462.
242. Nichols M, Singh A, Sarfo FS, Treiber F, Tagge R, Jenkins C, et al. Post-intervention qualitative assessment of mobile health technology to manage hypertension among Ghanaian stroke survivors. Journal of the neurological sciences. 2019;406:116462.
243. Nichols M, Singh A, Sarfo FS, Treiber F, Tagge R, Jenkins C, et al. Post-intervention qualitative assessment of mobile health technology to manage hypertension among Ghanaian stroke survivors. Journal of the Neurological Sciences. 2019;406:116462.
244. Nichols M, Sarfo FS, Singh A, Qanungo S, Treiber F, Ovbiagele B, et al. Assessing Mobile Health Capacity and Task Shifting Strategies to Improve Hypertension Among Ghanaian Stroke Survivors. Am J Med Sci. 2017;354(6):573-80.
245. Nichols M, Sarfo FS, Singh A, Qanungo S, Treiber F, Ovbiagele B, et al. Assessing Mobile Health Capacity and Task Shifting Strategies to Improve Hypertension Among Ghanaian Stroke Survivors. The American journal of the medical sciences. 2017;354(6):573-80.
246. Nichols M, Sarfo FS, Singh A, Qanungo S, Treiber F, Ovbiagele B, et al. Assessing Mobile Health Capacity and Task Shifting Strategies to Improve Hypertension Among Ghanaian Stroke Survivors. American Journal of the Medical Sciences. 2017;354(6):573-80.
247. Nichols M, Sarfo FS, Singh A, Qanungo S, Treiber F, Ovbiagele B, et al. Assessing Mobile Health Capacity and Task Shifting Strategies to Improve Hypertension Among Ghanaian Stroke Survivors. The American journal of the medical sciences. 2017;354(6):573-80.
248. Nichols M, Sarfo FS, Singh A, Qanungo S, Treiber F, Ovbiagele B, et al. Assessing Mobile Health Capacity and Task Shifting Strategies to Improve Hypertension Among Ghanaian Stroke Survivors. The American journal of the medical sciences. 2017;354(6):573-80.
249. Nichols M, Sarfo FS, Singh A, Qanungo S, Treiber F, Ovbiagele B, et al. Assessing Mobile Health Capacity and Task Shifting Strategies to Improve Hypertension Among Ghanaian Stroke Survivors. American Journal of the Medical Sciences. 2017;354(6):573-80.
250. Nichols M, PhD RN, Sarfo F, Singh A, Qanungo S, Treiber F, et

al. Assessing Mobile Health Capacity and Task Shifting Strategies to Improve Hypertension Among Ghanaian Stroke Survivors[white star],[white star][white star]. Am J Med Sci. 2017;354(6):573-80.
251. Nicholls M. The ongoing impact of COVID-19 on cardiovascular care. European Heart Journal. 2021;42(42):4292-4.
252. Nicholls M. The ongoing impact of COVID-19 on cardiovascular care. European Heart Journal. 2021;42(42):4292-4.
253. Newman-Casey PA, Niziol LM, Elam AR, Bicket AK, Killeen O, John D, et al. Michigan Screening and Intervention for Glaucoma and Eye Health Through Telemedicine Program: First-Year Outcomes and Implementation Costs. Am J Ophthalmol. 2023;251:43-51.
254. Newman-Casey PA, Aliancy J, Lu MC, Woodward MA, Hicks PM, Niziol LM, et al. Social Deprivation and the Risk of Screening Positive for Glaucoma in the MI-SIGHT Telemedicine-Based Glaucoma Detection Program. Ophthalmology. 2023;130(10):1053-65.
255. Nelissen HE, Cremers AL, Okwor TJ, Kool S, van Leth F, Brewster L, et al. Pharmacy-based hypertension care employing mHealth in Lagos, Nigeria - a mixed methods feasibility study. BMC HEALTH SERVICES RESEARCH. 2018;18.
256. Nelissen HE, Cremers AL, Okwor TJ, Kool S, van Leth F, Brewster L, et al. Pharmacy-based hypertension care employing mHealth in Lagos, Nigeria - a mixed methods feasibility study. BMC Health Serv Res. 2018;18(1):934.
257. Nelissen HE, Cremers AL, Okwor TJ, Kool S, van Leth F, Brewster L, et al. Pharmacy-based hypertension care employing mHealth in Lagos, Nigeria - a mixed methods feasibility study. BMC health services research. 2018;18(1):934.
258. Nelissen HE, Cremers AL, Okwor TJ, Kool S, van Leth F, Brewster L, et al. Pharmacy-based hypertension care employing mHealth in Lagos, Nigeria - a mixed methods feasibility study. BMC health services research. 2018;18(1):934.
259. Nelissen HE, Cremers AL, Okwor TJ, Kool S, van Leth F, Brewster L, et al. Pharmacy-based hypertension care employing mHealth in Lagos, Nigeria - a mixed methods feasibility study. BMC health services research. 2018;18(1):934.
260. Nelissen HE, Cremers AL, Okwor TJ, Kool S, van Leth F, Brewster L, et al. Pharmacy-based hypertension care employing mHealth in Lagos, Nigeria - a mixed methods feasibility study. BMC health services research. 2018;18(1):934.
261. Nelissen HE, Cremers AL, Okwor TJ, Kool S, van Leth F, Brewster L, et al. Pharmacy-based hypertension care employing mHealth in Lagos, Nigeria - a mixed methods feasibility study. BMC health services research. 2018;18(1):934.
262. Nelissen HE, Cremers AL, Okwor TJ, Kool S, Leth Fv, Brewster L, et al. Pharmacy-based hypertension care employing mHealth in Lagos, Nigeria - a mixed methods feasibility study. BMC Health Services Research. 2018;18(934).
263. Nelissen HE, Cremers AL, Okwor TJ, Kool S, Leth Fv, Brewster L, et al. Pharmacy-based hypertension care employing mHealth in Lagos, Nigeria - a mixed methods feasibility study. BMC Health Services

Research. 2018;18(934).
264. Ndejjo R, Nuwaha F, Bastiaens H, Wanyenze RK, Musinguzi G. Cardiovascular disease prevention knowledge and associated factors among adults in Mukono and Buikwe districts in Uganda. BMC Public Health. 2020;20(1151).
265. Ndejjo R, Nuwaha F, Bastiaens H, Wanyenze RK, Musinguzi G. Cardiovascular disease prevention knowledge and associated factors among adults in Mukono and Buikwe districts in Uganda. BMC Public Health. 2020;20(1151).
266. Ndaribitse C, Durieux ME, Adorno W, Brown DE, Tsang S, Naik BI. Digitization of Symbol-Denoted Blood Pressure Data From Intraoperative Paper Health Records in a Low-Middle-Income Country Using Deep Image Segmentation and Associated Postoperative Outcomes: A Feasibility Study. Anesth Analg. 2023;136(4):753-60.
267. Nakwafila O, Mashamba-Thompson T, Godi A, Sartorius B. A Cross-Sectional Study on Hypertension Medication Adherence in a High-Burden Region in Namibia: Exploring Hypertension Interventions and Validation of the Namibia Hill-Bone Compliance Scale. International Journal of Environmental Research and Public Health. 2022;19(7):4416.
268. Nakwafila O, Mashamba-Thompson T, Godi A, Sartorius B. A Cross-Sectional Study on Hypertension Medication Adherence in a High-Burden Region in Namibia: Exploring Hypertension Interventions and Validation of the Namibia Hill-Bone Compliance Scale. International Journal of Environmental Research and Public Health. 2022;19(7):4416.
269. Müller AM, Alley S, Schoeppe S, Vandelanotte C. The effectiveness of e-& mHealth interventions to promote physical activity and healthy diets in developing countries: A systematic review. Int J Behav Nutr Phys Act. 2016;13(1):109.
270. Mullen L, Richardson C, Gardner A, Jones T, Tolley C. Medicines management of long-term conditions during the COVID19 pandemic: a narrative systematic review. International Journal of Pharmacy Practice. 2023;31(Supplement 1):i14-i5.
271. Mullen L, Richardson C, Gardner A, Jones T, Tolley C. Medicines management of long-term conditions during the COVID19 pandemic: a narrative systematic review. International Journal of Pharmacy Practice. 2023;31(Supplement 1):i14-i5.
272. Muli E, Waithanji R, Kamita M, Gitau T, Obonyo I, Mweni S, et al. Leveraging technology for health services continuity in times of COVID-19 pandemic: Patient follow-up, and mitigation of worse patient outcomes. J Glob Health. 2021;11:05024.
273. Muli E, Waithanji R, Kamita M, Gitau T, Obonyo I, Mweni S, et al. Leveraging technology for health services continuity in times of COVID-19 pandemic: Patient follow-up, and mitigation of worse patient outcomes. Journal of global health. 2021;11:05024.
274. Muli E, Waithanji R, Kamita M, Gitau T, Obonyo I, Mweni S, et al. Leveraging technology for health services continuity in times of COVID-19 pandemic: Patient follow-up, and mitigation of worse patient outcomes. Journal of global health. 2021;11:05024.
275. Muli E, Waithanji R, Kamita M, Gitau T, Obonyo I, Mweni S, et al. Leveraging technology for health services continuity in times of

COVID-19 pandemic: Patient follow-up, and mitigation of worse patient outcomes. Journal of global health. 2021;11:05024.
276. Muli E, Waithanji R, Kamita M, Gitau T, Obonyo I, Mweni S, et al. Leveraging technology for health services continuity in times of COVID-19 pandemic: Patient follow-up, and mitigation of worse patient outcomes. Journal of global health. 2021;11:05024.
277. Muli E, Waithanji R, Kamita M, Gitau T, Obonyo I, Mweni S, et al. Leveraging technology for health services continuity in times of COVID-19 pandemic: Patient follow-up, and mitigation of worse patient outcomes. Journal of global health. 2021;11:05024.
278. Muli E, Waithanji R, Kamita M, Gitau T, Obonyo I, Mweni S, et al. Leveraging technology for health services continuity in times of COVID-19 pandemic: Patient follow-up, and mitigation of worse patient outcomes. Journal of global health. 2021;11:05024.
279. Muli E, Obonyo I, Waithanji R, Kamita M, Gitau T, Kirira P, et al. Leveraging technology for health services continuity in times of COVID-19 pandemic: patient follow-up, and mitigation of worse patient outcomes. Journal of Global Health. 2021;11.
280. Muli E, Obonyo I, Waithanji R, Kamita M, Gitau T, Kirira P, et al. Leveraging technology for health services continuity in times of COVID-19 pandemic: patient follow-up, and mitigation of worse patient outcomes. Journal of Global Health. 2021;11.
281. Muiruri C, Manavalan P, Jazowski SA, Knettel BA, Vilme H, Zullig LL. Opportunities to Leverage Telehealth Approaches Along the Hypertension Control Cascade in Sub-Saharan Africa. Curr Hypertens Rep. 2019;21(10):75.
282. Mugisha JO, Seeley J. "We shall have gone to a higher standard": Training village heath teams (VHTs) to use a smartphone-guided intervention to link older Ugandans with hypertension and diabetes to care. AAS Open Res. 2020;3:25.
283. Mugisha JO, Seeley J. "We shall have gone to a higher standard": Training village heath teams (VHTs) to use a smartphone-guided intervention to link older Ugandans with hypertension and diabetes to care. AAS open research. 2020;3:25.
284. Mugisha JO, Seeley J. "We shall have gone to a higher standard": Training village heath teams (VHTs) to use a smartphone-guided intervention to link older Ugandans with hypertension and diabetes to care. AAS open research. 2020;3:25.
285. Mugisha JO, Seeley J. "We shall have gone to a higher standard": Training village heath teams (VHTs) to use a smartphone-guided intervention to link older Ugandans with hypertension and diabetes to care. AAS open research. 2020;3:25.
286. Mugisha JO, Seeley J. "We shall have gone to a higher standard": Training village heath teams (VHTs) to use a smartphone-guided intervention to link older Ugandans with hypertension and diabetes to care. AAS open research. 2020;3:25.
287. Mugabirwe B, Flickinger T, Cox L, Ariho P, Dillingham R, Okello S. Acceptability and feasibility of a mobile health application for blood pressure monitoring in rural Uganda. JAMIA Open. 2021;4(3):ooaa068.

288. Mugabirwe B, Flickinger T, Cox L, Ariho P, Dillingham R, Okello S. Acceptability and feasibility of a mobile health application for blood pressure monitoring in rural Uganda. JAMIA open. 2021;4(3):ooaa068.
289. Mugabirwe B, Flickinger T, Cox L, Ariho P, Dillingham R, Okello S. Acceptability and feasibility of a mobile health application for blood pressure monitoring in rural Uganda. JAMIA Open. 2021;4(3):ooaa068.
290. Mugabirwe B, Flickinger T, Cox L, Ariho P, Dillingham R, Okello S. Acceptability and feasibility of a mobile health application for blood pressure monitoring in rural Uganda. JAMIA open. 2021;4(3):ooaa068.
291. Mugabirwe B, Flickinger T, Cox L, Ariho P, Dillingham R, Okello S. Acceptability and feasibility of a mobile health application for blood pressure monitoring in rural Uganda. JAMIA Open. 2021;4(3):ooaa068.
292. Monroe VDZ. Testing a Smartphone Application Intervention to Improve Medication Adherence in African American Female Clinic Patients with Unstable High Blood Pressure: A Two-group Randomized Control Trial2018 2018.
293. Mobisson N, Bankole F, Njoku K, Adediran O, Adebo A. Leveraging public-private partnerships to provide a person-centred high-tech high-touch approach to obesity management in Lagos, Nigeria. Obesity Reviews. 2020;21(SUPPL 1).
294. Mobisson N, Bankole F, Njoku K, Adediran O, Adebo A. Leveraging public-private partnerships to provide a person-centred high-tech high-touch approach to obesity management in Lagos, Nigeria. Obesity Reviews. 2020;21(SUPPL 1).
295. Migneault JP, Dedier JJ, Wright JA, Heeren T, Campbell MK, Morisky DE, et al. A culturally adapted telecommunication system to improve physical activity, diet quality, and medication adherence among hypertensive African-Americans: a randomized controlled trial. Ann Behav Med. 2012;43(1):62-73.
296. Mfinanga SG, Mbuli TW, Moshiro C. Leveraging remote consultations in resource-limited settings. The Lancet Global Health. 2023;11(11):e1674-e5.
297. Mfinanga SG, Mbuli TW, Moshiro C. Leveraging remote consultations in resource-limited settings. The Lancet Global Health. 2023;11(11):e1674-e5.
298. Meurer WJ, Dinh M, Kidwell KM, Flood A, Champoux E, Whitfield C, et al. Reach out behavioral intervention for hypertension initiated in the emergency department connecting multiple health systems: study protocol for a randomized control trial. Trials. 2020;21(1):456.
299. Mercer T, Nulu S, Vedanthan R. Innovative Implementation Strategies for Hypertension Control in Low- and Middle-Income Countries: a Narrative Review. Curr Hypertens Rep. 2020;22(5):39.
300. Mburu S, editor A predictive model for optimizing acceptance and use of mHealth interventions in low-resource settings: A case of Mamacare prototype. IEEE AFRICON 2017, September 18, 2017 - September 20, 2017; 2017; Cape Town, South africa: Institute of Electrical and

Electronics Engineers Inc.
301. Mburu S, editor A predictive model for optimizing acceptance and use of mHealth interventions in low-resource settings: A case of Mamacare prototype. 2017 IEEE AFRICON, 18-20 Sept 2017; 2017; Piscataway, NJ, USA: IEEE.
302. Maurizi N, Peschiera S, Ochola E, Fumagalli C, Rughetti R, Avvantaggiato F, et al. Telemedicine cardiovascular screening program in low-income settings by smartphone based technology. European Heart Journal. 2018;39(Supplement 1):860-1.
303. Maurizi N, Peschiera S, Ochola E, Fumagalli C, Rughetti R, Avvantaggiato F, et al. Telemedicine cardiovascular screening program in low-income settings by smartphone based technology. European Heart Journal. 2018;39(Supplement 1):860-1.
304. Maurizi N, Imberti JF, Faragli A, Targetti M, Baldini K, Sall A, et al. Comparative analysis of a 4-lead portable smartphone-based versus standard 12-lead electrocardiograph for cardiovascular screening in low-income settings. European Heart Journal. 2016;37(Supplement 1):826.
305. Maurizi N, Imberti JF, Faragli A, Targetti M, Baldini K, Sall A, et al. Comparative analysis of a 4-lead portable smartphone-based versus standard 12-lead electrocardiograph for cardiovascular screening in low-income settings. European Heart Journal. 2016;37(Supplement 1):826.
306. Masucci MM, Homko C, Santamore WP, Berger P, McConnell TR, Shirk G, et al. Cardiovascular disease prevention for underserved patients using the internet: Bridging the digital divide. Telemedicine and e-Health. 2006;12(1):58-65.
307. Masucci MM, Homko C, Santamore WP, Berger P, McConnell TR, Shirk G, et al. Cardiovascular disease prevention for underserved patients using the Internet: bridging the digital divide. Telemedicine Journal and e-Health. 2006;12(1):58-65.
308. Martin TD, Green MS, Whitehead MT, Scheett TP, Webster MJ, Hudson GM. Six weeks of oral Echinacea purpurea supplementation does not enhance the production of serum erythropoietin or erythropoietic status in recreationally active males with above-average aerobic fitness. Applied physiology, nutrition & metabolism. 2019;44(7):791.
309. Martin TD, Green MS, Whitehead MT, Scheett TP, Webster MJ, Hudson GM. Six weeks of oral Echinacea purpurea supplementation does not enhance the production of serum erythropoietin or erythropoietic status in recreationally active males with above-average aerobic fitness. Applied physiology, nutrition & metabolism. 2019;44(7):791.
310. Maduka O, Tobin-West C. Is living in a gas-flaring host community associated with being hypertensive? Evidence from the Niger Delta region of Nigeria. BMJ Glob Health. 2017;2(4):e000413.
311. Maduka O, Tobin-West C. Is living in a gas-flaring host community associated with being hypertensive? Evidence from the Niger Delta region of Nigeria. BMJ global health. 2017;2(4):e000413.
312. Maduka O, Tobin-West C. Is living in a gas-flaring host community associated with being hypertensive? Evidence from the Niger Delta region of Nigeria. BMJ Global Health. 2017;2(4):e000413.

313. Maduka O, Tobin-West C. Is living in a gas-flaring host community associated with being hypertensive? Evidence from the Niger Delta region of Nigeria. BMJ global health. 2017;2(4):e000413.
314. Maduka O, Tobin-West C. Is living in a gas-flaring host community associated with being hypertensive? Evidence from the Niger Delta region of Nigeria. BMJ Global Health. 2017;2(4):e000413.
315. Maar MA, Yeates K, Toth Z, Barron M, Boesch L, Hua-Stewart D, et al. Unpacking the Black Box: A Formative Research Approach to the Development of Theory-Driven, Evidence-Based, and Culturally Safe Text Messages in Mobile Health Interventions. JMIR Mhealth Uhealth. 2016;4(1):e10.
316. Maar MA, Yeates K, Toth Z, Barron M, Boesch L, Hua-Stewart D, et al. Unpacking the Black Box: A Formative Research Approach to the Development of Theory-Driven, Evidence-Based, and Culturally Safe Text Messages in Mobile Health Interventions. JMIR mHealth and uHealth. 2016;4(1):e10.
317. Maar MA, Yeates K, Toth Z, Barron M, Boesch L, Hua-Stewart D, et al. Unpacking the Black Box: A Formative Research Approach to the Development of Theory-Driven, Evidence-Based, and Culturally Safe Text Messages in Mobile Health Interventions. JMIR mHealth and uHealth. 2016;4(1):e10.
318. Maar MA, Yeates K, Perkins N, Boesch L, Hua-Stewart D, Liu P, et al. A Framework for the Study of Complex mHealth Interventions in Diverse Cultural Settings. JMIR Mhealth Uhealth. 2017;5(4):e47.
319. Lynch CP, Williams JS, K JR, R GK, Egede LE. Tablet-Aided BehavioraL intervention EffecT on Self-management skills (TABLETS) for Diabetes. Trials. 2016;17:157.
320. Lyles E, Paik K, Kiogora J, Hussein H, Cordero Morales A, Kiapi L, et al. Adoption of Electronic Medical Records for Chronic Disease Care in Kenyan Refugee Camps: Quantitative and Qualitative Prospective Evaluation. JMIR Mhealth Uhealth. 2023;11:e43878.
321. Lyles E, Paik K, Kiogora J, Hussein H, Cordero Morales A, Kiapi L, et al. Adoption of Electronic Medical Records for Chronic Disease Care in Kenyan Refugee Camps: Quantitative and Qualitative Prospective Evaluation. JMIR mHealth and uHealth. 2023;11:e43878.
322. Lyles E, Paik K, Kiogora J, Hussein H, Cordero Morales A, Kiapi L, et al. Adoption of Electronic Medical Records for Chronic Disease Care in Kenyan Refugee Camps: Quantitative and Qualitative Prospective Evaluation. JMIR mHealth and uHealth. 2023;11:e43878.
323. Lyles E, Paik K, Kiogora J, Hussein H, Cordero Morales A, Kiapi L, et al. Adoption of Electronic Medical Records for Chronic Disease Care in Kenyan Refugee Camps: Quantitative and Qualitative Prospective Evaluation. JMIR mHealth and uHealth. 2023;11:e43878.
324. Lyles E, Paik K, Kiogora J, Hussein H, Cordero Morales A, Kiapi L, et al. Adoption of Electronic Medical Records for Chronic Disease Care in Kenyan Refugee Camps: Quantitative and Qualitative Prospective Evaluation. JMIR mHealth and uHealth. 2023;11:e43878.
325. Li WW, Lai WS. [The Use of Telemedicine Interventions to Improve Hypertension Management Among Racial Ethnic Minorities: A Systematic Review]. Hu Li Za Zhi. 2016;63(4):25-34.

326. Li C, Su HB, Liu XY, Zhang LN, Hu JH. High-dependency units play a key role in the treatment of a Chinese military patient who developed liver failure while abroad. Military Medical Research. 2019;6(1):29.
327. Li C, Su HB, Liu XY, Zhang LN, Hu JH. High-dependency units play a key role in the treatment of a Chinese military patient who developed liver failure while abroad. Military Medical Research. 2019;6(1):29.
328. Leon N, Surender R, Bobrow K, Muller J, Farmer A. Improving treatment adherence for blood pressure lowering via mobile phone SMS-messages in South Africa: a qualitative evaluation of the SMS-text Adherence SuppoRt (StAR) trial. BMC Fam Pract. 2015;16:80.
329. Leon N, Surender R, Bobrow K, Muller J, Farmer A. Improving treatment adherence for blood pressure lowering via mobile phone SMS-messages in South Africa: a qualitative evaluation of the SMS-text Adherence SuppoRt (StAR) trial. BMC family practice. 2015;16:80.
330. Leon N, Surender R, Bobrow K, Muller J, Farmer A. Improving treatment adherence for blood pressure lowering via mobile phone SMS-messages in South Africa: a qualitative evaluation of the SMS-text Adherence SuppoRt (StAR) trial. BMC Family Practice. 2015;16(80).
331. Leon N, Surender R, Bobrow K, Muller J, Farmer A. Improving treatment adherence for blood pressure lowering via mobile phone SMS-messages in South Africa: a qualitative evaluation of the SMS-text Adherence SuppoRt (StAR) trial. BMC family practice. 2015;16:80.
332. Leon N, Surender R, Bobrow K, Muller J, Farmer A. Improving treatment adherence for blood pressure lowering via mobile phone SMS-messages in South Africa: a qualitative evaluation of the SMS-text Adherence SuppoRt (StAR) trial. BMC family practice. 2015;16:80.
333. Leon N, Surender R, Bobrow K, Muller J, Farmer A. Improving treatment adherence for blood pressure lowering via mobile phone SMS-messages in South Africa: a qualitative evaluation of the SMS-text Adherence SuppoRt (StAR) trial. BMC family practice. 2015;16:80.
334. Leon N, Surender R, Bobrow K, Muller J, Farmer A. Improving treatment adherence for blood pressure lowering via mobile phone SMS-messages in South Africa: a qualitative evaluation of the SMS-text Adherence SuppoRt (StAR) trial. BMC family practice. 2015;16:80.
335. Leon N, Surender R, Bobrow K, Muller J, Farmer A. Improving treatment adherence for blood pressure lowering via mobile phone SMS-messages in South Africa: a qualitative evaluation of the SMS-text Adherence SuppoRt (StAR) trial. BMC Family Practice. 2015;16(80).
336. Leon N, Surender R, Bobrow K, Muller J, Farmer A. Improving treatment adherence for blood pressure lowering via mobile phone SMS-messages in South Africa: a qualitative evaluation of the SMS-text Adherence SuppoRt (StAR) trial. BMC family practice. 2015;16:80.
337. Leon N, Surender R, Bobrow K, Muller J, Farmer A. Improving treatment adherence for blood pressure lowering via mobile phone SMS-messages in South Africa: a qualitative evaluation of the SMS-text Adherence SuppoRt (StAR) trial. BMC family practice. 2015;16:80.
338. Leon N, Namadingo H, Bobrow K, Cooper S, Crampin A, Pauly B, et al. Intervention development of a brief messaging intervention for

a randomised controlled trial to improve diabetes treatment adherence in sub-Saharan Africa. BMC Public Health. 2021;21(1):147.
339. Leon N, Namadingo H, Bobrow K, Cooper S, Crampin A, Pauly B, et al. Intervention development of a brief messaging intervention for a randomised controlled trial to improve diabetes treatment adherence in sub-Saharan Africa. BMC public health. 2021;21(1):147.
340. Leon N, Namadingo H, Bobrow K, Cooper S, Crampin A, Pauly B, et al. Intervention development of a brief messaging intervention for a randomised controlled trial to improve diabetes treatment adherence in sub-Saharan Africa. BMC Public Health. 2021;21(147).
341. Leon N, Namadingo H, Bobrow K, Cooper S, Crampin A, Pauly B, et al. Intervention development of a brief messaging intervention for a randomised controlled trial to improve diabetes treatment adherence in sub-Saharan Africa. BMC public health. 2021;21(1):147.
342. Leon N, Namadingo H, Bobrow K, Cooper S, Crampin A, Pauly B, et al. Intervention development of a brief messaging intervention for a randomised controlled trial to improve diabetes treatment adherence in sub-Saharan Africa. BMC public health. 2021;21(1):147.
343. Leon N, Namadingo H, Bobrow K, Cooper S, Crampin A, Pauly B, et al. Intervention development of a brief messaging intervention for a randomised controlled trial to improve diabetes treatment adherence in sub-Saharan Africa. BMC public health. 2021;21(1):147.
344. Leon N, Namadingo H, Bobrow K, Cooper S, Crampin A, Pauly B, et al. Intervention development of a brief messaging intervention for a randomised controlled trial to improve diabetes treatment adherence in sub-Saharan Africa. BMC public health. 2021;21(1):147.
345. Leon N, Namadingo H, Bobrow K, Cooper S, Crampin A, Pauly B, et al. Intervention development of a brief messaging intervention for a randomised controlled trial to improve diabetes treatment adherence in sub-Saharan Africa. BMC Public Health. 2021;21(147).
346. Leon N, Namadingo H, Bobrow K, Cooper S, Crampin A, Pauly B, et al. Intervention development of a brief messaging intervention for a randomised controlled trial to improve diabetes treatment adherence in sub-Saharan Africa. BMC public health. 2021;21(1):147.
347. Leon N, Namadingo H, Bobrow K, Cooper S, Crampin A, Pauly B, et al. Intervention development of a brief messaging intervention for a randomised controlled trial to improve diabetes treatment adherence in sub-Saharan Africa. BMC public health. 2021;21(1):147.
348. Lee Y, Chang G, Chang H. Effects of education and support groups organized by IBCLCs in early postpartum on breastfeeding. Midwifery. 2019;75:5.
349. Lee Y, Chang G, Chang H. Effects of education and support groups organized by IBCLCs in early postpartum on breastfeeding. Midwifery. 2019;75:5.
350. Lee NS, Anastos-Wallen R, Chaiyachati KH, Reitz C, Asch DA, Mehta SJ. Clinician Decisions After Notification of Elevated Blood Pressure Measurements From Patients in a Remote Monitoring Program. JAMA Netw Open. 2022;5(1):e2143590.
351. Lee ES, Vedanthan R, Jeemon P, Kamano JH, Kudesia P, Rajan V, et al. Quality Improvement in Cardiovascular Disease Care. In:

Prabhakaran D, Anand S, Gaziano TA, Mbanya JC, Wu Y, Nugent R, editors. Cardiovascular, Respiratory, and Related Disorders. Washington (DC): The International Bank for Reconstruction and Development / The World Bank
© 2017 International Bank for Reconstruction and Development / The World Bank.; 2017.

352. Lee ES, Vedanthan R, Jeemon P, Kamano JH, Kudesia P, Rajan V, et al. Quality Improvement in Cardiovascular Disease Care Cardiovascular, Respiratory, and Related Disorders. 2017.

353. Lee ES, Vedanthan R, Jeemon P, Kamano JH, Kudesia P, Rajan V, et al. Quality Improvement in Cardiovascular Disease Care Cardiovascular, Respiratory, and Related Disorders. 2017.

354. Lederer S, Ruggiero L, Sisen NM, Lepain N, O'Connor KG, Wang Y, et al. The National Kidney Foundation of Illinois KidneyMobile: a mobile resource for community based screenings of chronic kidney disease and its risk factors. BMC Nephrol. 2018;19(1):295.

355. Le H, Schiff N, Plessis JD, Hoang D, editors. A pervasive tele-health system for continual and low intrusive monitoring using peer-to-peer networks. 5th International Conference on Information Technology and Applications, ICITA 2008, June 23, 2008 - June 26, 2008; 2008; Cairns, QLD, Australia: International Conference on Information Technology and Applications.

356. Le H, Schiff N, Du Plessis J, Hoang DB. A pervasive tele-health system for continual and low intrusive monitoring using peer-to-peer networks. International Journal of Computer Applications in Technology. 2009;34(4):330-4.

357. Lauffenburger JC, Choudhry NK. Text Messaging and Patient Engagement in an Increasingly Mobile World. Circulation. 2016;133(6):555-6.

358. Lauffenburger JC, Choudhry NK. Text Messaging and Patient Engagement in an Increasingly Mobile World. Circulation. 2016;133(6):555-6.

359. Lanuit R, Klotz F, Delegue P, Ribeau G, Capron A. [Value of hepatosplenic ultrasonography in the surveillance of Schistosoma mansoni endemics (a study conducted in the Richard Toll region of Senegal)]. Med Trop (Mars). 1996;56(3):271-4.

360. Lanuit R, Klotz F, Delegue P, Ribeau G, Capron A. [Value of hepatosplenic ultrasonography in the surveillance of Schistosoma mansoni endemics (a study conducted in the Richard Toll region of Senegal)]. Interet de l'echographie Hepato-splenique dans la surveillance de l'endemie a Schistosoma mansoni (a propos d'une etude realisee dans la region de Richard Toll au Senegal). 1996;56(3):271-4.

361. Lanuit R, Klotz F, Delegue P, Ribeau G, Capron A. [Value of hepatosplenic ultrasonography in the surveillance of Schistosoma mansoni endemics (a study conducted in the Richard Toll region of Senegal)]. Interet de l'echographie Hepato-splenique dans la surveillance de l'endemie a Schistosoma mansoni (a propos d'une etude realisee dans la region de Richard Toll au Senegal). 1996;56(3):271-4.

362. Lanuit R, Klotz F, Delegue P, Ribeau G, Capron A. [Value of hepatosplenic ultrasonography in the surveillance of Schistosoma

mansoni endemics (a study conducted in the Richard Toll region of Senegal)]. Interet de l'echographie Hepato-splenique dans la surveillance de l'endemie a Schistosoma mansoni (a propos d'une etude realisee dans la region de Richard Toll au Senegal). 1996;56(3):271-4.
363. Lakshminarayan K, Murray TA, Westberg SM, Connett J, Overton V, Nyman JA, et al. Mobile Health Intervention to Close the Guidelines-To-Practice Gap in Hypertension Treatment: Protocol for the mGlide Randomized Controlled Trial. JMIR Res Protoc. 2021;10(1):e25424.
364. Lakshminarayan K, Murray TA, Westberg SM, Connett J, Overton V, Nyman JA, et al. Mobile Health Intervention to Close the Guidelines-To-Practice Gap in Hypertension Treatment: Protocol for the mGlide Randomized Controlled Trial. JMIR research protocols. 2021;10(1):e25424.
365. Lakshminarayan K, Murray TA, Westberg SM, Connett J, Overton V, Nyman JA, et al. Mobile Health Intervention to Close the Guidelines-To-Practice Gap in Hypertension Treatment: Protocol for the mGlide Randomized Controlled Trial. JMIR research protocols. 2021;10(1):e25424.
366. Lakshminarayan K, Murray TA, Westberg SM, Connett J, Overton V, Nyman JA, et al. Mobile Health Intervention to Close the Guidelines-To-Practice Gap in Hypertension Treatment: Protocol for the mGlide Randomized Controlled Trial. JMIR research protocols. 2021;10(1):e25424.
367. Lakshminarayan K, Murray TA, Westberg SM, Connett J, Overton V, Nyman JA, et al. Mobile Health Intervention to Close the Guidelines-To-Practice Gap in Hypertension Treatment: Protocol for the mGlide Randomized Controlled Trial. JMIR research protocols. 2021;10(1):e25424.
368. Laken MA, O'Rourke K, Duffy NG, Swinton R, Jordan J. Use of the Internet for health information by African-Americans with modifiable risk factors for cardiovascular disease. Telemedicine Journal and e-Health. 2004;10(3):304-10.
369. Laken MA, O'Rourke K, Duffy NG, Swinton R, Jordan J. Use of the Internet for health information by African-Americans with modifiable risk factors for cardiovascular disease. Telemedicine and e-Health. 2004;10(3):304-10.
370. Kumpusch H, Hayn D, Kreiner K, Falgenhauer M, Mor J, Schreier G, editors. A mobile phone based telemonitoring concept for the simultanous acquisition of biosignals physiological parameters. 13th World Congress on Medical and Health Informatics, Medinfo 2010, September 12, 2010 - September 15, 2010; 2010; Cape Town, South africa: IOS Press BV.
371. Komodromos M. Interactive radio, social network sites and development in Africa: a literature review study. Journal of Enterprising Communities: People and Places in the Global Economy. 2021;15(2):282-95.
372. KofiOwusu I, Adomako-Boateng F, Grossman D, Kueffer F, Guy M, Lang'At C, et al. Novel hypertension management model of care decreases blood pressure in West African population. Journal of

Hypertension. 2017;35(Supplement 2):e305.
373. KofiOwusu I, Adomako-Boateng F, Grossman D, Kueffer F, Guy M, Lang'At C, et al. Novel hypertension management model of care decreases blood pressure in West African population. Journal of Hypertension. 2017;35(Supplement 2):e305.
374. KofiOwusu I, Adomako-Boateng F, Grossman D, Kueffer F, Guy M, Lang'At C, et al. Novel hypertension management model of care decreases blood pressure in West African population. Journal of Hypertension. 2016;34(Supplement 2):e305.
375. KofiOwusu I, Adomako-Boateng F, Grossman D, Kueffer F, Guy M, Lang'At C, et al. Novel hypertension management model of care decreases blood pressure in West African population. Journal of Hypertension. 2016;34(Supplement 2):e305.
376. Kobe EA, Diamantidis CJ, Bosworth HB, Davenport CA, Oakes M, Alexopoulos AS, et al. Racial Differences in the Effectiveness of a Multifactorial Telehealth Intervention to Slow Diabetic Kidney Disease. Med Care. 2020;58(11):968-73.
377. Kleczka B, Musiega A, Rabut G, Wekesa P, Mwaniki P, Marx M, et al. Rubber stamp templates for improving clinical documentation: a paper-based, m-Health approach for quality improvement in low-resource settings. International Journal of Medical Informatics. 2018;114:121-9.
378. Kleczka B, Musiega A, Rabut G, Wekesa P, Mwaniki P, Marx M, et al. Rubber stamp templates for improving clinical documentation: a paper-based, m-Health approach for quality improvement in low-resource settings. International Journal of Medical Informatics. 2018;114:121-9.
379. Kleczka B, Musiega A, Rabut G, Wekesa P, Mwaniki P, Marx M, et al. Rubber stamp templates for improving clinical documentation: A paper-based, m-Health approach for quality improvement in low-resource settings. Int J Med Inform. 2018;114:121-9.
380. Kleczka B, Musiega A, Rabut G, Wekesa P, Mwaniki P, Marx M, et al. Rubber stamp templates for improving clinical documentation: A paper-based, m-Health approach for quality improvement in low-resource settings. International journal of medical informatics. 2018;114:121-9.
381. Kleczka B, Musiega A, Rabut G, Wekesa P, Mwaniki P, Marx M, et al. Rubber stamp templates for improving clinical documentation: A paper-based, m-Health approach for quality improvement in low-resource settings. International Journal of Medical Informatics. 2018;114:121-9.
382. Kleczka B, Musiega A, Rabut G, Wekesa P, Mwaniki P, Marx M, et al. Rubber stamp templates for improving clinical documentation: A paper-based, m-Health approach for quality improvement in low-resource settings. International journal of medical informatics. 2018;114:121-9.
383. Kleczka B, Musiega A, Rabut G, Wekesa P, Mwaniki P, Marx M, et al. Rubber stamp templates for improving clinical documentation: A paper-based, m-Health approach for quality improvement in low-resource settings. International journal of medical informatics.

2018;114:121-9.
384. Kleczka B, Musiega A, Rabut G, Wekesa P, Mwaniki P, Marx M, et al. Rubber stamp templates for improving clinical documentation: A paper-based, m-Health approach for quality improvement in low-resource settings. International Journal of Medical Informatics. 2018;114:121-9.
385. Kleczka B, Musiega A, Rabut G, Wekesa P, Mwaniki P, Marx M, et al. Rubber stamp templates for improving clinical documentation: A paper-based, m-Health approach for quality improvement in low-resource settings. International Journal of Medical Informatics. 2018;114:121-9.
386. Kleczka B, Musiega A, Rabut G, Wekesa P, Mwaniki P, Marx M, et al. Rubber stamp templates for improving clinical documentation: A paper-based, m-Health approach for quality improvement in low-resource settings. International Journal of Medical Informatics. 2018;114:121-9.
387. Kingue S, Angandji P, Menanga AP, Ashuntantang G, Sobngwi E, Dossou-Yovo RA, et al. Efficiency of an intervention package for arterial hypertension comprising telemanagement in a Cameroonian rural setting: The TELEMED-CAM study. Pan Afr Med J. 2013;15:153.
388. Khosla K, Suresh S, Mueller A, Perdigao JL, Stewart K, Duncan C, et al. Elimination of racial disparities in postpartum hypertension follow-up after incorporation of telehealth into a quality bundle. Am J Obstet Gynecol MFM. 2022;4(3):100580.
389. Karlen W, Lim J, Ansermino JM, Dumont GA, Scheffer C, editors. Recognition of correct finger placement for photoplethysmographic imaging. 2013 35th Annual International Conference of the IEEE Engineering in Medicine and Biology Society (EMBC), 3-7 July 2013; 2013; Piscataway, NJ, USA: IEEE.
390. Kadima NJ, Nyiranteziryayo R, Umumararungu T, Adedeji AA. Use of mobile phones for patient self-reporting adverse drug reactions: A pilot study at a tertiary hospital in Rwanda. Health and Technology. 2021;11(1):185-91.
391. Kadima NJ, Nyiranteziryayo R, Umumararungu T, Adedeji AA. Use of mobile phones for patient self-reporting adverse drug reactions: A pilot study at a tertiary hospital in Rwanda. Health and Technology. 2021;11(1):185-91.
392. Joubert J, Nkomazana O, Mompati K, Joubert L, Preux PM, La Croix P, et al. A community survey of cardiovascular risk factors in an urban population in Botswana exploring potential for telemedicine. European Research in Telemedicine. 2014;3(3):95-103.
393. Joubert J, Nkomazana O, Mompati K, Joubert L, Preux PM, La Croix P, et al. A community survey of cardiovascular risk factors in an urban population in Botswana exploring potential for telemedicine. European research in telemedicine. 2014;3(3):95.
394. Joubert J, Nkomazana O, Mompati K, Joubert L, Preux PM, La Croix P, et al. A community survey of cardiovascular risk factors in an urban population in Botswana exploring potential for telemedicine. European Research in Telemedicine. 2014;3(3):95-103.
395. Joubert J, Nkomazana O, Mompati K, Joubert L, Preux PM, La

Croix P, et al. A community survey of cardiovascular risk factors in an urban population in Botswana exploring potential for telemedicine. European research in telemedicine. 2014;3(3):95.
396. Joshi R, Thrift AG, Smith C, Praveen D, Vedanthan R, Gyamfi J, et al. Task-shifting for cardiovascular risk factor management: lessons from the Global Alliance for Chronic Diseases. BMJ Glob Health. 2018;3(Suppl 3):e001092.
397. Jhamb M, Cavanaugh KL, Bian A, Chen G, Ikizler TA, Unruh ML, et al. Disparities in Electronic Health Record Patient Portal Use in Nephrology Clinics. Clin J Am Soc Nephrol. 2015;10(11):2013-22.
398. Jeemon P, Séverin T, Amodeo C, Balabanova D, Campbell NRC, Gaita D, et al. World Heart Federation Roadmap for Hypertension - A 2021 Update. Glob Heart. 2021;16(1):63.
399. James DCS, Kang P, McQueen D. African American Women's Willingness to Participate in Online Health Communities During COVID-19. Telemed J E Health. 2023;29(5):657-64.
400. Jackson GL, Oddone EZ, Olsen MK, Powers BJ, Grubber JM, McCant F, et al. Racial differences in the effect of a telephone-delivered hypertension disease management program. J Gen Intern Med. 2012;27(12):1682-9.
401. Ibekwe T, Folorunso D, Ebuta A, Amodu J, Nwegbu M, Mairami Z, et al. EVALUATION OF THE ENVIRONMENTAL NOISE LEVELS IN ABUJA MUNICIPALITY USING MOBILE PHONES. Ann Ib Postgrad Med. 2016;14(2):58-64.
402. Hoodbhoy Z, Sheikh SS, Qureshi R, Memon J, Raza F, Kinshella MW, et al. Role of community engagement in maternal health in rural Pakistan: Findings from the CLIP randomized trial. J Glob Health. 2021;11:04045.
403. Herron EK, Powers K, Mullen L, Burkhart B. Effect of case study versus video simulation on nursing students' satisfaction, self-confidence, and knowledge: a quasi-experimental study. Nurse education today. 2019;79:129.
404. Herron EK, Powers K, Mullen L, Burkhart B. Effect of case study versus video simulation on nursing students' satisfaction, self-confidence, and knowledge: a quasi-experimental study. Nurse education today. 2019;79:129.
405. Henderson JT, Webber EM, Vesco KK, Thomas RG. U.S. Preventive Services Task Force Evidence Syntheses, formerly Systematic Evidence Reviews. Screening for Hypertensive Disorders of Pregnancy: An Evidence Update for the US Preventive Services Task Force. Rockville (MD): Agency for Healthcare Research and Quality (US); 2023.
406. Hellem AK, Whitfield C, Casetti A, Robles MC, Dinh M, Meurer W, et al. Engagement in Self-measured Blood Pressure Monitoring Among Medically Underresourced Participants (the Reach Out Trial): Digital Framework Qualitative Study. JMIR Cardio. 2023;7:e38900.
407. Hearn J, Ssinabulya I, Schwartz JI, Akiteng AR, Ross HJ, Cafazzo JA. Self-management of non-communicable diseases in low- and middle-income countries: A scoping review. PLoS One. 2019;14(7):e0219141.
408. Hark LA, Myers JS, Ines A, Jiang A, Rahmatnejad K, Zhan T, et

al. Philadelphia Telemedicine Glaucoma Detection and Follow-up Study: confirmation between eye screening and comprehensive eye examination diagnoses. Br J Ophthalmol. 2019;103(12):1820-6.
409. Hark LA, Katz LJ, Myers JS, Waisbourd M, Johnson D, Pizzi LT, et al. Philadelphia Telemedicine Glaucoma Detection and Follow-up Study: Methods and Screening Results. Am J Ophthalmol. 2017;181:114-24.
410. Haricharan HJ, Heap M, Hacking D, Lau YK. Health promotion via SMS improves hypertension knowledge for deaf South Africans. BMC Public Health. 2017;17(1):663.
411. Hanh L, Schiff N, du Plessis J, Hoang DB. A pervasive tele-health system for continual and low intrusive monitoring using peer-to-peer networks. International Journal of Computer Applications in Technology. 2009;34(4):330-4.
412. Hacking D, Lau YK, Haricharan HJ, Heap M. Comparison of two text message (mHealth) campaigns for the Deaf: Contracted out v. conducted in-house. S Afr Med J. 2015;106(1):47-9.
413. Hacking D, Haricharan HJ, Brittain K, Lau YK, Cassidy T, Heap M. Hypertension Health Promotion via Text Messaging at a Community Health Center in South Africa: A Mixed Methods Study. JMIR Mhealth Uhealth. 2016;4(1):e22.
414. Hacking D, Haricharan HJ, Brittain K, Lau YK, Cassidy T, Heap M. Hypertension Health Promotion via Text Messaging at a Community Health Center in South Africa: A Mixed Methods Study. JMIR mHealth and uHealth. 2016;4(1):e22.
415. Hacking D, Haricharan HJ, Brittain K, Lau YK, Cassidy T, Heap M. Hypertension Health Promotion via Text Messaging at a Community Health Center in South Africa: a Mixed Methods Study. JMIR mHealth and uHealth. 2016;4(1):e22.
416. Hacking D, Haricharan HJ, Brittain K, Lau YK, Cassidy T, Heap M. Hypertension Health Promotion via Text Messaging at a Community Health Center in South Africa: A Mixed Methods Study. JMIR mHealth and uHealth. 2016;4(1):e22.
417. Hacking D, Haricharan HJ, Brittain K, Lau YK, Cassidy T, Heap M. Hypertension Health Promotion via Text Messaging at a Community Health Center in South Africa: a Mixed Methods Study. JMIR mHealth and uHealth. 2016;4(1):e22.
418. Groemer G, Tlustos R, Haider O, Luger U, Hauth E, Frischauf N, editors. Preliminary results from a crewed Mars exploration simulation at the Rio Tinto analogue site. 62nd International Astronautical Congress 2011, IAC 2011, October 3, 2011 - October 7, 2011; 2011; Cape Town, South africa: International Astronautical Federation, IAF.
419. Greer DB, Abel WM. Exploring Feasibility of mHealth to Manage Hypertension in Rural Black Older Adults: A Convergent Parallel Mixed Method Study. Patient Prefer Adherence. 2022;16:2135-48.
420. Green BB, Anderson ML, Ralston JD, Catz S, Fishman PA, Cook AJ. Patient ability and willingness to participate in a web-based intervention to improve hypertension control. Journal of Medical Internet Research. 2011;13(1):e1 (8 pp.).
421. Goessens BM, Visseren FL, de Nooijer J, van den Borne HW,

Algra A, Wierdsma J, et al. A pilot-study to identify the feasibility of an Internet-based coaching programme for changing the vascular risk profile of high-risk patients. Patient Educ Couns. 2008;73(1):67-72.
422. Gaziano T, Abrahams-Gessel S, Surka S, Sy S, Pandya A, Denman CA, et al. Cardiovascular Disease Screening By Community Health Workers Can Be Cost-Effective In Low-Resource Countries. Health Affairs. 2015;34(9):1538-45,A1-A23.
423. Friedberg JP, Robinaugh DJ, Wang B, Allegrante JP, Lipsitz SR, Natarajan S. Who is being reached for a telephone-delivered intervention for patients with uncontrolled hypertension? Telemed J E Health. 2014;20(3):229-34.
424. Friedberg JP, Robinaugh DJ, Wang B, Allegrante JP, Lipsitz SR, Natarajan S. Who is being reached for a telephone-delivered intervention for patients with uncontrolled hypertension? Telemedicine and e-Health. 2014;20(3):229-34.
425. Foley P, Steinberg D, Levine E, Askew S, Batch BC, Puleo EM, et al. Track: A randomized controlled trial of a digital health obesity treatment intervention for medically vulnerable primary care patients. Contemp Clin Trials. 2016;48:12-20.
426. Fischer A, Chadyiwa M, Tshuma N, Nkosi V. Acceptability of Mobile Health Interventions to Increase Diabetic Risk Factor Awareness Among the Commuter Population in Johannesburg: Descriptive Cross-Sectional Study. JMIR Diabetes. 2019;4(3):e12600.
427. Fischer A, Chadyiwa M, Tshuma N, Nkosi V. Acceptability of Mobile Health Interventions to Increase Diabetic Risk Factor Awareness Among the Commuter Population in Johannesburg: Descriptive Cross-Sectional Study. JMIR diabetes. 2019;4(3):e12600.
428. Fischer A, Chadyiwa M, Tshuma N, Nkosi V. Acceptability of Mobile Health Interventions to Increase Diabetic Risk Factor Awareness Among the Commuter Population in Johannesburg: Descriptive Cross-Sectional Study. JMIR diabetes. 2019;4(3):e12600.
429. Finkelstein J, Cha E. Hypertension telemanagement in blacks. Circ Cardiovasc Qual Outcomes. 2009;2(3):272-8.
430. Festo C, Vannevel V, Ali H, Tamrat T, Mollel GJ, Hlongwane T, et al. Accuracy of a smartphone application for blood pressure estimation in Bangladesh, South Africa, and Tanzania. npj Digital Medicine. 2023;6(1).
431. Festo C, Vannevel V, Ali H, Tamrat T, Mollel GJ, Hlongwane T, et al. Accuracy of a smartphone application for blood pressure estimation in Bangladesh, South Africa, and Tanzania. npj Digital Medicine. 2023:69 (9 pp.).
432. Ferrell A. 25th IFSO World Congress Silver Anniversary. Obesity Surgery. 2022;32(Supplement 2):39-1044.
433. Ferrell A. 25th IFSO World Congress Silver Anniversary. Obesity Surgery. 2022;32(Supplement 2):39-1044.
434. Faleyimu BL, Ajayi PA, Ubuane LA, editors. Committing teleinformatics into delivering preventive medicare in an African oil and gas company. SPE International Conference on Health, Safety and Environment in Oil and Gas Exploration and Production, March 29, 2004 - March 31, 2004; 2004; Calgary, Alta., Canada: Society of Petroleum

Engineers (SPE).
435. Fajola A, Oduneye F, Ogbimi R, Mosuro O, Oyo-Ita A, Ovwigho U, editors. Taking goal zero outside the fence: Lifestyle and health influences on tanker and commercial drivers' performance and road safety. SPE International Conference and Exhibition on Health, Safety, Environment, and Sustainability 2020, HSE and Sustainability 2020, July 27, 2020 - July 31, 2020; 2020; Virtual, Online: Society of Petroleum Engineers.
436. Evans GF, Shirk A, Muturi P, Soliman EZ. Feasibility of Using Mobile ECG Recording Technology to Detect Atrial Fibrillation in Low-Resource Settings. Global Heart. 2017;12(4):285-9.
437. Evans GF, Shirk A, Muturi P, Soliman EZ. Feasibility of Using Mobile ECG Recording Technology to Detect Atrial Fibrillation in Low-Resource Settings. Global Heart. 2017;12(4):285-9.
438. Enslin D, Mallya P. Factors influencing treatment adherence in hypertension and HIV management in South Africa: A comparative literature review. South African Family Practice. 2022;64(1):a5434.
439. Enslin D, Mallya P. Factors influencing treatment adherence in hypertension and HIV management in South Africa: A comparative literature review. South African Family Practice. 2022;64(1):a5434.
440. Eaton C, Comer M, Pruette C, Psoter K, Riekert K. Text Messaging Adherence Intervention for Adolescents and Young Adults with Chronic Kidney Disease: Pilot Randomized Controlled Trial and Stakeholder Interviews. J Med Internet Res. 2020;22(8):e19861.
441. Earle KA, Istepanian RS, Zitouni K, Sungoor A, Tang B. Mobile telemonitoring for achieving tighter targets of blood pressure control in patients with complicated diabetes: a pilot study. Diabetes Technol Ther. 2010;12(7):575-9.
442. Douglas-Jones B, Mohr-Holland E, Mema N, Mathee S, Mathews G, Hurribance S, et al. A home-based care programme for rifampicin-resistant TB. International Journal of Tuberculosis and Lung Disease. 2021;25(7):587-9.
443. Douglas-Jones B, Mohr-Holland E, Mema N, Mathee S, Mathews G, Hurribance S, et al. A home-based care programme for rifampicin-resistant TB. International Journal of Tuberculosis and Lung Disease. 2021;25(7):587-9.
444. Dim S, Aliyu B, editors. Upstream Nigeria Telemedicine Pilot Program. SPE International Conference on Health, Safety and Environment 2014: The Journey Continues, HSE 2014, March 17, 2014 - March 19, 2014; 2014; Long Beach, CA, United states: Society of Petroleum Engineers.
445. Dim S, Aliyu B, editors. Upstream Nigeria Telemedicine Pilot Program. SPE International Conference on Health, Safety and Environment 2014: The Journey Continues, March 17, 2014 - March 19, 2014; 2014; Long Beach, CA, United states: Society of Petroleum Engineers.
446. Dim S, Aliyu B, editors. Upstream Nigeria telemedicine pilot program. SPE International Conference on Health, Safety and Environment 2014: The Journey Continues, March 17, 2014 - March 19, 2014; 2014; Long Beach, CA, United states: Society of Petroleum

Engineers.
447. Diby FK, Adoubi AK, Esse-Diby MCA, Gnaba AL, Zadi SZ, Ouattara AEP, et al. A Medico-Socio-Economic Analysis of TeleEKG in Ivory Coast. Telemedicine journal and e-health : the official journal of the American Telemedicine Association. 2021;27(3):286-95.
448. Diby FK, Adoubi AK, Esse-Diby MCA, Gnaba AL, Zadi SZ, Ouattara AEP, et al. A Medico-Socio-Economic Analysis of TeleEKG in Ivory Coast. Telemedicine journal and e-health : the official journal of the American Telemedicine Association. 2021;27(3):286-95.
449. Diby FK, Adoubi AK, Esse-Diby MA, Gnaba AL, Zadi SZ, Ouattara AEP, et al. A Medico-Socio-Economic Analysis of TeleEKG in Ivory Coast. Telemed J E Health. 2021;27(3):286-95.
450. Diby FK, Adoubi AK, Esse-Diby M-CA, Gnaba AL, Zadi SZ, Ouattara AEP, et al. A Medico-Socio-Economic Analysis of TeleEKG in Ivory Coast. Telemedicine journal and e-health : the official journal of the American Telemedicine Association. 2021;27(3):286-95.
451. Diby FK, Adoubi AK, Esse-Diby M-CA, Gnaba AL, Zadi SZ, Ouattara AEP, et al. A Medico-Socio-Economic Analysis of TeleEKG in Ivory Coast. Telemedicine journal and e-health : the official journal of the American Telemedicine Association. 2021;27(3):286-95.
452. Diby FK, Adoubi AK, Esse-Diby M-CA, Gnaba AL, Zadi SZ, Ouattara AEP, et al. A Medico-Socio-Economic Analysis of TeleEKG in Ivory Coast. Telemedicine journal and e-health : the official journal of the American Telemedicine Association. 2021;27(3):286-95.
453. Diby FK, Adoubi AK, Esse-Diby M-CA, Gnaba AL, Zadi SZ, Ouattara AEP, et al. A Medico-Socio-Economic Analysis of TeleEKG in Ivory Coast. Telemedicine and e-Health. 2021;27(3):286-95.
454. Dese K, Ayana G, Lamesgin simegn G. Low cost, non-invasive, and continuous vital signs monitoring device for pregnant women in low resource settings (Lvital device). HardwareX. 2022:e00276.
455. Dele-Ojo BF, Oseni TIA, Duodu F, Echieh CP, Blankson PK, Alabi BS, et al. The effect of mobile health technology on blood pressure control among patients with hypertension in Ghana and Nigeria. Res Sq. 2023.
456. Dele-Ojo BF, Oseni TIA, Duodu F, Echieh CP, Blankson P-K, Alabi BS, et al. The effect of mobile health technology on blood pressure control among patients with hypertension in Ghana and Nigeria. Research square. 2023.
457. Dele-Ojo BF, Oseni TIA, Duodu F, Echieh CP, Blankson P-K, Alabi BS, et al. The effect of mobile health technology on blood pressure control among patients with hypertension in Ghana and Nigeria. Research square. 2023.
458. DeLaughter KL, Fix GM, McDannold SE, Pope C, Bokhour BG, Shimada SL, et al. Incorporating African American Veterans' Success Stories for Hypertension Management: Developing a Behavioral Support Texting Protocol. JMIR Res Protoc. 2021;10(12):e29423.
459. de Jongh T, Gurol-Urganci I, Vodopivec-Jamsek V, Car J, Atun R. Mobile phone messaging for facilitating self-management of long-term illnesses. Cochrane Database Syst Rev. 2012;12(12):Cd007459.
460. Davidson TM, McGillicuddy J, Mueller M, Brunner-Jackson B,

Favella A, Anderson A, et al. Evaluation of an mHealth Medication Regimen Self-Management Program for African American and Hispanic Uncontrolled Hypertensives. J Pers Med. 2015;5(4):389-405.
461. Dalal S, Holmes MD, Laurence C, Bajunirwe F, Guwatudde D, Njelekela M, et al. Feasibility of a large cohort study in sub-Saharan Africa assessed through a four-country study. Glob Health Action. 2015;8:27422.
462. Dalal S, Holmes MD, Laurence C, Bajunirwe F, Guwatudde D, Njelekela M, et al. Feasibility of a large cohort study in sub-Saharan Africa assessed through a four-country study. Global Health Action. 2015;8:27422.
463. Dalal S, Holmes MD, Laurence C, Bajunirwe F, Guwatudde D, Njelekela M, et al. Feasibility of a large cohort study in sub-Saharan Africa assessed through a four-country study. Global health action. 2015;8:27422.
464. Dalal S, Holmes MD, Laurence C, Bajunirwe F, Guwatudde D, Njelekela M, et al. Feasibility of a large cohort study in sub-Saharan Africa assessed through a four-country study. Global Health Action. 2015;8:27422.
465. Dalal S, Holmes MD, Laurence C, Bajunirwe F, Guwatudde D, Njelekela M, et al. Feasibility of a large cohort study in sub-Saharan Africa assessed through a four-country study. Global health action. 2015;8:27422.
466. Dalal S, Holmes MD, Laurence C, Bajunirwe F, Guwatudde D, Njelekela M, et al. Feasibility of a large cohort study in sub-Saharan Africa assessed through a four-country study. Global health action. 2015;8:27422.
467. Dalal S, Holmes MD, Laurence C, Bajunirwe F, Guwatudde D, Njelekela M, et al. Feasibility of a large cohort study in sub-Saharan Africa assessed through a four-country study. Global health action. 2015;8:27422.
468. Dalal S, Holmes MD, Laurence C, Bajunirwe F, Guwatudde D, Njelekela M, et al. Feasibility of a large cohort study in sub-Saharan Africa assessed through a four-country study. Global health action. 2015;8:27422.
469. Dadelszen Pv, Bhutta ZA, Sharma S, Bone J, Singer J, Wong H, et al. The Community-Level Interventions for Pre-eclampsia (CLIP) cluster randomised trials in Mozambique, Pakistan, and India: an individual participant-level meta-analysis. Lancet (British edition). 2020;396(10250):553-63.
470. Dadelszen Pv, Bhutta ZA, Sharma S, Bone J, Singer J, Wong H, et al. The Community-Level Interventions for Pre-eclampsia (CLIP) cluster randomised trials in Mozambique, Pakistan, and India: an individual participant-level meta-analysis. Lancet (British edition). 2020;396(10250):553-63.
471. Cremers AL, Alege A, Nelissen HE, Okwor TJ, Osibogun A, Gerrets R, et al. Patients' and healthcare providers' perceptions and practices regarding hypertension, pharmacy-based care, and mHealth in Lagos, Nigeria: a mixed methods study. J Hypertens. 2019;37(2):389-97.
472. Cremers AL, Alege A, Nelissen HE, Okwor TJ, Osibogun A,

Gerrets R, et al. Patients' and healthcare providers' perceptions and practices regarding hypertension, pharmacy-based care, and mHealth in Lagos, Nigeria: a mixed methods study. Journal of hypertension. 2019;37(2):389-97.
473. Cremers AL, Alege A, Nelissen HE, Okwor TJ, Osibogun A, Gerrets R, et al. Patients' and healthcare providers' perceptions and practices regarding hypertension, pharmacy-based care, and mHealth in Lagos, Nigeria: a mixed methods study. Journal of hypertension. 2019;37(2):389-97.
474. Cremers AL, Alege A, Nelissen HE, Okwor TJ, Osibogun A, Gerrets R, et al. Patients' and healthcare providers' perceptions and practices regarding hypertension, pharmacy-based care, and mHealth in Lagos, Nigeria: a mixed methods study. Journal of hypertension. 2019;37(2):389-97.
475. Cremers AL, Alege A, Nelissen HE, Okwor TJ, Osibogun A, Gerrets R, et al. Patients' and healthcare providers' perceptions and practices regarding hypertension, pharmacy-based care, and mHealth in Lagos, Nigeria: A mixed methods study. Journal of Hypertension. 2019;37(2):389-97.
476. Cremers AL, Alege A, Nelissen HE, Okwor TJ, Osibogun A, Gerrets R, et al. Patients' and healthcare providers' perceptions and practices regarding hypertension, pharmacy-based care, and mHealth in Lagos, Nigeria: A mixed methods study. Journal of Hypertension. 2019;37(2):389-97.
477. Cremers AL, Alege A, Nelissen HE, Okwor TJ, Osibogun A, Gerrets R, et al. Patients' and healthcare providers' perceptions and practices regarding hypertension, pharmacy-based care, and mHealth in Lagos, Nigeria: a mixed methods study. Journal of Hypertension. 2019;37(2):389-97.
478. Cremers AL, Alege A, Nelissen HE, Okwor TJ, Osibogun A, Gerrets R, et al. Patients' and healthcare providers' perceptions and practices regarding hypertension, pharmacy-based care, and mHealth in Lagos, Nigeria: a mixed methods study. Journal of Hypertension. 2019;37(2):389-97.
479. Cremers A, Alege A, Nelissen H, Okwor T, Osibogun A, Gerrets R, et al. Patients' and healthcare providers' perceptions and practices regarding hypertension, pharmacy-based care, and mHealth in Lagos, Nigeria: a mixed methods study. J Hypertens. 2019;37(2):389-97.
480. Cremers A, Alege A, Nelissen H, Okwor T, Osibogun A, Gerrets R, et al. Patients' and healthcare providers' perceptions and practices regarding hypertension, pharmacy-based care, and mHealth in Lagos, Nigeria: a mixed methods study. J Hypertens. 2019;37(2):389-97.
481. Coombs P, Nicol C, Shimba J, Rweyemamu S, Mutayabalwa D, Peck R, et al. Implementing a telemedicine screening program for ophthalmic disease at bugando medical center in Mwanza, Tanzania. Investigative Ophthalmology and Visual Science. 2016;57(12):1581.
482. Coombs P, Nicol C, Shimba J, Rweyemamu S, Mutayabalwa D, Peck R, et al. Implementing a telemedicine screening program for ophthalmic disease at bugando medical center in Mwanza, Tanzania. Investigative Ophthalmology and Visual Science. 2016;57(12):1581.

483. Commodore-Mensah Y, Sarfo FS, Turkson-Ocran RA, Foti K, Mobula LM, Himmelfarb CD, et al. Addressing Hypertension Care in Africa (ADHINCRA): Study protocol for a cluster-randomized controlled pilot trial. Contemp Clin Trials. 2023;125:107077.
484. Commodore-Mensah Y, Sarfo FS, Turkson-Ocran RA, Foti K, Mobula LM, Himmelfarb CD, et al. Addressing Hypertension Care in Africa (ADHINCRA): Study protocol for a cluster-randomized controlled pilot trial. Contemporary Clinical Trials. 2023;125:107077.
485. Commodore-Mensah Y, Sarfo FS, Turkson-Ocran RA, Foti K, Mobula LM, Himmelfarb CD, et al. Addressing Hypertension Care in Africa (ADHINCRA): Study protocol for a cluster-randomized controlled pilot trial. Contemporary Clinical Trials. 2023;125:107077.
486. Commodore-Mensah Y, Sarfo FS, Turkson-Ocran R-A, Foti K, Mobula LM, Himmelfarb CD, et al. Addressing Hypertension Care in Africa (ADHINCRA): Study protocol for a cluster-randomized controlled pilot trial. Contemporary clinical trials. 2023;125:107077.
487. Commodore-Mensah Y, Sarfo FS, Turkson-Ocran R-A, Foti K, Mobula LM, Himmelfarb CD, et al. Addressing Hypertension Care in Africa (ADHINCRA): Study protocol for a cluster-randomized controlled pilot trial. Contemporary clinical trials. 2023;125:107077.
488. Commodore-Mensah Y, Sarfo FS, Turkson-Ocran R-A, Foti K, Mobula LM, Himmelfarb CD, et al. Addressing Hypertension Care in Africa (ADHINCRA): Study protocol for a cluster-randomized controlled pilot trial. Contemporary clinical trials. 2023;125:107077.
489. Commodore-Mensah Y, Sarfo FS, Ruth-Alma TO, Foti K, Mobula LM, Himmelfarb CD, et al. Addressing Hypertension Care in Africa (ADHINCRA): Study protocol for a cluster-randomized controlled pilot trial. Contemporary clinical trials. 2022:107077.
490. Commodore-Mensah Y, Sarfo FS, Ruth-Alma TO, Foti K, Mobula LM, Himmelfarb CD, et al. Addressing Hypertension Care in Africa (ADHINCRA): study protocol for a cluster-randomized controlled pilot trial. Contemporary clinical trials. 2022:107077.
491. Commodore-Mensah Y, Sarfo FS, Ruth-Alma TO, Foti K, Mobula LM, Himmelfarb CD, et al. Addressing Hypertension Care in Africa (ADHINCRA): Study protocol for a cluster-randomized controlled pilot trial. Contemporary clinical trials. 2022:107077.
492. Commodore-Mensah Y, Sarfo FS, Ruth-Alma TO, Foti K, Mobula LM, Himmelfarb CD, et al. Addressing Hypertension Care in Africa (ADHINCRA): study protocol for a cluster-randomized controlled pilot trial. Contemporary clinical trials. 2022:107077.
493. Coleman A. Medication adherence of elderly citizens in retirement homes through a mobile phone adherence monitoring framework (Mpamf) for developing countries: A case study in South Africa. Indian Journal of Pharmaceutical Education and Research. 2014;48(3):6-11.
494. Coleman A. Medication adherence of elderly citizens in retirement homes through a mobile phone adherence monitoring framework (Mpamf) for developing countries: A case study in South Africa. Indian Journal of Pharmaceutical Education and Research. 2014;48(3):6-11.
495. Clarke A, El Moudden I, Wood L, Newby O, Dodani S. Effectiveness of a Telehealth-Based Behavioral Lifestyle Program for

Hypertension Control in African Americans: Results From Heals Med-Tech Randomized Controlled Study. CIRCULATION. 2022;146.
496. Chori BS, Abubakar SM, Ibrahim EA, Ajanya O, Ijir A, Maduka DC, et al. FEASIBILITY OF HOME BLOOD PRESSURE TELEMONITORING IN A LOW RESOURCE SETTING. Blood Pressure Monitoring. 2022;27(Supplement 1):e1.
497. Chori BS, Abubakar SM, Ibrahim EA, Ajanya O, Ijir A, Maduka DC, et al. FEASIBILITY OF HOME BLOOD PRESSURE TELEMONITORING IN A LOW RESOURCE SETTING. Blood pressure monitoring. 2022;27:e1.
498. Chori BS, Abubakar SM, Ibrahim EA, Ajanya O, Ijir A, Maduka DC, et al. FEASIBILITY OF HOME BLOOD PRESSURE TELEMONITORING IN A LOW RESOURCE SETTING. Blood Pressure Monitoring. 2022;27(Supplement 1):e1.
499. Chori BS, Abubakar SM, Ibrahim EA, Ajanya O, Ijir A, Maduka DC, et al. FEASIBILITY OF HOME BLOOD PRESSURE TELEMONITORING IN A LOW RESOURCE SETTING. Blood pressure monitoring. 2022;27:e1.
500. Chapman RR, Mohamed SB, Rage H, Abdulahi A, Jimenez J, Gavin AR, et al. Preventing Health Disparities during COVID through Perinatal Home Screening as Black Authoritative Knowledge. J Racial Ethn Health Disparities. 2023:1-15.
501. Chang W, Chamie G, Mwai D, Clark TD, Thirumurthy H, Charlebois ED, et al. Implementation and Operational Research: Cost and Efficiency of a Hybrid Mobile Multidisease Testing Approach With High HIV Testing Coverage in East Africa. J Acquir Immune Defic Syndr. 2016;73(3):e39-e45.
502. Chang W, Chamie G, Mwai D, Clark TD, Thirumurthy H, Charlebois ED, et al. Implementation and Operational Research: Cost and Efficiency of a Hybrid Mobile Multidisease Testing Approach With High HIV Testing Coverage in East Africa. Journal of acquired immune deficiency syndromes (1999). 2016;73(3):e39-e45.
503. Chang W, Chamie G, Mwai D, Clark TD, Thirumurthy H, Charlebois ED, et al. Implementation and Operational Research: Cost and Efficiency of a Hybrid Mobile Multidisease Testing Approach With High HIV Testing Coverage in East Africa. Journal of acquired immune deficiency syndromes (1999). 2016;73(3):e39-e45.
504. Chang W, Chamie G, Mwai D, Clark TD, Thirumurthy H, Charlebois ED, et al. Implementation and Operational Research: Cost and Efficiency of a Hybrid Mobile Multidisease Testing Approach With High HIV Testing Coverage in East Africa. Journal of acquired immune deficiency syndromes (1999). 2016;73(3):e39-e45.
505. Chandler J, Sox L, Kellam K, Feder L, Nemeth L, Treiber F. Impact of a Culturally Tailored mHealth Medication Regimen Self-Management Program upon Blood Pressure among Hypertensive Hispanic Adults. Int J Environ Res Public Health. 2019;16(7).
506. Champoux E, Price R, Cowdery JE, Dinh M, Meurer WJ, Rehman N, et al. Reach Out Emergency Department: Partnering With an Economically Disadvantaged Community in the Development of a Text-Messaging Intervention to Address High Blood Pressure. Health Promot Pract. 2020;21(5):791-801.
507. Cardozo L, Steinberg J. Telemedicine for recently discharged older patients. Telemed J E Health. 2010;16(1):49-55.
508. Cardozo L, Steinberg J. Telemedicine for recently discharged

older patients. Telemedicine and e-Health. 2010;16(1):49-55.
509. Buis LR, Dawood K, Kadri R, Dawood R, Richardson CR, Djuric Z, et al. Improving Blood Pressure Among African Americans With Hypertension Using a Mobile Health Approach (the MI-BP App): Protocol for a Randomized Controlled Trial. JMIR Res Protoc. 2019;8(1):e12601.
510. Buis LR, Artinian NT, Schwiebert L, Yarandi H, Levy PD. Text Messaging to Improve Hypertension Medication Adherence in African Americans: BPMED Intervention Development and Study Protocol. JMIR Res Protoc. 2015;4(1):e1.
511. Buis L, Hirzel L, Dawood RM, Dawood KL, Nichols LP, Artinian NT, et al. Text Messaging to Improve Hypertension Medication Adherence in African Americans From Primary Care and Emergency Department Settings: Results From Two Randomized Feasibility Studies. JMIR Mhealth Uhealth. 2017;5(2):e9.
512. Brewer LC, Jones C, Slusser JP, Pasha M, Lalika M, Chacon M, et al. mHealth Intervention for Promoting Hypertension Self-management Among African American Patients Receiving Care at a Community Health Center: Formative Evaluation of the FAITH! Hypertension App. JMIR FORMATIVE RESEARCH. 2023;7.
513. Brewer LC, Jones C, Slusser JP, Pasha M, Lalika M, Chacon M, et al. mHealth Intervention for Promoting Hypertension Self-management Among African American Patients Receiving Care at a Community Health Center: Formative Evaluation of the FAITH! Hypertension App. JMIR Form Res. 2023;7:e45061.
514. Brewer LC, Jenkins S, Hayes SN, Kumbamu A, Jones C, Burke LE, et al. Community-based, cluster-randomized pilot trial of a cardiovascular mHealth intervention: Rationale, design, and baseline findings of the FAITH! Trial. Am Heart J. 2022;247:1-14.
515. Brennan T, Spettell C, Villagra V, Ofili E, McMahill-Walraven C, Lowy EJ, et al. Disease management to promote blood pressure control among African Americans. Popul Health Manag. 2010;13(2):65-72.
516. Bowser DM, Shepard DS, Nandakumar A, Okunogbe A, Morrill T, Halasa-Rappel Y, et al. Cost effectiveness of mobile health for antenatal care and facility births in Nigeria. Annals of Global Health. 2018;84(4):592-602.
517. Bowser DM, Shepard DS, Nandakumar A, Okunogbe A, Morrill T, Halasa-Rappel Y, et al. Cost effectiveness of mobile health for antenatal care and facility births in Nigeria. Annals of Global Health. 2018;84(4):592-602.
518. Bosworth HB, Powers BJ, Olsen MK, McCant F, Grubber J, Smith V, et al. Home blood pressure management and improved blood pressure control: results from a randomized controlled trial. Arch Intern Med. 2011;171(13):1173-80.
519. Bosworth HB, Olsen MK, McCant F, Stechuchak KM, Danus S, Crowley MJ, et al. Telemedicine cardiovascular risk reduction in veterans: The CITIES trial. Am Heart J. 2018;199:122-9.
520. Bone JN, Khowaja AR, Vidler M, Payne BA, Bellad MB, Goudar SS, et al. Economic and cost-effectiveness analysis of the Community-Level Interventions for Pre-eclampsia (CLIP) trials in India, Pakistan and Mozambique. BMJ global health. 2021;6(5).

521. Bone JN, Khowaja AR, Vidler M, Payne BA, Bellad MB, Goudar SS, et al. Economic and cost-effectiveness analysis of the Community-Level Interventions for Pre-eclampsia (CLIP) trials in India, Pakistan and Mozambique. BMJ Glob Health. 2021;6(5).
522. Bone JN, Khowaja AR, Vidler M, Payne BA, Bellad MB, Goudar SS, et al. Economic and cost-effectiveness analysis of the Community-Level Interventions for Pre-eclampsia (CLIP) trials in India, Pakistan and Mozambique. BMJ global health. 2021;6(5).
523. Bone JN, Khowaja AR, Vidler M, Payne BA, Bellad MB, Goudar SS, et al. Economic and cost-effectiveness analysis of the Community-Level Interventions for Pre-eclampsia (CLIP) trials in India, Pakistan and Mozambique. BMJ Global Health. 2021;6(5):e004123.
524. Bone JN, Khowaja AR, Vidler M, Payne BA, Bellad MB, Goudar SS, et al. Economic and cost-effectiveness analysis of the Community-Level Interventions for Pre-eclampsia (CLIP) trials in India, Pakistan and Mozambique. BMJ global health. 2021;6(5).
525. Bone JN, Khowaja AR, Vidler M, Payne BA, Bellad MB, Goudar SS, et al. Economic and cost-effectiveness analysis of the Community-Level Interventions for Pre-eclampsia (CLIP) trials in India, Pakistan and Mozambique. BMJ global health. 2021;6(5).
526. Bone JN, Khowaja AR, Vidler M, Payne BA, Bellad MB, Goudar SS, et al. Economic and cost-effectiveness analysis of the Community-Level Interventions for Pre-eclampsia (CLIP) trials in India, Pakistan and Mozambique. BMJ Global Health. 2021;6(5):e004123.
527. Bone JN, Khowaja AR, Vidler M, Payne BA, Bellad MB, Goudar SS, et al. Economic and cost-effectiveness analysis of the Community-Level Interventions for Pre-eclampsia (CLIP) trials in India, Pakistan and Mozambique. BMJ global health. 2021;6(5).
528. Bone JN, Khowaja AR, Vidler M, Payne BA, Bellad MB, Goudar SS, et al. Economic and cost-effectiveness analysis of the community-level interventions for pre-eclampsia (CLIP) trials in India, Pakistan and Mozambique. BMJ Global Health. 2021;6(5).
529. Bone JN, Khowaja AR, Vidler M, Payne BA, Bellad MB, Goudar SS, et al. Economic and cost-effectiveness analysis of the community-level interventions for pre-eclampsia (CLIP) trials in India, Pakistan and Mozambique. BMJ Global Health. 2021;6(5).
530. Bondmass M, Bolger N, Castro G, Avitall B. The effect of home monitoring and telemanagement on blood pressure control among African Americans. Telemedicine Journal. 2000;6(1):15-23.
531. Bobrow K, Farmer AJ, Springer D, Shanyinde M, Yu LM, Brennan T, et al. Mobile Phone Text Messages to Support Treatment Adherence in Adults With High Blood Pressure (SMS-Text Adherence Support [StAR]): A Single-Blind, Randomized Trial. Circulation. 2016;133(6):592-600.
532. Bobrow K, Farmer AJ, Springer D, Shanyinde M, Yu LM, Brennan T, et al. Mobile Phone Text Messages to Support Treatment Adherence in Adults with High Blood Pressure (SMS-Text Adherence Support [StAR]): A Single-Blind, Randomized Trial. Circulation. 2016;133(6):592-600.
533. Bobrow K, Farmer AJ, Springer D, Shanyinde M, Yu LM, Brennan T, et al. Mobile Phone Text Messages to Support Treatment Adherence in Adults With High Blood Pressure (SMS-Text Adherence Support [StAR]): a

Single-Blind, Randomized Trial. Circulation. 2016;133(6):592.
534. Bobrow K, Farmer AJ, Springer D, Shanyinde M, Yu LM, Brennan T, et al. Mobile Phone Text Messages to Support Treatment Adherence in Adults with High Blood Pressure (SMS-Text Adherence Support [StAR]): A Single-Blind, Randomized Trial. Circulation. 2016;133(6):592-600.
535. Bobrow K, Farmer AJ, Springer D, Shanyinde M, Yu LM, Brennan T, et al. Mobile Phone Text Messages to Support Treatment Adherence in Adults With High Blood Pressure (SMS-Text Adherence Support [StAR]): a Single-Blind, Randomized Trial. Circulation. 2016;133(6):592.
536. Bobrow K, Farmer AJ, Springer D, Shanyinde M, Yu L-M, Brennan T, et al. Mobile Phone Text Messages to Support Treatment Adherence in Adults With High Blood Pressure (SMS-Text Adherence Support [StAR]): A Single-Blind, Randomized Trial. Circulation. 2016;133(6):592-600.
537. Bobrow K, Farmer AJ, Springer D, Shanyinde M, Yu L-M, Brennan T, et al. Mobile Phone Text Messages to Support Treatment Adherence in Adults With High Blood Pressure (SMS-Text Adherence Support [StAR]): A Single-Blind, Randomized Trial. Circulation. 2016;133(6):592-600.
538. Bobrow K, Farmer AJ, Springer D, Shanyinde M, Yu L-M, Brennan T, et al. Mobile Phone Text Messages to Support Treatment Adherence in Adults With High Blood Pressure (SMS-Text Adherence Support [StAR]): A Single-Blind, Randomized Trial. Circulation. 2016;133(6):592-600.
539. Bobrow K, Farmer A, Springer D, Shanyinde M, Yu L-M, Brennan T, et al. Mobile Phone Text Messages to Support Treatment Adherence in Adults With High Blood Pressure (SMS-Text Adherence Support [StAR]): A Single-Blind, Randomized Trial. Circulation. 2016;133(6):592-600.
540. Bobrow K, Farmer A, Springer D, Shanyinde M, Yu L-M, Brennan T, et al. Mobile Phone Text Messages to Support Treatment Adherence in Adults With High Blood Pressure (SMS-Text Adherence Support [StAR]): A Single-Blind, Randomized Trial. Circulation. 2016;133(6):592-600.
541. Bobrow K, Farmer A, Cishe N, Nwagi N, Namane M, Brennan TP, et al. Using the Medical Research Council framework for development and evaluation of complex interventions in a low resource setting to develop a theory-based treatment support intervention delivered via SMS text message to improve blood pressure control. BMC Health Serv Res. 2018;18(1):33.
542. Bobrow K, Farmer A, Cishe N, Nwagi N, Namane M, Brennan TP, et al. Using the Medical Research Council framework for development and evaluation of complex interventions in a low resource setting to develop a theory-based treatment support intervention delivered via SMS text message to improve blood pressure control. BMC health services research. 2018;18(1):33.
543. Bobrow K, Farmer A, Cishe N, Nwagi N, Namane M, Brennan TP, et al. Using the Medical Research Council framework for development and evaluation of complex interventions in a low resource setting to develop a theory-based treatment support intervention delivered via SMS text message to improve blood pressure control. BMC health services research. 2018;18(1):33.
544. Bobrow K, Farmer A, Cishe N, Nwagi N, Namane M, Brennan TP, et al. Using the Medical Research Council framework for development and evaluation of complex interventions in a low resource setting to

develop a theory-based treatment support intervention delivered via SMS text message to improve blood pressure control. BMC health services research. 2018;18(1):33.
545. Bobrow K, Farmer A, Cishe N, Nwagi N, Namane M, Brennan TP, et al. Using the Medical Research Council framework for development and evaluation of complex interventions in a low resource setting to develop a theory-based treatment support intervention delivered via SMS text message to improve blood pressure control. BMC health services research. 2018;18(1):33.
546. Bobrow K, Farmer A, Cishe N, Nwagi N, Namane M, Brennan TP, et al. Using the Medical Research Council framework for development and evaluation of complex interventions in a low resource setting to develop a theory-based treatment support intervention delivered via SMS text message to improve blood pressure control. BMC health services research. 2018;18(1):33.
547. Bobrow K, Brennan T, Springer D, Levitt NS, Rayner B, Namane M, et al. Efficacy of a text messaging (SMS) based intervention for adults with hypertension: protocol for the StAR (SMS Text-message Adherence suppoRt trial) randomised controlled trial. BMC Public Health. 2014;14:28.
548. Bobrow K, Brennan T, Springer D, Levitt NS, Rayner B, Namane M, et al. Efficacy of a text messaging (SMS) based intervention for adults with hypertension: protocol for the StAR (SMS Text-message Adherence suppoRt trial) randomised controlled trial. BMC Public Health. 2014;14(28).
549. Bobrow K, Brennan T, Springer D, Levitt NS, Rayner B, Namane M, et al. Efficacy of a text messaging (SMS) based intervention for adults with hypertension: protocol for the StAR (SMS Text-message Adherence suppoRt trial) randomised controlled trial. BMC public health. 2014;14:28.
550. Bobrow K, Brennan T, Springer D, Levitt NS, Rayner B, Namane M, et al. Efficacy of a text messaging (SMS) based intervention for adults with hypertension: protocol for the StAR (SMS Text-message Adherence suppoRt trial) randomised controlled trial. BMC public health. 2014;14:28.
551. Bobrow K, Brennan T, Springer D, Levitt NS, Rayner B, Namane M, et al. Efficacy of a text messaging (SMS) based intervention for adults with hypertension: protocol for the StAR (SMS Text-message Adherence suppoRt trial) randomised controlled trial. BMC Public Health. 2014;14(28).
552. Bobrow K, Brennan T, Springer D, Levitt NS, Rayner B, Namane M, et al. Efficacy of a text messaging (SMS) based intervention for adults with hypertension: protocol for the StAR (SMS Text-message Adherence suppoRt trial) randomised controlled trial. BMC public health. 2014;14:28.
553. Bobrow K, Brennan T, Springer D, Levitt NS, Rayner B, Namane M, et al. Efficacy of a text messaging (SMS) based intervention for adults with hypertension: protocol for the StAR (SMS Text-message Adherence suppoRt trial) randomised controlled trial. BMC public health. 2014;14:28.

554. Bobrow K, Brennan T, Springer D, Levitt NS, Rayner B, Namane M, et al. Efficacy of a text messaging (SMS) based intervention for adults with hypertension: protocol for the StAR (SMS Text-message Adherence suppoRt trial) randomised controlled trial. BMC public health. 2014;14:28.
555. Bobrow K, Brennan T, Springer D, Levitt NS, Rayner B, Namane M, et al. Efficacy of a text messaging (SMS) based intervention for adults with hypertension: protocol for the StAR (SMS Text-message Adherence suppoRt trial) randomised controlled trial. BMC public health. 2014;14:28.
556. Bobrow K, Brennan T, Springer D, Levitt NS, Rayner B, Namane M, et al. Efficacy of a text messaging (SMS) based intervention for adults with hypertension: protocol for the StAR (SMS Text-message Adherence suppoRt trial) randomised controlled trial. BMC public health. 2014;14:28.
557. Boatin AA, Ngonzi J, Wylie BJ, Lugobe HM, Bebell LM, Mugyenyi G, et al. Wireless versus routine physiologic monitoring after cesarean delivery to reduce maternal morbidity and mortality in a resource-limited setting: protocol of type 2 hybrid effectiveness-implementation study. BMC Pregnancy and Childbirth. 2021;21(1):124.
558. Boatin AA, Ngonzi J, Wylie BJ, Lugobe HM, Bebell LM, Mugyenyi G, et al. Wireless versus routine physiologic monitoring after cesarean delivery to reduce maternal morbidity and mortality in a resource-limited setting: protocol of type 2 hybrid effectiveness-implementation study. BMC Pregnancy and Childbirth. 2021;21(1):124.
559. Blyler CA, Ebinger J, Rashid M, Moy NP, Cheng S, Albert CM, et al. Improving Efficiency of the Barbershop Model of Hypertension Care for Black Men With Virtual Visits. J Am Heart Assoc. 2021;10(13):e020796.
560. Blanckenberg M, Worst C, Scheffer C, editors. Development of a mobile phone based ophthalmoscope for telemedicine. 2011 33rd Annual International Conference of the IEEE Engineering in Medicine and Biology Society, 30 Aug-3 Sept 2011; 2011; Piscataway, NJ, USA: IEEE.
561. Blanckenberg M, Worst C, Scheffer C, editors. Development of a mobile phone based ophthalmoscope for telemedicine. 33rd Annual International Conference of the IEEE Engineering in Medicine and Biology Society, EMBS 2011, August 30, 2011 - September 3, 2011; 2011; Boston, MA, United states: Institute of Electrical and Electronics Engineers Inc.
562. Bertoncello C, Cocchio S, Fonzo M, Bennici SE, Russo F, Putoto G. The potential of mobile health clinics in chronic disease prevention and health promotion in universal healthcare systems. An on-field experiment. Int J Equity Health. 2020;19(1):59.
563. Bernardes ML, Rosendo-Silva B, Rosendo I, Monteiro-Soares M. Web-based interventions to improve blood pressure control in patients with hypertension: a protocol for a systematic review. BMJ Open. 2023;13(8):e067610.
564. Benard R, Chipungahelo MS. Accessibility of women to health information in Tanzania. A case study of Morogoro. Library Review. 2017;66(6-7):415-29.

565. Barsky J, Hunter R, McAllister C, Yeates K, Campbell N, Liu P, et al. Analysis of the Implementation, User Perspectives, and Feedback From a Mobile Health Intervention for Individuals Living With Hypertension (DREAM-GLOBAL): Mixed Methods Study. JMIR Mhealth Uhealth. 2019;7(12):e12639.
566. Barsky J, Hunter R, McAllister C, Yeates K, Campbell N, Liu P, et al. Analysis of the Implementation, User Perspectives, and Feedback From a Mobile Health Intervention for Individuals Living With Hypertension (DREAM-GLOBAL): Mixed Methods Study. JMIR mHealth and uHealth. 2019;7(12):e12639.
567. Barsky J, Hunter R, McAllister C, Yeates K, Campbell N, Liu P, et al. Analysis of the Implementation, User Perspectives, and Feedback From a Mobile Health Intervention for Individuals Living With Hypertension (DREAM-GLOBAL): Mixed Methods Study. JMIR mHealth and uHealth. 2019;7(12):e12639.
568. Barsky J, Hunter R, McAllister C, Yeates K, Campbell N, Liu P, et al. Analysis of the Implementation, User Perspectives, and Feedback From a Mobile Health Intervention for Individuals Living With Hypertension (DREAM-GLOBAL): Mixed Methods Study. JMIR mHealth and uHealth. 2019;7(12):e12639.
569. Barsky J, Hunter R, McAllister C, Yeates K, Campbell N, Liu P, et al. Analysis of the Implementation, User Perspectives, and Feedback From a Mobile Health Intervention for Individuals Living With Hypertension (DREAM-GLOBAL): mixed Methods Study. JMIR mHealth and uHealth. 2019;7(12):e12639.
570. Barsky J, Hunter R, McAllister C, Yeates K, Campbell N, Liu P, et al. Analysis of the Implementation, User Perspectives, and Feedback From a Mobile Health Intervention for Individuals Living With Hypertension (DREAM-GLOBAL): Mixed Methods Study. JMIR mHealth and uHealth. 2019;7(12):e12639.
571. Barsky J, Hunter R, McAllister C, Yeates K, Campbell N, Liu P, et al. Analysis of the Implementation, User Perspectives, and Feedback From a Mobile Health Intervention for Individuals Living With Hypertension (DREAM-GLOBAL): Mixed Methods Study. JMIR mHealth and uHealth. 2019;7(12):e12639.
572. Barsky J, Hunter R, McAllister C, Yeates K, Campbell N, Liu P, et al. Analysis of the Implementation, User Perspectives, and Feedback From a Mobile Health Intervention for Individuals Living With Hypertension (DREAM-GLOBAL): mixed Methods Study. JMIR mHealth and uHealth. 2019;7(12):e12639.
573. Baatiema L, Sanuade OA, Allen LN, Abimbola S, Hategeka C, Koram KA, et al. Health system adaptions to improve care for people living with non-communicable diseases during COVID-19 in low-middle income countries: A scoping review. J Glob Health. 2023;13:06006.
574. Aycock DM, Kirkendoll KD, Coleman KC, Clark PC, Albright KC, Alexandrov AW. Family history of stroke among African Americans and its association with risk factors, knowledge, perceptions, and exercise. J Cardiovasc Nurs. 2015;30(2):E1-6.
575. Aw M, Ochieng BO, Attambo D, Opot D, Aw J, Francis S, et al. Critical appraisal of a mHealth-assisted community-based

cardiovascular disease risk screening program in rural Kenya: an operational research study. Pathog Glob Health. 2020;114(7):379-87.
576. Aw M, Ochieng BO, Attambo D, Opot D, Aw J, Francis S, et al. Critical appraisal of a mHealth-assisted community-based cardiovascular disease risk screening program in rural Kenya: an operational research study. Pathogens and global health. 2020;114(7):379-87.
577. Aw M, Ochieng BO, Attambo D, Opot D, Aw J, Francis S, et al. Critical appraisal of a mHealth-assisted community-based cardiovascular disease risk screening program in rural Kenya: an operational research study. Pathogens and Global Health. 2020;114(7):379-87.
578. Aw M, Ochieng BO, Attambo D, Opot D, Aw J, Francis S, et al. Critical appraisal of a mHealth-assisted community-based cardiovascular disease risk screening program in rural Kenya: an operational research study. Pathogens and Global Health. 2020;114(7):379-87.
579. Aw M, Ochieng BO, Attambo D, Opot D, Aw J, Francis S, et al. Critical appraisal of a mHealth-assisted community-based cardiovascular disease risk screening program in rural Kenya: an operational research study. Pathogens and global health. 2020;114(7):379-87.
580. Aw M, Ochieng BO, Attambo D, Opot D, Aw J, Francis S, et al. Critical appraisal of a mHealth-assisted community-based cardiovascular disease risk screening program in rural Kenya: an operational research study. Pathogens and global health. 2020;114(7):379-87.
581. Aw M, Ochieng BO, Attambo D, Opot D, Aw J, Francis S, et al. Critical appraisal of a mHealth-assisted community-based cardiovascular disease risk screening program in rural Kenya: an operational research study. Pathogens and Global Health. 2020;114(7):379-87.
582. Aw M, Ochieng BO, Attambo D, Opot D, Aw J, Francis S, et al. Critical appraisal of a mHealth-assisted community-based cardiovascular disease risk screening program in rural Kenya: an operational research study. Pathogens and Global Health. 2020;114(7):379-87.
583. Asgary R, Sckell B, Alcabes A, Naderi R, Schoenthaler A, Ogedegbe G. Rates and Predictors of Uncontrolled Hypertension Among Hypertensive Homeless Adults Using New York City Shelter-Based Clinics. Ann Fam Med. 2016;14(1):41-6.
584. Asan O, Cooper Ii F, Nagavally S, Walker RJ, Williams JS, Ozieh MN, et al. Preferences for Health Information Technologies Among US Adults: Analysis of the Health Information National Trends Survey. J Med Internet Res. 2018;20(10):e277.
585. Artinian NT, Washington OG, Templin TN. Effects of home telemonitoring and community-based monitoring on blood pressure control in urban African Americans: a pilot study. Heart Lung. 2001;30(3):191-9.
586. Artinian NT, Flack JM, Nordstrom CK, Hockman EM, Washington

OG, Jen KL, et al. Effects of nurse-managed telemonitoring on blood pressure at 12-month follow-up among urban African Americans. Nurs Res. 2007;56(5):312-22.
587. Aronson T, Dave J, Ras T. Improving outcomes in co-morbid diabetes and COVID-19: A quasi-experimental study. South African Family Practice. 2023;65(1):a5631.
588. Aronson T, Dave J, Ras T. Improving outcomes in co-morbid diabetes and COVID-19: A quasi-experimental study. South African Family Practice. 2023;65(1):a5631.
589. Aovare P, Abdulai K, Laar A, van der Linden EL, Moens N, Richard E, et al. Assessing the Effectiveness of mHealth Interventions for Diabetes and Hypertension Management in Africa: Systematic Review and Meta-Analysis. JMIR mHealth and uHealth. 2023;11:e43742-e.
590. Aovare P, Abdulai K, Laar A, van der Linden EL, Moens N, Richard E, et al. Assessing the Effectiveness of mHealth Interventions for Diabetes and Hypertension Management in Africa: Systematic Review and Meta-Analysis. JMIR Mhealth Uhealth. 2023;11:e43742.
591. Anuluoha ACZ. Use of Telehealth Education to Improve Medication Adherence for African American Adults with Hypertension2021 2021.
592. Anstey Watkins J, Goudge J, Gómez-Olivé FX, Huxley C, Dodd K, Griffiths F. mHealth text and voice communication for monitoring people with chronic diseases in low-resource settings: a realist review. BMJ Glob Health. 2018;3(2):e000543.
593. Anstey Watkins J, Goudge J, Gomez-Olive FX, Huxley C, Dodd K, Griffiths F. mHealth text and voice communication for monitoring people with chronic diseases in low-resource settings: a realist review. BMJ global health. 2018;3(2):e000543.
594. Anstey Watkins J, Goudge J, Gomez-Olive FX, Huxley C, Dodd K, Griffiths F. mHealth text and voice communication for monitoring people with chronic diseases in low-resource settings: a realist review. BMJ global health. 2018;3(2):e000543.
595. Anonymous. Planetary Health Annual Meeting 2019. The Lancet Planetary Health. 2019;3(Supplement 1):S1-S22.
596. Anonymous. Planetary Health Annual Meeting 2019. The Lancet Planetary Health. 2019;3(Supplement 1):S1-S22.
597. Andrews LJB, Raja L, Shanmugasundaram S. Mobile android-based remote patient monitoring system through wearable sensors. Journal of Discrete Mathematical Sciences and Cryptography. 2019;22(4):557-68.
598. Amuasi J, Agbogbatey MK, Sarfo FS, Beyuo A, Duah K, Agasiya P, et al. Feasibility, acceptability, and appropriateness of a mobile health stroke intervention among Ghanaian health workers. J Neurol Sci. 2022;439:120304.
599. Amuasi J, Agbogbatey MK, Sarfo FS, Beyuo A, Duah K, Agasiya P, et al. Feasibility, acceptability, and appropriateness of a mobile health stroke intervention among Ghanaian health workers. Journal of the Neurological Sciences. 2022;439:1-5.
600. Amuasi J, Agbogbatey MK, Sarfo FS, Beyuo A, Duah K, Agasiya P, et al. Feasibility, acceptability, and appropriateness of a mobile health stroke intervention among Ghanaian health workers. Journal of

the neurological sciences. 2022;439:120304.
601. Amuasi J, Agbogbatey MK, Sarfo FS, Beyuo A, Duah K, Agasiya P, et al. Feasibility, acceptability, and appropriateness of a mobile health stroke intervention among Ghanaian health workers. Journal of the Neurological Sciences. 2022;439:120304.
602. Amuasi J, Agbogbatey MK, Sarfo FS, Beyuo A, Duah K, Agasiya P, et al. Feasibility, acceptability, and appropriateness of a mobile health stroke intervention among Ghanaian health workers. Journal of the neurological sciences. 2022;439:120304.
603. Amuasi J, Agbogbatey MK, Sarfo FS, Beyuo A, Duah K, Agasiya P, et al. Feasibility, acceptability, and appropriateness of a mobile health stroke intervention among Ghanaian health workers. Journal of the neurological sciences. 2022;439:120304.
604. Amuasi J, Agbogbatey MK, Sarfo FS, Beyuo A, Duah K, Agasiya P, et al. Feasibility, acceptability, and appropriateness of a mobile health stroke intervention among Ghanaian health workers. Journal of the Neurological Sciences. 2022;439:120304.
605. Amuasi J, Agbogbatey MK, Sarfo FS, Beyuo A, Duah K, Agasiya P, et al. Feasibility, acceptability, and appropriateness of a mobile health stroke intervention among Ghanaian health workers. Journal of the Neurological Sciences. 2022;439:1-5.
606. Amoakoh HB, Klipstein-Grobusch K, Grobbee DE, Amoakoh-Coleman M, Oduro-Mensah E, Sarpong C, et al. Using Mobile Health to Support Clinical Decision-Making to Improve Maternal and Neonatal Health Outcomes in Ghana: Insights of Frontline Health Worker Information Needs. JMIR Mhealth Uhealth. 2019;7(5):e12879.
607. Amoakoh HB, Klipstein-Grobusch K, Grobbee DE, Amoakoh-Coleman M, Oduro-Mensah E, Sarpong C, et al. Using Mobile Health to Support Clinical Decision-Making to Improve Maternal and Neonatal Health Outcomes in Ghana: Insights of Frontline Health Worker Information Needs. JMIR mHealth and uHealth. 2019;7(5):e12879.
608. Amoakoh HB, Klipstein-Grobusch K, Grobbee DE, Amoakoh-Coleman M, Oduro-Mensah E, Sarpong C, et al. Using Mobile Health to Support Clinical Decision-Making to Improve Maternal and Neonatal Health Outcomes in Ghana: Insights of Frontline Health Worker Information Needs. JMIR mHealth and uHealth. 2019;7(5):e12879.
609. Amoakoh HB, Klipstein-Grobusch K, Grobbee DE, Amoakoh-Coleman M, Oduro-Mensah E, Sarpong C, et al. Using Mobile Health to Support Clinical Decision-Making to Improve Maternal and Neonatal Health Outcomes in Ghana: Insights of Frontline Health Worker Information Needs. JMIR mHealth and uHealth. 2019;7(5):e12879.
610. Aliku T, Dewyer A, Namuyonga J, Ssinabulya I, Kamarembo J, Okello E, et al. Telemedicine Support of Cardiac Care In Northern Uganda: Leveraging Hand-held Echocardiography and Task Shifting. Global Heart. 2018;13(4):382.
611. Aliku T, Dewyer A, Namuyonga J, Ssinabulya I, Kamarembo J, Okello E, et al. Telemedicine Support of Cardiac Care In Northern Uganda: Leveraging Hand-held Echocardiography and Task Shifting. Global Heart. 2018;13(4):382.
612. Alene KA, Gelaw YA, Fetene DM, Koye DN, Melaku YA, Gesesew H,

et al. COVID-19 in Ethiopia: A geospatial analysis of vulnerability to infection, case severity and death. BMJ Open. 2021;11(2):e044606.
613. Alene KA, Gelaw YA, Fetene DM, Koye DN, Melaku YA, Gesesew H, et al. COVID-19 in Ethiopia: A geospatial analysis of vulnerability to infection, case severity and death. BMJ Open. 2021;11(2):e044606.
614. Akutey R, Der R, Owusu-Daaku F, Baiden F. Using community pharmacies to expand access to screening for noncommunicable diseases in suburban Ghana-A facility-based survey on client needs and acceptability. Health Sci Rep. 2018;1(9):e79.
615. Akematsu Y, Tsuji M, editors. Empirical analysis of the reduction of medical expenditures by eHealth. 13th World Congress on Medical and Health Informatics, Medinfo 2010, September 12, 2010 - September 15, 2010; 2010; Cape Town, South africa: IOS Press BV.
616. Akam EY, Nuako AA, Daniel AK, Stanford FC. Racial Disparities and Cardiometabolic Risk: New Horizons of Intervention and Prevention. Curr Diab Rep. 2022;22(3):129-36.
617. Ajayi IOO, Oyewole OE, Ogah OS, Akinyemi JO, Salawu MM, Bamgboye EA, et al. Development and evaluation of a package to improve hypertension control in Nigeria [DEPIHCON]: a cluster-randomized controlled trial. Trials. 2022;23(1):366.
618. Ajayi IOO, Oyewole OE, Ogah OS, Akinyemi JO, Salawu MM, Bamgboye EA, et al. Development and evaluation of a package to improve hypertension control in Nigeria [DEPIHCON]: a cluster-randomized controlled trial. Trials. 2022;23(1):366.
619. Agbogbatey MK, Sarfo FS, Beyuo A, Duah K, Agasiya P, Tagge R, et al. FEASIBILITY, ACCEPTABILITY, AND APPROPRIATENESS OF THE PHONE BASED INTERVENTIONS UNDER NURSE GUIDANCE AFTER STROKE (PINGS) IN GHANA. Journal of Stroke and Cerebrovascular Diseases. 2022;31(4):106374.
620. Agbogbatey MK, Sarfo FS, Beyuo A, Duah K, Agasiya P, Tagge R, et al. FEASIBILITY, ACCEPTABILITY, AND APPROPRIATENESS OF THE PHONE BASED INTERVENTIONS UNDER NURSE GUIDANCE AFTER STROKE (PINGS) IN GHANA. Journal of Stroke and Cerebrovascular Diseases. 2022;31(4):106374.
621. Adepoju AA, Vidler M, Akadri AA, Jaiyesimi E, Nwankpa CC, Odubena OO, et al. The ability and safety of community-based health workers to safely initiate lifesaving therapies for pre-eclampsia in Ogun State, Nigeria: An analysis of 260 community treatments with MgSO4 and/or methyldopa. Pregnancy hypertension. 2021;25:179-84.
622. Adepoju AA, Vidler M, Akadri AA, Jaiyesimi E, Nwankpa CC, Odubena OO, et al. The ability and safety of community-based health workers to safely initiate lifesaving therapies for pre-eclampsia in Ogun State, Nigeria: An analysis of 260 community treatments with MgSO(4) and/or methyldopa. Pregnancy Hypertens. 2021;25:179-84.
623. Adepoju AA, Vidler M, Akadri AA, Jaiyesimi E, Nwankpa CC, Odubena OO, et al. The ability and safety of community-based health workers to safely initiate lifesaving therapies for pre-eclampsia in Ogun State, Nigeria: An analysis of 260 community treatments with MgSO4 and/or methyldopa. Pregnancy hypertension. 2021;25:179-84.
624. Adepoju AA, Vidler M, Akadri AA, Jaiyesimi E, Nwankpa CC,

Odubena OO, et al. The ability and safety of community-based health workers to safely initiate lifesaving therapies for pre-eclampsia in Ogun State, Nigeria: An analysis of 260 community treatments with MgSO4 and/or methyldopa. Pregnancy Hypertension. 2021;25:179-84.
625. Adepoju AA, Vidler M, Akadri AA, Jaiyesimi E, Nwankpa CC, Odubena OO, et al. The ability and safety of community-based health workers to safely initiate lifesaving therapies for pre-eclampsia in Ogun State, Nigeria: An analysis of 260 community treatments with MgSO4 and/or methyldopa. Pregnancy hypertension. 2021;25:179-84.
626. Adepoju AA, Vidler M, Akadri AA, Jaiyesimi E, Nwankpa CC, Odubena OO, et al. The ability and safety of community-based health workers to safely initiate lifesaving therapies for pre-eclampsia in Ogun State, Nigeria: An analysis of 260 community treatments with MgSO4 and/or methyldopa. Pregnancy Hypertension. 2021;25:179-84.
627. Abul-Fadl AM, Sanad AR, Khallaf M, Ibrahim RS, Sarhan AE. E-Health Platforms for Strengthening Control of Cardiovascular Diseases in 14 Countries. Global Heart. 2018;13(4):462.
628. Abul-Fadl AM, Sanad AR, Khallaf M, Ibrahim RS, Sarhan AE. E-Health Platforms for Strengthening Control of Cardiovascular Diseases in 14 Countries. Global Heart. 2018;13(4):462.
629. Abebe BT, Weiss M, Modess C, Roustom T, Tadken T, Wegner D, et al. Effects of the P‐Glycoprotein Inhibitor Clarithromycin on the Pharmacokinetics of Intravenous and Oral Trospium Chloride: a 4‐Way Crossover Drug‐Drug Interaction Study in Healthy Subjects. Journal of clinical pharmacology. 2019;59(10):1319.
630. Abebe BT, Weiss M, Modess C, Roustom T, Tadken T, Wegner D, et al. Effects of the P‐Glycoprotein Inhibitor Clarithromycin on the Pharmacokinetics of Intravenous and Oral Trospium Chloride: a 4‐Way Crossover Drug‐Drug Interaction Study in Healthy Subjects. Journal of clinical pharmacology. 2019;59(10):1319.
631. "Community-based, eHealth Supported Management of Cardiovascular Risk Factors by Lay Village Health Workers (ComBaCaL aHT TwiC 1 & ComBaCaL aHT TwiC 2). Community-based, eHealth Supported Management of Cardiovascular Risk Factors by Lay Village Health Workers for People With Controlled and Uncontrolled Arterial Hypertension in Rural Lesotho: joint Protocol for Two Cluster-randomized Trials Within the ComBaCaL Cohort Study (ComBaCaL aHT TwiC 1 & ComBaCaL aHT TwiC 2). 2023.
632. "Community-based, eHealth Supported Management of Cardiovascular Risk Factors by Lay Village Health Workers (ComBaCaL aHT TwiC 1 & ComBaCaL aHT TwiC 2). Community-based, eHealth Supported Management of Cardiovascular Risk Factors by Lay Village Health Workers for People With Controlled and Uncontrolled Arterial Hypertension in Rural Lesotho: joint Protocol for Two Cluster-randomized Trials Within the ComBaCaL Cohort Study (ComBaCaL aHT TwiC 1 & ComBaCaL aHT TwiC 2). 2023.
633. Phone-based support for high blood pressure control feasibility study. Improving control of hypertension in a high HIV prevalence setting: a feasibility cluster-randomized trial. 2021.
634. Effectiveness of a Midwife Led Continuity of Antenatal care

caseload model on preterm birth and maternal satisfaction among women in Malawi. Effectiveness of a Midwife Led Continuity of Antenatal care caseload model on preterm birth and maternal satisfaction among women in Malawi: a randomised clinical trial. 2021.
635. Determining trustworthiness and safety of remote consulting during the COVID-19 pandemic in primary healthcare for chronic disease populations in Nigeria and Tanzania. COVID-19: determining trustworthiness and safety of REmote Consulting in primary Healthcare for chronic disease populations in Nigeria and Tanzania using a stepped wedge design - The REaCH Trial. 2021.
636. Phone-based support for high blood pressure control feasibility study. Improving control of hypertension in a high HIV prevalence setting: a feasibility cluster-randomized trial. 2021.
637. Effectiveness of a Midwife Led Continuity of Antenatal care caseload model on preterm birth and maternal satisfaction among women in Malawi. Effectiveness of a Midwife Led Continuity of Antenatal care caseload model on preterm birth and maternal satisfaction among women in Malawi: a randomised clinical trial. 2021.
638. Determining trustworthiness and safety of remote consulting during the COVID-19 pandemic in primary healthcare for chronic disease populations in Nigeria and Tanzania. COVID-19: determining trustworthiness and safety of REmote Consulting in primary Healthcare for chronic disease populations in Nigeria and Tanzania using a stepped wedge design - The REaCH Trial. 2021.
639. An mHealth Strategy to Reduce Pre-Eclampsia and Infant Death in Tanzania. A Cluster Randomized, Parallel-group, Superiority Study to Compare the Effectiveness of a Community-based mHealth Strategy Versus Enhanced Usual Care in the Detection and Management of Pre-eclampsia/Eclampsia in Tanzania. 2019.
640. An mHealth Strategy to Reduce Pre-Eclampsia and Infant Death in Tanzania. A Cluster Randomized, Parallel-group, Superiority Study to Compare the Effectiveness of a Community-based mHealth Strategy Versus Enhanced Usual Care in the Detection and Management of Pre-eclampsia/Eclampsia in Tanzania. 2019.
641. Mobile phone SMS for adherence support for hypertension in South African adults in care for HIV infection. MObile Phone text messages to support Hypertension treatment ADherence in adults attending HIV treatment centres in the Western Cape Province of South Africa: a pilot study â“ The MOPHADHIV trial. 2018.
642. Mobile phone SMS for adherence support for hypertension in South African adults in care for HIV infection. MObile Phone text messages to support Hypertension treatment ADherence in adults attending HIV treatment centres in the Western Cape Province of South Africa: a pilot study â“ The MOPHADHIV trial. 2018.
643. Phone-based Intervention Under Nurse Guidance After Stroke. 2015.
644. Phone-based Intervention Under Nurse Guidance After Stroke. 2015.
645. Hypertension health promotion via Short Message Service (SMS) at a Community Health Centre in South Africa: a mixed method study.

Can cell phones lower language barriers and advance the right of access to health information ¿ and if so how? A case study among hypertensive patients in a Community Health Centre in Cape Town. 2014.
646. Hypertension health promotion via Short Message Service (SMS) at a Community Health Centre in South Africa: a mixed method study. Can cell phones lower language barriers and advance the right of access to health information ¿ and if so how? A case study among hypertensive patients in a Community Health Centre in Cape Town. 2014.
647. Effectiveness of SMS-text Message Support for Patients With Hypertension to Improve Blood Pressure. SMS-text Adherence Support (StAR*) Study, a Randomized Three-arm Open Parallel Group Trial to Evaluate the Effects of a Structured Programme of Hypertension Treatment Adherence Support Delivered Remotely Through SMS-text Messages on Blood Pressure at 12 Months as Compared With Usual Care. 2013.
648. Community Level Interventions for Pre-eclampsia. The CLIP (Community Level Interventions for Pre-eclampsia) Cluster Randomized Controlled Trial. 2013.
649. Effectiveness of SMS-text Message Support for Patients With Hypertension to Improve Blood Pressure. SMS-text Adherence Support (StAR*) Study, a Randomized Three-arm Open Parallel Group Trial to Evaluate the Effects of a Structured Programme of Hypertension Treatment Adherence Support Delivered Remotely Through SMS-text Messages on Blood Pressure at 12 Months as Compared With Usual Care. 2013.
650. Community Level Interventions for Pre-eclampsia. The CLIP (Community Level Interventions for Pre-eclampsia) Cluster Randomized Controlled Trial. 2013.
