# Supplementary material for: Mobile health interventions for African adults with hypertension: Evidence from a systematic review of implementation research outcomes
Source: PLOS Digit Health. 2026 Aug 14;5(8):e0001612. doi: 10.1371/journal.pdig.0001612 (PMC13475905; doi:10.1371/journal.pdig.0001612)
Supplement: Supplementary file 2 [file pdig.0001612.s015.pdf]

PLOS Digital Health

Determi[illegible]g Acceptability, Feasibility, and Adoption rates associated with Mobile Health interventions for hypertension management among adults in Africa - A Systematic Review

--Manuscript Draft--

**Manuscript Number:**	PDIG-D-24-00124
**Article Type:**	Research Article
**Full Title:**	Determining Acceptability, Feasibility, and Adoption rates associated with Mobile Health interventions for hypertension management among adults in Africa - A Systematic Review
**Short Title:**	Mobile Health and Hypertension Management
**Corresponding Author:**	Priyanka Lanka, MDS<br>New York University<br>New York City, New York UNITED STATES
**Order of Authors:**	Priyanka Lanka, MDS
	Ayomide Enitan, MPH
	Angela Makgabo
	Sai Sravanthi Rompicharla
	Afnan Alsufyani
	John Patena, MPH,MA
	Dorice Vieira, MLS, MA, MPH
	Joyce Gyamfi, EdD, MS
	Emmanuel Peprah, PhD
**Keywords:**	
**Abstract:**	Introduction - Africa faces a disproportionate burden of hypertension, with a prevalence of 27% compared to 18% in the Americas. Despite cost-effective lifestyle and medical interventions, the region suffers from poor detection, treatment, and control rates. With the advent of technology, mobile health (mHealth) interventions offer promise in managing hypertension, but their implementation outcomes in African countries remain underexplored. Therefore, this systematic review aims to determine the acceptability, feasibility, and adoption rates of mHealth intervention among adults diagnosed with hypertension across African countries.<br>Methods - Employing a rigorous approach, we conducted a systematic review across nine databases, adhering to the PRISMA checklist and World Bank criteria for LMICs. Risk of bias was evaluated using the ASSESS tool. Data extraction involved meticulous screening with tools like Covidence to ensure accuracy, resolving conflicts by consensus.<br>Results - We found eighteen articles focusing on mHealth interventions for hypertension in Africa, exploring acceptability (n=10), feasibility (n=11), adoption (n=4), and appropriateness (n=2). Service outcomes including effectiveness (n=3), efficacy (n=1), patient-centeredness (n=1), and scalability (n=2) were also examined. Studies exclusively targeted African adults diagnosed with hypertension, revealing robust acceptability and effectiveness. However, feasibility challenges, mainly financial constraints and cellular network issues, were prominent. Most studies were urban-centric, indicating a gap in understanding rural challenges. Risk of bias was low across all the included studies.<br>Conclusion - Application-based mHealth interventions have gained traction in Africa, especially in countries like Nigeria, Ghana, and South Africa. SMS-based interventions show promise in reaching non-smartphone users. Despite the observed successes, feasibility challenges persist, highlighting the need for targeted interventions addressing financial constraints and network infrastructure. Future research should aim to bridge the urban-rural gap and explore innovative solutions to enhance the feasibility

Powered by Editorial Manager® and ProduXion Manager® from Aries Systems Corporation

	as well as sustainability of mHealth interventions for hypertension management across diverse African settings.
**Additional Information:**	
**Question**	**Response**
**Government Employee**<br><br>Are you or any of the contributing authors an employee of the United States government?<br><br>Manuscripts authored by one or more US Government employees are not copyrighted, but are licensed under a CC0 Public Domain Dedication, which allows unlimited distribution and reuse of the article for any lawful purpose. This is a legal requirement for US Government employees.<br><br>This will be typeset if the manuscript is accepted for publication.	No - No authors are employees of the U.S. government.
**Financial Disclosure**<br><br>Enter a financial disclosure statement that describes the sources of funding for the work included in this submission. Review the submission guidelines for detailed requirements. View published research articles from *PLOS Digital Health* for specific examples.<br><br>This statement is required for submission and **will appear in the published article** if the submission is accepted. Please make sure it is accurate.	The author(s) received no specific funding for this work.

**Funded studies**<br>Enter a statement with the following details:<br>• Initials of the authors who received each award<br>• Grant numbers awarded to each author<br>• The full name of each funder<br>• URL of each funder website<br>• Did the sponsors or funders play any role in the study design, data collection and analysis, decision to publish, or preparation of the manuscript?<br><br>Did you receive funding for this work?	
**Competing Interests**<br><br>On behalf of all authors, disclose any competing interests that could be perceived to bias this work.<br><br>This statement will be typeset if the manuscript is accepted for publication.<br><br>Review the instructions link below and PLOS Digital Health's competing interests policy to determine what information must be disclosed at submission.	The authors have declared that no competing interests exist.
**Data Availability**<br><br>Provide a **Data Availability Statement** in the box below. This statement should detail where the data used in this submission can be accessed. This statement will be typeset if the manuscript is accepted for publication.<br><br>Before publication, authors are required to make all data underlying their findings fully available, without restriction. Review our PLOS Data Policy page for detailed information on this policy. Instructions for writing your Data Availability statement can be accessed via the Instructions link below.	This systematic review was registered with OSF and is available at https://osf.io/f7dhe

Powered by Editorial Manager® and ProduXion Manager® from Aries Systems Corporation

Manuscript

Click here to access/download;Manuscript;Hypertension and mHealth and Hypertension MANUSCRIPT.docx

Determining Acceptability, Feasibility, and Adoption rates associated with Mobile Health interventions for hypertension management among adults in Africa - A Systematic Review

Priyanka Lanka[1,2 ¶*], *Ayomide Enitan*[1¶&], *Angela Makgabo*[1,2¶&], *Sai Sravanthi Rompicharla*[1¶], *Afnan Alsufyani*[1¶], *John Patena*[1,2¶], *Dorice Vieira*[1,2&], *Joyce Gyamfi*[1,2&], *Emmanuel Peprah*[1,2&]

Affiliations

1. School of Global Public Health, New York University, New York City, NY, USA
2. ISEE (Implementing Sustainable Evidence-based interventions through Engagement) Lab, School of Global Public Health, New York University, New York City, NY, USA

Contributions

Priyanka Lanka - *Conceptualization, Investigation, Data curation, Data Extraction, Formal analysis, Project administration, Writing – original draft, Writing – review & editing*

Ayomide Enitan - *Conceptualization, Data Extraction, Formal analysis, Writing – original draft, Writing - review & editing*

Angela Makgabo - *Conceptualization, Data Extraction, Formal analysis, Writing – original draft, Writing - review & editing*

Sai Sravanthi Rompicharla - *Data Extraction, Writing – review & editing*

Afnan Alsufyani - *Data Extraction*

John Patena - *Resources, Software, Supervision, Writing – review & editing*

Dorice Vieira - *Data extraction, Resources, Software*

Joyce Gyamfi - *Writing – review & editing*

Emmanuel Peprah - *Validation, Supervision, Writing – review & editing*

1. Abstract

Introduction: Africa faces a disproportionate burden of hypertension, with a prevalence of 27% compared to 18% in the Americas. Despite cost-effective lifestyle and medical interventions, the region suffers from poor detection, treatment, and control rates. With the advent of technology, mobile health (mHealth) interventions offer promise in managing hypertension, but their implementation outcomes in African countries remain underexplored. Therefore, this systematic review aims to determine the acceptability, feasibility, and adoption rates of mHealth intervention among adults diagnosed with hypertension across African countries.

Methods: Employing a rigorous approach, we conducted a systematic review across nine databases, adhering to the PRISMA checklist and World Bank criteria for LMICs. Risk of bias was evaluated using the ASSESS tool. Data extraction involved meticulous screening with tools like Covidence to ensure accuracy, resolving conflicts by consensus.

Results: We found eighteen articles focusing on mHealth interventions for hypertension in Africa, exploring acceptability (n=10), feasibility (n=11), adoption (n=4), and appropriateness (n=2). Service outcomes including effectiveness (n=3), efficacy (n=1), patient-centeredness (n=1), and scalability (n=2) were also examined. Studies exclusively targeted African adults diagnosed with hypertension, revealing robust acceptability and effectiveness. However, feasibility challenges, mainly financial constraints and cellular network issues, were prominent. Most studies were urban-centric, indicating a gap in understanding rural challenges. Risk of bias was low across all the included studies.

Conclusion: Application-based mHealth interventions have gained traction in Africa, especially in countries like Nigeria, Ghana, and South Africa. SMS-based interventions show promise in reaching non-smartphone users. Despite the observed successes, feasibility challenges persist, highlighting the need for targeted interventions addressing financial constraints and network infrastructure. Future research should aim to bridge the urban-rural gap and explore innovative solutions to enhance the feasibility as well as sustainability of mHealth interventions for hypertension management across diverse African settings.

2. Background/Introduction

Hypertension affects over 1.28 billion adults aged 30–79 years globally, with at least two-thirds (857 million) accounting for people living in low- and middle-income countries (LMICs).[1] Africa is disproportionately burdened with hypertension compared to other LMICs, with the World Health Organization estimating the highest prevalence of hypertension (27%) in the region compared to prevalence rates of 18% in the Americas.[2] Despite cost-effective lifestyle and medical interventions for hypertension control, with potential to avoid unnecessary death and disability, the African region still bears a very high disease prevalence, with poor rates of detection, treatment, and control.

Hypertension, defined as an average of repeatedly measured systolic blood pressure ≥ 130 mmHg and or diastolic blood pressure ≥80 mm Hg[3], is the leading preventable risk factor for cardiovascular diseases (CVD) and disability globally.[2] The largest number of deaths attributed to elevated systolic BP are linked to Ischaemic heart disease (4.9 million), ischaemic stroke (1.5 million), and hemorrhagic stroke (2 million).[4] Notably, there is a significant rise in stroke prevalence in sub-Saharan Africa, with hypertension serving as a highly modifiable risk factor.[5] Hypertension and diabetes also share common risk factors, including obesity, physical inactivity, and unhealthy dietary habits. Similarly, in pregnant women the incidence of preeclampsia exacerbated by hypertension, is significantly higher in Africa (10 % of all pregnant women in Africa suffer from pregnancy induced hypertension compared to the global average of 2 percent).[6] However, due to the complexity of disease pathology addressing this research gap is beyond the scope of our study. Within African populations, where lifestyle shifts and

urbanization have altered diets and activity levels, the prevalence of hypertension alongside comorbidities such as diabetes and stroke are on the ascent.

Evidence-based interventions have been developed to assist patients in managing their hypertension including educational initiatives to improve hypertension knowledge, medication adherence strategies, medical team-based approaches to increase access to preventative care, and community-based screening and self-monitoring.[7] The use of community- and peer-based interventions seems especially promising for reaching underserved populations.[8] Mobile health (mHealth) is one such intervention that offers a potential approach to managing chronic conditions in LMICs. mHealth is a subset of digital health and encompasses a wide range of mobile and wireless technologies to help improve health care and overcome barriers such as limited access to healthcare facilities and resources.[9] It has demonstrated efficacy in enhancing patient–provider communication and supporting disease management through various channels. This includes the dissemination of healthy lifestyle information via Short-Message-Service (SMS), utilizing mHealth applications (mHealth Apps) for remote monitoring of blood pressure readings to monitor patients' progress, sending reminders for medication schedules to enhance treatment plan adherence, and providing tele/remote consultations for patients who cannot physically visit a healthcare facility for in-person consultations with a healthcare provider. The adoption and application of information and communications technology (ICT) tools have been seen as a sort of silver lining through which health systems in various countries can be improved. Notably, the prevalence and penetration of mobile technologies, especially mobile phones in LMICs, have proven to

be a major ingredient needed to improve healthcare service delivery[10], although its sustainability is challenging due to internet connectivity challenges.

In 2021, the number of mobile phone users worldwide stood at 7.1 billion, with forecasts suggesting this is likely to rise to 7.49 billion by 2025.[11] Within continents, there is country-to-country variability, but penetration in some LMICs exceeds 100%.[12] With the basic connectivity infrastructure in place, the integration of mobile technology into the healthcare system may be a feasible way to complement and improve current chronic disease management strategies in LMICs. According to Gupta et al, 2014, the widespread adoption of mobile technology is positively impacting the African region, as it is being utilized to address health problems.[13]

To date, relatively few studies address usability and feasibility issues from an African context. With Africa facing a disproportionately high burden of hypertension, contributing significantly to the region's overall disease profile, understanding the acceptability and feasibility of mHealth interventions is vital for addressing this prevalent health issue. Many regions in Africa also experience challenges related to limited access to healthcare services due to compromised health care systems with scarce human and material resources.[14] mHealth interventions have the potential to bridge gaps in accessibility, thus offering a feasible solution for reaching a broader population and providing timely hypertension management. Assessing the acceptability of mHealth interventions ensures that healthcare approaches align with patients' preferences and needs. Therefore, tailoring hypertension management to be patient-centric enhances engagement, self - efficacy, and adherence, thus contributing to improved health outcomes. Understanding

the feasibility and adoption rates of mHealth interventions in Africa will contribute to health system strengthening. Identifying potential barriers and facilitators to implementation will allow for strategic planning and resource allocation, optimizing the integration of mHealth into existing healthcare structures.

This systematic review utilitizes Proctor's framework on implementation research outcomes to provide insights, contributing factors, and limitations that may exist in the implementation and scale-up of mHealth solutions in the management of Hypertension amongst adults in Africa.[15] Specifically, this review examines the acceptability, adoption, and feasibility of mHealth interventions within the African context. According to Proctor et al.,[15] acceptability is characterized by the perception among implementation stakeholders (including beneficiaries and implementers) that the innovation is agreeable, palatable, or satisfactory. Adoption is defined as the intention, initial decision, or action to try or employ the innovation (i.e., uptake). Lastly, feasibility is defined as the extent to which the innovation can be successfully utilized or carried out within a given agency or setting.[15]

3. Methods

Search Strategy

This systematic review was registered with OSF (https://osf.io/f7dhe). We developed an extensive search strategy to locate published trials across nine databases employing the Preferred Reporting Items for Systematic Reviews and Meta-Analysis (PRISMA) [16] and the World Bank [1] criteria to delineate LMICs. Our search strategy did not impose any

date restrictions. The following databases were searched: Pubmed, Embase, Engineering Village, Medline, Cochrane Library, Global Health, Web of Science, PAIS (Public Affairs Information Service), and PsycINFO. We developed a set of comprehensive search syntax specific for each database including key terms and their Medical Subject Heading (MeSH) for 'hypertension', 'high blood pressure,' 'mobile health,' and 'implementation outcomes' according to Proctor et al[15] and 'Africa' as defined by the World Bank. The search was conducted October 2023 and updated in January 2024. A sample of the search strategy is provided in Appendix A.

<u>Inclusion and Exclusion criteria</u>

The articles were included if they met the following criteria: 1) Studies addressing hypertension management (either on prevalence, treatment, screening, referral to care, retention in care, treatment adherence) amongst adults across disease categories such as diabetes and stroke, 2) Study population diagnosed with hypertension 3) Studies that investigate the use of mHealth intervention in the management of hypertension, and 4) Studies conducted in Africa.

Studies were excluded if they met the following criteria: 1) Studies related to Preeclampsia amongst pregnant women. 2) Studies conducted amongst African populations living abroad, 3) Studies not addressing hypertension management amongst adults, and 4) Study designs such as systematic reviews, abstracts, protocols, and commentaries.

Data extraction

Resulting articles were imported into a citation manager tool, Endnote, followed by a screening and data extraction tool for conducting systematic reviews, Covidence, where duplicates were removed. Title and abstracts were screened by a minimum of two reviewers and conflicts were resolved by a third reviewer which was selected randomly by Covidence. Each article underwent assessment according to the implementation outcomes outlined by Proctor et al.,[15] encompassing Adoption, Appropriateness, Acceptability, Cost, Feasibility, Fidelity, Penetration, and Sustainability. The service outcomes outlined by Proctor et al.,[15] include Efficiency, Safety, Effectiveness, Equity, Patient centeredness and Timeliness. For the purpose of this review, we focused on relevant information to address the acceptability, adoption, and feasibility of mHealth interventions within the African context. We also draw attention to other implementation and service outcomes reported by the included articles.

4. Results

Literature Search

The initial database search produced 650 articles, resulting in 265 remaining after the elimination of 385 duplicates. Upon conducting a more detailed review of the abstract and title screening process, we identified 204 articles as irrelevant. These exclusions were based on criteria aligned with our research parameters, including studies conducted on African populations outside of Africa, studies lacking mHealth as part of their intervention, study designs such as study protocols, and conference abstracts. Sixty articles were

further assessed under full text review out of which ultimately 18 articles were found to be relevant to the review topic. All articles included in the final review and data extraction were recently published (ranging from 2012 to 2023). Our review comprised randomized control trials (n=8), qualitative assessments (n=1), quantitative non-randomized assessments (n=3), and mixed methods study design (n=6). We visually outlined the data extraction process through a PRISMA flowchart, developed using the Covidence search and data extraction tool **(Figure 1:** PRISMA flow chart. PRISMA, Preferred Reporting Items for Systematic Reviews and Meta-Analysis**)**.

Characteristics of identified studies

I. Demographic and Study Duration Characteristics

Characteristics of the studies reviewed are summarized in **Table 1**. All included studies targeted adult populations, encompassing both men and women with varying age criteria. The mean duration of the studies was approximately eleven months, ranging from a minimum of one month to a maximum of thirty-two months. It was interesting to note that 38% of the 18 studies recorded higher female participants than male.

II. Geographical Distribution and Setting

Fourteen studies predominantly collected data in urban settings, three in rural areas, and one in a peri-urban location. Notably, 39% of the 18 studies were conducted in Ghana, 33% in Nigeria, and 17% in South Africa, with the remaining studies in Kenya, Uganda and Tanzania.

III. Target Population and Health Focus

Most of the experimental research concentrated on subjects with elevated blood pressure and confirmed hypertension. The studies aimed to enhance hypertension control, treatment adherence, health knowledge, or assess the effectiveness of mHealth tools in hypertension management. Comorbidity considerations were present in five studies involving patients with stroke and hypertension, one study involving diabetes mellitus and one involving patients with HIV.

IV. Technological Interventions

Mobile phone technology, including SMS and mobile Apps, was uniformly utilized across all studies. Our findings revealed successful strategies employing SMS to improve adherence behaviors and a high receptivity towards mHealth apps for blood pressure monitoring. However, some articles identified concerns related to patients' ability to use technology, comprehend relayed information, and inconsistencies in network connectivity.

V. Study Design and Technological Approaches

Study designs comprised 44% randomized controlled trials (RCTs), 28% mixed methods, 22% quantitative, and the remaining studies were qualitative. App-based technology was predominant in over 67% of the studies, while approximately 33% utilized SMS-based interventions.

Implementation Outcomes

**Table 1**<br>**Characteristics of the studies**							
**Author (year)**	**Study design**	**Study duration (months)**	**Country**	**Sample (N)**	**Intervention Type**	**Implementation Research Outcomes**	**Other outcomes (eg., client, service and clinical outcomes)**
Amausi et al., (2022)	Cross sectional - Quantitative study	9 months	Ghana	4000	**mHealth App**	Feasibility, Acceptability	Appropriateness
Bobrow, K., (2016)	RCT	5 months	South Africa	1372	**Short Message Service (SMS)**	Feasibility	Effectiveness
Cremers, A.L. et al.,(2019)	Mixed Methods study	13 months	Nigeria	30+328	**mHealth App**	Feasibility and Acceptability	
Dele-Ojo et al., (2023)	RCT	6 months	Ghana and Nigeria	225	**mHealth App**	Adoption	
Hacking,D.,et al (2016)	Mixed Methods Study	17 weeks	South Africa	223	**Short Message Service (SMS)**	Adoption and Feasibility	Effectiveness
KofiOwusu (2016)	Cohort - Quantitative	6 months	Ghana	150	**mHealth App** The model consisted of a mobile tablet, an automated blood pressure machine, and a novel software	Adoption	

					application for longitudinal patient management.		
Leon, N. et al., (2015)	RCT	12 months	South Africa	22	**Short Message Service (SMS)**	Acceptability and Adoption	
Mannik, J. et al., (2016)	Commu nity screenin g (qualitati ve research)	22 months	Rural Kenya	2865	**Short Message Service (SMS)**	Acceptability	Scalability
Mugabirw e, B. et al., (2021)	Cohort - Quantita tive non randomi zed	30 days	Uganda	37	**mHealth App**	Acceptability Feasibility	
Nelissen, H. E.et al., (2018)	Mixed Methods Study	6-8 months	Nigeria	336	**mHealth App**	Feasibility	
Nichols et al., (2017)	Mixed Method	Not mentioned	Ghana	200	**mHealth App**<br><br>Bluetooth enabled blood pressure monitor, smartphone app, and SMS	Acceptability, Feasibility	
Nichols, M. et al., (2019)	RCT	3 months	Ghana	16	**mHealth App**<br><br>Phone-based Intervention under Nurse Guidance (PINGS)	Acceptability	
Ola-Olorun, O. J. et al.,	Cross sectional study -	8 months	Nigeria	117	**Short Message Service (SMS)**	Acceptability	Patient-centeredne ss

(2012)	Quantitai ve						
Otu, A et al., (2022)	Mixed Methods	12 months	Nigeria	24	**mHealth App** mHealth training module (video & text based) delivered via android tablets and group support via Whatsapp	Feasibility	Scalability and Effectivene ss
Sarfo et al., (2018)	RCT	3 months	Ghana	60	**mHealth App-** Bluetooth enabled smartphone	Feasibility	Efficacy
Sarfo et al., (2019)	RCT	9 months	Ghana	60	**mHealth App** - Bluetooth link to blood pressure apparatus	Feasibility	
Sturt et al., (2023)	RCT	12 months	Nigeria and Tanzani a	20 clusters with 100 patients in each cluster	**mHealth App**	Acceptability and Feasibility	Appropriat eness
Vedantha n et al., (2019)	RCT	32 months	Western Kenya	1460	**Short Message Service (SMS)**	Acceptability	

Acceptability and Feasibility was the most common reported implementation outcome throughout all the studies. Approximately 61% of the 18 studies focused on feasibility, 56% on acceptability, 22% on adoption, 17% effectiveness, and 11% on appropriateness. Furthermore, approximately 11 % of the studies reported on scalability. The latter is defined and reported according to the WHO ExpandNet definition, as ‘deliberate efforts

to increase the impact of successfully tested health innovations, to benefit more people and to foster policy and program development on a lasting basis'.[17] None of the studies discussed fidelity, penetration and sustainability. In the collective analysis of the nine articles that evaluated acceptability, both participants and healthcare providers expressed noteworthy satisfaction with the mHealth intervention.

Most of the participants demonstrated a high level of comfort with the SMS reminders and mobile Apps utilized for blood pressure monitoring. Particularly, stroke patients and their caregivers exhibited an overwhelming positive response, citing the intervention's role in enhancing medication adherence, heightening awareness of blood pressure control, fostering improved dietary practices, and encouraging stress reduction.[18] The overall sentiment suggests a robust acceptance and appreciation for the mHealth strategies employed, affirming their positive impact on both patients and their caregivers. The eleven articles that evaluated feasibility revealed that the accessibility of mobile phones played a crucial role in the feasibility of adopting mHealth interventions. Additionally, aligning the intervention with accessible pharmacies or involving nurses in the triaging process emerged as factors that could further enhance feasibility. These findings underscore the importance of not only mobile phone accessibility but also strategic partnerships with healthcare facilities or task shifting to optimize the feasibility of mHealth interventions. The four studies assessing the adoption rates advocated for addition of mHealth to the usual care among patients with hypertension which would lead to an increase in the proportion of those with good blood pressure control.[19-22] Effectiveness, as evaluated by three studies, revealed that participants perceived the SMS messages positively. Rather than being seen as merely conveying new information, the SMS messages were

regarded as motivational reminders for promoting behavioral change. Participants described the SMS messages as "trustworthy" and "caring," creating a sense of being looked after, which contributed to their overall positive reception and effectiveness in encouraging behavioral adjustments.[20, 23, 24] A study measuring acceptability and patient centeredness revealed that their intervention garnered positive perceptions from the involved patients, and the assessment of patient-centeredness highlighted a strong inclination towards the chosen means of communication.[25] The respondents' attitudes in the study underscored a robust desire for this method of interaction.

Our findings affirm that the participants held a pronounced perception of the intervention's usefulness, demonstrating a favorable disposition towards its adoption in the realm of mHealth, a singular exploration of scalability led by Mannik, J. et al (2016)[26] stands out. This study, often overshadowed by other outcome assessments, uniquely delved into the scalability of a novel mHealth tool named AFYACHAT, derived from 'afya,' meaning health in Kiswahili (Kenya's national language). The research concluded that this innovative tool holds the potential to empower Community Health Workers (CHWs) in efficiently screening large numbers of patients in rural African communities for CVD risk. Impressively, the AFYACHAT system gained widespread acceptance among the target population. Notably, the study underscores the scalability of AFYACHAT, especially when leveraged in conjunction with the extensive reach of CHWs across rural Africa, suggesting its capacity for significant and far-reaching public health impact.

Similarly, in a study assessing the feasibility, acceptability, and appropriateness of a mHealth intervention from the perspective of health workers, an overwhelming majority

of health workers, particularly clinical staff, regarded PINGS (Phone-based Intervention under Nurse Guidance after Stroke) as feasible, acceptable, and suitable for blood pressure control among stroke survivors in Ghana.

The study further revealed that our findings contribute to the evidence that mHealth interventions for post-stroke care are implementable in resource-constrained settings.[27] Furthermore, another study emphasized the importance of task-shifting strategies and culturally tailored m-health interventions in enhancing post-stroke care in sub-Saharan Africa, highlighting their significance alongside feasibility considerations.[28]

Facilitators and Barriers to implement mHealth technology in Hypertension management in Africa

Table 2 presents our interpretations of various barriers and facilitators influencing the implementation of mHealth for hypertension management in the African context.

Table 2
Facilitators and Barriers to implement mHealth technology in Hypertension management in Africa

Facilitators	Description
1. **Growing Cell Phone Market in Africa**	Africa's rapidly expanding cell phone market, with over 75% ownership among the low-income South African population, facilitates the reach of SMS interventions.[11]
2. **Localization of SMS Messages**	SMS messages translated into local provincial languages potentially contributed to increased adoption. Personalization and tailoring of SMS messages to individual recipients show a marked improvement in outcomes.[18, 28]
3. **Mobile Phone-Based**	Implementation of adherence interventions with behavior change theories, considering the complex mix of

**Adherence Interventions**	psychosocial and health service influences on adherence behaviors.[29]
**4. Awareness of Chronic Nature of Hypertension**	Recognition of hypertension's chronic nature and the necessity of continuous drug intake, as well as maintaining a healthy lifestyle (healthy nutrition and physical activity).[30]
**5. Quality Training of Pharmacists**	Five-year training of pharmacists is of high quality, facilitating task shifting. Frequent booster training also helps with Knowledge retention.[25]
**6. Culturally Relevant and Cost-Effective Interventions**	Culturally tailored and relevant, cost-effective interventions built upon existing infrastructure for expediency, and sustainability. [18, 31]
**Barriers**	
**1. General Belief in Hypertension Symptoms**	General belief in symptoms of hypertension poses a barrier.[32]
**2. Rejection of Cheaper Local Hypertensive Drugs**	Rejection of cheaper local hypertensive drugs by some individuals.[32, 33]
**3. High Cost of Anti-hypertensive Drugs**	High cost of anti-hypertensive drugs acts as a barrier.[32]
**4. Technology-Related Concerns**	Some patients, especially older ones, may experience increased insecurity due to technology. - Unstable internet connection as a technological barrier.[20, 30, 34]
**5. Socioeconomic Disparities**	Differences in monthly income and education level contribute to disparities.[19]
**6. Proximity to Care Issues**	Proximity to care prevents patients from resorting to remote consultations.[35]
**7. Financial Limitations**	Financial constraints may hinder owning a cellphone or affording hypertensive medications.[19, 22]
**8. High Patient to Physician Ratios**	High patient to physician ratios can be addressed by substituting physicians with Community Health Workers

	(CHWs) and Pharmacists or Nurses for remote monitoring and intervention design. - CHWs and healthcare professionals play a key role in promoting medication adherence and behavior change through remote interventions. Task-shifting strategies can be employed. [28]

Quality Assessment

We employed the ASSESS tool (A Comprehensive Tool to Support Reporting and Critical Appraisal of Qualitative, Quantitative, and Mixed Methods Implementation Research Outcomes)[36] to evaluate the risk of bias in the study **(Table 3)**. Two authors individually assessed the risk of bias of each study that met the inclusion criteria. Conflicts were resolved via discussion. This tool comprises five criteria for each study design. Each criterion was used to assess the study, and an aggregate score was calculated to categorize studies into high or low bias. A score of 1-2 indicate high risk of bias and 3-5 indicate low risk of bias. Further, the PRISMA checklist was completed for this study.

Table 3: Risk of Bias Assessment		
**Article**	**Score**	**Degree of Bias**
Amuasi et al. (2022)	5	Low bias
Bobrow, K. et al. (2016)	5	Low bias
Cremers, A.L. et al (2019)	4	Low bias
Dele-Ojo et al. (2023)	5	Low bias
Hacking, D. et al (2016)	5	Low bias
KofiOwusu et al. (2016)	4	Low bias
Leon, N. (2015)	5	Low bias
Mannik, J. et al (2016)	5	Low bias
Mugabirwe, B. et al (2021)	4	Low bias
Nelissen, H. E. et al (2018)	4	Low bias
Nichols et al. (2017)	5	Low bias
Nichols, M. et al (2019)	5	Low bias
Ola-Olorun, O. J. et al (2012)	4	Low bias
Otu, A. et al. (2022)	4	Low Bias
Sarfo et al. (2018)	4	Low Bias
Sarfo et al. (2019)	4	Low Bias
Sturt et al. (2023)	5	Low Bias
Vedanthan et al (2019)	5	Low bias
Based on ASSESS tool by Ryan et al. 2022[36]		

5. Discussion

Our analysis of a diverse range of studies on mHealth interventions for hypertension in African countries has revealed significant trends and considerations. Notable insights have emerged from the analysis of these articles, shedding light on research conducted in urban and rural areas across Africa. We have grouped these trends under facilitators and barriers towards implementing mHealth interventions in Africa which are provided in **Table 2** (Facilitators and Barriers to implement mHealth technology in Hypertension management in Africa).

The studies reviewed provide valuable perspectives on the acceptability and feasibility of interventions utilizing mHealth Apps and SMS-based strategies*.* While evaluating acceptability, the focus was primarily on patients' attitudes toward receiving health information through SMS or call reminders. Notably, specific studies emphasized that SMS interventions delivered in the local languages garnered more favorable responses from the patient population.[22] Some studies also suggested unfamiliarity of the older population to technology which might pose a barrier to acceptability of intervention.[37] These results show the significance of cultural considerations in customizing mHealth interventions to enhance acceptability among the intended audience. It is essential to note that strategies utilizing SMS were highly effective in promoting positive patient behavioral changes, however, the translation of these self-reported behavioral changes into tangible improvements in blood pressure reduction remains to be observed.

Adoption can be defined as the intention, initial decision, or action to try or employ an innovation or evidence-based practice.[15] Elevated adoption rates signify the successful

integration of technology, enhancing its potential for widespread impact and opportunity for scale-up. Study conducted by Dele-Ojo et al.,[19] showed that the addition of mHealth to the usual care among patients with hypertension led to an increase in the proportion of those with good blood pressure control in the target population. In a study conducted by Kofi Owusu et al.,[21] there was a remarkable adoption of the Akoma Pa program, leading to a substantial reduction in blood pressure levels across the entire participant cohort. The most significant decrease in systolic blood pressure were observed among those with initially uncontrolled blood pressure. Notably, no participants withdrew prematurely, and compliance with completing weekly blood pressure assessments and scheduled in-person medical reviews remained consistently high throughout the 6-month follow-up period. The study reported an 82% improvement in overall health awareness among participants, and an impressive 95% expressed a desire to continue utilizing the program in the future. Studies done by Mugabirwe et al.,[32] showed an increase in adherence to medication.

Even with a meticulously designed mHealth intervention, obstacles may arise if external factors, such as unreliable network carrier services, which may hinder seamless implementation. A study by Hacking et al., [20] exemplifies this challenge, revealing that over 29% of SMS deliveries failed due to issues with the service provider. Sarfo et al., [34] also reported concerns regarding patients' ability to use the technology, comprehend the information conveyed, and noted inconsistencies in network connectivity. According to the Global System for Mobile Communications report, "The Mobile Economy Sub-Saharan Africa 2022," smartphones are rapidly gaining adoption, accounting for an average of 49% of connections in 2022 and predicted to reach 61% by 2025. [38]

However, this also implies that nearly 40% of people in Sub-Saharan Africa use basic cell phones or have no mobile phone access at all. This observation prompts questions about the need to consider external conditions that can significantly influence feasibility and ultimate success. In a study conducted by Mannik, J. et al (2016) [26] challenges arose because of power interruptions at the clinic where the central database was housed. Consequently, the system became intermittently unavailable when Community Health Workers (CHWs) attempted to send SMS messages. CHWs faced difficulties in texting swiftly, particularly during instances when a sizable group of patients awaited screening. To overcome technological limitations, CHWs innovatively addressed the issue by recording results and sending SMS messages at a later time. These kinds of innovations can be incorporated either into initial training of care providers or directly into the design of the mHealth intervention. No other study reported a reasonable alternative to failure of intervention delivery.

Targeted messaging aimed at addressing behavioral aspects can enhance the overall effectiveness of interventions. This theory is supported by studies conducted by Vedanthan et al [29] and Nelissen et al [39] which demonstrated improved linkage to care.

Our review has provided valuable insights into the positive impact of mHealth interventions on both patients and providers. However, it has also sparked a crucial discussion on the need for context-specific research that considers the diverse contexts prevalent throughout the entire African continent, which remains largely unexplored. This review represents the first attempt, to our knowledge, to assess implementation outcomes

specific to mHealth for hypertension management in Africa. This acknowledgment underscores the significant gap in research that this study seeks to address.

6. Limitations

Our systematic review has several limitations. Although we did not explicitly focus on hypertension management for patients with other comorbidities, we welcomed and considered the results obtained through our search. However, incorporating diseases prone to hypertension in our search strategy might have provided a more comprehensive perspective. The majority of the studies were conducted in urban settings, limiting the generalizability of the results to various resource-limited settings. Additionally, none of the studies addressed alternatives to intervention delivery failure, such as issues with SMS delivery or interruptions in App-based interventions. Anticipating and addressing these technological glitches during the landscape analysis is crucial to developing sustainable interventions suitable for scale-up.

7. Conclusion

Africa has the fastest-growing market for cell phone technology, and over 75% of the low-income population already owns or has access to a cell phone, particularly in countries such as Nigeria, Ghana, and South Africa. With this said, there are notable facilitators and barriers that would facilitate the upscale of evidence -based interventions that aim at reducing the burden of hypertension in Africa. Based on the result trends in the studies observed, there are emerging good practices that could be further explored through research such as the Patient-centered Care Model between Patients, Pharmacists and Cardiologists. Recognizing and understanding these unique factors may influence the

implementation and effectiveness of an intervention which is essential for its success. Future research should aim to bridge the urban-rural gap and explore innovative solutions to enhance the feasibility as well as sustainability of mHealth interventions for hypertension management across diverse African settings.

Appendix A

Search Strategy for mHealth Intervention

(Mobile Health OR mhealth OR Tele-Referral OR Tele Referral OR Tele-Referrals OR Virtual Medicine OR Telehealth OR eHealth OR Telemedicine OR Remote Consultation OR Mobile Application OR Portable Cellular Phone OR Portable Cellular Phones OR Mobile Apps OR Mobile App OR Portable Electronic Apps OR Portable Electronic App OR Portable Software App OR Portable Software Apps OR Portable Software Applications OR Portable Software Application OR Smartphone Apps OR Smartphone App OR Portable Electronic Application OR Portable Electronic Applications OR Smartphone Application OR Smartphone Applications OR Smartphones OR Smart Phones OR Smart Phone OR Mobile Phone OR Mobile Phones OR Cell Phones OR Cellular Phone OR Cellular Phones OR Internet Based Interventions)

Search Strategy for Hypertension and Hypertension Management

(High Blood Pressure OR High Blood Pressures OR Hypertension OR Primary Hypertension OR Primary Hypertension OR Essential Hypertension OR Essential Hypertensions OR Hypertension Management)

Search Strategy for Implementation and Service outcomes

(acceptability OR Patient Acceptance of Healthcare OR patient compliance OR adherence OR patient participation OR diffusion of innovation OR healthcare utilization OR adoption OR uptake OR empirically supported treatment OR appropriateness OR program evaluation OR program effectiveness OR benchmarking OR cost OR economics OR feasibility OR outcomes assessment OR process assessment OR scale OR measure OR fidelity OR integrity OR penetration OR program sustainability OR evidence-based

intervention OR evidence-based practice OR intervention)

Search Strategy for African Countries

(Africa OR Africans OR African Countries OR African Continent OR South of the Sahara Africa OR Sub Saharan Africa OR sub Saharan Africans OR Central Africa OR Central Africans OR Cameroon OR Cameroonian OR Cameroonians OR Central African Republic OR Central African OR Chad OR Chadians OR Chadian OR Congo OR Democratic Republic of Congo OR Congolese OR Equatorial Guinea OR Equatoguinean OR Equatorial Guinean OR Gabon OR Gabonese OR Gabonaise OR Sao Tome and Principe OR São Toméan OR Sao Tomean OR Santomean OR Eastern Africa OR Eastern Africans OR Burundi OR Burundian OR Burundians OR Comoros OR Comorian OR Djibouti OR Djiboutian OR Eritrea OR Eritrean OR Eritreans OR Ethiopia OR Ethiopian OR Ethiopians OR Kenya OR Kenyan OR Kenyans OR Madagascar OR Malagasy OR Rwanda OR Rwandan OR Rwandans OR Rwandese OR Seychelles OR Seychellois OR Seychelloise OR Seselwa OR Somalia OR Somali Or Somalis OR South Sudan OR South Sudanese OR Sudan OR Sudanese OR Tanzania OR Tanzanian OR Tanzanians OR Uganda OR Ugandans OR Southern Africa OR Angola OR Angolan OR Angolans OR Botswana OR Batswana OR Motswana OR Eswatini OR Swazi OR Emaswati OR Liswati OR Lesotho OR Mosotho OR Basotho OR Malawi OR Malawian OR Malawians OR Mozambique OR Mozambican OR Mozambicans OR Namibia OR Namibian OR Namibians OR South Africa OR South African OR South Africans OR Zambia OR Zambian OR Zambians OR Zimbabwe OR Zimbabwean OR Zimbo OR Western Africa OR Western Africans OR Benin OR Beninese OR Burkina Faso OR Burkinabè OR Cabo Verde OR Cape Verdean OR Cabo Verdean OR Cote d'Ivoire OR

Ivory Coast OR Republic of Côte d'Ivoire OR Ivorian OR Gambia OR Gambian OR Ghana OR Ghanaian OR Ghanaians OR Guinea OR Guinean OR Guineans OR Guinea-Bissau OR Republic of Guinea-Bissau OR Bissau-Guinean OR Guinea-Bissauan OR Liberia OR Republic of Liberia OR Liberian OR Mali OR Republic of Mali OR Malian OR Mauritania OR Islamic Republic of Mauritania OR Mauritanian OR Mauritanians OR Niger OR Nigerien OR Nigeriens OR Nigeria OR Nigerian OR Senegal OR Republic of Senegal OR Senegalese OR Sierra Leone OR Sierra Leonean OR Togo OR Togolese)

Acknowledgements

We are grateful to the members of the Implementing Sustainable Evidence-based interventions through Engagement (ISEE) Lab at New York University School of Global Public Health for their feedback and support on this paper.

References

1. *Low and Middle Income Countries*. 2022 [cited 2024 22nd March]; Available from: https://data.worldbank.org/income-level/low-and-middle-income?view=chart.
2. WHO. *A global brief on hypertension*. 2023 [cited 2024 22nd March]; Available from: https://www.who.int/publications/i/item/9789240081062.
3. Flack, J.M. and B. Adekola, *Blood pressure and the new ACC/AHA hypertension guidelines.* Trends Cardiovasc Med, 2020. **30**(3): p. 160-164.
4. Mills, K.T., A. Stefanescu, and J. He, *The global epidemiology of hypertension.* Nat Rev Nephrol, 2020. **16**(4): p. 223-237.
5. Nichols, M., et al., *Assessing Mobile Health Capacity and Task Shifting Strategies to Improve Hypertension Among Ghanaian Stroke Survivors[white star],[white star][white star].* Am J Med Sci, 2017. **354**(6): p. 573-580.
6. Duley, L., *Pre-eclampsia, eclampsia, and hypertension.* BMJ Clin Evid, 2008. **2008**.
7. Xia, T., F. Zhao, and R.A. Nianogo, *Interventions in hypertension: systematic review and meta-analysis of natural and quasi-experiments.* Clin Hypertens, 2022. **28**(1): p. 13.
8. Jeemon, P., et al., *World Heart Federation Roadmap for Hypertension - A 2021 Update.* Glob Heart, 2021. **16**(1): p. 63.
9. Zakerabasali, S., et al., *Mobile Health Technology and Healthcare Providers: Systemic Barriers to Adoption.* Healthc Inform Res, 2021. **27**(4): p. 267-278.
10. Adebowale, I.O., *mHealth Interventions in South Africa: A Review.* SAGE Open, 2018. **8**.
11. Taylor, P., *Forecast number of mobile users worldwide 2020-2025*.
12. Beratarrechea, A., et al., *The impact of mobile health interventions on chronic disease outcomes in developing countries: a systematic review.* Telemed J E Health, 2014. **20**(1): p. 75-82.
13. Gupta, T., *Mobilising Africa: the growing role of mobile phone technology in healthcare.* Africa Health, 2014. **36**(4): p. 27-29.
14. Azevedo, M.J., *The State of Health System(s) in Africa: Challenges and Opportunities.* Historical Perspectives on the State of Health and Health Systems in Africa, Volume II, 2017: p. 1-73.
15. Proctor, E., et al., *Outcomes for implementation research: conceptual distinctions, measurement challenges, and research agenda.* Adm Policy Ment Health, 2011. **38**(2): p. 65-76.
16. Page, M.J., et al., *PRISMA 2020 explanation and elaboration: updated guidance and exemplars for reporting systematic reviews.* bmj, 2021. **372**.
17. WHO, *Nine steps for developing a scaling-up strategy*, W.H.O. ExpandNet, Editor. 20 September 2010, World Health Organization. p. 41.
18. Sarfo, F.S., et al., *Phone-based intervention for blood pressure control among Ghanaian stroke survivors: A pilot randomized controlled trial.* International Journal of Stroke, 2019. **14**(6): p. 630-638.
19. Dele-Ojo, B.F., et al., *The effect of mobile health technology on blood pressure control among patients with hypertension in Ghana and Nigeria.* Research square, 2023.

20. Hacking, D., et al., *Hypertension Health Promotion via Text Messaging at a Community Health Center in South Africa: A Mixed Methods Study.* JMIR Mhealth Uhealth, 2016. **4**(1): p. e22.
21. KofiOwusu, I., et al., *Novel hypertension management model of care decreases blood pressure in West African population.* Journal of Hypertension, 2016. **34**(Supplement 2): p. e305.
22. Leon, N., et al., *Improving treatment adherence for blood pressure lowering via mobile phone SMS-messages in South Africa: a qualitative evaluation of the SMS-text Adherence SuppoRt (StAR) trial.* BMC family practice, 2015. **16**: p. 80.
23. Bobrow, K., et al., *Mobile Phone Text Messages to Support Treatment Adherence in Adults With High Blood Pressure (SMS-Text Adherence Support [StAR]): a Single-Blind, Randomized Trial.* Circulation, 2016. **133**(6): p. 592.
24. Otu, A.A., et al., *mHealth guideline training for non-communicable diseases in primary care facilities in Nigeria: a mixed methods pilot study.* BMJ open, 2022. **12**(8): p. e060304.
25. Ola-Olorun, O.J., et al., *Exploring medicine information needs of hypertensive patients using short message service (SMS) of mobilie phone.* Pharmacoepidemiology and Drug Safety, 2012. **21**(1): p. 116.
26. Mannik, J., et al., *Community-based screening for cardiovascular risk using a novel mobile health (mhealth) technology in rural Kenya.* American Journal of Tropical Medicine and Hygiene, 2016. **95**(5 Supplement 1): p. 63-64.
27. Amuasi, J., et al., *Feasibility, acceptability, and appropriateness of a mobile health stroke intervention among Ghanaian health workers.* J Neurol Sci, 2022. **439**: p. 120304.
28. Sarfo, F., et al., *PINGS (Phone-Based Intervention Under Nurse Guidance After Stroke): Interim Results of a Pilot Randomized Controlled Trial.* Stroke, 2018. **49**(1): p. 236-239.
29. Vedanthan, R., et al., *Community Health Workers Improve Linkage to Hypertension Care in Western Kenya.* Journal of the American College of Cardiology, 2019. **74**(15): p. 1897.
30. Nichols, M., et al., *Post-intervention qualitative assessment of mobile health technology to manage hypertension among Ghanaian stroke survivors.* Journal of the neurological sciences, 2019. **406**: p. 116462.
31. Ochieng, B.O., et al., *Community-based screening for cardiovascular risk using a novel mobile health (mhealth) technology in rural Kenya.* American Journal of Tropical Medicine and Hygiene, 2016. **95**(5 Supplement 1): p. 63-64.
32. Mugabirwe, B., et al., *Acceptability and feasibility of a mobile health application for blood pressure monitoring in rural Uganda.* JAMIA Open, 2021. **4**(3): p. ooaa068.
33. Bobrow, K., et al., *Mobile Phone Text Messages to Support Treatment Adherence in Adults With High Blood Pressure (SMS-Text Adherence Support [StAR]): A Single-Blind, Randomized Trial.* Circulation, 2016. **133**(6): p. 592-600.
34. Sarfo, F.S., et al., *Phone-based intervention for blood pressure control among Ghanaian stroke survivors: a pilot randomized controlled trial.* International journal of stroke, 2019. **14**(6): p. 630.
35. Sturt, J., et al., *Safety and upscaling of remote consulting for long-term conditions in primary health care in Nigeria and Tanzania (REaCH trials): stepped-wedge*

trials of training, mobile data allowance, and implementation. Lancet Glob Health, 2023. **11**(11): p. e1753-e1764.
36. Ryan, N., et al., *Development of the ASSESS tool: a comprehensive tool to Support rEporting and critical appraiSal of qualitative, quantitative, and mixed methods implementation reSearch outcomes.* Implementation Science Communications, 2022. **3**(1): p. 34.
37. Cremers, A., et al., *Patients' and healthcare providers' perceptions and practices regarding hypertension, pharmacy-based care, and mHealth in Lagos, Nigeria: a mixed methods study.* J Hypertens, 2019. **37**(2): p. 389-397.
38. Rennie, S., et al., *Public health research using cell phone derived mobility data in sub-Saharan Africa: Ethical issues.* South African Journal of Science, 2023. **119**: p. 38-44.
39. Nelissen, H.E., et al., *Pharmacy-based hypertension care employing mHealth in Lagos, Nigeria - a mixed methods feasibility study.* BMC Health Serv Res, 2018. **18**(1): p. 934.

Figure Click here to access/download;Figure;Figure 1.docx

Figure 1

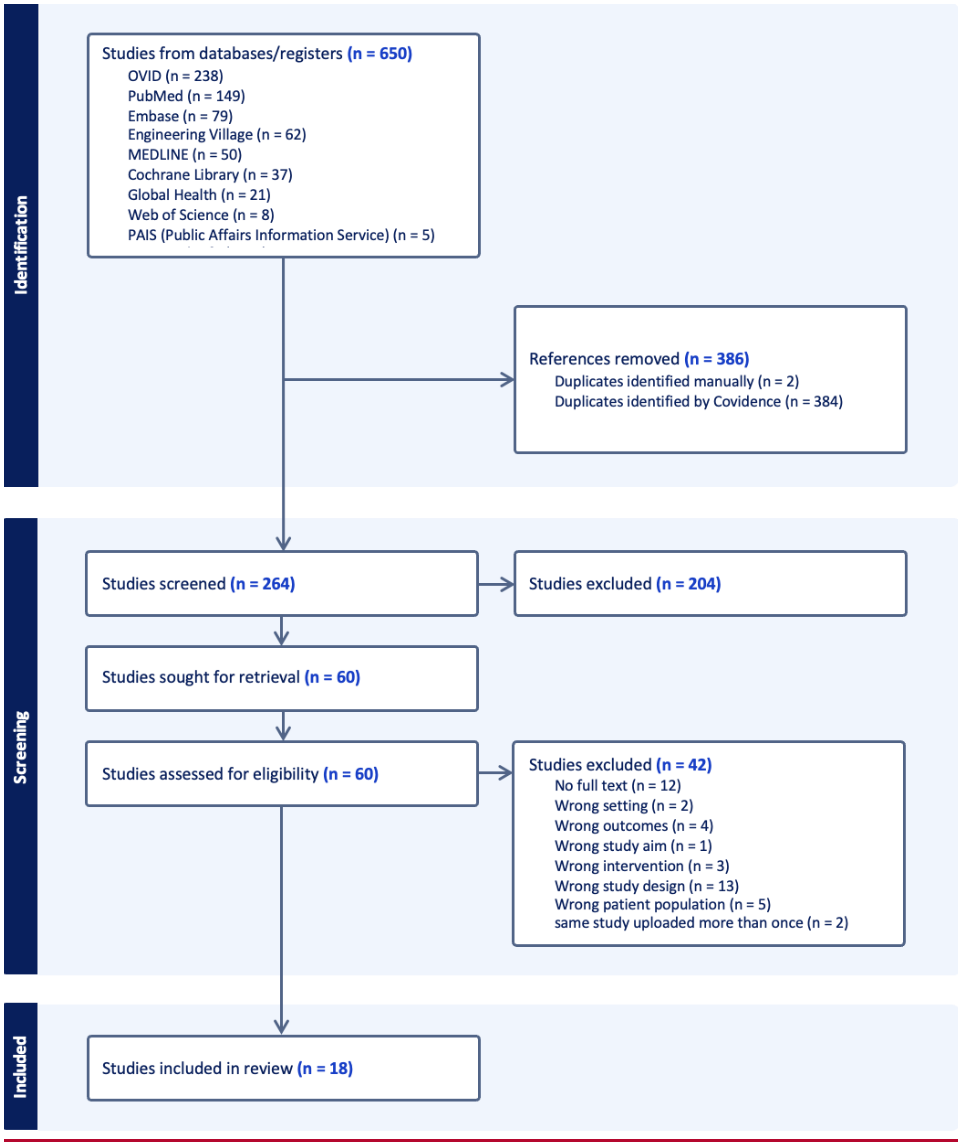

PRISMA Checklist

Click here to access/download
Supporting Information
PRISMA checklist.docx
