# Supplementary material for: Mobile health interventions for African adults with hypertension: Evidence from a systematic review of implementation research outcomes
Source: PLOS Digit Health. 2026 Aug 14;5(8):e0001612. doi: 10.1371/journal.pdig.0001612 (PMC13475905; doi:10.1371/journal.pdig.0001612)
Supplement: Supplementary file 3 [file pdig.0001612.s016.pdf]

Date: February 12th, 2025

Manuscript title: Determining Acceptability, Feasibility, and Adoption rates associated with Mobile Health interventions for hypertension management among adults in Africa - A Systematic Review

Manuscript number: PDIG-D-24-00124R1

Dear Editor,

We sincerely appreciate the reviewers' invaluable feedback on our manuscript. Their insightful comments have significantly contributed to improving the quality of our work. We have carefully addressed each of the reviewers' suggestions, resulting in enhanced clarity and coherence throughout the manuscript.

Below, we have provided a detailed response to the specific points raised by the reviewers.

Thank you for considering our revisions. We look forward to your feedback.

Best regards,

Priyanka Lanka

Master of Public Health Candidate
NYU School of Global Public Health
pl2461@nyu.edu

1. **A numbered table of all studies identified in the literature search, including those that were excluded from the analyses.**

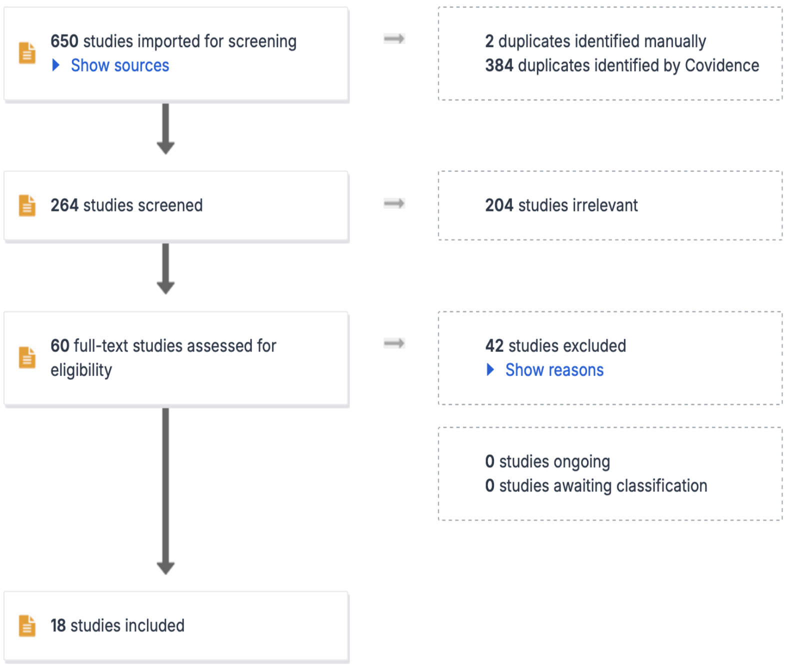

Response to the Editors: We provided two supplementary documents to support our systematic review submission, containing a PDF with the list of all 650 articles used in the systematic review and an excel file containing excluded articles. A description of each attachment is below. We hope these additional materials meet your expectations and provide clarity on our review methodology. Please do not hesitate to reach out if further information is required.

- **PDF Document:** This file contains a comprehensive list of all 650 articles included in the review, clearly detailing the foundation of our analysis. **(Ref file titled "All articles ")**
- **Excel File**: This file outlines the articles that were excluded during the review process, along with relevant exclusion criteria where applicable. **(Ref file titled "Excluded and Irrelevant Articles.xlsx")**

2. **For every excluded study, the table should list the reason(s) for exclusion.**

Response to the Editors: We sincerely thank the reviewer for their thorough and insightful assessment of our manuscript. As outlined in the methods section, articles were excluded from the review solely because they did not meet the predefined inclusion criteria. We ensured that this rigorous screening process was conducted systematically to maintain the quality and relevance of our findings.

To provide additional transparency, our PRISMA (Preferred Reporting Items for Systematic Reviews and Meta-Analyses) flowchart clearly details the number of articles excluded at each stage of the review process. Following the reviewers' request, we have also included comprehensive supplementary materials:

- **Included Articles:** A complete list of the 650 articles that formed the basis of our systematic review.
- **Excluded Articles:** Documentation outlining the articles removed during screening, along with the corresponding reasons for exclusion, as aligned with PRISMA guidelines.

We hope that these materials enhance the clarity and robustness of our submission. Please let us know if any further information or clarification is required.

3. **If any of the included studies are unpublished, include a link (URL) to the primary source or detailed information about how the content can be accessed.**

Response to the Editors: We would like to confirm that all studies included in our systematic review are published and have undergone rigorous peer review in reputable journals, ensuring the reliability and quality of the evidence synthesized.

In line with the reviewers' request, we have provided comprehensive supplemental materials for greater transparency:

- Included Studies: A PDF file containing a detailed list of all 650 publications incorporated in the review, along with relevant citation information.
- Excluded Studies: An Excel file documenting articles excluded during various phases of the screening process, accompanied by exclusion criteria in accordance with PRISMA guidelines.

We hope this additional information offers clarity and supports a better understanding of our systematic review process. Please feel free to reach out if further details are needed.

4. **A table of all data extracted from the primary research sources for the systematic review and/or meta-analysis. The table must include the following information for each study:**

A. Name of data extractors and date of data extraction

Response to Editors: We appreciate the opportunity to provide further clarification and supporting materials for our submission. As requested, we have attached an Excel file containing the comprehensive data extraction findings from all 18 articles included in the review. This file provides detailed information on key study characteristics, outcomes, and relevant data points essential for understanding our synthesis process.

Additionally, to ensure transparency and accountability, we have included the name of the individual responsible for the data extraction process. This ensures full traceability and supports the integrity of the review.

We trust that this supplemental material will enhance the clarity and completeness of our manuscript. Please do not hesitate to reach out if additional information or clarification is required.

(Ref file titled "Final Data Extraction table")

Response to the Editors: Thank you for the opportunity to provide additional information regarding the data extraction timeline. As requested, we have included a screenshot from Covidence, which visually displays the timeline associated with the extraction process. This material can be found in the folder titled ***"Screenshots Displaying Dates of Extraction."***

Unfortunately, Covidence does not currently support the direct download of specific extraction dates as a standalone report. However, the provided screenshot captures the timeline of the key extraction activities conducted during the review process, offering a transparent view of our workflow and adherence to systematic review protocols.

We hope this supplemental material meets your requirements. Please feel free to reach out for any additional information or clarification.

B. Confirmation that the study was eligible to be included in the review.

Response to the Editors:: We appreciate the opportunity to provide additional clarification regarding the eligibility of studies included in our systematic review. We confirm that all studies met the inclusion criteria outlined in the Methods section, ensuring their relevance and appropriateness for addressing the review objectives.

To enhance transparency, we have provided supplementary materials that include:

- **Included Articles:** An Excel file titled ***"Included Articles"*** that lists all studies incorporated in the final synthesis.

- **PRISMA Flowchart:** A detailed visual representation highlighting the number of articles included and excluded at each stage of the systematic review process, in accordance with PRISMA guidelines.

These supplemental materials are intended to offer a clearer understanding of our selection process and the rigor applied during the review. Please do not hesitate to reach out if any further details or clarifications are required.

C. All data extracted from each study for the reported systematic review and/or meta-analysis that would be needed to replicate your analyses.

Response to the Editors:: We appreciate the opportunity to provide additional information in support of our manuscript. As requested, we have included supplementary material in the form of an Excel file titled ***"Final Data Extraction Table."***

This document contains detailed extracted data from each of the articles included in our review. The table captures key study characteristics, outcome measures, and relevant data points essential to understanding the synthesis process and findings. By providing this file, we aim to ensure full transparency regarding the evidence base and data collection efforts underpinning our analysis.

We hope this supplemental material meets your expectations and provides valuable insights into the data extraction process. Please feel free to reach out if further details are needed.

5. If data or supporting information were obtained from another source (e.g. correspondence with the author of the original research article), please provide the source of data and dates on which the data/information were obtained by your research group.

Response to the Editors:: We confirm that no data was obtained from any sources outside of the ones explicitly mentioned in our manuscript. All data utilized in the study were sourced exclusively from the identified datasets, and no additional or external data was incorporated at any stage of the research process. We hope this clarification addresses your concern, and please feel free to reach out if further information is needed.

6. If applicable for your analysis, a table showing the completed risk of bias and quality/certainty assessments for each study or outcome. Please ensure this is provided for each domain or parameter assessed. For example, if you used the

Cochrane risk-of-bias tool for randomized trials, provide answers to each of the signalling questions for each study. If you used GRADE to assess certainty of evidence, provide judgements about each of the quality of evidence factor. This should be provided for each outcome.

Response to the Editors:: In response to your request, we have included a table within the manuscript that outlines the completed risk of bias and quality/certainty assessments for each study included in the systematic review. This table is designed to provide a clear overview of the evaluations conducted for each study, and we have made sure that it covers all relevant domains or parameters assessed during the review process.

Furthermore, we have utilized the ASSESS tool to facilitate our evaluations, and we have attached the corresponding Excel sheet titled 'ASSESS - Risk of Bias Assessment' for your reference. The ASSESS tool also includes a section for assessing the certainty of evidence, which allows us to provide detailed judgments for each factor related to the quality of evidence. As requested, these judgments are presented for each individual outcome evaluated in the review.

For additional context, the ASSESS tool is a well-established and widely recognized resource, and it was published in *Implementation Science Communications*. It is available via the EQUATOR Network, and is specifically designed to evaluate and report on implementation science research. The tool can be used to assess the risk of bias in implementation outcomes, as well as to describe studies that employ qualitative, quantitative, or mixed methods. It provides a structured and transparent approach to assessing the quality of evidence across various study designs, helping to ensure consistency in the evaluation process.

We trust that this provides the necessary information for your analysis, and we are happy to offer further clarification if needed.

7. An explanation of how missing data were handled.

Response to the Editors: Thank you for your inquiry regarding how missing data were handled in the study. In cases where data were unavailable or not provided, we ensured that 'Not Recorded' (NR) was added in the relevant fields to indicate the absence of data. This approach was employed wherever necessary, in order to maintain transparency and to provide a clear record of the data available for each variable assessed. By marking these instances as 'NR,' we aimed to ensure that readers could distinguish between genuinely missing data and fields where data were not applicable or not yet collected.

Moreover, this method was consistently applied across all studies and was carefully documented to avoid any ambiguity in the presentation of results. In line with best practices, we took steps to minimize the potential impact of missing data on our analysis. Additionally, any assumptions made regarding missing data were clearly stated in the methodology section to ensure full transparency. We believe that by handling missing data in this manner, we have adhered to established guidelines for data integrity and reporting, minimizing the risk of bias or misinterpretation.

8. Reviewer 2 Comment 1: The authors have adequately addressed the prior critiques

Author Response to Review 2 Comment 1: We thank the reviewer for this comment

9. Comment 1: I suggest including a table of Procter's feasibility outcomes with the definitions to aid reviewers not familiar with the lexicon.

Author Response to Review 3 Comment 1:: We would like to sincerely thank the reviewer for this insightful comment. In response, we have included the definitions of Proctor’s implementation outcomes, as well as the service outcomes, to provide greater clarity and ensure consistency throughout the manuscript. These definitions are now presented in detail under Table 1 and Table 2, respectively, so that readers can easily reference the key concepts related to these outcomes. We believe this addition will help enhance the understanding of the terms used and strengthen the overall presentation of the outcomes in the context of our study. We appreciate the reviewer’s attention to this matter and are grateful for the opportunity to improve the manuscript.

10. **Comment 2: Page 21 Table 2, please review text in certain rows and clarify what you mean. For instance, the first barrier merely states: "General belief in symptoms of hypertension poses a barrier [32]." The third facilitator: Implementation of adherence interventions with behavior change theories, considering the complex mix of psychosocial and health service influences on adherence behaviors [28].**

Author Response to Review 3 Comment 2:: Thank you for your comment. The passage in question was directly quoted from the referenced article. However, after reviewing the feedback, we realized that the author's intended message could benefit from further clarification. To improve understanding, we expanded upon the original quote to provide additional context and enhance its clarity. The revised version now reads as follows:

“Emotional distress along with fear and frustration from seeing consistently high blood pressure readings posed as a barrier and reduced engagement with the intervention. [32]”'

Additionally, the original passage discussing the effectiveness of mobile phone-based adherence interventions has been clarified to emphasize the role of behavior change theories in improving long-term adherence. The updated version now reads:

“Implementation of mobile phone-based adherence interventions grounded in established behavior change theories can be more effective in promoting long-term adherence to treatment. [23]”

We believe these revisions will help readers gain a deeper understanding of the challenges faced by participants and the potential strategies for improving intervention outcomes. Thank you again for the valuable feedback.

11. Comment 11: My main comment is that the discussion still requires revision to enhance the value of the manuscript. Specifically, the organizational framework needs some work to make the manuscript useful to the audience. For instance, page 25 Par 1 "Hence, incorporating educational components to address these beliefs are necessary, as it directly impacts the feasibility and effectiveness of implementing mHealth solutions to address this condition" is quite out of place in the flow of the discussion.

Author Response to Review 3 Comment 3: We would like to sincerely thank Reviewer 3 for their thoughtful and constructive comment regarding the need for further revision of the discussion to enhance the overall value of the manuscript. We fully appreciate the feedback that the organizational framework requires refinement to ensure that the manuscript is more useful and accessible to the intended audience. In response to this important concern, we have undertaken a significant restructuring of the discussion section.

To improve the clarity and flow, we have reorganized the content to ensure a more logical progression of ideas. Specifically, we have addressed the suggestion regarding the placement of the sentence about the educational components aimed at addressing beliefs about hypertension. We have moved this sentence to a more appropriate location within the discussion of patient-related barriers, where it now fits seamlessly with the broader context of the challenges patients face in managing hypertension. This revision not only improves the coherence of the discussion but also helps to emphasize

the importance of targeted educational interventions as a key strategy in overcoming these barriers.

We believe these revisions significantly improve the readability of the manuscript and align with the valuable feedback provided. The overall structure of the discussion is now more focused and organized, ensuring that each point is introduced and explored in a logical sequence. We hope this restructuring enhances the manuscript's utility to the audience, providing clearer insights into the key findings and implications.

Once again, we would like to thank Reviewer 3 for their insightful feedback, which has greatly contributed to the strengthening of this section.

12. Comment 4: Table 1 might be a better summary if the findings of the studies were summarized instead of merely stating which outcomes were studied (e. feasibility).

Author Response to Review 3 Comment 4: "Thank you for your comment and for taking the time to review the table in question. The primary purpose of the table is to succinctly summarize the key characteristics of the included studies, with a particular focus on implementation outcomes such as accessibility, feasibility, and adoption. Additionally, the table provides an overview of the geographical location and the type of mHealth intervention employed across the studies. This concise presentation allows readers to easily grasp the essential aspects of each study without being overwhelmed by excessive detail.

We then used the data from the table to quantify and analyze these key outcomes in the 'Characteristics of Identified Studies' section (Page 13), providing further context and insight into the broader trends observed across the studies. While we recognize the importance of the detailed findings, we believe that including them directly within the table would unnecessarily complicate its structure and detract from its core objective of offering a high-level summary.

To ensure a comprehensive presentation of the findings, we have instead categorized the facilitators and barriers identified across the studies within the Discussion section. Here, we provide a more in-depth analysis of these factors, offering specific examples from the studies along with quantitative data where available. This approach allows for a clearer, more focused discussion of the factors that influence the success of mHealth interventions, without overloading the table with excessive information.

We hope this explanation clarifies the rationale behind the organization of the table and the placement of detailed findings in the Discussion section. Thank you again for your thoughtful feedback, which has helped us refine our approach.
