# Supplementary material for: Mobile health interventions for African adults with hypertension: Evidence from a systematic review of implementation research outcomes
Source: PLOS Digit Health. 2026 Aug 14;5(8):e0001612. doi: 10.1371/journal.pdig.0001612 (PMC13475905; doi:10.1371/journal.pdig.0001612)
Supplement: Supplementary file 6 [file pdig.0001612.s006.docx]

**Appendix A**

**Search Strategy for mHealth Intervention**

(Mobile Health OR mhealth OR Tele-Referral OR Tele Referral OR Tele-Referrals OR Virtual Medicine OR Telehealth OR eHealth OR Telemedicine OR Remote Consultation OR Mobile Application OR Portable Cellular Phone OR Portable Cellular Phones OR Mobile Apps OR Mobile App OR Portable Electronic Apps OR Portable Electronic App OR Portable Software App OR Portable Software Apps OR Portable Software Applications OR Portable Software Application OR Smartphone Apps OR Smartphone App OR Portable Electronic Application OR Portable Electronic Applications OR Smartphone Application OR Smartphone Applications OR Smartphones OR Smart Phones OR Smart Phone OR Mobile Phone OR Mobile Phones OR Cell Phones OR Cellular Phone OR Cellular Phones OR Internet Based Interventions)

**Search Strategy for Hypertension and Hypertension Management**

(High Blood Pressure OR High Blood Pressures OR Hypertension OR Primary Hypertension OR Primary Hypertension OR Essential Hypertension OR Essential Hypertensions OR Hypertension Management)

**Search Strategy for Implementation and Service outcomes**

(acceptability OR Patient Acceptance of Healthcare OR patient compliance OR adherence OR patient participation OR diffusion of innovation OR healthcare utilization OR adoption OR uptake OR empirically supported treatment OR appropriateness OR program evaluation OR program effectiveness OR benchmarking OR cost OR economics OR feasibility OR outcomes assessment OR process assessment OR scale OR measure OR fidelity OR integrity OR penetration OR program sustainability OR evidence-based intervention OR evidence-based practice OR intervention)

**Search Strategy for African Countries**

(Africa OR Africans OR African Countries OR African Continent OR South of the Sahara Africa OR Sub Saharan Africa OR sub Saharan Africans OR Central Africa OR Central Africans OR Cameroon OR Cameroonian OR Cameroonians OR Central African Republic OR Central African OR Chad OR Chadians OR Chadian OR Congo OR Democratic Republic of Congo OR Congolese OR Equatorial Guinea OR Equatoguinean OR Equatorial Guinean OR Gabon OR Gabonese OR Gabonaise OR Sao Tome and Principe OR São Toméan OR Sao Tomean OR Santomean OR Eastern Africa OR Eastern Africans OR Burundi OR Burundian OR Burundians OR Comoros OR Comorian OR Djibouti OR Djiboutian OR Eritrea OR Eritrean OR Eritreans OR Ethiopia OR Ethiopian OR Ethiopians OR Kenya OR Kenyan OR Kenyans OR Madagascar OR Malagasy OR Rwanda OR Rwandan OR Rwandans OR Rwandese OR Seychelles OR Seychellois OR Seychelloise OR Seselwa OR Somalia OR Somali Or Somalis OR South Sudan OR South Sudanese OR Sudan OR Sudanese OR Tanzania OR Tanzanian OR Tanzanians OR Uganda OR Ugandans OR Southern Africa OR Angola OR Angolan OR Angolans OR Botswana OR Batswana OR Motswana OR Eswatini OR Swazi OR Emaswati OR Liswati OR Lesotho OR Mosotho OR Basotho OR Malawi OR Malawian OR Malawians OR Mozambique OR Mozambican OR Mozambicans OR Namibia OR Namibian OR Namibians OR South Africa OR South African OR South Africans OR Zambia OR Zambian OR Zambians OR Zimbabwe OR Zimbabwean OR Zimbo OR Western Africa OR Western Africans OR Benin OR Beninese OR Burkina Faso OR Burkinabè OR Cabo Verde OR Cape Verdean OR Cabo Verdean OR Cote d'Ivoire OR Ivory Coast OR Republic of Côte d'Ivoire OR Ivorian OR Gambia OR Gambian OR Ghana OR Ghanaian OR Ghanaians OR Guinea OR Guinean OR Guineans OR Guinea-Bissau OR Republic of Guinea-Bissau OR Bissau-Guinean OR Guinea-Bissauan OR Liberia OR Republic of Liberia OR Liberian OR Mali OR Republic of Mali OR Malian OR Mauritania OR Islamic Republic of Mauritania OR Mauritanian OR Mauritanians OR Niger OR Nigerien OR Nigeriens OR Nigeria OR Nigerian OR Senegal OR Republic of Senegal OR Senegalese OR Sierra Leone OR Sierra Leonean OR Togo OR Togolese)
