# Supplementary material for: Mobile health interventions for African adults with hypertension: Evidence from a systematic review of implementation research outcomes
Source: PLOS Digit Health. 2026 Aug 14;5(8):e0001612. doi: 10.1371/journal.pdig.0001612 (PMC13475905; doi:10.1371/journal.pdig.0001612)
Supplement: Supplementary file 14 [file pdig.0001612.s021.docx]

**Response to the Editors**

Dear Editors,

We sincerely thank you and the reviewers for the continued time and careful consideration given to our manuscript. The thoughtful feedback provided throughout the review process has been valuable in helping us further refine and strengthen the manuscript.

In this revised submission, we have carefully considered each of the reviewer comments and provided detailed, point-by-point responses. The manuscript has been revised accordingly, with careful attention to addressing the concerns raised and improving the overall clarity and coherence of the work.

We are pleased to resubmit the revised manuscript for your consideration. We greatly appreciate the time and effort invested by the editors and reviewers throughout this process and hope that the current revisions have satisfactorily addressed the remaining concerns.

Sincerely,

Dr Priyanka Lanka, MDS, MPH

NYU School of Global Public Health

[pl2461@nyu.edu](mailto:pl2461@nyu.edu)

**Reviewer #6**: I thank the authors for the revisions made in response to the previous round of review. The manuscript has been improved substantially, including adding inter-rater agreement reporting, clearer description of the consensus adjudication process, description of the distribution of studies by setting, and acknowledgement of the limitations of the studies in terms of rural representation, short-to-medium follow-up, and lack of assessment of sustained implementation.

**At this point, I find the manuscript borderline acceptable from a methodological perspective. I can only offer the following methodological refinements for final approval:**

1. **Clarify whether the Proctor framework was an inclusion criteria or an analytic classification framework.**

Current methods suggest that studies were included if they ‘used Proctor’s taxonomy’. Methodologically, this should be noted because relevant hypertension mHealth studies might have been excluded if the studies had to use Proctor’s framework explicitly. The authors should clarify if eligible studies were required to report Proctor’s taxonomy or if the review team mapped reported outcomes to Proctor’s implementation outcomes during extraction.

Improve the interpretation of low and variable inter-rater agreement.

**Response to Reviewer #6 Comment 1:**

We thank the reviewer for this important methodological distinction. To clarify: Proctor’s taxonomy functioned as an analytic classification framework applied by the review team during data extraction. We provided our specific search terms used in this SR on page 9 , line 192-207, and our inclusion and exclusion criteria in our methods section.

During extraction, the review team systematically mapped each study’s reported outcomes onto Proctor’s eight implementation outcome categories.

2. The manuscript now reports Cohen’s kappa and raw agreement, as it should. However, as the **reported kappa values are low and variable**, authors should briefly describe the impact on methodological confidence in the screening process and how consensus adjudication protected the validity of final inclusion decisions. **No new analysis is needed**, just a more transparent methodological interpretation of the implications of low agreement.

**Response Reviewer #6 Comment 2:**

These values likely reflect the high exclusion rate in this review, which can artificially depress kappa even when reviewers are making broadly concordant decisions.

The review team implemented a structured consensus adjudication process to protect the validity of final inclusion decisions: all disagreements between primary reviewers were flagged within Covidence and resolved through discussion, with a third reviewer providing adjudication where consensus was not reached. This process ensured that no article was included or excluded based on a single reviewer’s judgement, and that borderline cases received additional scrutiny. We have added a clarifying statement to the Methods section as such “Low kappa values indicate that the screening process used a consensus mechanism in addition to independent reviewer agreement. We do not believe this undermines the validity of the included studies, as the full-text review stage was characterized by greater reviewer consistency.”

3. **Improve the connection between implementation outcomes and clinical relevance at the level of the study.**

As the review is about hypertension management, implementation outcomes (eg, acceptability, adoption and feasibility) should be interpreted in the context of available clinical indicators (eg, blood pressure control, treatment adherence, follow-up attendance or hypertension knowledge). The authors should not be expected to conduct a meta-analysis, but a brief synthesis of the studies that reported clinical indicators and whether these outcomes supported or limited the interpretation of implementation success should be included.

**Response Reviewer #6 Comment 3:**

Although this manuscript objective was to identify implementation outcomes, we agree that situating implementation outcomes within their clinical context strengthens the interpretive value of the review. A synthesis of clinical indicators across the included studies is now provided as such in the *Results section (line 367 – 381)* “Of the 18 included studies, a subset reported clinical outcomes alongside implementation outcomes. Dele-Ojo et al. (2023) found that mHealth integration with usual care produced a significant increase in blood pressure control, directly supporting the adoption findings. Sarfo et al. (2018, 2019) reported improvements in blood pressure levels among stroke survivors in their PINGS RCTs, lending clinical weight to the feasibility and acceptability data documented in those studies. Bobrow et al. (2016) demonstrated that SMS-based interventions reduced systolic blood pressure compared to control, underlying the feasibility findings from that trial. Kofi Owusu et al. (2016) reported a significant decrease in systolic blood pressure and an 82% improvement in health awareness, reinforcing the adoption evidence. In contrast, studies that reported implementation challenges—particularly around network connectivity and technology literacy (Sarfo et al., 2019; Cremers et al., 2019)—did not consistently demonstrate clinical improvements, suggesting that feasibility barriers may have limited clinical impact. Studies that reported only qualitative or descriptive implementation data without clinical endpoints (e.g., Nichols et al., 2017; Mannik et al., 2016) limited authors ability to conclude whether high acceptability translated to clinical benefit.” We have also added the following synthesis to the *Discussion section (line 460-462)*: “Overall, where clinical indicators were reported alongside positive implementation outcomes, the evidence was broadly supportive; however, many studies lacked clinical outcome data, limiting the extent to which implementation success can be interpreted as clinically meaningful.”

4. **Barriers and Facilitators Prioritization: Barriers are ranked by severity. Facilitators are ranked by impact.**

The manuscript identifies a few barriers and facilitators, but the applied value of these would be increased if they were organized by frequency, recurrence across studies, and potential impact on implementation. If the evidence does not allow this it should not be presented as a formal weighted ranking.

For example, a descriptive prioritization would suffice, such as: most recurrent barriers, context dependent barriers, and barriers with greatest implications for scale-up.

**Response Reviewer #6 Comment 4:**

Previously, Reviewer (#1) suggested ranking for which we have implemented in the manuscript (see below for history of comments).

**(Revision 5 - *Jul 08 2025)* Reviewer 1 Comment 9: The table of barriers and facilitators, although useful, is too long and not ranked. For example, facilitators such as increased mobile phone use are mentioned, but they are not categorized according to their level of impact. Recommendations: Clearly specify the clinical outcomes assessed, including the duration of hypertension, the magnitude of improvement in adherence, and any clinically significant blood pressure reduction. If clinical outcomes were not reported in the studies, authors should clearly state this and highlight this as a research gap. Consider reorganizing the barriers and facilitators into shorter, thematically grouped tables, and indicate how many studies reported each item.**

**Author Response to Comment 9:** We thank the reviewer for this insightful comment. We feel that the current table is appropriate in providing a comprehensive overview of the barriers and facilitators reported across the included publications. Ranking these factors would require us to make subjective judgments, as the studies varied in design, intervention type, and context (as detailed in Table 3). Given this heterogeneity, it would be difficult to assign relative levels of impact across such diverse settings.

**(Revision 7***-* ***Dec 30 2025****)* **REVIEWER COMMENT 6: RANKING/PRIORITIZATION OF BARRIERS AND FACILITATORS**

**WHAT IS REQUESTED:** to rank or prioritize barriers/facilitators (for greater applied utility).

**WHAT THE AUTHOR DOES:** does not make the ranking and justifies it as subjective and potentially biased in heterogeneous contexts; maintains a descriptive approach.

The central problem with this work is that it dismisses clinical relevance as the very foundation of the research it seeks to justify; in other words, it dismisses the applicability of the work. As a public health researcher... And as a clinical physician, I believe that in the context of a high-impact chronic disease such as hypertension arterial, to perform an analysis in which it is deliberately declared “narrative” and “conceptual” not It manages to substantively integrate clinical outcomes but falls into a recurring vice of contemporary science: the production of self-referential knowledge, methodologically coherent but socially sterile.

The acceptability, feasibility, or adoption of the proposed model is meaningless if it is not linked to its actual capacity to modify blood pressure control, prevent cardiovascular events, or reduce disease burden. These aspects are only mentioned. Within the theoretical framework and taxonomies without clinical anchoring, the study substitutes impact for classification and evidence for description, which limits its falsifiability, its usefulness for decision-makers, and its transformative potential in communities facing a disproportionate burden of disease.

The title mentions Hypertension Management in African In adults, management corresponds to a clinical intervention with a measurable outcome. I believe the title of the work should be modified to "digital education" or "digital use, not management."
This approach not only weakens the scientific contribution of the manuscript, but also reproduces a form of research that prioritizes academic exercise over the ethical responsibility to generate actionable knowledge with real impact on public health.

***Author Response:*** *We sincerely thank the reviewer for their thoughtful reflection on the clinical relevance of this work. While we respectfully differ from the reviewer’s narrower framing of hypertension management solely in terms of clinical outcomes, we fully agree that, given the high burden of hypertension across African settings, clinical measures such as blood pressure control, treatment adherence, and cardiovascular risk reduction are critical. Achieving meaningful public health impact, however, requires not only clinical efficacy but also the broad implementation of evidence-based interventions. Foundational work by Balas and Boren (2000, Managing Clinical Knowledge for Health Care Improvement) and others (Ramly E, Brown HW. Beyond Effectiveness: Implementation Science 101 for Clinicians and Clinical Researchers. Urogynecology (Phila). 2023 Mar 1;29(3):307-312.; Kelly, B. (2017). Implementation science: Applying the evidence of effectiveness in real-world contexts. Frameworks for practice in educational psychology: A textbook for trainees and practitioners, 78-94.) provides the conceptual basis for implementation science, highlighting how implementation research is essential for translating clinical knowledge into public health impact.*

*This systematic review was intentionally designed to address a complementary question: how mHealth interventions for hypertension are implemented in real-world African contexts. Our primary focus was on synthesizing implementation outcomes—acceptability, feasibility, and adoption—guided by Proctor’s implementation outcomes framework. While clinical effectiveness and implementation performance are interdependent, they represent analytically distinct domains that require separate but coordinated evaluation.*

*In high-burden, resource-constrained settings, evidence of clinical efficacy alone is insufficient to guide scale-up. Interventions must also demonstrate consistent deliverability, acceptability to patients and providers, and integration into existing health systems. By emphasizing implementation outcomes, this review addresses a critical translational gap between efficacy and sustainable scale-up.*

*To address the reviewer’s concern regarding framing and emphasis, we have:*

1. *Revised the title to explicitly reflect the implementation-focused scope of the review.*
2. *Ensured transparent reporting of clinical outcomes, systematically extracted and presented in Supplementary Table 1 to provide contextual interpretation.*

*We believe that clearly distinguishing between clinical effectiveness and implementation performance strengthens the methodological coherence of the manuscript while preserving its clinical and policy relevance. This clarification ensures readers understand both the scope and the contribution of the review in informing effective and scalable mHealth strategies in diverse African contexts.*

5. **Urban-rural interpretation.**

Rural studies are scarce and the manuscript does not over-interpret urban–rural differences correctly. Nevertheless, a short descriptive comparative review should be provided, dividing urban, peri-urban and rural studies by outcome measures, intervention modality and barriers to feasibility. This would increase transparency while not implying statistical comparison.

**Response Reviewer #6 Comment 5:**

Please see below for previous comments for which we have sufficiently addressed in the manuscript.

**(Revision 2 *- Jun 10 2024* ) Reviewer 1 Comment 30: “It would be helpful to add a column in the table for studies’ settings (Urban/ Peri-Urban/ Rural). Cite them in the text as well.”**

Author Response to Comment 30: We have added a column in Table 2 for studies’ settings (Urban/Peri-Urban/Rural) and have cited them in the text as well.

**(Revision 4 *- Apr 06 2025****)* **Reviewer #5 Comment 4: Tables are generally too many and too detailed. They do not assist the reader in acquiring an overview of the covered material.**

**Author Response to Reviewer#5, Comment 4:** The additional column on study settings (Urban/Peri-Urban/Rural) was included in response to a previous reviewer’s request (Reviewer 1, Comment 30, 12/17/2024: *“It would be helpful to add a column in the table for studies’ settings (Urban/ Peri-Urban/ Rural). Cite them in the text as well.”*) to enhance contextual clarity. While we understand the concern about table length and complexity, we believe the current structure balances comprehensiveness with clarity and supports readers in interpreting the findings.

**(Revision 5 - *Jul 08 2025*) Reviewer 1 Comment 7: Results should be more clearly linked to specific studies; currently, study-level findings are inconsistently described. There is no exploration of variations between rural and urban settings, which is essential given the digital divide in African contexts.**

**Author Response to Comment 7:** We would like to clarify that study-specific outcomes were consistently reported in Table 3, where similarities and differences between studies are highlighted. In response to this comment, we have further elaborated in the Results section to ensure clearer linkage between findings and individual studies. Additionally, we have expanded our analysis to include distinctions between rural and urban settings, thereby addressing the digital divide in African contexts and providing greater contextual understanding.

**(Revision 7 - *Dec 30 2025***) **Reviewer Comment 4: Comparative urban-rural analysis**

**WHAT IS REQUESTED:** a clearer comparison, with methodological justification, better integrating the “gap” "digital that divides" and the scarcity of rural studies in results and discussion.

**WHAT THE AUTHOR DOES:** explicitly rejects making the formal comparison and justifies it by insufficient evidence and risk of bias/ overinterpretation; states that "it would not be methodologically sound".

Response to Reviewer #6 – Comment 4

We thank the reviewer for highlighting the relevance of urban–rural context and the broader issue of digital inequities. The distribution of studies included in this review reflects the outcome of a predefined and rigorous search strategy and application of inclusion and exclusion criteria. Among eligible studies, most were conducted in urban settings, with three studies from rural settings and one from a peri-urban context.

While we acknowledge that urban–rural differences and digital access are important considerations in digital health research, this systematic review was not designed to formally assess the digital divide, nor was this construct specified within the search strategy or analytic framework. Given the limited number of rural studies and the absence of standardized measures of digital access across studies, conducting a formal comparative analysis would risk overinterpretation beyond the available evidence.

Accordingly, the Results and Discussion focus on synthesizing implementation outcomes—acceptability, feasibility, and adoption of mHealth interventions among adults diagnosed with hypertension—across included studies, independent of geographic setting. We believe this approach is consistent with the methodological scope of the review and aligns with principles of transparency and rigor in evidence synthesis.

However, we appreciate this suggestion and have added a descriptive comparative overview to the Results section without implying any statistical comparison. “Of the 18 included studies, 14 were conducted in urban settings, three in rural areas, and one in a peri-urban setting. Among the urban studies, implementation outcomes included acceptability (n=8), feasibility (n=9), and adoption (n=4), with app-based interventions predominating (11 of 14). In the three rural studies (Vedanthan et al., 2019 in western Kenya; Mannik et al., 2016 in rural Kenya; Mugabirwe et al., 2021 in rural Uganda), intervention modalities included SMS-based community screening (Mannik et al.), SMS for linkage to care (Vedanthan et al.), and app-based blood pressure monitoring (Mugabirwe et al.). Rural studies reported acceptability as the primary implementation outcome across all three, with scalability noted in one (Mannik et al.). Key feasibility barriers specific to rural settings included power interruptions to clinic infrastructure (Mannik et al.), limited connectivity, and device access constraints (Mugabirwe et al.)—barriers that differ in nature and severity from those reported in urban studies, where technology literacy and network instability were more prominent. The single peri-urban study (Otu et al., 2022 in Nigeria) reported feasibility and scalability outcomes, with tablet-delivered training and WhatsApp group support as the intervention modality.” We have also added this to the study limitations section: “This descriptive comparison reveals that rural and peri-urban studies are too few and heterogeneous to support any inferential comparison with urban findings.”

6. **Consider the effect of follow-up time on sustainability.**

The manuscript now acknowledges that most of the studies had short to medium follow-up. The authors should go the extra mile and explicitly state that sustainability, duration and maintenance of mHealth implementation cannot be inferred from the current evidence. Hence, any claims on scalability or sustained implementation should be treated as preliminary.

**Response Reviewer #6 Comment 6:**

We fully accept this recommendation. The mean follow-up duration across the 18 included studies was approximately 11 months, ranging from one month to 32 months, with the majority of studies falling in the short-to-medium range. None of the included studies were designed to assess long-term sustainability, duration, or maintenance of mHealth implementation beyond the study period. We have added explicit language to both the Limitations and Conclusion sections stating that sustainability, duration, and maintenance of mHealth implementations cannot be inferred from the current body of evidence.

7. **How risk of bias influenced the synthesis**

The manuscript reports low overall risk of bias. However, the authors should briefly mention if and how the risk of bias findings were used to interpret the evidence. For example, they should state whether studies with a high risk in any domain were interpreted cautiously, whether all studies were given equal weight in the synthesis, or whether risk of bias was used only descriptively.

**Response Reviewer #6 Comment 7:**

We thank the reviewer for this comment. As stated in the manuscript (pages 68-69), risk of bias was low across studies. Risk of bias was used descriptively to characterize the methodological quality of the evidence base, rather than as a formal weighting mechanism in the evidence synthesis. Given that most studies demonstrated low overall risk of bias across the ASSESS tool domains, all studies were considered in the synthesis without differential weighting. High risk in any domain were interpreted cautiously. Please refer to the Risk of Bias supplemental files (image and excel sheet) attached to the submission.
