# Supplementary material for: Mobile health interventions for African adults with hypertension: Evidence from a systematic review of implementation research outcomes
Source: PLOS Digit Health. 2026 Aug 14;5(8):e0001612. doi: 10.1371/journal.pdig.0001612 (PMC13475905; doi:10.1371/journal.pdig.0001612)
Supplement: Supplementary file 15 [file pdig.0001612.s011.docx]

**Summary**

Thank you for the opportunity to review this manuscript. This is a systematic review to determine the acceptability, feasibility, and adoption rates of mHealth intervention among adults diagnosed with hypertension across African countries. The authors completed a search of the literature in 2023. The review identified 18 studies (RCTs, cohort, cross-sectional, qualitative and mixed-methods studies). The studies were summarized narratively. The significance of the review lies in its focus on addressing the pressing issue of hypertension, which poses a substantial burden on global health, particularly in the context of African nations. By examining the acceptability, feasibility, and adoption of mHealth interventions, the review contributes to the understanding of how technology-based solutions can be leveraged to improve hypertension management among African adults. My comments provided are structured according to different sections of the manuscript, aiming to offer constructive feedback for enhancing the clarity, rigour, and coherence of this systematic review.

**Abstract**

1. The aim of the review should be clearer regarding including facilitators and barriers affecting implementation outcomes.
2. What do you mean by the World Bank criteria in the abstract? You should clarify it for the reader
3. The Abstract is a standalone, abbreviations should be spelt out in full the first time mentioned, PRISMA, LMICs, etc...
4. You should include the date of the search
5. In the method section, it does not specify the specific study designs included or any other criteria used for study selection
6. Please follow the PRISMA checklist for Abstract : Page, M.J., McKenzie, J.E., Bossuyt, P.M. *et al.* The PRISMA 2020 statement: an updated guideline for reporting systematic reviews. *Syst Rev* **10**, 89 (2021). <https://doi.org/10.1186/s13643-021-01626-4>
7. The abstract should be tightened up for conciseness

**Introduction Section**

1. The aim of the review should be clearer regarding including facilitators and barriers affecting implementation outcomes.
2. The first two sentences: “Hypertension affects over 1.28 billion adults … (LMICs)”, **AND** “Africa is disproportionately burdened with hypertension … compared to prevalence rates of 18% in the Americas” do not exist in the cited references [1] and [2] respectively. Instead, they are in the following factsheet regarding hypertension by WHO: <https://www.who.int/news-room/fact-sheets/detail/hypertension>
3. According to WHO (WHO. *A global brief on hypertension*. 2023 <https://www.who.int/publications/i/item/9789240081062>), which you cited for literature, they defined hypertension as having (SBP) ≥140 mmHg or (DBP) ≥90 mmHg or taking medication for hypertension. Given that, the sentence in the introduction according to AHA guidelines that “Hypertension, defined as an average of repeatedly measured (SBP)≥ 130 mmHg and or (DBP)≥80 mmHg[3]” makes it not consistent.
4. The sentence “Hypertension and diabetes also share common risk factors, including obesity, physical inactivity, …”. This sentence isn’t cited with a reference and despite the shared risk factors between hypertension and diabetes. It’s not clear why you mentioned diabetes here? Is there something in particular you want to raise regarding diabetes?
5. The sentence “(10 % of all pregnant women in Africa suffer from pregnancy-induced hypertension compared to the global average of 2 percent).[6]”, could you please indicate if this sentence reflects the reference you cited?
6. The reference is missing for the sentence starting with “It has demonstrated efficacy in enhancing patient-provider communication … for in-person consultations with a healthcare provider”.
7. The sentence “To date, relatively few studies address usability and feasibility issues from an African context”. It would be helpful to cite the few studies that addressed this topic, for clarity for the readers.

**Method section**

1. The databases in the PRISMA flow chart are not the same databases you stated in the method section.
2. Was the search strategy developed with an experienced librarian in conducting systematic reviews? Was the search strategy peer-reviewed using PRESS?
3. Inclusion criteria: “Study population diagnosed with hypertension”. What is the definition of hypertension? According to which guidelines?
4. What about the studies in which their populations weren’t diagnosed with hypertension? Did you exclude them? This should be stated clearly
5. Was the framework used for implementation outcomes (acceptability, feasibility, etc) considered as part of the eligibility criteria for the studies included?
6. Dele-Ojo, B.F., et al The effect of mobile health technology on blood pressure control among patients with hypertension in Ghana and Nigeria. This study is a **preprint** and has not yet been peer-reviewed. Please be transparent in your review about the inclusion of preprints. Pay extra attention to the methodology, data integrity, and potential biases.
7. In the Data extraction section, the authors who handled the screening process for the citations, their initials should be mentioned between brackets for transparency and clarity. Also, this section lacks the strategy of full-text data extraction, including but not limited to, who did extract the data, what the data extracted e.g., author, study design, duration, implementation outcomes, etc.
8. The study risk of bias assessment is not mentioned in the method section instead, it was reported in the result section. Specify the methods used to assess the risk of bias in the included studies, including details of the ASSESS tool you used, how many reviewers assessed each study and whether you worked independently in method section.
9. Please provide the OSF registration number/ DOI instead of the URL website.
10. “We developed an extensive search strategy to locate published trials across nine databases” You included different types of studies not only trials.

**Result section**

1. Number of the studies per design in PRISMA (identification & Screening) is not identical in the Method section. Also, the number of Quantitative method studies and mixed method are not the same in Table 1 and literature search in result section. This should be revised.
2. There are no citations in Table 1 (characteristics of the studies) for the studies included. You should embed the references for clarity and the ease of accessing them.
3. Reporting the study designs in Table 1 is not consistent for non-randomised studies, i.e., should write either (non-randomised- type of observational study) or the type of observational study, this helps make reporting consistent and easy for the reader to follow.
4. 10 articles not 9 which assessed acceptability, is there any chance you missed the last article which discussed acceptability?
5. It would be helpful to add a column in the table for studies’ settings (Urban/ Peri-Urban/ Rural). Cite them in the text as well.
6. In-text citations in the result sections for the studies included are missing. The citations should be embedded.
7. What do you mean by “Most of the experimental research concentrated on subjects with elevated blood pressure and confirmed hypertension” aren’t all the studies’ subjects are hypertensive participants per Eligibility criteria?
8. Were you trying to interpret the findings in the results section e.g. “These findings underscore…”, and “ The respondents' attitudes in the study underscored a robust desire…”? Leave the comments and interpretations for the Discussion section.
9. “The study further revealed that our findings contribute to the evidence that mHealth interventions for post-stroke care are implementable in resource-constrained settings”. Is it your finding or their finding? Please revise and paraphrase.
10. Risk of Bias**:** It’s quite interesting to see all the studies included in this review are low risk of bias studies. However, it’s important to see explicitly how the studies were being assessed and scored by the ASSESS tool domains. Is there any supplementary material showing that? If not, I would highly recommend compelling one to help see how the studies are being assessed against the five criteria for each study design.

**Discussion section**

1. The manuscript includes a section on facilitators and barriers in the result section, but it could more explicitly link these in the discussion to the acceptability, feasibility, and adoption discussed earlier
2. The discussion structure could be enhanced more, to make it easier for readers to follow the interpretation of the results, comparison with existing literature etc...
3. The critical analysis should be enhanced by including the strengths and limitations of the reviewed studies (study designs, potential biases, etc..) and the review process itself.
4. The conclusion section could benefit from clearer, more concise statements. Some sentences are long and could be split to enhance readability and impact.
