# Supplementary material for: Mobile health interventions for African adults with hypertension: Evidence from a systematic review of implementation research outcomes
Source: PLOS Digit Health. 2026 Aug 14;5(8):e0001612. doi: 10.1371/journal.pdig.0001612 (PMC13475905; doi:10.1371/journal.pdig.0001612)
Supplement: Supplementary file 16 [file pdig.0001612.s012.docx]

**Manuscript Review**

**Manuscript Number:** PDIG-D-24-00124.

**Title**: Determining Acceptability, Feasibility, and Adoption rates associated with Mobile

Health interventions for hypertension management among adults in Africa - A

Systematic Review

**General Comments:** The present paper addresses the utility of mobile health technologies as interventions in the management of hypertension in Africa. The paper is a systematic review which has sourced 9 databases with keywords of hypertension, blood pressure, mobile health and implementation outcomes. 650 articles were identified of which 60 were advanced for this revIew, which upon further detailed review by the authors 18 were ultimately found to be relevant and utilized in the present paper. These papers were then visually outlined utilizing the PRISMA flow chart as a systematic means of reporting particular data and variables. In particular, feasibility, acceptability and adoption we're highlighted. Following data extraction the authors contributed in providing detailed presentation in table form, followed by discussion, of the facilitators and barriers to implementation of mHealth technology. Papers utilized were also assessed as to the quality in terms of content bias. In general, the review presents significant information and highlights remaining limitations which need to be overcome in order to facilitate the utility of mHealth and phone-based technologies as a means of making a difference in hypertension management in Africa. In general, this is a well done study and a well-written paper which addresses a clinical disease state of major significance.

To enhance the paper the following issues and suggestions are raised and should be addressed:

1. While this is a systematic review the paper would still benefit from including a hypothesis up front in the introduction. The utility of this is that it enhances the overall value for readers in leading to the direction of purpose and future research, rather than simply analyzing data. So, suggesting something along the order of “widespread utilization and adoption of mHealth technologies may or will facilitate the management of hypertension. Here we sought to ascertain the degree of present efficacy utilizing a systematic review….” would set the paper up in a better way.

It would also be useful to state exactly what questions or aims are being explored in this review rather than simply stating that this is a review. The way stated presently it leaves the reader to guess.

2. In the abstract a range of acronyms and abbreviations are introduced. It would be useful to provide explanation of abbreviations, at least for LMICS.

3. In the abstract it would be useful to expand the first method sentence to explain more clearly is this a published paper review or did you do actual perform data analysis. It is not clear for the reader. As this is right up front and what most people (only, sadly) read greater clarity is needed here.

4. The introduction is well written but a bit too wordy. Being more concise would be much more useful.

5. In the methods it would be useful to state explicitly whether or not a large language model or other AI system was utilized in this paper. In the current world in which we live with the increasing use of AI and people questioning the veracity of information stating this is very important.

6. In the results. In description of information extracted throughout the section “characteristics of identified studies” qualitative descriptor terms such as “most” studies or “some” studies are often utilized. This these terms are too general. It would be much better to state the number divided by the denominator of studies such as four out of eleven (4/11) or three out of seven (3/7). Using “most” or “some” inadvertently adds interpretive bias, which is something the authors have worked hard to define in the basal studies, so utilizing this approach is much cleaner. Finally, if you decide to continue to use most, often and frequently it is good to add the numbers next to these descriptors as has been illustrated here.

7. In section V. Study design and technological approaches. Here again it would be cleaner instead of saying 44% of randomized controlled trials, since the numbers are small, it would be better to state X over the denominator and the percentage. i.e. X/Y Studies (44%).

8. In the discussion it would be useful to provide insights and suggestions as to means of assessing the impact of the mHealth and digital interventions that have been revealed. Some of this is done in the third paragraph of the discussion though this could be expanded and would add value for the reader.

9. In that discussion section, i.e. in the 3rd and 4th paragraph it might be useful to comment on the speed it has taken for any change to occur with any of the mHealth interventions utilized as to having impact on HTN management. How quickly did this alter hypertensive management? And similarly, to comment on what the recidivism or recrudescence rates were despite utilizing these technologies? - if that information was available from any of the studies reviewed.

10. It would be useful in the conclusion or the end of the discussion to provide for the reader the authors perspective on some expanded next steps. There are only one or two sentences that address this. Again, tying this to an upfront hypothesis one might suggest that additional studies be done related to the combination of continued education along with economic efforts to enhance phone and SMS use and understanding in patients across an educational spectrum or enhanced education related to the importance of hypertension as a silent disease would be useful. In this reviewer's eyes reviews are useful to provide data but also should be suggestive of next steps. Adding a few sentences here would be valuable for broad readership.
