# Supplementary material for: Mobile health interventions for African adults with hypertension: Evidence from a systematic review of implementation research outcomes
Source: PLOS Digit Health. 2026 Aug 14;5(8):e0001612. doi: 10.1371/journal.pdig.0001612 (PMC13475905; doi:10.1371/journal.pdig.0001612)
Supplement: Supplementary file 17 [file pdig.0001612.s014.docx]

**Date:** September 12^th^, 2024

**Manuscript title:** Determining Acceptability, Feasibility, and Adoption rates associated with Mobile Health interventions for hypertension management among adults in Africa - A Systematic Review

**Manuscript number:** PDIG-D-24-00124R1

Dear Editor,

We sincerely appreciate the reviewers' invaluable feedback on our manuscript. Their insightful comments have significantly contributed to improving the quality of our work. We have carefully addressed each of the reviewers' suggestions, resulting in enhanced clarity and coherence throughout the manuscript.

Below, we have provided a detailed response to the specific points raised by the reviewers.

Thank you for considering our revisions. We look forward to your feedback.

Best regards,

Priyanka Lanka

Master of Public Health Candidate

NYU School of Global Public Health

[pl2461@nyu.edu](mailto:pl2461@nyu.edu)

**Responses to Reviewers’ Critiques to Enhance the Abstract**

**Reviewer 1, Comment 1: “The aim of the review should be clearer regarding including facilitators and barriers affecting implementation outcomes.”**

Author Response to Comment 1: We appreciate the reviewer's insightful comment. In response, we have revised the aim of the review in the abstract to explicitly highlight both the facilitators and barriers that affect implementation outcomes. This revision clarifies the focus of the review and provides a more comprehensive understanding for the reader.

**Reviewer 2, Comment 2: “In the abstract it would be useful to expand the first method sentence to explain more clearly is this a published paper review or did you do actual perform data analysis.  It is not clear for the reader. As this is right up front and what most people (only, sadly) read greater clarity is needed here.”**

Author Response to Comment 2: We thank the reviewer for their thorough review of the manuscript. In response, we have revised the abstract to enhance clarity and address the concern raised.

**Reviewer 1, Comment 3: “What do you mean by the World Bank criteria in the abstract? You should clarify it for the reader”**

Author Response to Comment 3: In response to the reviewer's comment, we have expanded on the World Bank criteria in the abstract to clarify its relevance and application within the context of our study.

**Reviewer 1 Comment 4: “The Abstract is a standalone, abbreviations should be spelt out in full the first time mentioned, PRISMA, LMICs, etc...”**

**Reviewer 2 Comment 4: “In the abstract a range of acronyms and abbreviations are introduced.   It would be useful to provide explanation of abbreviations, at least for LMICs.”**

Author Response to Comment 4: We apologize for not thoroughly reviewing our manuscript prior to submission and thank the reviewer for their valuable suggestions. We have now spelled out all abbreviations, such as PRISMA and LMICs, in full upon their first mention in the abstract.

**Reviewer 1 Comment 5: “You should include the date of the search**”

Author Response to Comment 5: We have included the date of the search in the abstract, specifying that the last search was conducted on March 26th, 2024.

**Reviewer 1 Comment 6: “In the method section, it does not specify the specific study designs included or any other criteria used for study selection”**

Author Response to Comment 6: The method section of the abstract has been revised to specify the study designs included as well as other criteria used for study selection.

**Reviewer 1 Comment 7: “Please follow the PRISMA checklist for Abstract: Page, M.J., McKenzie, J.E., Bossuyt, P.M. *et al.* The PRISMA 2020 statement: an updated guideline for reporting systematic reviews. *Syst Rev* 10, 89 (2021).** [**https://doi.org/10.1186/s13643-021-01626-4**](https://doi.org/10.1186/s13643-021-01626-4)**”**

Author Response to Comment 7: We thank the reviewer for their suggestions. In response, we have updated the PRISMA checklist for the abstract in accordance with the guidelines by Page et al. (2021). Additionally, we confirm that our abstract now aligns with the PRISMA 2020 statement.

**Reviewer 1 Comment 8: “The abstract should be tightened up for conciseness”**

Author Response to Comment 8: We have revised the abstract, ensuring that the information is presented in a more streamlined and effective manner.

**Responses to Reviewer Critiques to enhance the Introduction Section**

**Reviewer 2 Comment 9: “While this is a systematic review the paper would still benefit from including a hypothesis up front in the introduction. The utility of this is that it enhances the overall value for readers in leading to the direction of purpose and future research, rather than simply analyzing data. So, suggesting something along the order of “widespread utilization and adoption of mHealth technologies may or will facilitate the management of hypertension. Here we sought to ascertain the degree of present efficacy utilizing a systematic review….” would set the paper up in a better way. – agreed and added as suggested in the last paragraph of introduction.”**

Author Response to Comment 9: We appreciate the reviewer's valuable input. In response, we have added a hypothesis in the final paragraph of the introduction to address this concern.

**Reviewer 1 Comment 10: “The aim of the review should be clearer regarding including facilitators and barriers affecting implementation outcomes.”**

**Reviewer 2 Comment 10: “The introduction is well written but a bit too wordy. Being more concise would be much more useful.”**

Author Response to Comment 10: We have revised the introduction to provide clarity and conciseness for systematic review, particularly regarding the inclusion of facilitators and barriers affecting implementation outcomes. This is how we revised it by adding the aim more clearly - “Therefore, this systematic review aims to determine the acceptability, feasibility, and adoption rates of mHealth intervention among adults diagnosed with hypertension across African countries, while also identifying various facilitators and barriers influencing these implementation outcomes to provide a comprehensive understanding of the factors affecting their successful implementation.”

**Reviewer 1 Comment 11: “The first two sentences: “Hypertension affects over 1.28 billion adults … (LMICs)”, AND “Africa is disproportionately burdened with hypertension … compared to prevalence rates of 18% in the Americas” do not exist in the cited references [1] and [2] respectively. Instead, they are in the following factsheet regarding hypertension by WHO:** [**https://www.who.int/news-room/fact-sheets/detail/hypertension**](https://www.who.int/news-room/fact-sheets/detail/hypertension) **“**

Reviewer 1 Comment 11: We sincerely thank the reviewer for their thorough review. We regret the citation error and have now corrected it. The first two sentences of the introduction have been revised to accurately reflect the source of the information, and the correct reference to the WHO factsheet on hypertension has been added.

**Reviewer 1 Comment 12: “According to WHO (WHO. *A global brief on hypertension*. 2023** [**https://www.who.int/publications/i/item/9789240081062**](https://www.who.int/publications/i/item/9789240081062)**), which you cited for literature, they defined hypertension as having (SBP) ≥140 mmHg or (DBP) ≥90 mmHg or taking medication for hypertension. Given that, the sentence in the introduction according to AHA guidelines that “Hypertension, defined as an average of repeatedly measured (SBP)≥ 130 mmHg and or (DBP)≥80 mm Hg [3]” makes it not consistent.”**

Author Response to Comment 12: We have addressed the reviewer’s concern about the differing definitions of hypertension. While the definition for hypertension was taken from the AHA guidelines, only the second sentence was cited from the WHO document. The sentence has been amended to provide clarity to the reader about the data source. Furthermore, we used the updated AHA guidelines for this review.

**Reviewer 1 Comment 13: “The sentence “Hypertension and diabetes also share common risk factors, including obesity, physical inactivity, …”. This sentence isn’t cited with a reference and despite the shared risk factors between hypertension and diabetes. It’s not clear why you mentioned diabetes here? Is there something in particular you want to raise regarding diabetes?”**

Author Response to Comment 13: The sentence regarding the shared risk factors between hypertension and diabetes has been clarified. We included this information because our review covers studies on both conditions. Additionally, we have provided further context to explain the relevance of including diabetes and stroke in our study.

**Reviewer 1 Comment 14: “The sentence “(10 % of all pregnant women in Africa suffer from pregnancy-induced hypertension compared to the global average of 2 percent).[6]”, could you please indicate if this sentence reflects the reference you cited?”**

Author Response to Comment 14: The sentence regarding pregnancy-induced hypertension in Africa has been revised to accurately reflect the correct reference. The previous citation was an error, and the correct reference has now been included.

**Reviewer 1 Comment 15: “The reference is missing for the sentence starting with “It has demonstrated efficacy in enhancing patient-provider communication … for in-person consultations with a healthcare provider””**

Author Response to Comment 15: We have added the missing reference for the sentence discussing the efficacy of enhancing patient-provider communication for in-person consultations with a healthcare provider.

**Reviewer 1 Comment 16: “The sentence “To date, relatively few studies address usability and feasibility issues from an African context”. It would be helpful to cite the few studies that addressed this topic, for clarity for the readers.”**

Author Response to Comment 16: In response to the comment regarding studies addressing usability and feasibility issues in the African context, we have added citations for the few studies that have explored this topic.

### Responses to Reviewer Critiques to enhance the Method Section

**Reviewer 1 Comment 17: “The databases in the PRISMA flow chart are not the same databases you stated in the method section.”**

Author Response to Comment 17: There was downloading error with the PRISMA file from Covidence, which resulted in APA PsychInfo being deleted. We have corrected this issue, edited, and downloaded a new PRISMA flow diagram that accurately reflects the databases used.

**Reviewer 1 Comment 18: “Was the search strategy developed with an experienced librarian in conducting systematic reviews? Was the search strategy peer-reviewed using PRESS?”**

Author Response to Comment 18: We thank the reviewer for this comment. Our search strategy was developed in collaboration with an expert medical librarian (co-author DV) who has over 25 years of experience conducting systematic reviews at NYU School of Medicine and is a professor of library sciences specializing in best practices for systematic reviews. The medical librarian (DV) followed the Peer Review of Electronic Search Strategies-recommended approach.

**Reviewer 1 Comment 19: “Inclusion criteria: “Study population diagnosed with hypertension”. What is the definition of hypertension? According to which guidelines?” “What about the studies in which their populations weren’t diagnosed with hypertension? Did you exclude them? This should be stated clearly”**

Author Response to Comment 19: The definition of hypertension, as outlined by the guidelines utilized, is detailed in the introduction and reiterated in the methods section to ensure clarity. Additionally, we have explicitly stated that studies involving populations not diagnosed with hypertension were excluded, a fact clearly reflected in the inclusion and exclusion criteria. To guide our assessment of implementation outcomes, we employed Proctor’s taxonomy, which has been incorporated into the eligibility criteria for the studies.

**Reviewer 1 Comment 20: “Dele-Ojo, B.F., et al The effect of mobile health technology on blood pressure control among patients with hypertension in Ghana and Nigeria. This study is a preprint and has not yet been peer-reviewed. Please be transparent in your review about the inclusion of preprints. Pay extra attention to the methodology, data integrity, and potential biases.”**

Author Response to Comment 20: We acknowledge that the study by Dele-Ojo, B.F., et al. is a preprint and has not yet undergone peer review. In response, we have added a statement in the methods section clarifying that preprints were not excluded from our search strategy, a decision made following consultations with experts and the medical librarian overseeing the review. We have addressed potential biases through our rigorous methodology and comprehensive risk of bias assessments. We appreciate the reviewer's thorough examination and constructive recommendations.

**Reviewer 1 Comment 21: “In the Data extraction section, the authors who handled the screening process for the citations, their initials should be mentioned between brackets for transparency and clarity. Also, this section lacks the strategy of full-text data extraction, including but not limited to, who did extract the data, what the data extracted e.g., author, study design, duration, implementation outcomes, etc.”**

Author Response to Comment 21: We have revised the manuscript to include the initials of the co-authors involved in the screening process, as recommended by the reviewer. Furthermore, we have provided a comprehensive description of the full-text data extraction strategy. This includes details on which co-authors performed the data extraction, the specific data elements extracted (e.g., author, study design, duration, implementation outcomes, etc.), and this information has been documented in Appendix A.

**Reviewer 1 Comment 22: “The study risk of bias assessment is not mentioned in the method section instead, it was reported in the result section. Specify the methods used to assess the risk of bias in the included studies, including details of the ASSESS tool you used, how many reviewers assessed each study and whether you worked independently in method section.”**

Author Response to Comment 22: The risk of bias assessment has been moved to the methods section, where we have expanded on the ASSESS tool used, including details on the number of reviewers and whether they worked independently.

**Reviewer 1 Comment 23: “Please provide the OSF registration number/ DOI instead of the URL website.”**

Author Response to Comment 23: We have provided the OSF registration number/DOI instead of the URL website within the revised manuscript.

**Reviewer 1 Comment 24: “We developed an extensive search strategy to locate published trials across nine databases” You included different types of studies not only trials**.”

Author Response to Comment 24: We thank the reviewer for their thorough review and have revised the manuscript to address this concern. The phrase "published trials" has been rectified to "published articles".

**Reviewer 2 Comment 25: “In the methods it would be useful to state explicitly whether or not a large language model or other AI system was utilized in this paper. In the current world in which we live with the increasing use of AI and people questioning the veracity of information stating this is very important.”**

Author Response to Comment 25: No large language model or AI system was used in the development of this paper ad this has been explicitly mentioned in methods section.

**Responses to Reviewer Critiques to enhance the Result Section**

**Reviewer 1 Comment 26: “Number of the studies per design in PRISMA (identification & Screening) is not identical in the Method section. Also, the number of Quantitative method studies and mixed method are not the same in Table 1 and literature search in result section. This should be revised.”**

Author Response to Comment 26: We sincerely apologize for the oversight and have corrected the discrepancies concerning the number of studies per design in the PRISMA flow chart and the methods section. Additionally, we have resolved the inconsistencies between Table 1 and the literature search in the results section regarding the counts of quantitative and mixed-method studies.

**Reviewer 1 Comment 27: “There are no citations in Table 1 (characteristics of the studies) for the studies included. You should embed the references for clarity and the ease of accessing them”**

Author Response to Comment 27: We have reviewed Table 1 (now renamed as Table 2) and embedded citations in the 'Author' column for the studies included.

**Reviewer 1 Comment 28: “Reporting the study designs in Table 1 is not consistent for non-randomised studies, i.e., should write either (non-randomised- type of observational study) or the type of observational study, this helps make reporting consistent and easy for the reader to follow.”**

Author Response to Comment 28: We have ensured consistent reporting of study designs for non-randomized studies in Table 2 by adhering to a clear and uniform classification of observational study types. This approach enhances clarity and uniformity in the presentation of study designs.

**Reviewer 1 Comment 29: “10 articles not 9 which assessed acceptability, is there any chance you missed the last article which discussed acceptability?”**

Author Response to Comment 29: We identified an oversight where 10 articles assessed acceptability, not 9. This has been corrected in Table 2 and reflected as 'n=10' in the text, correctly representing 56% of the studies included.

**Reviewer 1 Comment 30: “It would be helpful to add a column in the table for studies’ settings (Urban/ Peri-Urban/ Rural). Cite them in the text as well.”**

Author Response to Comment 30: We have added a column in Table 2 for studies’ settings (Urban/Peri-Urban/Rural) and have cited them in the text as well.

**Reviewer 1 Comment 31: “In-text citations in the result sections for the studies included are missing. The citations should be embedded.”**

Author Response to Comment 31: We have embedded in-text citations for the studies included in the results section to ensure all references are clearly identified.

**Reviewer 1 Comment 32: “What do you mean by “Most of the experimental research concentrated on subjects with elevated blood pressure and confirmed hypertension” aren’t all the studies’ subjects are hypertensive participants per Eligibility criteria?”**

**Reviewer 2 Comment 32: “In the results. In description of information extracted throughout the section “characteristics of identified studies” qualitative descriptor terms such as “most” studies or “some” studies are often utilized. This these terms are too general.”**

Author Response to Comment 32: We have revised the statement regarding study subjects to accurately reflect that "All of the experimental research concentrated on subjects with elevated blood pressure and confirmed hypertension," consistent with our eligibility criteria.

We have also ensured not to use qualitative descriptor terms such as “most” studies or “some” studies and instead tried to mention accurate numbers and percentages.

**Reviewer 1 Comment 33: “Were you trying to interpret the findings in the results section e.g. “These findings underscore…”, and “The respondents' attitudes in the study underscored a robust desire…”? Leave the comments and interpretations for the Discussion section.”**

Author Response to Comment 33: We have removed interpretative comments from the results section, such as “These findings underscore…” and “The respondents' attitudes in the study underscored a robust desire…,” and ensured that interpretations are confined to the discussion section.

**Reviewer 1 Comment 34: ““The study further revealed that our findings contribute to the evidence that mHealth interventions for post-stroke care are implementable in resource-constrained settings”. Is it your finding or their finding? Please revise and paraphrase.”**

Author Response to Comment 34: We have revised the statement about the findings on post-stroke care to clarify that it reflect the study’s findings and not ours.

**Reviewer 2 Comment 35: “In section V. Study design and technological approaches.  Here again it would be cleaner instead of saying 44% of randomized controlled trials, since the numbers are small, it would be better to state X over the denominator and the percentage. i.e. X/Y Studies (44%).”**

Author Response to Comment 35: We thank the reviewer for this comment. We have amended the manuscript to indicate 'X/ 18 studies (44%)' and the percentage, ensuring greater clarity for readers.

**Reviewer 1 Comment 36: “Risk of Bias: It’s quite interesting to see all the studies included in this review are low risk of bias studies. However, it’s important to see explicitly how the studies were being assessed and scored by the ASSESS tool domains. Is there any supplementary material showing that? If not, I would highly recommend a compelling one to help see how the studies are being assessed against the five criteria for each study design.”**

Author Response to Comment 36: We thank the author for this review. We used the ASSESS Tool for the risk of bias (RoB) analysis. We have uploaded the excel files of the ASSESS tool to OSF.

### Responses to Reviewer Critiques to enhance the Discussion Section

**Reviewer 1 Comment 37: “The manuscript includes a section on facilitators and barriers in the result section, but it could more explicitly link these in the discussion to the acceptability, feasibility, and adoption discussed earlier.”**

Author Response to Comment 37: We have revised the discussion section to explicitly connect the facilitators and barriers identified in the results with the implementation outcomes previously discussed. This linkage provides a clearer understanding of how these factors influence the outcomes.

**Reviewer 1 Comment 38: “The discussion structure could be enhanced more, to make it easier for readers to follow the interpretation of the results, comparison with existing literature etc...”**

Author Response to Comment 38: The structure of the discussion section has been enhanced to improve the flow and readability of the section. For example :

**Earlier version**:

Our analysis of a diverse range of studies on mHealth interventions for hypertension in African countries has revealed significant trends and considerations. We have grouped these trends under facilitators and barriers that can support or impede the implementation of evidence-based mHealth interventions in Africa (Table 2). Among the facilitators, that can promote mHealth implementation and adoption in Africa is the increasing availability and usage of cell phones in Africa that enable mHealth interventions to be acceptable to the population [12]. With the widespread of cell phone utilization among African populations, the technical feasibility of deploying mHealth solutions across diverse regions with high cell phone penetration becomes feasible as the availability of devices increase. The studies included in this systematic review provide valuable perspectives on the acceptability and feasibility of interventions utilizing mHealth Apps and SMS-based strategies that can be supported on currently available devices in Africa.

**Revised version**:

Our analysis of a diverse range of studies on mHealth interventions for hypertension in African countries has revealed significant trends and considerations, grouped into facilitators and barriers that can support or impede the implementation of evidence-based mHealth interventions in Africa (**Table 2**). Among the facilitators, the increasing availability and usage of cell phones in Africa is a major driver of mHealth adoption, making these interventions more feasible across regions with high mobile penetration[12].

**Reviewer 1 Comment 39: “The critical analysis should be enhanced by including the strengths and limitations of the reviewed studies (study designs, potential biases, etc..) and the review process itself.”**

Author Response to Comment 39: We have enhanced the critical analysis in the discussion section by including a detailed evaluation of the strengths and limitations of the reviewed studies.

**Reviewer 2 Comment 40: “In the discussion it would be useful to provide insights and suggestions as to means of assessing the impact of the mHealth and digital interventions that have been revealed. Some of this is done in the third paragraph of the discussion though this could be expanded and would add value for the reader.”**

Author response to Comment 40: We have addressed this comment by revising the discussion section to address the gap in impact measurement through adherence tracking and long-term clinical outcome measurement. We highlighted Dele Ojo for using the Hill – Bone Adherence scale to assess adherence through the study period. However, we also noted that none of the studies suggested the use of such tools beyond the study period which points to need for future research.

**Reviewer 2 Comment 41: “In that discussion section, i.e. in the 3rd and 4th paragraph it might be useful to comment on the speed it has taken for any change to occur with any of the mHealth interventions utilized as to having impact on HTN management. How quickly did this alter hypertensive management? And similarly, to comment on what the recidivism or recrudescence rates were despite utilizing these technologies? - if that information was available from any of the studies reviewed.”**

Author Response to Comment 41: While we agree that the reviewer's suggestion would enhance the manuscript, the speed of change is not explicitly addressed in the reviewed studies, except for the duration of the intervention after which its impact on hypertension management was evaluated. Additionally, none of the studies reported recidivism rates, as they focused solely on the specific intervention within a defined timeframe. Further research is needed to explore the sustainability of these interventions and to examine their characteristics, as suggested by the reviewer.

**Responses to Reviewer Critiques to enhance the Conclusion Section**

**Reviewer 1 Comment 42: “The conclusion section could benefit from clearer, more concise statements. Some sentences are long and could be split to enhance readability and impact.”**

Author Response to Comment 42: As suggested, the conclusion has been revised to incorporate future directions. The revised conclusion emphasizes the utility of mHealth interventions in hypertension management.

**Reviewer 2 Comment 43: “It would be useful in the conclusion or the end of the discussion to provide for the reader the authors perspective on some expanded next steps.  There are only one or two sentences that address this.”**

Author response to Comment 43: Thank you for your valued feedback. We have revised the conclusion to incorporate more specific next steps based on our review.
