# Supplementary material for: Mobile health interventions for African adults with hypertension: Evidence from a systematic review of implementation research outcomes
Source: PLOS Digit Health. 2026 Aug 14;5(8):e0001612. doi: 10.1371/journal.pdig.0001612 (PMC13475905; doi:10.1371/journal.pdig.0001612)
Supplement: Supplementary file 18 [file pdig.0001612.s017.docx]

**Manuscript Title:** Determining Acceptability, Feasibility, and Adoption rates associated with Mobile Health interventions for hypertension management among adults in Africa - A Systematic Review

Dear Editor and Reviewers,

We sincerely appreciate the time and effort you have dedicated to reviewing our manuscript and providing thoughtful, constructive feedback. Your insights have been invaluable in helping us strengthen the quality and clarity of our work.

We have carefully reviewed each of the comments and provided detailed, point-by-point responses to address all concerns raised during the review process. We are pleased to resubmit our revised manuscript to *PLOS ONE* for your further consideration. All reviewer suggestions have been thoroughly addressed in the revised version.

***Reviewer #4, Comment 1:*** *Formatting and titles of the tables is not correct for a scientific article. The introduction should have more information about the use of platforms. It focus too much on hypertension and not enough on the management platform.*

**Author Response to Reviewer#4, Comment 1:** We thank the reviewer for these insightful observations. Regarding the introduction, we acknowledge the importance of providing context for the use of digital management platforms. However, we maintain that it is essential to include a definition of hypertension, especially given the evolving diagnostic criteria over the years. This framing provides necessary context for readers unfamiliar with recent changes. Furthermore, our focus is indeed on mHealth interventions, and we have discussed the relevant management platforms and throughout the results and discussion sections of the systematic review.

Additionally, we have corrected the formatting and titles of all tables to ensure alignment with scientific publication standards and the PLOS ONE formatting guidelines.

***Reviewer #5 Comment 1:*** *The manuscript by Lanka et al addresses the usage of mobile health solutions (mHealth) for hypertension in Africa by means of a careful literature review of 18 articles. Generally the manuscript is clearly written and the arguments fairly sound. However, my major concern is that the authors draw conclusions from on 18 studies. What is more, the manuscript is too detailed.*

**Author Response to Reviewer#5, Comment 1:** We sincerely thank Reviewer #5 for their thoughtful feedback and constructive critique of our manuscript. We appreciate the acknowledgment of the clarity of our writing and the soundness of our arguments.

Regarding the number of included studies, we agree that 18 may appear limited at first glance. However, we want to clarify that the number reflects the outcome of a rigorous, systematic review process guided by established protocols such as the PRISMA (Preferred Reporting Items for Systematic Reviews and Meta-Analyses) guidelines. We also engaged a trained medical librarian to support the development and refinement of our search strategy to ensure comprehensiveness and minimize bias. The 18 studies included in the final analysis represent the current and available peer-reviewed literature that met our strict eligibility criteria, which were developed to ensure relevance, methodological rigor, and focus on mHealth interventions for hypertension management among African populations.

While we acknowledge the concern about the manuscript being too detailed, we believe the level of detail is essential to accurately convey the nuances, methodological variations, and contextual factors that emerged from our review. This depth is particularly important given the diversity of mHealth interventions, populations, and settings across the African continent. Reducing the level of detail could risk oversimplifying the findings and obscuring meaningful distinctions that are critical for informing future research and implementation efforts.

Nonetheless, we remain open to suggestions from the editor and reviewers about specific areas where the text could be condensed without compromising clarity or completeness, and we have revisited the manuscript to streamline redundant sections while preserving the integrity of the findings.

***Reviewer #5 Comment 2:*** *I suggest that the authors relate much of the material from the current manuscript to the supplementary document. For example, Table 1 is neither news nor specific to the manuscript. Therefore, I suggest to simply refer to the relevant reference instead of spelling out the definitions according to reference #17.*

**Author Response to Reviewer#5, Comment 2:** We appreciate Reviewer #5’s thoughtful suggestion to streamline the manuscript by relocating certain material to the supplementary files. With respect to Table 1, we understand the concern regarding its originality and specificity. However, we chose to retain the table in the main manuscript in response to an earlier recommendation from Reviewer #3, who emphasized the value of including Proctor’s implementation outcomes and their definitions to support readers who may be unfamiliar with this terminology. Specifically, Reviewer #3 previously commented (12/17/2024): “I suggest including a table of Proctor’s feasibility outcomes with the definitions to aid reviewers not familiar with the lexicon.”

In line with that feedback, we believe that presenting the table within the main text improves clarity and enhances accessibility for a broader audience, particularly those less familiar with implementation science frameworks. That said, we have carefully reviewed the manuscript for opportunities to reduce redundancy and have ensured that all other supplementary materials are appropriately referenced and structured.

We remain open to further editorial guidance on the placement of this content should the journal prefer it be moved to supplementary materials.

***Reviewer #5 Comment 3:*** *Similarly, there are too many (and too short) subsections in the Methods section.*

**Author Response to Reviewer#5, Comment 3:** We thank Reviewer #5 for this observation regarding the structure of the Methods section. Our decision to organize the Methods section into multiple short subsections was intentional and is consistent with both best practices for systematic reviews and the structure used in our prior peer-reviewed publications.

Systematic reviews typically involve multiple, discrete methodological components—such as search strategy, inclusion and exclusion criteria, data extraction, and quality appraisal—which we believe are best presented in clearly delineated subsections to improve transparency and replicability. This format aligns with established guidelines such as PRISMA, which encourage systematic and structured reporting to facilitate reader comprehension and critical appraisal.

That said, we understand that excessive fragmentation may interrupt the flow of the narrative. In response to this comment, we have revisited the Methods section and tried to consolidate some of the shorter subsections where appropriate, while preserving clarity and alignment with systematic review standards. We hope this revision strikes a better balance between readability and methodological rigor.

We appreciate the reviewer’s feedback and believe the revised version now presents the methods more cohesively without compromising the transparency of our approach.

***Reviewer #5 Comment 4:*** *Tables are generally too many and too detailed. They do not assist the reader in acquiring an overview of the covered material.*

**Author Response to Reviewer#5, Comment 4:** We thank Reviewer #5 for this helpful observation regarding the tables. We acknowledge that the number and level of detail in the tables may seem extensive; however, our intent was to provide a comprehensive summary that would facilitate comparison across studies and enhance the transparency of our review process.

In particular, we included an additional column on study settings (i.e., Urban, Peri-Urban, and Rural) in response to a prior recommendation from Reviewer #1 (Comment 30, 12/17/2024): “It would be helpful to add a column in the table for studies’ settings (Urban/Peri-Urban/Rural). Cite them in the text as well.” This addition was intended to provide readers with important contextual information to better understand the relevance and applicability of the findings across different settings.

That said, we understand the concern about the density of information and potential for reader fatigue. As a result, we have reviewed all tables with a focus on streamlining their content. Where feasible, we consolidated overlapping information. We believe this revised approach improves the balance between comprehensiveness and readability, helping the reader to more efficiently grasp the key findings without losing important context.

***Reviewer #5 Comment 5:*** *Finally, I kindly ask the authors to be more concise and precise at that. For example, it is not entirely clear how the 18 references allow the authors to conclude that mHealth has gained traction across three countries.*

**Author Response to Reviewer#5, Comment 5:** We appreciate this important point and thank Reviewer #5 for calling attention to the need for more careful wording. Upon review, we agree that the original phrasing may have overstated the strength of the evidence regarding the extent of mHealth adoption across countries.

To address this, we have revised the relevant section to ensure that our conclusions are more accurately grounded in the available data. Specifically, we have clarified that while the number of studies is limited, several of them report on mHealth interventions being actively implemented and evaluated in countries such as Nigeria, Ghana, and South Africa. We now describe this as "significant utilization" rather than suggesting broad or definitive adoption.

The revised section (Lines 71–73 in the Conclusion) now reads:
"Application-based mHealth interventions have gained significant utilization in Africa, particularly in countries like Nigeria, Ghana, and South Africa, while SMS-based interventions show promise in reaching non-smartphone users."

We hope this adjustment addresses the reviewer’s concern and better reflects the scope and limitations of our findings.

Regards,

Dr Priyanka Lanka MDS, MPH

NYU School of Global Public Health

[pl2461@nyu.edu](mailto:pl2461@nyu.edu)
