# Supplementary material for: Mobile health interventions for African adults with hypertension: Evidence from a systematic review of implementation research outcomes
Source: PLOS Digit Health. 2026 Aug 14;5(8):e0001612. doi: 10.1371/journal.pdig.0001612 (PMC13475905; doi:10.1371/journal.pdig.0001612)
Supplement: Supplementary file 19 [file pdig.0001612.s018.docx]

**Response to reviewers**

**Dear Editors,**

We would like to express our sincere gratitude for the time and thoughtful attention you and the reviewers have given to our manuscript. The feedback provided was highly constructive and has greatly contributed to improving the depth, organization, and overall quality of our paper.

In this revised submission, we have carefully addressed all reviewer comments through detailed, point-by-point responses. Revisions have been made throughout the manuscript to reflect these suggestions and to strengthen the clarity and coherence of our findings.

We are pleased to resubmit the updated version of our manuscript to PLOS ONE and hope that the revisions meet your expectations for publication. We are deeply appreciative of the reviewers’ and editors’ efforts in guiding this work toward improvement.

Sincerely,

Dr Priyanka Lanka, MDS, MPH

NYU School of Global Public Health

[pl2461@nyu.edu](mailto:pl2461@nyu.edu)

**Reviewer 1 Comment 1: Suggested Reformulation of Objectives: General Objective: To analyze the acceptability, feasibility, and adoption of mobile health (mHealth) interventions for the management of hypertension in adults in Africa through a systematic review.**

**Author Response to Comment 1:** Thank you for this thoughtful input. In alignment with this comment, we have revised our review to explicitly frame the analysis through Proctor’s implementation research outcomes, with a focus on **acceptability, feasibility, and adoption**. In doing so, we identified a gap in the literature, as only three of the eight outcomes were clearly defined. To better reflect this emphasis, we have updated the manuscript and revised the title to: “Mobile Health Interventions for Hypertension Management in African Adults: Evidence from a Systematic Review of Implementation Research Outcomes.”.

**Reviewer 1 Comment 2: Methodological Issues: The study is registered as a systematic review, but although the registration date is mentioned, the full search strategy is not attached.**

**Author Response to Comment 2:** We thank the reviewer for this comment. The full search strategy was previously uploaded as part of the supplementary files. However, we have noticed a preview issue with the DOCX format, which restricts viewing in the platform unless the file is downloaded. To address this, we have now re-submitted the files in both DOCX and PDF formats to ensure ease of access and review.

**Reviewer 1 Comment 3: The search strategy is extensive; however, the manuscript does not report whether double reviewers were used or how the review process was carried out (e.g., selection, conflict resolution).**

**Author Response to Comment 3:** We thank the reviewer for this important comment. We would like to clarify that a detailed description of our screening process has been included in the Methods section. This description specifies that all studies were independently screened by two reviewers, whose initials are provided for transparency. The Methods section also outlines the procedures for study selection, including how disagreements between reviewers were identified and resolved through discussion or consultation with a third reviewer when necessary. By providing these details, we aim to ensure transparency and reproducibility of our review process, and to demonstrate that the review adhered to rigorous methodological standards.

**Reviewer 1 Comment 4: The justification for excluding pregnant women is weak, especially given the high rate of hypertensive disorders in pregnancy throughout Africa. A justification such as ethical concerns or the transient nature of the condition could strengthen this decision.**

**Author Response to Comment 4:** We excluded pregnant women from our study because hypertension in this population is most commonly associated with pre-eclampsia, a condition with distinct underlying mechanisms and contributing factors that differ from those of primary (essential) hypertension. Pre-eclampsia is a complex, pregnancy-specific disorder influenced by placental, vascular, and immunologic factors, which are not typically present in the general hypertensive population. Additionally, hypertension in the context of pre-eclampsia is often transient, usually resolving after delivery, and therefore does not reflect the chronic patterns of blood pressure regulation and cardiovascular risk observed in the broader adult population. Given these differences in etiology, clinical course, and management, including pregnant women could have introduced heterogeneity that might confound our analyses. For these reasons, this population was excluded to ensure the study focused specifically on primary hypertension and maintained methodological consistency with our inclusion criteria.

**Reviewer 1 Comment 5: Interobserver reliability (e.g., kappa coefficient) for risk of bias assessment is not reported.**

**Author Response to Comment 5:** We thank the reviewer for raising this important point. The kappa coefficient is a widely used statistical measure that quantifies the degree of agreement between reviewers beyond what would be expected by chance. In systematic reviews, it is commonly applied to demonstrate interobserver reliability when multiple reviewers independently assess study quality or risk of bias. In our review, two reviewers independently assessed the risk of bias for each included study. Any initial discrepancies were resolved through discussion, and, when necessary, consultation with a third reviewer. Our review protocol emphasized a consensus-based approach, whereby final judgments were reached collaboratively rather than relying solely on individual assessments. This approach aligns with the PRISMA 2020 statement (Page et al., BMJ 2021; citation 18 in our manuscript), which notes that while reporting interrater agreement can be informative, it is not a mandatory requirement when consensus methods are used to finalize risk of bias assessments. To enhance transparency, we have now clarified this process in the Methods section (see Data Extraction section). We utilized Covidence, which automatically calculates kappa coefficients, and have provided an Excel file as supplementary material containing the calculated kappa coefficients for each reviewer comparison (Supplemental Table 2: Covidence Inter-rater Reliability Scores). The kappa values ranged from 0.372 to 0.702, reflecting fair to substantial agreement between reviewers prior to consensus discussions. In summary, while consensus-based assessment was the primary approach in our review, the inclusion of kappa coefficients provides additional evidence of the reliability of our risk of bias evaluation. These data further reinforce the rigor, reproducibility, and transparency of our methodological process, giving readers confidence in the validity of our findings.

**Reviewer 1 Comment 6: The use of the ASSESS tool is appropriate, but there is no detailed discussion of quality by criterion. It is recommended that a table of methodological limitations be added per study.**

**Author Response to Comment 6:** We sincerely thank the reviewer for this insightful and constructive feedback. In response, we have prepared a detailed Excel sheet summarizing the methodological limitations of each included study using the ASSESS criteria (based on the MMAT- mixed methods assessment tool). We propose to include this Excel sheet as supplemental material (Supplemental Table 3: Risk of Bias Assessment) alongside the manuscript, which will allow readers to access comprehensive, study-specific information and enhance transparency in our systematic review process. By providing this supplemental table, we aim to give readers a clear understanding of how each study was assessed, the specific methodological strengths and weaknesses identified, and the criteria underlying our evaluations. Furthermore, to ensure that key findings regarding study limitations are clearly communicated, we have highlighted the most critical limitations within the text just prior to the Conclusion section. This approach provides a concise yet informative overview of the limitations, ensuring that readers gain an immediate understanding of potential biases or methodological concerns without the need to consult supplemental materials exclusively. We believe that this dual approach—providing a detailed supplemental table while summarizing key limitations in the main text—strikes an appropriate balance between transparency, comprehensiveness, and readability. It not only strengthens the reproducibility of our review but also ensures that readers can critically appraise our methodology and findings. Overall, we are confident that these additions enhance the rigor of the manuscript and support an open, transparent discussion of the strengths and limitations of the included studies of the limitations without significantly increasing the length of the manuscript.

**Reviewer 1 Comment 7: Results should be more clearly linked to specific studies; currently, study-level findings are inconsistently described. There is no exploration of variations between rural and urban settings, which is essential given the digital divide in African contexts.**

**Author Response to Comment 7:** We would like to clarify that study-specific outcomes were consistently reported in Table 3, where similarities and differences between studies are highlighted. In response to this comment, we have further elaborated in the Results section to ensure clearer linkage between findings and individual studies. Additionally, we have expanded our analysis to include distinctions between rural and urban settings, thereby addressing the digital divide in African contexts and providing greater contextual understanding.

**Reviewer 1 Comment 8: All outcomes are presented with equal weight, without distinguishing which interventions demonstrated greater clinical or implementation benefits. The review reports perceived changes in adherence, but there is no assessment of hard clinical outcomes, such as blood pressure control or cardiovascular events.
No follow-up data or duration of effect are discussed or analyzed. Clearly specify the clinical outcomes assessed, including the duration of hypertension, the magnitude of improvement in adherence, and any clinically significant blood pressure reduction.**

**Author Response to Comment 8:** We sincerely thank the reviewer for this thoughtful and detailed comment. We would like to clarify that the primary focus of our systematic review was on **implementation research (IR) outcomes**, as outlined in the Introduction and guided by the Proctor et al. (2011) framework (citation 17 in our manuscript). IR outcomes differ from clinical outcomes in that they assess how evidence-based interventions are delivered and integrated into practice. Specifically, IR outcomes include measures such as feasibility, acceptability, fidelity, adoption, appropriateness, penetration, and costs. These outcomes are critical to understanding whether evidence-based interventions can be successfully implemented in real-world settings, particularly among diverse and resource-limited populations. It is important to distinguish that IR outcomes are not designed to evaluate the efficacy of interventions on “hard” clinical endpoints, such as blood pressure control or cardiovascular events, since the evidence base for their clinical efficacy has already been established. Instead, our review was designed to determine whether these interventions could be effectively adopted and sustained in practice.

That said, we agree with the reviewer that clinical outcomes are important for context and transparency. In response, we revisited the included publications and extracted available data on clinical outcomes, such as changes in systolic blood pressure (SBP) and diastolic blood pressure (DBP). We have summarized these findings in an Excel file that is now provided as supplementary material (Supplemental Table 1: Clinical assessment of Blood Pressure from Studies). These data offer additional context regarding the duration of follow-up, and clinically meaningful reductions in blood pressure where reported.

In the manuscript, we have clarified the distinction between implementation research outcomes (Table 1) and clinical outcomes (Supplemental Table 1: Clinical assessment of Blood Pressure from Studies). We intentionally chose not to add a narrative summary of the extracted clinical outcomes within the main text, as we felt this would shift the emphasis away from the central focus of the review—implementation research. Several recent reviews have already provided comprehensive analyses of clinical outcomes for mHealth interventions in hypertension management (e.g., Zhou et al., 2023; Hamza et al., 2025). By focusing on IR outcomes, our review complements this existing body of work and provides novel insights into the feasibility, adoption, and sustainability of mHealth interventions. Readers who wish to explore clinical endpoints further are directed to the supplemental material, where extracted clinical data are presented in detail, ensuring both transparency and completeness without detracting from the primary aims of the review.

Importantly, while clinical outcomes of mHealth interventions for hypertension are increasingly well documented, there remains a critical gap in understanding how and under what conditions these interventions can be successfully implemented, scaled, and sustained in diverse contexts. Our review directly addresses this gap by systematically synthesizing implementation outcomes, thereby offering a perspective that extends beyond efficacy to inform real-world application and policy translation.

**Reviewer 1 Comment 9: The table of barriers and facilitators, although useful, is too long and not ranked. For example, facilitators such as increased mobile phone use are mentioned, but they are not categorized according to their level of impact.**

**Author Response to Comment 9:** We thank the reviewer for this insightful comment. We feel that the current table is appropriate in providing a comprehensive overview of the barriers and facilitators reported across the included publications. Ranking these factors would require us to make subjective judgments, as the studies varied in design, intervention type, and context (as detailed in Table 3). Given this heterogeneity, it would be difficult to assign relative levels of impact across such diverse settings.

**Reviewer 1 Comment 10: Consider reorganizing the barriers and facilitators into shorter, thematically grouped tables, and indicate how many studies reported each item.**

**Author Response to Comment 10:** We appreciate the reviewer’s thoughtful suggestion to improve the clarity of how barriers and facilitators are presented. In response, we have reorganized the original table into two separate tables—Table 4a (Facilitators) and Table 4b (Barriers)—to allow for easier interpretation and comparison. To further enhance transparency and usability, we have provided corresponding references and inserted citations directly within the tables. We believe these revisions improve the readability of the results, strengthen the presentation of key findings, and make it easier for readers to identify recurring themes across the included studies.
