# Supplementary material for: Mobile health interventions for African adults with hypertension: Evidence from a systematic review of implementation research outcomes
Source: PLOS Digit Health. 2026 Aug 14;5(8):e0001612. doi: 10.1371/journal.pdig.0001612 (PMC13475905; doi:10.1371/journal.pdig.0001612)
Supplement: Supplementary file 20 [file pdig.0001612.s019.docx]

**December 10, 2025**

***Manuscript Title*: Mobile Health Interventions for Hypertension Management in African Adults: Evidence from a Systematic Review of Implementation Research Outcomes**

**Response to Reviewers and Editors’ Previous Comments**

We sincerely thank the reviewers and the editorial team for their thoughtful, detailed, and constructive feedback throughout this extensive review process. We deeply appreciate the time, expertise, and careful evaluation devoted to improving our manuscript. The reviewers’ insights have strengthened the methodological rigor, clarity, and overall quality of our work, and we are grateful for this continued engagement.

We also recognize that, across the multiple rounds of revision, new points for clarification have emerged. We have carefully revisited each comment and have provided below a comprehensive, point-by-point response, reaffirming where previous revisions addressed earlier concerns and elaborating further where needed.

### **__________________________________________________________________________________________**

### **Clarification Regarding Reviewer 1 Comment 4 (Pregnant Women Exclusion)**

In the September 2025 revision, Reviewer 1 requested stronger justification for excluding pregnant women. We provided this explanation in our prior response, and we reiterate it here for completeness and transparency:

**Author Response to Comment 4:** We excluded pregnant women because hypertension arising during pregnancy—primarily pre-eclampsia—has distinct etiologic mechanisms, clinical trajectories, and management pathways that differ fundamentally from primary hypertension. These differences include placental and immunologic contributors and the generally transient nature of pregnancy-related hypertension, which typically resolves postpartum. Including this population would have introduced substantial heterogeneity and confounded interpretation of primary hypertension–focused implementation outcomes. Therefore, this exclusion criterion ensured methodological consistency and conceptual clarity within the review.

### **__________________________________________________________________________________________**

### **Reviewer 1 Comment 5: Interobserver Reliability**

We appreciate the reviewer’s emphasis on transparency regarding risk of bias assessment. As detailed previously:

**Author Response to Comment 5:** Two reviewers independently assessed risk of bias, resolving discrepancies through discussion or a third reviewer when needed—an approach consistent with PRISMA 2020 guidance, which does not mandate reporting kappa statistics for consensus-based reviews. However, in response to the reviewer’s request, we have now included Covidence-generated kappa coefficients as **Supplemental Table 2**, ranging from 0.372 to 0.702 (fair to substantial agreement). We have also clarified our process in the Methods section.

These additions strengthen transparency and demonstrate the reliability of our evaluative procedures.

### **__________________________________________________________________________________________**

### **Reviewer 1 Comment 8: Equal Weight of Outcomes and Lack of Clinical Endpoints**

We appreciate the reviewer’s interest in integrating clinical outcomes; however, we emphasize that:

**Author Response to Comment 8:**The central aim of this review was to assess **implementation research (IR) outcomes**, as framed by the Proctor et al. (2011) taxonomy. IR outcomes—such as acceptability, adoption, feasibility, fidelity, and penetration—are conceptually distinct from clinical outcomes and are not intended to establish intervention efficacy. Nonetheless, in response to the reviewer’s concerns, we extracted and summarized available clinical outcomes (e.g., SBP/DBP changes, follow-up duration), which are now included in **Supplemental Table 1**. We elected not to integrate these into the main narrative to avoid shifting emphasis away from the core implementation-focused objectives of the review. Several recent systematic reviews have comprehensively evaluated clinical effects of mHealth interventions for hypertension; our work complements these efforts by addressing the less-explored, yet highly consequential, question of real-world implementation feasibility, sustainability, and system readiness.

This clarification is now more explicitly stated in the manuscript.

### **__________________________________________________________________________________________**

### **Reviewer 1 Comment 9: Length and Ranking of Barriers and Facilitators**

**Author Response to Comment 9:** We appreciate the suggestion to rank barriers and facilitators but believe such ranking would require subjective weighting across heterogeneous study contexts, intervention types, and health system environments. Given this variability, and to avoid introducing unintended bias, we elected to retain a descriptive and comprehensive approach.

### **__________________________________________________________________________________________**

### **Reviewer 1 Comment 10: Reorganization of Barriers and Facilitators**

**Author Response to Comment 10:** We agree that restructuring the presentation would enhance clarity. In response, we reorganized the material into two separate tables—**Table 4a (Facilitators)** and **Table 4b (Barriers)**—and added citations directly within the tables to allow readers to easily identify the source studies.

### **__________________________________________________________________________________________**

### **Closing Statement**

As demonstrated in the responses above, we have carefully and comprehensively addressed each comment raised across the review cycles. While we believe that the manuscript has already undergone substantial strengthening through several rounds of iterative revision, we welcome the opportunity provided by this most recent review to further clarify methodological choices, enhance transparency, and refine the organization of key sections.

At this point, we are confident that the manuscript represents a rigorous, clearly articulated, and well-balanced synthesis that aligns with the stated aims of the PLOS DH and the conventions of implementation science research. We respectfully submit the revised manuscript which is now suitable for acceptance.

We again extend our sincere thanks to the reviewers and editors for their thoughtful engagement and valuable contributions to improving this work.

**Revision - 5**

**Reviewer Comment 1: Lack of reporting of the kappa coefficient. Although the authors state in their response that Covidence generated kappa values, these are never described or interpreted in the manuscript. Inter-observer reliability was specifically requested by reviewers, and its absence must be considered a methodological omission that requires correction.**

***Authors Response to Reviewer Comment 1:* Lack of Reporting of the Kappa Coefficient**

We appreciate the reviewer’s thoughtful attention to inter-observer reliability and the request for clarification regarding the reporting of the kappa coefficient. As described in our earlier response, Covidence did generate kappa statistics during both the title/abstract and full-text screening phases. **This table was submitted with the last revision (see Response to Reviewers and Editors’ Previous Comments above).** However, after reviewing methodological guidance and reflecting on the interpretive challenges inherent to kappa, we made an intentional decision not to report these values in the manuscript **but only as a supplemental table.**

Although the kappa coefficient is used, it carries several significant and well-recognized limitations. As Dettori and Norvell (2020) highlight,[1] kappa can produce paradoxically low values even when reviewers demonstrate high absolute agreement. This issue, commonly known as the “kappa paradox,” was first systematically described by Feinstein and Cicchetti (1990) [2], who showed that kappa values can be distorted when category prevalence is either very high or very low. Under these circumstances, the statistic may underestimate true reviewer agreement, thereby misrepresenting the reliability of the screening process [3]. These paradoxical behaviors make kappa particularly difficult to interpret in systematic review screening, where inclusion/exclusion decisions often display strong prevalence imbalance.

In light of these methodological concerns, experts—including Dettori and Norvell (2020) [1]—recommend the use of more robust and prevalence-insensitive metrics such as Gwet’s AC1. Because AC1 provides a more stable and accurate measure of inter-rater agreement, its use is increasingly encouraged in screening contexts. Consistent with this methodological guidance, our review prioritized consensus-based decision-making within Covidence, whereby all reviewer disagreements were discussed and resolved using a structured adjudication process. This approach ensures transparency, rigor, and reliability, even without relying on potentially misleading kappa values.

For these reasons—and consistent with the methodological decisions in our previously published systematic and scoping reviews—we elected not to report kappa statistics in either the Methods or Results sections. Specifically, none of our prior reviews (references 4–11) have included kappa values due to the same interpretive limitations documented in the methodological literature. This decision reflects a deliberate and consistent approach to ensuring that reported reliability metrics enhance clarity rather than introduce confusion.

Nevertheless, in the interest of transparency and to directly address the reviewer’s request, we have included a supplementary table presenting the kappa output generated by Covidence. These values demonstrate substantial inter-observer agreement but, for the reasons described above, are not included as primary indicators of screening reliability. We hope this expanded explanation fully clarifies our rationale and satisfies the reviewer’s concern.

### **References**

**Methodological References**

1. **Dettori JR, Norvell DC.** Kappa and Beyond: Is There Agreement? Global Spine Journal. 2020;10(4):499–501.
2. **Feinstein AR, Cicchetti DV.** High agreement but low kappa: I. The problems of two paradoxes. Journal of Clinical Epidemiology. 1990;43:543–549.
3. **Cohen J.** Weighted kappa: nominal scale agreement with provision for scaled disagreement or partial credit. Psychological Bulletin. 1968;70:213–220.

**Previously Published Systematic and Scoping Reviews (Consistently Not Reporting Kappa)**

4. **Karbasi AB, Iloegbu C, Ruan C, Osei-Tutu N, Patel K, Frerichs L, Patena J, Vieira D, Adenikinju D, Samuels L, Gyamfi J, Peprah E.** Syndemic interactions between HIV/AIDS, mental health conditions, and non-communicable diseases in sub-Saharan Africa: A scoping review of contributing factors. PLoS One. 2025;20(8):e0328515.
5. **Patena J, Adenikinju D, Lanka P, Hameed T, Kulkarni S, Osei-Tutu N, Zuniga S, Ruan C, Shenoy S, Thakkar D, Noble E, Angulo B, Vieira D, Gyamfi J, Peprah E.** Evaluating implementation research outcomes for a task-sharing mental health intervention: A systematic review of the Friendship Bench. Global Mental Health. 2025;12:e65.
6. **Noble E, Moinul D, Khairy Djim Sylla O, Friedmann S, Amick K, Rowhani N, Dua R, Mannan N, Seaman C, Ayo O, Pant S, Osoko O, Gogineni S, Malburg C, Dickey C, Peprah E.** Downward accountability mechanism effectiveness by non-governmental organizations in low- and middle-income countries: A qualitative systematic review. PLoS One. 2025;20(5):e0324098.
7. **Meda S, Gyamfi J, Patel K, Islam F, Vieira D, Nyame S, Ruan C, Boradia K, Chhetri H, Thunga S, Adenikinju D, Amesimeku E, Diawara A, Opeyemi J, Ojo T, Chirinos C, Williams O, Ogedegbe O, Peprah E.** Assessment of musical interventions and its effect on blood pressure among United States populations: a systematic review and meta-analysis. Frontiers in Cardiovascular Medicine. 2024;11:1405455.
8. **AlRashed H, Miele J, Prasad J, Adenikinju D, Iloegbu C, Patena J, Vieira D, Gyamfi J, Peprah E.** Systematic review of end stage renal disease in Pakistan: Identifying implementation research outcomes. PLoS One. 2023;18(12):e0296243.
9. **Shenoy S, Sharma P, Rao A, Aparna N, Adenikinju D, Iloegbu C, Patena J, Vieira D, Gyamfi J, Peprah E.** Evidence-based interventions to reduce maternal malnutrition in low and middle-income countries: a systematic review. Frontiers in Health Services. 2023;3:1155928.
10. **Fitzpatrick R, Pant S, Li J, Ritterman R, Adenikinju D, Iloegbu C, Patena J, Vieira D, Gyamfi J, Peprah E.** Implementation of non-insulin-dependent diabetes self-management education (DSME) in LMICs: a systematic review of cost, adoption, acceptability, and fidelity in resource-constrained settings. Frontiers in Health Services. 2023;3:1155911.
11. **Karbasi A, Fordjuoh J, Abbas M, Iloegbu C, Patena J, Adenikinju D, Vieira D, Gyamfi J, Peprah E.** An Evolving HIV Epidemic in the Middle East and North Africa (MENA) Region: A Scoping Review. International Journal of Environmental Research and Public Health. 2023;20(5):3844.

**Table 1. Covidence Kappa Statistic for Inter-observer Reliability**

**Reviewer Comment 2. Lack of study-level results. While the authors mention that individual study outcomes are presented “in tables,” the narrative text continues to rely on general statements without indicating which study supports which findings. This narrative aggregation makes the analysis more difficult to follow. It is suggested to highlight key individual results directly in the text or incorporate them more clearly into the existing tables.**

***Authors Response to Reviewer Comment 2:* Lack of Study-Level Results**

We appreciate the reviewer’s emphasis on the clarity of study-level reporting. We respectfully note, however, that the manuscript already includes study-specific detail in both the narrative text and the accompanying tables.

In the Characteristics of Identified Studies section (e.g., **Geographical Distribution and Setting**; **Target Population and Health Focus**), we explicitly cite individual studies to anchor each descriptive statement. Similarly, within the Results section, study-level findings are consistently linked to in-text citations and corresponding table entries. Tables were intentionally designed to present detailed study outcomes in a structured and accessible format to reduce redundancy in the narrative and to enhance readability. As a result, the narrative summarizes patterns across studies while the tables provide the full granularity of individual study results.

We acknowledge the reviewer’s preference for more extensive narrative detail; however, we believe the current balance between narrative synthesis and table-based reporting aligns with standard methodological guidance for systematic and umbrella reviews, which emphasizes avoiding unnecessary repetition of information already contained in tables. Adding additional study-level descriptions directly in the text would risk duplication and reduce the manuscript’s clarity.

Nonetheless, in response to the reviewer’s concern, we have revisited the manuscript to ensure that key findings are clearly referenced and that study-level examples are highlighted where this enhances comprehension. We trust that these refinements will improve the flow of the Results section while maintaining methodological rigor and avoiding redundancy.

Ultimately, we defer to the editor regarding stylistic preferences for the degree of narrative versus table-based detail.

**Reviewer Comment 3. Insufficient justification for excluding pregnant women. The added explanation regarding hypertensive disorders of pregnancy does not fully resolve the original methodological concern. Clear exclusion criteria are required. The manuscript needs to justify the exclusion based not only on the distinct pathophysiology of pregnancy but also on the transient course of gestational hypertension and the need for different evaluation frameworks—criteria that align with systematic review standards rather than broad clinical arguments.**

***Authors Response to Reviewer Comment 3:* Insufficient Justification for Excluding Pregnant Women**

We *sincerely* appreciate the reviewer’s ongoing attention to the precision and clarity of our eligibility criteria. **In our previous response we submitted justification for exclusion of pregnant (see Response to Reviewers and Editors’ Previous Comments).** We expand on that explains specially that the exclusion of studies involving pregnant women was deliberate and grounded in established clinical distinctions as well as methodological standards fundamental to high-quality systematic reviews. We agree that the rationale must extend beyond broad clinical differences, and we welcome the opportunity to further articulate and strengthen our justification.

Hypertensive disorders of pregnancy—including gestational hypertension, preeclampsia, and related conditions—constitute a clinically and epidemiologically distinct category of hypertensive disease. These disorders differ fundamentally from chronic or essential hypertension among non-pregnant adults in ways that directly affect the comparability of populations, outcomes, and intervention strategies. Our rationale for exclusion is based on the following considerations:

1. **Distinct Pathophysiology:**
   Hypertensive disorders of pregnancy emerge after 20 weeks’ gestation and typically resolve following delivery. Their underlying pathophysiology, risk profiles, diagnostic thresholds, and disease trajectory differ substantially from chronic hypertension in the general adult population [1–3]. Because these conditions necessitate tailored diagnostic and therapeutic frameworks, combining these populations would conflate fundamentally different disease processes and undermine the interpretability of the review.
2. **Transient and Pregnancy-Specific Course:**
   Unlike chronic hypertension, hypertensive disorders of pregnancy are time-limited and closely tied to gestational physiology. Their incidence, clinical course, and outcomes are intrinsically pregnancy-dependent, making them conceptually misaligned with the long-term, persistent nature of chronic hypertension. Including these studies would introduce substantial clinical heterogeneity and shift the conceptual focus of the review away from chronic hypertension, thereby limiting the validity of cross-study comparisons [4,5].
3. **Different Intervention and Evaluation Frameworks:**
   Evidence-based guidelines, screening protocols, and management strategies for pregnancy-related hypertension are designed to optimize maternal–fetal outcomes rather than adult chronic disease outcomes. Interventions involve unique risk–benefit considerations, monitoring strategies, and outcome metrics that are not directly comparable to standard hypertension management in non-pregnant adults. Including pregnancy-specific interventions would therefore compromise methodological coherence and weaken the interpretability of synthesized findings [3].
4. **Alignment With Systematic Review Standards:**
   In accordance with PRISMA recommendations and systematic review methodological standards, we prespecified our target population as **adults with chronic or primary hypertension** [5,6]. Clear delineation of the population of interest is essential for mitigating unwarranted heterogeneity. Including studies of hypertensive disorders of pregnancy—conditions that differ in pathophysiology, treatment paradigms, and outcome structures—would violate these methodological principles and weaken the internal consistency of the review [4,6].

Finally, our decision to exclude studies involving pregnant women is consistent with prior hypertension research conducted by our team, as well as with standard epidemiologic and clinical conventions in the hypertension literature [4,6]. Our intent is not to minimize the importance of hypertensive disorders of pregnancy—which are critical global health concerns—but rather to ensure that the review remains scientifically coherent, internally valid, and aligned with its stated objectives.

**References**

1. Gestational Hypertension and Preeclampsia: ACOG Practice Bulletin Summary, Number 222. Obstet Gynecol. 2020 Jun;135(6):1492-1495. doi: 10.1097/AOG.0000000000003892. PMID: 32443077.
2. Hutcheon JA, Lisonkova S, Joseph KS. Epidemiology of pre-eclampsia and the other hypertensive disorders of pregnancy. Best Pract Res Clin Obstet Gynaecol. 2011 Aug;25(4):391-403. doi: 10.1016/j.bpobgyn.2011.01.006. Epub 2011 Feb 18. PMID: 21333604.
3. Henderson JT, Webber EM, Vesco KK, et al. Screening for Hypertensive Disorders of Pregnancy: An Evidence Update for the U.S. Preventive Services Task Force [Internet]. Rockville (MD): Agency for Healthcare Research and Quality (US); 2023 Sep. (Evidence Synthesis, No. 227.) Chapter 2, Methods. Available from: https://www.ncbi.nlm.nih.gov/books/NBK595106/
4. Gyamfi J, Vieira D, Iwelunmor J, Watkins BX, Williams O, **Peprah E,** Ogedegbe G, Allegrante JP. Assessing descriptions of scalability for hypertension control interventions implemented in low-and middle-income countries: A systematic review. PLoS One. 2022 Jul 28;17(7):e0272071. doi: 10.1371/journal.pone.0272071. PMID: 35901114; PMCID: PMC9333290.
5. Mensah GA, Fuster V, Murray CJL, Roth GA; Global Burden of Cardiovascular Diseases and Risks Collaborators. Global Burden of Cardiovascular Diseases and Risks, 1990-2022. J Am Coll Cardiol. 2023 Dec 19;82(25):2350-2473. doi: 10.1016/j.jacc.2023.11.007. PMID: 38092509; PMCID: PMC7615984.
   - **E. Peprah is a member of the GBD Global Burden of Cardiovascular Diseases and Risks Collaborators.**
6. Akpa OM, Made F, Ojo A, Ovbiagele B, Adu D, Motala AA, Mayosi BM, Adebamowo SN, Engel ME, Tayo B, Rotimi C, Salako B, Akinyemi R, Gebregziabher M, Sarfo F, Wahab K, Agongo G, Alberts M, Ali SA, Asiki G, Boua RP, Gómez-Olivé FX, Mashinya F, Micklesfield L, Mohamed SF, Nonterah EA, Norris SA, Sorgho H, Tollman S, Parekh RS, Chishala C, Ekoru K, Waddy SP, **Peprah E**, Mensah GA, Wiley K, Troyer J, Ramsay M, Owolabi MO; as members of the CVD Working Group of the H3Africa Consortium. Regional Patterns and Association Between Obesity and Hypertension in Africa: Evidence From the H3Africa CHAIR Study. Hypertension. 2020 May;75(5):1167-1178. doi: 10.1161/HYPERTENSIONAHA.119.14147. Epub 2020 Mar 16. PMID: 32172619; PMCID: PMC7176339.

**Reviewer Comment 4. Lack of comparative analysis between urban and rural contexts. The manuscript briefly discusses the digital divide and the scarcity of rural studies, yet the integration of these observations into the Results and Discussion is limited and lacks methodological justification. A clearer comparative analysis is needed.**

***Author Response to Reviewer Comment 4:* Lack of Comparative Analysis Between Urban and Rural Contexts**

We sincerely appreciate the reviewer’s interest in a more explicit comparison between urban and rural contexts, and we agree that the digital divide and uneven geographic distribution of studies are essential considerations when interpreting the findings of this review. These contextual factors have direct implications for feasibility, equity, and scalability of mHealth interventions for hypertension management.

However, our ability to conduct a formal comparative analysis was fundamentally constrained by the available evidence (see Table 3. Characteristics of the Studies). As documented in the manuscript, only **three studies** were conducted in rural settings, while the vast majority were situated in urban or peri-urban areas. This marked imbalance results in insufficient representation of rural populations and a lack of comparable intervention types, outcome measures, and implementation frameworks across settings. Systematic review methodological standards advise against drawing comparative inferences when data are sparse, highly uneven, or lack conceptual equivalence, as doing so could introduce bias and overinterpretation. For these reasons, a formal urban–rural comparison would not be methodologically sound.

Despite these limitations, we agree that the geographic context merits explicit discussion. In response to the reviewer’s suggestion, we have strengthened the manuscript in several key ways:

1. **Clarified and Expanded Reporting of the Urban–Rural Distribution:**
   We clearly describe the geographic classification of included studies, specifying the number conducted in urban, peri-urban, rural, or mixed settings (see lines 276-281, page 13). This provides readers with a transparent overview of the distribution and highlights where evidence is most and least developed.
2. **Expanded Discussion of the Implications of Geographic Imbalance:**
   We have substantially enriched the Discussion section to address how the predominance of urban studies affects generalizability, particularly for rural and resource-limited contexts (see Strengths and Limitations). This includes attention to disparities in digital access, mobile network reliability, device ownership, and healthcare infrastructure—factors that may limit the applicability and effectiveness of mHealth interventions in underserved rural communities. We also discuss how this imbalance could exacerbate existing health inequities if not addressed in future research and implementation planning.
3. **More Explicit Acknowledgment of Methodological Limitations:**
   Within the Strengths and Limitations section (lines 508-533), we explicitly highlight how the lack of rural studies restricts our ability to draw meaningful comparisons or make robust inferences about differential effectiveness across geographic contexts. We also note that this limitation reflects broader gaps in the literature and underscores the need for targeted rural-focused implementation research.

Together, these revisions reinforce the narrative connection between the geographic distribution of the evidence and its implications for intervention scalability, equity, and policy relevance. While a formal comparative analysis is not feasible given the limited and uneven evidence base, the revised manuscript now more thoroughly addresses the importance of geographic context and articulates its meaning for both research and practice.

We hope these additions fully address the reviewer’s concern and strengthen the manuscript’s contribution to the field.

**Reviewer Comment 5. Limited discussion of the sustained effect of interventions. Although supplementary tables include clinical data, the manuscript does not explicitly state that the absence of long-term follow-up limits the interpretation of implementation outcomes. This point must be articulated to contextualize the findings appropriately.**

***Author Response to Reviewer Comment 5:* Limited Discussion of the Sustained Effect of Interventions**

We appreciate the reviewer’s attention to the issue of sustainability, as the long-term impact of digital health interventions is an important consideration for implementation science. However, we would like to clarify the conceptual boundaries of the present review and the nature of the evidence available.

This manuscript is explicitly focused on **implementation outcomes**, as defined by Proctor et al. (2010) [1] including acceptability, feasibility, adoption, fidelity, cost, penetration, and sustainability as reported in the included studies. Our goal was not to evaluate the clinical effectiveness or *long-term clinical impact of interventions*, but rather to **synthesize how implementation processes and determinants have been assessed and reported in studies involving adults with hypertension in the African context**.

The reviewer’s reference to “long-term follow-up” aligns more closely with **clinical outcome evaluation [2]**—such as sustained blood pressure reduction or recurrence of hypertension—rather than implementation research outcomes. Because long-term clinical trajectories are outside the scope of implementation-focused reviews, they were not included as primary or secondary outcomes in our protocol.

That said, we recognize the value of clarifying the limitations of the evidence base. To address the reviewer’s concern, we have strengthened the manuscript by:

1. **Explicitly noting in the Discussion that most included studies had short implementation periods**, with limited reporting on sustainability or long-term maintenance of intervention use.
2. **Directing readers to Table 3**, which already summarizes study duration and highlights the variability and often short time frames of included interventions.
3. **Adding a statement emphasizing that conclusions regarding long-term sustainability are inherently limited by the short duration of most studies**, a common constraint in digital health implementation research.

These revisions ensure that the reader understands how the limited follow-up reporting affects interpretation without implying that the review sought to evaluate clinical outcomes beyond its methodological scope.

We hope this expanded clarification resolves the reviewer’s concern and strengthens the transparency of the manuscript.

**Reference**

1. Proctor, E., et al., *Outcomes for implementation research: conceptual distinctions, measurement challenges, and research agenda.* Adm Policy Ment Health, 2011. 38(2): p. 65-76.
2. Walton MK, Powers JH 3rd, Hobart J, Patrick D, Marquis P, Vamvakas S, Isaac M, Molsen E, Cano S, Burke LB; International Society for Pharmacoeconomics and Outcomes Research Task Force for Clinical Outcomes *Assessment. Clinical Outcome Assessments: Conceptual Foundation-Report of the ISPOR Clinical Outcomes Assessmen*t - Emerging Good Practices for Outcomes Research Task Force. Value Health. 2015 Sep;18(6):741-52. doi: 10.1016/j.jval.2015.08.006. Epub 2015 Aug 24. PMID: 26409600; PMCID: PMC4610138.

**Reviewer/Editorial Comment 6: Overall, the manuscript shows a solid methodological foundation and clear progress in addressing earlier concerns. Nevertheless, minor revisions are still needed to improve transparency, strengthen methodological justification, and enhance the clarity of the narrative synthesis. With these adjustments, the manuscript would be suitable for publication.**

***Author Response to Comment 6:*** We sincerely appreciate the reviewer’s thoughtful assessment and constructive feedback. We are encouraged that the manuscript is viewed as having a solid methodological foundation and that our revisions to date have meaningfully addressed earlier concerns. In response to the reviewer’s suggestion for additional refinements, we have implemented several targeted revisions to further enhance transparency, strengthen methodological justification, and improve the clarity and coherence of the narrative synthesis.

Specifically, we have:

1. **Enhanced methodological transparency** by clarifying key elements of our search strategy, eligibility criteria, and analytic framework to ensure that each step of the review process is explicitly described (see Authors Response to Reviewer Comment 1 - 4) .
2. **Strengthened justification for methodological choices**—including population definitions, intervention categorizations, and synthesis decisions—by providing clearer rationale and citing relevant methodological guidance where appropriate (see Author Response to Reviewer Comment 5).
3. **Refined the narrative synthesis** to improve readability and conceptual flow, ensuring that themes are presented more cohesively and that findings are directly linked to the review objectives (see edits throughout the manuscript).

We believe these revisions address the reviewer’s remaining concerns and further improve the rigor and clarity of the manuscript. We thank the reviewer again for their careful evaluation and constructive input, which have substantially strengthened the final submission.
