# Supplementary material for: Mobile health interventions for African adults with hypertension: Evidence from a systematic review of implementation research outcomes
Source: PLOS Digit Health. 2026 Aug 14;5(8):e0001612. doi: 10.1371/journal.pdig.0001612 (PMC13475905; doi:10.1371/journal.pdig.0001612)
Supplement: Supplementary file 21 [file pdig.0001612.s020.docx]

**Response to reviewers**

**Dear Editors,**

We would like to sincerely thank you and the reviewers for your continued time, care, and thoughtful feedback on our manuscript. The comments provided have been highly constructive and have meaningfully strengthened the depth, clarity, and organization of our work.

In this revised submission, we have carefully addressed all reviewer comments through detailed, point-by-point responses. The manuscript has been revised accordingly to improve transparency, strengthen the integration of study-level findings, and enhance the overall coherence of the Results and Discussion sections.

We are pleased to resubmit the updated version of our manuscript to *PLOS ONE* and hope that the revisions adequately address the reviewers’ concerns. We are truly appreciative of the editorial and reviewer guidance in refining this work.

Sincerely,
Dr. Priyanka Lanka, MDS, MPH
NYU School of Global Public Health
[pl2461@nyu.edu](mailto:pl2461@nyu.edu)

**Reviewer #6: I believe that the author responds to the comments in the most concrete way as part of the review, and I have decided to respond in the same way.**

**REVIEWER COMMENT 1: REPORT KAPPA IN MANUSCRIPT**

**WHAT IS REQUESTED:** Describe the kappa or other values and interpret them in the manuscript; their absence is considered a methodological omission that must be corrected.

**WHAT THE AUTHOR DOES:** Declares an intentional decision NOT to report it in the manuscript and to keep it only as supplementary, justifying it by “limitations” and “paradoxes” of kappa .
It is necessary to inform the author that the aim is not to defend the use of the kappa coefficient, but rather to ensure a formal and transparent analysis of interobserver agreement. The use of kappa is suggested as a standardized and well-known tool. In this regard, the following alternative methodological strategies are proposed:

1.Use of Gwet's AC1 / AC2 as the main measure of agreement, due to its greater stability in the face of prevalence problems and marginal bias.
2.Krippendorff's Application alpha when multiple evaluators or complex categories are involved, given its greater methodological flexibility.
3.Combined report of absolute agreement percentage along with a robust metric (AC1 or alpha ), to improve interpretability.
4.Documented consensus award , with the participation of a third reviewer, as an integral part of the analytical process.

These strategies ensure methodological rigor and transparency, without depending on the kappa coefficient, and avoid confusing the discussion with a defense of a specific metric instead of an evaluation of the process; therefore, it is suggested that one of these be carried out.

***Author Response:*** *Thank you for this important and constructive comment. We appreciate the reviewer’s clarification that the primary objective is not to defend a specific statistic, but to ensure transparent and rigorous reporting of inter-reviewer agreement. We acknowledge that our prior response may have overemphasized methodological limitations of Cohen’s kappa rather than directly addressing the core issue of formal reporting. We apologize for any confusion this may have caused.*

*In the revised manuscript, we have taken the following steps to strengthen methodological transparency and align with the reviewer’s recommendations:*

1. ***Explicit Reporting of Inter-Rater Agreement in the Main Text****: We now report Cohen’s kappa coefficients directly within the Methods section of the manuscript, rather than limiting these results to the Supplementary Materials. The kappa values are presented alongside their interpretation using established benchmarks (e.g., slight, fair, moderate, substantial agreement), consistent with guidance from Cohen and widely accepted interpretation thresholds (e.g., Landis and Koch). This ensures that readers can directly assess the level of agreement achieved during screening.*
2. ***Clarification of the Consensus Adjudication Process****: We have expanded the description of our discrepancy resolution procedures to document the structured consensus process. Specifically, disagreements between the two primary reviewers were resolved through discussion, and when consensus could not be reached, a third senior reviewer adjudicated the decision. This process is now clearly described as an integral component of the analytical workflow, reinforcing the rigor of study selection.*
3. ***Supplementary Transparency****: Detailed inter-reviewer agreement statistics, including stage-specific reliability scores generated through Covidence, remain available in Supplementary Table 2 (Stable 2). These supplementary materials provide additional granularity without overburdening the main text.*

*While we continue to recognize the well-documented limitations of kappa under conditions of prevalence imbalance, our revisions ensure formal reporting of agreement metrics within the manuscript while maintaining clarity about how discrepancies were resolved.*

*For clarity, the following text has been added to the Methods section (Lines 224–231): “Inter-Rater Agreement
Inter-rater reliability during the title and abstract screening stages was assessed using Cohen’s kappa coefficient, calculated for reviewer pairs to quantify agreement beyond chance. Kappa values were interpreted using established benchmarks (e.g., slight, fair, moderate, substantial agreement), and proportionate (raw) agreement was also examined to aid interpretation, particularly in the presence of highly imbalanced inclusion decisions. Discrepancies were resolved through consensus discussion, with adjudication by a third reviewer.”*

*We believe these revisions address the reviewer’s concern by ensuring methodological rigor, transparency, and clarity in the reporting of inter-reviewer agreement.*

**REVIEWER COMMENT 2: LACK OF STUDY-LEVEL RESULTS IN THE TEXT.**

**WHAT IS REQUESTED:** that the narrative text be less concise and that key results be indicated for each study or more clearly integrated into tables/text.

WHAT THE AUTHOR DOES: The author primarily rejects the change, stating that there is “already” enough detail and that adding more text would be redundant; they defend maintaining the current “balance” and even “defer to the editor” as a stylistic preference. However, there is a rigor in the way the results should be presented; this is defended in the context of standard manuscript writing.

***Author Response:*** *We thank the reviewer for their thoughtful suggestion regarding the integration of key study results into the narrative text. We would like to note that all primary findings from each study are already comprehensively captured in our tables, which present detailed information on the implementation outcomes, study characteristics, and relevant metrics. The narrative text has been intentionally structured to highlight overarching patterns and trends rather than reiterate every individual data point, in order to maintain clarity and readability.*

*We believe that this approach preserves the rigor of the results section and aligns with standard practices in systematic review reporting, where detailed data are primarily summarized in tables while the text emphasizes synthesis and interpretation. Nonetheless, we have carefully reviewed the results narrative to ensure that major findings are clearly referenced and aligned with the tables, and we have added minor clarifications where necessary to guide readers without introducing redundancy. (see revised manuscript, (Lines 283-303).*

*We hope this approach balances completeness with readability and effectively conveys the key results of our review.*

**REVIEWER COMMENT 3: COMPARATIVE URBAN-RURAL ANALYSIS**

**WHAT IS REQUESTED:** a clearer comparison, with methodological justification, better integrating the “gap” "digital that divides" and the scarcity of rural studies in results and discussion.

**WHAT THE AUTHOR DOES:** explicitly rejects making the formal comparison and justifies it by insufficient evidence and risk of bias/ overinterpretation; states that "it would not be methodologically sound".

***Author Response:*** *We sincerely thank the reviewer for emphasizing the importance of urban–rural context and digital inequities in digital health implementation research. We agree that geographic setting and differential access to digital infrastructure are highly relevant factors that may influence the implementation and uptake of mHealth interventions.*

*To ensure this concern is adequately addressed, we have carefully reviewed and strengthened the manuscript in several areas when necessary. Geographic distribution and setting are explicitly reported in the Results section (Line 277, “II. Geographical Distribution and Setting”), with additional contextual references provided in Lines 456 and 522-526, as well as in the relevant summary tables. These revisions make the predominance of urban-based studies more visible and transparent to readers.*

*The distribution of study settings reflects the outcome of a predefined, protocol-driven search strategy and the consistent application of explicit inclusion and exclusion criteria. Among eligible studies, most were conducted in urban settings, with three in rural areas and one in a peri-urban context. This pattern reflects the current state of the published evidence rather than selection bias within our review process.*

*We acknowledge that urban–rural differences in connectivity, device ownership, and digital literacy may influence implementation outcomes. However, this review was not designed to formally assess the digital divide. Digital equity was not specified within the a priori search strategy, data extraction framework, or analytic plan, and included studies did not consistently report standardized indicators of digital access or infrastructure. As such, conducting a formal comparative analysis would risk overinterpretation of sparse and heterogeneous data.*

*To directly address the reviewer’s concern, we have strengthened the Strengths and Limitations (Line 510) to explicitly acknowledge: (1) the predominance of urban-based evidence, (2) the limited representation of rural settings, and (3) the need for future research to intentionally examine digital equity and contextual determinants of mHealth implementation.*

*We believe these revisions enhance transparency, appropriately contextualize our findings, and directly respond to the reviewer’s concern.*

**REVIEWER COMMENT 4: SUSTAINED EFFECT AND LIMITATION DUE TO LACK OF FOLLOW-UP**

**WHAT IS REQUESTED:** that the manuscript explicitly states that the absence of long-term follow-up limits the interpretation of the implementation results, in order to provide adequate context.

**WHAT THE AUTHOR DOES:** reframes the request as “out of scope” and justifies it by “conceptual limitations”; argues that “ long-term The " follow -up" refers to clinical evaluation, not implementation outcomes , and therefore it is not included as an outcome of the protocol.

(Note: It says it will add a clarification under discussion, but the text deviates as "not applicable")

***Author Response:*** *We thank the reviewer for emphasizing the importance of long-term follow-up in the interpretation of implementation outcomes. We agree that the duration of follow-up is critical for assessing sustainability, durability, and the continued effectiveness of implementation strategies over time.*

*In response to this comment, we have strengthened the manuscript by explicitly acknowledging this issue in the Strengths and Limitations section (line 499). We now clearly state that the absence of long-term follow-up across included studies limits the interpretation of sustained implementation outcomes. Specifically, most studies evaluated short- to medium-term indicators of acceptability, feasibility, and adoption, which constrains our ability to draw conclusions about long-term sustainability, maintenance, and scale-up potential of mHealth interventions in these contexts.*

*This clarification ensures that readers appropriately contextualize the findings and understand that observed implementation success may not necessarily translate into sustained impact over extended periods. By explicitly framing this limitation, we enhance transparency and align the interpretation of our synthesis with established principles of rigorous implementation research reporting.*

*The following text has been incorporated into the Limitations section (Lines 500–502): “Third, follow-up durations were generally short to medium term, limiting insight into long-term sustainability and durability of implementation outcomes.”*

*We believe this revision directly addresses the reviewer’s concern and strengthens the methodological transparency of the manuscript.*

**REVIEWER COMMENT 5: INTEGRATE CLINICAL OUTCOMES (CLINICAL RELEVANCE)**

**WHAT IS REQUESTED:** integrate clinical endpoints / do not treat everything as if it had the same weight when the clinical aspect is central to relevance.

**WHAT THE AUTHOR DOES:** admits that he extracted clinical outcomes, but decides NOT to integrate them into the main narrative, justifying that it would shift the emphasis away from the “ implementation-focused ” objective, and leaves them in a supplementary table.

***Author Response:*** *We thank the reviewer for emphasizing the importance of clinical relevance when interpreting mHealth interventions. We fully agree that clinical outcomes—such as blood pressure reduction and related cardiovascular endpoints—are essential for determining the ultimate public health impact of these interventions.*

*As part of our data extraction process, clinical outcomes were systematically collected and are presented in Supplementary Table 1. However, consistent with our predefined objective and registered protocol, this review was designed to synthesize implementation science outcomes—specifically acceptability, feasibility, and adoption—guided by Proctor’s implementation outcomes framework. Therefore, clinical endpoints were not weighted equivalently in the primary narrative synthesis, as our central research question focused on implementation performance rather than clinical effectiveness.*

*Importantly, we recognize that implementation outcomes and clinical outcomes are complementary but analytically distinct domains. Implementation outcomes help determine whether an intervention can be successfully delivered, adopted, and integrated within real-world systems, whereas clinical outcomes assess its therapeutic impact. Both domains are critical for informing policy and scale-up decisions, but they address different stages of the translational pathway.*

*To ensure clarity and address the reviewer’s concern, we have revised the manuscript to explicitly state that clinical findings are reported alongside implementation outcomes in Stable 1. We believe this clarification strengthens the transparency of our analytic approach while maintaining alignment with the predefined scope of the review.*

**REVIEWER COMMENT 6: RANKING/PRIORITIZATION OF BARRIERS AND FACILITATORS**

**WHAT IS REQUESTED:** to rank or prioritize barriers/facilitators (for greater applied utility).

**WHAT THE AUTHOR DOES:** does not make the ranking and justifies it as subjective and potentially biased in heterogeneous contexts; maintains a descriptive approach.

The central problem with this work is that it dismisses clinical relevance as the very foundation of the research it seeks to justify; in other words, it dismisses the applicability of the work. As a public health researcher... And as a clinical physician, I believe that in the context of a high-impact chronic disease such as hypertension arterial, to perform an analysis in which it is deliberately declared “narrative” and “conceptual” not It manages to substantively integrate clinical outcomes but falls into a recurring vice of contemporary science: the production of self-referential knowledge, methodologically coherent but socially sterile .

The acceptability, feasibility, or adoption of the proposed model is meaningless if it is not linked to its actual capacity to modify blood pressure control, prevent cardiovascular events, or reduce disease burden. These aspects are only mentioned. Within the theoretical framework and taxonomies without clinical anchoring, the study substitutes impact for classification and evidence for description, which limits its falsifiability, its usefulness for decision-makers, and its transformative potential in communities facing a disproportionate burden of disease.

The title mentions Hypertension Management in African In adults, management corresponds to a clinical intervention with a measurable outcome. I believe the title of the work should be modified to "digital education" or "digital use, not management."
This approach not only weakens the scientific contribution of the manuscript, but also reproduces a form of research that prioritizes academic exercise over the ethical responsibility to generate actionable knowledge with real impact on public health.

***Author Response:*** *We sincerely thank the reviewer for their thoughtful reflection on the clinical relevance of this work. While we respectfully differ from the reviewer’s narrower framing of hypertension management solely in terms of clinical outcomes, we fully agree that, given the high burden of hypertension across African settings, clinical measures such as blood pressure control, treatment adherence, and cardiovascular risk reduction are critical. Achieving meaningful public health impact, however, requires not only clinical efficacy but also the broad implementation of evidence-based interventions. Foundational work by Balas and Boren (2000, Managing Clinical Knowledge for Health Care Improvement) and others (Ramly E, Brown HW. Beyond Effectiveness: Implementation Science 101 for Clinicians and Clinical Researchers. Urogynecology (Phila). 2023 Mar 1;29(3):307-312.; Kelly, B. (2017). Implementation science: Applying the evidence of effectiveness in real-world contexts. Frameworks for practice in educational psychology: A textbook for trainees and practitioners, 78-94.) provides the conceptual basis for implementation science, highlighting how implementation research is essential for translating clinical knowledge into public health impact.*

*This systematic review was intentionally designed to address a complementary question: how mHealth interventions for hypertension are implemented in real-world African contexts. Our primary focus was on synthesizing implementation outcomes—acceptability, feasibility, and adoption—guided by Proctor’s implementation outcomes framework. While clinical effectiveness and implementation performance are interdependent, they represent analytically distinct domains that require separate but coordinated evaluation.*

*In high-burden, resource-constrained settings, evidence of clinical efficacy alone is insufficient to guide scale-up. Interventions must also demonstrate consistent deliverability, acceptability to patients and providers, and integration into existing health systems. By emphasizing implementation outcomes, this review addresses a critical translational gap between efficacy and sustainable scale-up.*

*To address the reviewer’s concern regarding framing and emphasis, we have:*

1. *Revised the title to explicitly reflect the implementation-focused scope of the review.*
2. *Ensured transparent reporting of clinical outcomes, systematically extracted and presented in Supplementary Table 1 to provide contextual interpretation.*

*We believe that clearly distinguishing between clinical effectiveness and implementation performance strengthens the methodological coherence of the manuscript while preserving its clinical and policy relevance. This clarification ensures readers understand both the scope and the contribution of the review in informing effective and scalable mHealth strategies in diverse African contexts.*
