# Supplementary material for: Mobile health interventions for African adults with hypertension: Evidence from a systematic review of implementation research outcomes
Source: PLOS Digit Health. 2026 Aug 14;5(8):e0001612. doi: 10.1371/journal.pdig.0001612 (PMC13475905; doi:10.1371/journal.pdig.0001612)
Supplement: Peer review history

# Peer review: Decision Letter 0

**DOI:** 10.1371/journal.pdig.0001612.r001

**Authors:** Haleh Ayatollahi

10 Jun 2024

PDIG-D-24-00124

Determining Acceptability, Feasibility, and Adoption rates associated with Mobile Health interventions for hypertension management among adults in Africa - A Systematic Review

PLOS Digital Health

Dear Dr. Lanka,

Thank you for submitting your manuscript to PLOS Digital Health. After careful consideration, we feel that it has merit but does not fully meet PLOS Digital Health's publication criteria as it currently stands. Therefore, we invite you to submit a revised version of the manuscript that addresses the points raised during the review process.

Please submit your revised manuscript within 60 days Aug 09 2024 11:59PM. If you will need more time than this to complete your revisions, please reply to this message or contact the journal office at digitalhealth@plos.org. When you're ready to submit your revision, log on to https://www.editorialmanager.com/pdig/ and select the 'Submissions Needing Revision' folder to locate your manuscript file.

Please include the following items when submitting your revised manuscript:

*A rebuttal letter that responds to each point raised by the editor and reviewer(s). You should upload this letter as a separate file labeled 'Response to Reviewers'.

*A marked-up copy of your manuscript that highlights changes made to the original version. You should upload this as a separate file labeled 'Revised Manuscript with Track Changes'.

*An unmarked version of your revised paper without tracked changes. You should upload this as a separate file labeled 'Manuscript'.

If you would like to make changes to your financial disclosure, please include your updated statement in your cover letter. Guidelines for resubmitting your figure files are available below the reviewer comments at the end of this letter.

We look forward to receiving your revised manuscript.

Kind regards,

Haleh Ayatollahi

Section Editor

PLOS Digital Health

Journal Requirements:

Additional Editor Comments (if provided):

[Note: HTML markup is below. Please do not edit.]

Reviewers' comments:

Reviewer's Responses to Questions

**Comments to the Author**

1. Does this manuscript meet PLOS Digital Health’s publication criteria?

Reviewer #1: Yes

Reviewer #2: Yes

--------------------

2. Has the statistical analysis been performed appropriately and rigorously?

Reviewer #1: N/A

Reviewer #2: Yes

--------------------

3. Have the authors made all data underlying the findings in their manuscript fully available (please refer to the Data Availability Statement at the start of the manuscript PDF file)?

The PLOS Data policy

Reviewer #1: Yes

Reviewer #2: Yes

--------------------

4. Is the manuscript presented in an intelligible fashion and written in standard English?<br/><br/>PLOS Digital Health does not copyedit accepted manuscripts, so the language in submitted articles must be clear, correct, and unambiguous. Any typographical or grammatical errors should be corrected at revision, so please note any specific errors here.

Reviewer #1: Yes

Reviewer #2: Yes

--------------------

Reviewer #1: Please find the attached my review comments in a word document under Reviewer attachment section.

Thank you for the opportunity to review this manuscript "a systematic review to determine the acceptability, feasibility, and adoption rates of mHealth intervention among adults diagnosed with hypertension across African countries"

Reviewer #2: Manuscript Review

Manuscript Number: PDIG-D-24-00124.

Title: Determining Acceptability, Feasibility, and Adoption rates associated with Mobile

Health interventions for hypertension management among adults in Africa - A

Systematic Review

General Comments: The present paper addresses the utility of mobile health technologies as interventions in the management of hypertension in Africa. The paper is a systematic review which has sourced 9 databases with keywords of hypertension, blood pressure, mobile health and implementation outcomes. 650 articles were identified of which 60 were advanced for this revIew, which upon further detailed review by the authors 18 were ultimately found to be relevant and utilized in the present paper. These papers were then visually outlined utilizing the PRISMA flow chart as a systematic means of reporting particular data and variables. In particular, feasibility, acceptability and adoption we're highlighted. Following data extraction the authors contributed in providing detailed presentation in table form, followed by discussion, of the facilitators and barriers to implementation of mHealth technology. Papers utilized were also assessed as to the quality in terms of content bias. In general, the review presents significant information and highlights remaining limitations which need to be overcome in order to facilitate the utility of mHealth and phone-based technologies as a means of making a difference in hypertension management in Africa. In general, this is a well done study and a well-written paper which addresses a clinical disease state of major significance.

To enhance the paper the following issues and suggestions are raised and should be addressed:

1. While this is a systematic review the paper would still benefit from including a hypothesis up front in the introduction. The utility of this is that it enhances the overall value for readers in leading to the direction of purpose and future research, rather than simply analyzing data. So, suggesting something along the order of “widespread utilization and adoption of mHealth technologies may or will facilitate the management of hypertension. Here we sought to ascertain the degree of present efficacy utilizing a systematic review….” would set the paper up in a better way.

It would also be useful to state exactly what questions or aims are being explored in this review rather than simply stating that this is a review. The way stated presently it leaves the reader to guess.

2. In the abstract a range of acronyms and abbreviations are introduced. It would be useful to provide explanation of abbreviations, at least for LMICS.

3. In the abstract it would be useful to expand the first method sentence to explain more clearly is this a published paper review or did you do actual perform data analysis. It is not clear for the reader. As this is right up front and what most people (only, sadly) read greater clarity is needed here.

4. The introduction is well written but a bit too wordy. Being more concise would be much more useful.

5. In the methods it would be useful to state explicitly whether or not a large language model or other AI system was utilized in this paper. In the current world in which we live with the increasing use of AI and people questioning the veracity of information stating this is very important.

6. In the results. In description of information extracted throughout the section “characteristics of identified studies” qualitative descriptor terms such as “most” studies or “some” studies are often utilized. This these terms are too general. It would be much better to state the number divided by the denominator of studies such as four out of eleven (4/11) or three out of seven (3/7). Using “most” or “some” inadvertently adds interpretive bias, which is something the authors have worked hard to define in the basal studies, so utilizing this approach is much cleaner. Finally, if you decide to continue to use most, often and frequently it is good to add the numbers next to these descriptors as has been illustrated here.

7. In section V. Study design and technological approaches. Here again it would be cleaner instead of saying 44% of randomized controlled trials, since the numbers are small, it would be better to state X over the denominator and the percentage. i.e. X/Y Studies (44%).

8. In the discussion it would be useful to provide insights and suggestions as to means of assessing the impact of the mHealth and digital interventions that have been revealed. Some of this is done in the third paragraph of the discussion though this could be expanded and would add value for the reader.

9. In that discussion section, i.e. in the 3rd and 4th paragraph it might be useful to comment on the speed it has taken for any change to occur with any of the mHealth interventions utilized as to having impact on HTN management. How quickly did this alter hypertensive management? And similarly, to comment on what the recidivism or recrudescence rates were despite utilizing these technologies? - if that information was available from any of the studies reviewed.

10. It would be useful in the conclusion or the end of the discussion to provide for the reader the authors perspective on some expanded next steps. There are only one or two sentences that address this. Again, tying this to an upfront hypothesis one might suggest that additional studies be done related to the combination of continued education along with economic efforts to enhance phone and SMS use and understanding in patients across an educational spectrum or enhanced education related to the importance of hypertension as a silent disease would be useful. In this reviewer's eyes reviews are useful to provide data but also should be suggestive of next steps. Adding a few sentences here would be valuable for broad readership.

--------------------

what does this mean?). If published, this will include your full peer review and any attached files.

**Do you want your identity to be public for this peer review?**  If you choose “no”, your identity will remain anonymous but your review may still be made public.

For information about this choice, including consent withdrawal, please see our Privacy Policy

Reviewer #1: Yes: Mahmoud Al Ammouri

Reviewer #2: Yes: Marvin J. Slepian

--------------------

[NOTE: If reviewer comments were submitted as an attachment file, they will be attached to this email and accessible via the submission site. Please log into your account, locate the manuscript record, and check for the action link "View Attachments". If this link does not appear, there are no attachment files.]

While revising your submission, please upload your figure files to the Preflight Analysis and Conversion Engine (PACE) digital diagnostic tool, https://pacev2.apexcovantage.com/. PACE helps ensure that figures meet PLOS requirements. To use PACE, you must first register as a user. Registration is free. Then, login and navigate to the UPLOAD tab, where you will find detailed instructions on how to use the tool. If you encounter any issues or have any questions when using PACE, please email PLOS at figures@plos.org. Please note that Supporting Information files do not need this step.

Attachment Submitted filename: PLOS Reviewer comments.docx

Attachment Submitted filename: PDIG-D-24-00124.docx

# Author comment: Author response to Decision Letter 1

**DOI:** 10.1371/journal.pdig.0001612.r002

12 Sep 2024

Attachment Submitted filename: Response to Reviewers.docx

# Peer review: Decision Letter 1

**DOI:** 10.1371/journal.pdig.0001612.r003

**Authors:** Haleh Ayatollahi

17 Dec 2024

Response to Reviewers Revised Manuscript with Track Changes Manuscript **Journal Requirements:**

A numbered table of all studies identified in the literature search, including those that were excluded from the analyses.

For every excluded study, the table should list the reason(s) for exclusion.

If any of the included studies are unpublished, include a link (URL) to the primary source or detailed information about how the content can be accessed.

A table of all data extracted from the primary research sources for the systematic review and/or meta-analysis. The table must include the following information for each study:

Name of data extractors and date of data extraction

Confirmation that the study was eligible to be included in the review.

All data extracted from each study for the reported systematic review and/or meta-analysis that would be needed to replicate your analyses.

If data or supporting information were obtained from another source (e.g. correspondence with the author of the original research article), please provide the source of data and dates on which the data/information were obtained by your research group.

If applicable for your analysis, a table showing the completed risk of bias and quality/certainty assessments for each study or outcome. Please ensure this is provided for each domain or parameter assessed. For example, if you used the Cochrane risk-of-bias tool for randomized trials, provide answers to each of the signalling questions for each study. If you used GRADE to assess certainty of evidence, provide judgements about each of the quality of evidence factor. This should be provided for each outcome.

An explanation of how missing data were handled.

This information can be included in the main text, supplementary information, or relevant data repository. Please note that providing these underlying data is a requirement for publication in this journal, and if these data are not provided your manuscript might be rejected.

**Additional Editor Comments (if provided):** **Reviewers' Comments:**

**Comments to the Author**

Reviewer #2: All comments have been addressed

Reviewer #3: (No Response)

**********

publication criteria?

Reviewer #2: Yes

Reviewer #3: Partly

**********

3. Has the statistical analysis been performed appropriately and rigorously?

Reviewer #2: N/A

Reviewer #3: (No Response)

**********

4. Have the authors made all data underlying the findings in their manuscript fully available (please refer to the Data Availability Statement at the start of the manuscript PDF file)?

The PLOS Data policy

Reviewer #2: Yes

Reviewer #3: (No Response)

**********

5. Is the manuscript presented in an intelligible fashion and written in standard English?

Reviewer #2: Yes

Reviewer #3: (No Response)

**********

Reviewer #2: The authors have adequately addressed the prior critiques

Reviewer #3: Thank you for addressing the previous reviewers' comments. Here are a few more minor comments that I believe may enhance your work,

1. I suggest including a table of Procter's feasibility outcomes with the definitions to aid reviewers not familiar with the lexicon.

2. Page 21 Table 2, please review text in certain rows and clarify what you mean. For instance, the first barrier merely states: "General belief in symptoms of hypertension poses a barrier [32]." The third facilitator: Implementation of adherence interventions with behavior change theories, considering the complex mix of psychosocial and health service influences on adherence behaviors [28].

3. My main comment is that the discussion still requires revision to enhance the value of the manuscript. Specifically, the organizational framework needs some work to make the manuscript useful to the audience. For instance, page 25 Par 1 "Hence, incorporating educational components to address these beliefs are necessary, as it directly impacts the feasibility and effectiveness of implementing mHealth solutions to address this condition" is quite out of place in the flow of the discussion.

4. Table 1 might be a better summary if the findings of the studies were summarized instead of merely stating which outcomes were studied (e. feasibility).

**********

what does this mean?). If published, this will include your full peer review and any attached files.

**Do you want your identity to be public for this peer review?**  If you choose “no”, your identity will remain anonymous but your review may still be made public.

For information about this choice, including consent withdrawal, please see our Privacy Policy

Reviewer #2: **Yes:**  Marvin J. Slepian

Reviewer #3: No

**********

**Figure resubmission:** **Reproducibility:**

To enhance the reproducibility of your results, we recommend that authors of applicable studies deposit laboratory protocols in protocols.io, where a protocol can be assigned its own identifier (DOI) such that it can be cited independently in the future. Additionally, PLOS ONE offers an option to publish peer-reviewed clinical study protocols. Read more information on sharing protocols at https://plos.org/protocols?utm_medium=editorial-email&utm_source=authorletters&utm_campaign=protocols

# Author comment: Author response to Decision Letter 2

**DOI:** 10.1371/journal.pdig.0001612.r004

23 Feb 2025

Attachment Submitted filename: Response to Reviewers.pdf

# Peer review: Decision Letter 2

**DOI:** 10.1371/journal.pdig.0001612.r005

**Authors:** Haleh Ayatollahi

6 Apr 2025

Response to ReviewersRevised Manuscript with Track ChangesManuscript**Journal Requirements:** **Additional Editor Comments (if provided):** **Reviewers' Comments:**

**Comments to the Author**

Reviewer #4: All comments have been addressed

Reviewer #5: All comments have been addressed

**********

publication criteria?

Reviewer #4: Yes

Reviewer #5: Yes

**********

3. Has the statistical analysis been performed appropriately and rigorously?

Reviewer #4: Yes

Reviewer #5: N/A

**********

4. Have the authors made all data underlying the findings in their manuscript fully available (please refer to the Data Availability Statement at the start of the manuscript PDF file)?

The PLOS Data policy

Reviewer #4: Yes

Reviewer #5: Yes

**********

5. Is the manuscript presented in an intelligible fashion and written in standard English?

Reviewer #4: Yes

Reviewer #5: Yes

**********

Reviewer #4: Formatting and titles of the tables is not correct for a scientific article

The introduction should have more information about the use of platforms. It focus too much on hypertension and not enough on the management platform.

Reviewer #5: The manuscript by Lanka et al addresses the usage of mobile health solutions (mHealth) for hypertension in Africa by means of a careful literature review of 18 articles. Generally the manuscript is clearly written and the arguments fairly sound. However, my major concern is that the authors draw conclusions from on 18 studies. What is more, the manuscript is too detailed.

1) I suggest that the authors relate much of the material from the current manuscript to the supplementary document. For example, Table 1 is neither news nor specific to the manuscript. Therefore, I suggest to simply refer to the relevant reference instead of spelling out the definitions according to reference #17.

2) Similarly, there are too many (and too short) subsections in the Methods section.

3) Tables are generally too many and too detailed. They do not assists the reader in acquiring an overview of the covered material.

4) Finally, I kindly ask the authors to be more concise and precise at that. For example, it is not entirely clear how the 18 references allow the authors to conclude that mHealth has gained traction across three countries.

**********

what does this mean?). If published, this will include your full peer review and any attached files.

**Do you want your identity to be public for this peer review?**  If you choose “no”, your identity will remain anonymous but your review may still be made public.

For information about this choice, including consent withdrawal, please see our Privacy Policy

Reviewer #4: No

Reviewer #5: **Yes:**  Amalie Dahl Haue

**********

**Figure resubmission:** **Reproducibility:**

To enhance the reproducibility of your results, we recommend that authors of applicable studies deposit laboratory protocols in protocols.io, where a protocol can be assigned its own identifier (DOI) such that it can be cited independently in the future. Additionally, PLOS ONE offers an option to publish peer-reviewed clinical study protocols. Read more information on sharing protocols at https://plos.org/protocols?utm_medium=editorial-email&utm_source=authorletters&utm_campaign=protocols

Attachment Submitted filename: PDIG-D-24-00124_reviewer (1).pdf

# Author comment: Author response to Decision Letter 3

**DOI:** 10.1371/journal.pdig.0001612.r006

4 Jun 2025

Attachment Submitted filename: Response_to_reviewers_auresp_3.docx

# Peer review: Decision Letter 3

**DOI:** 10.1371/journal.pdig.0001612.r007

**Authors:** Haleh Ayatollahi

8 Jul 2025

Response to ReviewersRevised Manuscript with Track ChangesManuscript**Journal Requirements:** **Additional Editor Comments (if provided):** **Reviewers' Comments:**

**Comments to the Author**

Reviewer #2: All comments have been addressed

Reviewer #6: All comments have been addressed

**********

publication criteria?

Reviewer #2: Yes

Reviewer #6: Yes

**********

3. Has the statistical analysis been performed appropriately and rigorously?

Reviewer #2: Yes

Reviewer #6: No

**********

4. Have the authors made all data underlying the findings in their manuscript fully available (please refer to the Data Availability Statement at the start of the manuscript PDF file)?

The PLOS Data policy

Reviewer #2: Yes

Reviewer #6: Yes

**********

5. Is the manuscript presented in an intelligible fashion and written in standard English?

Reviewer #2: Yes

Reviewer #6: Yes

**********

Reviewer #2: Thank you for addressing the raised concerns

Reviewer #6: I consider this study to be quite relevant in understanding the application of health technology in developing countries. However, I believe the following points need to be addressed:

Suggested Reformulation of Objectives:

General Objective:

To analyze the acceptability, feasibility, and adoption of mobile health (mHealth) interventions for the management of hypertension in adults in Africa through a systematic review.

Methodological Issues:

The study is registered as a systematic review, but although the registration date is mentioned, the full search strategy is not attached.

The search strategy is extensive; however, the manuscript does not report whether double reviewers were used or how the review process was carried out (e.g., selection, conflict resolution).

The justification for excluding pregnant women is weak, especially given the high rate of hypertensive disorders in pregnancy throughout Africa. A justification such as ethical concerns or the transient nature of the condition could strengthen this decision.

Interobserver reliability (e.g., kappa coefficient) for risk of bias assessment is not reported.

The use of the ASSESS tool is appropriate, but there is no detailed discussion of quality by criterion. It is recommended that a table of methodological limitations be added per study.

Results:

Results should be more clearly linked to specific studies; currently, study-level findings are inconsistently described.

There is no exploration of variations between rural and urban settings, which is essential given the digital divide in African contexts.

All outcomes are presented with equal weight, without distinguishing which interventions demonstrated greater clinical or implementation benefits.

The review reports perceived changes in adherence, but there is no assessment of hard clinical outcomes, such as blood pressure control or cardiovascular events.

No follow-up data or duration of effect are discussed or analyzed.

The table of barriers and facilitators, although useful, is too long and not ranked. For example, facilitators such as increased mobile phone use are mentioned, but they are not categorized according to their level of impact.

Recommendations:

Clearly specify the clinical outcomes assessed, including the duration of hypertension, the magnitude of improvement in adherence, and any clinically significant blood pressure reduction.

If clinical outcomes were not reported in the studies, authors should clearly state this and highlight this as a research gap.

Consider reorganizing the barriers and facilitators into shorter, thematically grouped tables, and indicate how many studies reported each item.

**********

what does this mean?). If published, this will include your full peer review and any attached files.

**Do you want your identity to be public for this peer review?**  If you choose “no”, your identity will remain anonymous but your review may still be made public.

For information about this choice, including consent withdrawal, please see our Privacy Policy

Reviewer #2: No

Reviewer #6: **Yes:**  Adan Pacifuentes Orozco

**********

**Figure resubmission:** **Reproducibility:**

To enhance the reproducibility of your results, we recommend that authors of applicable studies deposit laboratory protocols in protocols.io, where a protocol can be assigned its own identifier (DOI) such that it can be cited independently in the future. Additionally, PLOS ONE offers an option to publish peer-reviewed clinical study protocols. Read more information on sharing protocols at https://plos.org/protocols?utm_medium=editorial-email&utm_source=authorletters&utm_campaign=protocols

# Author comment: Author response to Decision Letter 4

**DOI:** 10.1371/journal.pdig.0001612.r008

3 Nov 2025

Attachment Submitted filename: Response_to_reviewers_auresp_4.docx

# Peer review: Decision Letter 4

**DOI:** 10.1371/journal.pdig.0001612.r009

**Authors:** Haleh Ayatollahi

27 Nov 2025

Response to ReviewersRevised Manuscript with Track ChangesManuscript**Journal Requirements:** **Additional Editor Comments (if provided):** **Reviewers' Comments:**

**Comments to the Author**

Reviewer #6: All comments have been addressed

**********

publication criteria?

Reviewer #6: Yes

**********

3. Has the statistical analysis been performed appropriately and rigorously?

Reviewer #6: No

**********

4. Have the authors made all data underlying the findings in their manuscript fully available (please refer to the Data Availability Statement at the start of the manuscript PDF file)?

The PLOS Data policy

Reviewer #6: Yes

**********

5. Is the manuscript presented in an intelligible fashion and written in standard English?

Reviewer #6: Yes

**********

Reviewer #6: Corrections Still Needed

Some previously identified issues in the manuscript have been addressed; however, several methodological concerns remain unresolved.

a) Lack of reporting of the kappa coefficient

Although the authors state in their response that Covidence generated kappa values, these are never described or interpreted in the manuscript. Inter-observer reliability was specifically requested by reviewers, and its absence must be considered a methodological omission that requires correction.

b) Lack of study-level results

While the authors mention that individual study outcomes are presented “in tables,” the narrative text continues to rely on general statements without indicating which study supports which findings. This narrative aggregation makes the analysis more difficult to follow. It is suggested to highlight key individual results directly in the text or incorporate them more clearly into the existing tables.

c) Insufficient justification for excluding pregnant women

The added explanation regarding hypertensive disorders of pregnancy does not fully resolve the original methodological concern. Clear exclusion criteria are required. The manuscript needs to justify the exclusion based not only on the distinct pathophysiology of pregnancy but also on the transient course of gestational hypertension and the need for different evaluation frameworks—criteria that align with systematic review standards rather than broad clinical arguments.

d) Lack of comparative analysis between urban and rural contexts

The manuscript briefly discusses the digital divide and the scarcity of rural studies, yet the integration of these observations into the Results and Discussion is limited and lacks methodological justification. A clearer comparative analysis is needed.

e) Limited discussion of the sustained effect of interventions

Although supplementary tables include clinical data, the manuscript does not explicitly state that the absence of long-term follow-up limits the interpretation of implementation outcomes. This point must be articulated to contextualize the findings appropriately.

2. Suggestions for Modification

The following recommendations may address the remaining gaps:

a) Incorporate the kappa values directly into the Methods or Results section, explaining their range and interpretation.

b) Link each synthesized finding to its specific source study. For example:

“Sarfo et al. (2019) reported that…” rather than using vague statements such as “recent studies showed…”.

c) Add a subsection titled “Differences Between Urban and Rural Settings.”

Even if the available evidence is limited, discussing how geographic context influences feasibility, acceptability, and adoption is essential.

3. Conclusion: the manuscript requires minor changes

Overall, the manuscript shows a solid methodological foundation and clear progress in addressing earlier concerns. Nevertheless, minor revisions are still needed to improve transparency, strengthen methodological justification, and enhance the clarity of the narrative synthesis. With these adjustments, the manuscript would be suitable for publication.

**********

what does this mean?). If published, this will include your full peer review and any attached files.

**Do you want your identity to be public for this peer review?**  If you choose “no”, your identity will remain anonymous but your review may still be made public.

For information about this choice, including consent withdrawal, please see our Privacy Policy

Reviewer #6: **Yes:**  Adan Pacifuentes Orozco

**********

**Figure resubmission:**

**Reproducibility:**

To enhance the reproducibility of your results, we recommend that authors of applicable studies deposit laboratory protocols in protocols.io, where a protocol can be assigned its own identifier (DOI) such that it can be cited independently in the future. Additionally, PLOS ONE offers an option to publish peer-reviewed clinical study protocols. Read more information on sharing protocols at https://plos.org/protocols?utm_medium=editorial-email&utm_source=authorletters&utm_campaign=protocols

# Author comment: Author response to Decision Letter 5

**DOI:** 10.1371/journal.pdig.0001612.r010

10 Dec 2025

Attachment Submitted filename: Response_to_Reviewers_auresp_5.docx

# Peer review: Decision Letter 5

**DOI:** 10.1371/journal.pdig.0001612.r011

**Authors:** Haleh Ayatollahi

30 Dec 2025

Response to ReviewersRevised Manuscript with Track ChangesManuscript**Journal Requirements:** **Additional Editor Comments (if provided):** **Reviewers' Comments:**

**Comments to the Author**

Reviewer #6: All comments have been addressed

**********

publication criteria?

Reviewer #6: Partly

**********

3. Has the statistical analysis been performed appropriately and rigorously?

Reviewer #6: No

**********

4. Have the authors made all data underlying the findings in their manuscript fully available (please refer to the Data Availability Statement at the start of the manuscript PDF file)?

The PLOS Data policy

Reviewer #6: Yes

**********

5. Is the manuscript presented in an intelligible fashion and written in standard English?

Reviewer #6: Yes

**********

Reviewer #6: I believe that the author responds to the comments in the most concrete way as part of the review, and I have decided to respond in the same way.

REVIEWER COMMENT 1: REPORT KAPPA IN MANUSCRIPT

WHAT IS REQUESTED: describe the kappa or other values and interpret them in the manuscript; their absence is considered a methodological omission that must be corrected.

WHAT THE AUTHOR DOES: declares an intentional decision NOT to report it in the manuscript and to keep it only as supplementary, justifying it by “limitations” and “paradoxes” of kappa .

It is necessary to inform the author that the aim is not to defend the use of the kappa coefficient, but rather to ensure a formal and transparent analysis of interobserver agreement. The use of kappa is suggested as a standardized and well-known tool . In this regard, the following alternative methodological strategies are proposed:

1.Use of Gwet's AC1 / AC2 as the main measure of agreement, due to its greater stability in the face of prevalence problems and marginal bias.

2.Krippendorff's Application alpha when multiple evaluators or complex categories are involved, given its greater methodological flexibility.

3.Combined report of absolute agreement percentage along with a robust metric (AC1 or alpha ), to improve interpretability.

4.Documented consensus award , with the participation of a third reviewer, as an integral part of the analytical process.

These strategies ensure methodological rigor and transparency, without depending on the kappa coefficient , and avoid confusing the discussion with a defense of a specific metric instead of an evaluation of the process; therefore, it is suggested that one of these be carried out.

REVIEWER COMMENT 2: LACK OF STUDY-LEVEL RESULTS IN THE TEXT.

WHAT IS REQUESTED: that the narrative text be less concise and that key results be indicated for each study or more clearly integrated into tables/text. WHAT THE AUTHOR DOES: The author primarily rejects the change , stating that there is “already” enough detail and that adding more text would be redundant; they defend maintaining the current “balance” and even “ defer to the editor” as a stylistic preference. However, there is a rigor in the way the results should be presented; this is defended in the context of standard manuscript writing.

REVIEWER COMMENT 4: COMPARATIVE URBAN-RURAL ANALYSIS

WHAT IS REQUESTED: a clearer comparison, with methodological justification, better integrating the “gap” "digital that divides" and the scarcity of rural studies in results and discussion.

WHAT THE AUTHOR DOES: explicitly rejects making the formal comparison and justifies it by insufficient evidence and risk of bias/ overinterpretation ; states that "it would not be methodologically sound".

REVIEWER COMMENT 5: SUSTAINED EFFECT AND LIMITATION DUE TO LACK OF FOLLOW-UP

WHAT IS REQUESTED: that the manuscript explicitly states that the absence of long-term follow-up limits the interpretation of the implementation results, in order to provide adequate context.

WHAT THE AUTHOR DOES: reframes the request as “out of scope” and justifies it by “conceptual limitations”; argues that “ long-term The " follow -up" refers to clinical evaluation, not implementation outcomes , and therefore it is not included as an outcome of the protocol.

(Note: It says it will add a clarification under discussion, but the text deviates as "not applicable")

REVIEWER COMMENT 8: INTEGRATE CLINICAL OUTCOMES (CLINICAL RELEVANCE)

WHAT IS REQUESTED: integrate clinical endpoints / do not treat everything as if it had the same weight when the clinical aspect is central to relevance.

WHAT THE AUTHOR DOES: admits that he extracted clinical outcomes , but decides NOT to integrate them into the main narrative , justifying that it would shift the emphasis away from the “ implementation-focused ” objective, and leaves them in a supplementary table.

REVIEWER COMMENT 9: RANKING/PRIORITIZATION OF BARRIERS AND FACILITATORS

WHAT IS REQUESTED: to rank or prioritize barriers/facilitators (for greater applied utility).

WHAT THE AUTHOR DOES: does not make the ranking and justifies it as subjective and potentially biased in heterogeneous contexts; maintains a descriptive approach.

The central problem with this work is that it dismisses clinical relevance as the very foundation of the research it seeks to justify; in other words, it dismisses the applicability of the work . As a public health researcher... And as a clinical physician, I believe that in the context of a high-impact chronic disease such as hypertension arterial, to perform an analysis in which it is deliberately declared “narrative” and “ conceptual” not It manages to substantively integrate clinical outcomes but falls into a recurring vice of contemporary science: the production of self-referential knowledge, methodologically coherent but socially sterile .

The acceptability, feasibility, or adoption of the proposed model is meaningless if it is not linked to its actual capacity to modify blood pressure control, prevent cardiovascular events, or reduce disease burden. These aspects are only mentioned. Within the theoretical framework and taxonomies without clinical anchoring, the study substitutes impact for classification and evidence for description, which limits its falsifiability, its usefulness for decision-makers, and its transformative potential in communities facing a disproportionate burden of disease.

The title mentions Hypertension Management in African In adults , management corresponds to a clinical intervention with a measurable outcome. I believe the title of the work should be modified to "digital education" or "digital use, not management."

This approach not only weakens the scientific contribution of the manuscript, but also reproduces a form of research that prioritizes academic exercise over the ethical responsibility to generate actionable knowledge with real impact on public health .

**********

what does this mean?). If published, this will include your full peer review and any attached files.

**Do you want your identity to be public for this peer review?**  If you choose “no”, your identity will remain anonymous but your review may still be made public.

For information about this choice, including consent withdrawal, please see our Privacy Policy

Reviewer #6: No

**********

**Figure resubmission:**

**Reproducibility:**

To enhance the reproducibility of your results, we recommend that authors of applicable studies deposit laboratory protocols in protocols.io, where a protocol can be assigned its own identifier (DOI) such that it can be cited independently in the future. Additionally, PLOS ONE offers an option to publish peer-reviewed clinical study protocols. Read more information on sharing protocols at https://plos.org/protocols?utm_medium=editorial-email&utm_source=authorletters&utm_campaign=protocols

# Author comment: Author response to Decision Letter 6

**DOI:** 10.1371/journal.pdig.0001612.r012

28 Feb 2026

Attachment Submitted filename: Response_to_Reviewers_auresp_6.docx

# Peer review: Decision Letter 6

**DOI:** 10.1371/journal.pdig.0001612.r013

**Authors:** Haleh Ayatollahi

4 Jun 2026

Response to Reviewers Revised Manuscript with Track Changes Manuscript **Journal Requirements:**

1. Please provide an Author Summary. This should appear in your manuscript between the Abstract (if applicable) and the Introduction, and should be 150–200 words long. The aim should be to make your findings accessible to a wide audience that includes both scientists and non-scientists. Sample summaries can be found on our website under Submission Guidelines:

https://journals.plos.org/digitalhealth/s/submission-guidelines#loc-parts-of-a-submission

**Additional Editor Comments (if provided):** **Reviewers' Comments:**

**Comments to the Author**

Reviewer #6: All comments have been addressed

**********

publication criteria?

Reviewer #6: Yes

**********

3. Has the statistical analysis been performed appropriately and rigorously?

Reviewer #6: I don't know

**********

4. Have the authors made all data underlying the findings in their manuscript fully available (please refer to the Data Availability Statement at the start of the manuscript PDF file)?

The PLOS Data policy

Reviewer #6: Yes

**********

5. Is the manuscript presented in an intelligible fashion and written in standard English?

Reviewer #6: Yes

**********

**Reviewer #6** : I thank the authors for the revisions made in response to the previous round of review. The manuscript has been improved substantially, including adding inter-rater agreement reporting, clearer description of the consensus adjudication process, description of the distribution of studies by setting, and acknowledgement of the limitations of the studies in terms of rural representation, short-to-medium follow-up, and lack of assessment of sustained implementation.

At this point, I find the manuscript borderline acceptable from a methodological perspective. I can only offer the following methodological refinements for final approval:

Clarify whether the Proctor framework was an inclusion criteria or an analytic classification framework.

Current methods suggest that studies were included if they ‘used Proctor’s taxonomy’. Methodologically, this should be noted because relevant hypertension mHealth studies might have been excluded if the studies had to use Proctor’s framework explicitly. The authors should clarify if eligible studies were required to report Proctor’s taxonomy or if the review team mapped reported outcomes to Proctor’s implementation outcomes during extraction.

Improve the interpretation of low and variable inter-rater agreement.

The manuscript now reports Cohen’s kappa and raw agreement, as it should. However, as the reported kappa values are low and variable, authors should briefly describe the impact on methodological confidence in the screening process and how consensus adjudication protected the validity of final inclusion decisions. No new analysis is needed, just a more transparent methodological interpretation of the implications of low agreement.

Improve the connection between implementation outcomes and clinical relevance at the level of the study.

As the review is about hypertension management, implementation outcomes (eg, acceptability, adoption and feasibility) should be interpreted in the context of available clinical indicators (eg, blood pressure control, treatment adherence, follow-up attendance or hypertension knowledge). The authors should not be expected to conduct a meta-analysis, but a brief synthesis of the studies that reported clinical indicators and whether these outcomes supported or limited the interpretation of implementation success should be included.

Barriers and Facilitators Prioritization: Barriers are ranked by severity. Facilitators are ranked by impact.

The manuscript identifies a number of barriers and facilitators, but the applied value of these would be increased if they were organized by frequency, recurrence across studies, and potential impact on implementation. If the evidence does not allow this it should not be presented as a formal weighted ranking For example, a descriptive prioritization would suffice, such as: most recurrent barriers, context dependent barriers, and barriers with greatest implications for scale-up.

Delimit the limits of the urban-rural interpretation.

Rural studies are scarce and the manuscript does not over-interpret urban–rural differences correctly. Nevertheless, a short descriptive comparative review should be provided, dividing urban, peri-urban and rural studies by outcome measures, intervention modality and barriers to feasibility. This would increase transparency while not implying statistical comparison.

Consider the effect of follow-up time on sustainability.

The manuscript now acknowledges that most of the studies had short to medium follow-up. The authors should go the extra mile and explicitly state that sustainability, durability and maintenance of mHealth implementation cannot be inferred from the current evidence. Hence, any claims on scalability or sustained implementation should be treated as preliminary.

How risk of bias influenced the synthesis

The manuscript reports low overall risk of bias. However, the authors should briefly mention if and how the risk of bias findings were used to interpret the evidence. For example, they should state whether studies with a high risk in any domain were interpreted cautiously, whether all studies were given equal weight in the narrative synthesis, or whether risk of bias was used only descriptively.

With these methodological clarifications, I am happy to recommend acceptance of the manuscript.

I referred to the methodological points still open in the manuscript: inclusion linked to Proctor, report of kappa/agreement, urban-rural distribution, clinical outcomes, follow-up and sustainability.

**********

what does this mean?). If published, this will include your full peer review and any attached files.

**Do you want your identity to be public for this peer review?**  If you choose “no”, your identity will remain anonymous but your review may still be made public.

For information about this choice, including consent withdrawal, please see our Privacy Policy

Reviewer #6: None

**********

**Figure resubmission:**

**Reproducibility:**

To enhance the reproducibility of your results, we recommend that authors of applicable studies deposit laboratory protocols in protocols.io, where a protocol can be assigned its own identifier (DOI) such that it can be cited independently in the future. Additionally, PLOS ONE offers an option to publish peer-reviewed clinical study protocols. Read more information on sharing protocols at https://plos.org/protocols?utm_medium=editorial-email&utm_source=authorletters&utm_campaign=protocols

# Author comment: Author response to Decision Letter 7

**DOI:** 10.1371/journal.pdig.0001612.r014

4 Jul 2026

Attachment Submitted filename: Response_to_Reviewers_auresp_7.docx

# Editor report: Decision Letter 7

**DOI:** 10.1371/journal.pdig.0001612.r015

**Authors:** Haleh Ayatollahi

8 Jul 2026

Mobile Health Interventions for African Adults with Hypertension: Evidence from a Systematic Review of Implementation Research Outcomes

PDIG-D-24-00124R7

Dear Dr Lanka,

We are pleased to inform you that your manuscript 'Mobile Health Interventions for African Adults with Hypertension: Evidence from a Systematic Review of Implementation Research Outcomes' has been provisionally accepted for publication in PLOS Digital Health.

Before your manuscript can be formally accepted you will need to complete some formatting changes, which you will receive in a follow-up email from a member of our team.

Please note that your manuscript will not be scheduled for publication until you have made the required changes, so a swift response is appreciated.

IMPORTANT: The editorial review process is now complete. PLOS will only permit corrections to spelling, formatting or significant scientific errors from this point onwards. Requests for major changes, or any which affect the scientific understanding of your work, will cause delays to the publication date of your manuscript.

If your institution or institutions have a press office, please notify them about your upcoming paper to help maximize its impact. If they'll be preparing press materials, please inform our press team as soon as possible -- no later than 48 hours after receiving the formal acceptance. Your manuscript will remain under strict press embargo until 2 pm Eastern Time on the date of publication. For more information, please contact digitalhealth@plos.org.

Thank you again for supporting Open Access publishing; we are looking forward to publishing your work in PLOS Digital Health.

Best regards,

Haleh Ayatollahi

Section Editor

PLOS Digital Health

***********************************************************

**Additional Editor Comments (if provided):**

**Reviewer Comments (if any, and for reference):**
